# Accurate Reproduction of 161 Small-Molecule Complex Crystal Structures using the EUDOC Program: Expanding the Use of EUDOC to Supramolecular Chemistry

**DOI:** 10.1371/journal.pone.0000531

**Published:** 2007-06-13

**Authors:** Qi Wang, Yuan-Ping Pang

**Affiliations:** Computer-Aided Molecular Design Laboratory, Mayo Clinic, Rochester, Minnesota, United States of America; Center for Genomic Regulation, Spain

## Abstract

EUDOC is a docking program that has successfully predicted small-molecule-bound protein complexes and identified drug leads from chemical databases. To expand the application of the EUDOC program to supramolecular chemistry, we tested its ability to reproduce crystal structures of small-molecule complexes. Of 161 selected crystal structures of small-molecule guest-host complexes, EUDOC reproduced all these crystal structures with guest structure mass-weighted root mean square deviations (mwRMSDs) of <1.0 Å relative to the corresponding crystal structures. In addition, the average interaction energy of these 161 guest-host complexes (−50.1 kcal/mol) was found to be nearly half of that of 153 previously tested small-molecule-bound protein complexes (−108.5 kcal/mol), according to the interaction energies calculated by EUDOC. 31 of the 161 complexes could not be reproduced with mwRMSDs of <1.0 Å if neighboring hosts in the crystal structure of a guest-host complex were not included as part of the multimeric host system, whereas two of the 161 complexes could not be reproduced with mwRMSDs of <1.0 Å if water molecules were excluded from the host system. These results demonstrate the significant influence of crystal packing on small molecule complexation and suggest that EUDOC is able to predict small-molecule complexes and that it is useful for the design of new materials, molecular sensors, and multimeric inhibitors of protein-protein interactions.

## Introduction

In 1990, a computer was used to screen 10,000 chemicals in the Cambridge Structural Database (CSD) [1], leading to the identification of a haloperidol analog capable of inhibiting HIV-1 and HIV-2 proteases with a *K*_i_ of ≈100 µM [2]. The screening was accomplished using a computer docking program, DOCK, that docked each chemical of the database into the active sites of the enzymes and evaluated the shape complementarity of the docked compound relative to the active sites. Inspired by this seminal work, the EUDOC program was devised to search for the specific conformations, positions, and orientations of two three-dimensional (3D) structures that permit the strongest nonbonded intermolecular interactions between the two. The EUDOC program uses docking algorithms that differ from those of DOCK [3]. It addresses molecular flexibility by using conformation selection and conformation substitution mechanisms that enable massively parallel computing [3]. EUDOC was devised to perform on a cluster of more than 300 loosely connected processors [3] and has recently been ported to the IBM Blue Gene/L supercomputer [4], [5]. This program has successfully predicted small-molecule-bound protein complexes and identified drug leads from chemical databases [6]–[12].

The EUDOC program is also efficient. In a computational screen of 23,426 chemicals (at a resolution of 1.0 Å translation and 10° of arc rotation) for inhibitors of a chymotrypsin-like cysteine protease of the severe acute respiratory syndrome–associated coronavirus, the EUDOC program is able to reduce the wall-clock time of the screen from 242 minutes using 396 Xeon processors (2.2 GHz) on a Beowulf cluster to 13 and 7 minutes using 2048 and 4096 PowerPC-440 processors (700 MHz) on Blue Gene/L, respectively [4], [5]. Because a large database can be divided into subsets, a sustained petaflops capability would be able to screen 23 million chemicals in about 10 minutes or to screen 200×5000 billion chemicals for one drug target in a year [4]. This capability offers the possibility of identifying inhibitors that are effective enough for in vivo testing, eliminating the need of medicinal chemistry to improve the efficiency of inhibitor leads identified by terascale computers [4]. In the context of this promise, we seek to extend the application of the EUDOC program to supramolecular chemistry.

Supramolecular chemistry deals with creation of a large molecule assembled with noncovalent bonding among small molecular units, in contrast to organic synthesis that involves breaking and making covalent bonds to create a new molecule [13]. Such noncovalent bonding is reversible and comprises hydrogen bonding, metal coordination, hydrophobic force, van der Waals force, π-π interaction, cation-π interaction, and/or long-range electrostatic interaction to assemble small molecules into a multimolecular complex. Supramolecular chemistry principles have been used to develop new materials, molecular sensors, and multimolecular complexes designed to disrupt protein-protein interactions.

To expand the application of the EUDOC program to supramolecular chemistry, we tested its ability to reproduce the crystal structures of small-molecule guest-host complexes. Previously we had tested the ability of the program to reproduce crystal structures of proteins in complex with small molecules and found that EUDOC reproduced 97% of 154 crystal structures using the bound conformations of both proteins and their small-molecule partners [3]. This success may not transfer to with small-molecule guest-host complexes such as a crown ether in complex with 4-nitrobenzene-1,2-diamine, however, because the binding pocket or cavity in a small-molecule host is not as well formed as that in a protein.

Herein we report the results of our docking studies with 161 selected crystal structures of small-molecule guest-host complexes using the EUDOC program. These results show that the program is able to reproduce all 161 crystal structures and that the average interaction energy of these small-molecule complexes (−50.1 kcal/mol) is nearly half of that of the 153 small molecule-bound protein complexes we studied in previous tests (−108.5 kcal/mol). The results also demonstrate the significant influence of crystal packing on small-molecule complex crystal structures and suggest that the EUDOC program is able to predict 3D structures of small-molecule guest-host complexes with reasonable reliability.

## Results

### Docking without consideration of the influence of crystal packing or structural waters

The ability of EUDOC to reproduce crystal structures of small-molecule guest-host complexes was evaluated with the following procedure. The guest and host molecules in the complex crystal structure were separated, and the guest structure was then docked back into the host structure by the EUDOC program. This docking process used translational and rotational increments of 1.0 Å and 10° of arc, respectively, and a docking box that was defined to enclose the guest structure in the guest-host complex crystal structure. Of many EUDOC-generated guest-host complexes, only the complex with the strongest interaction energy was compared to the corresponding crystal structure of the complex. In this comparison, the host portion of the EUDOC-generated complex was superimposed onto the host portion of the crystal structure, and the mass-weighted root mean square deviation (mwRMSD) of the guest portion between the two superimposed complexes was calculated. If the mwRMSD was <2.0 or 1.0 Å, the crystal structure of the complex was reproduced or accurately reproduced, respectively, by the EUDOC program [3]. Because the uncertainty in calculating the interaction energy using the EUDOC program was estimated to be 0.7 kcal/mol [3], occasionally, a few EUDOC-generated complexes were considered to have the strongest interaction energy and compared to the crystal structure thus resulting in multiple mwRMSDs, if their interaction energies differed from the strongest interaction energy by ≤0.7 kcal/mol. In that case, as long as one of the mwRMSDs was <2.0 or 1.0 Å, the crystal structure of the binary complex was reproduced or accurately reproduced, respectively, by the EUDOC program.

**Figure 1:**
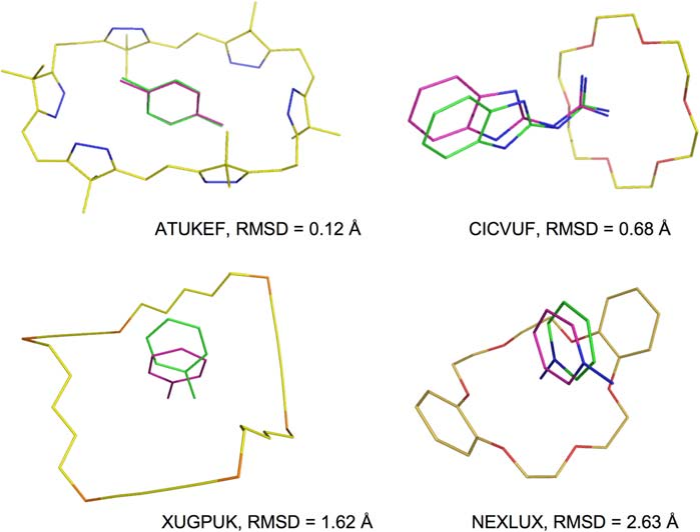
Overlays of the EUDOC-generated and crystal structures of complexes ATUKEF, CICVUF, XUGPUK, and NEXLUX. The C atoms of the EUDOC-generated and crystal structure are green and magenta, respectively. The O, N, and S atoms are red, blue and orange respectively.

A total of 161 crystal structures of small-molecule guest-host complexes were obtained from CSD for this study [1]. The selection criteria included the followings: (1) no covalent bond between a host and a guest; (2) the R factor of <15 to ensure good crystallographic quality; (3) a guest to host ratio of 1 in a unit cell; (4) no structures containing Ni^+2^, Ag^+^, Pd^+2^, Pt^+2^, Au^+^ or Ru^+2^ because force field parameters for these ions were unavailable in the EUDOC program. The results of the docking studies with the 161 guest-host complexes using the procedure described above are listed in Table 1. As apparent from the mwRMSD distribution listed in Table 2, the EUDOC program reproduced 93% and accurately reproduced 81% of the 161 complexes. The deviations between the EUDOC-generated and crystal complexes at different mwRMSD values are depicted in Figure 1.

**Table 1:** The intermolecular interaction energies of the 161 small-molecule complexes generated by the EUDOC program and their mass-weighted root mean square deviations (mwRMSDs) relative to their corresponding crystal structures.

CSD code^1^	E_total_ 2 (kcal/mol)	E_vdw_ 3 (kcal/mol)	E_ele_ 4 (kcal/mol)	mwRMSD (Å)	CSD code^1^	E_total_ 2 (kcal/mol)	E_vdw_ 3 (kcal/mol)	E_ele_ 4 (kcal/mol)	mwRMSD (Å)
ABELAU	−13.4	0.9	−14.4	0.21	HUNXUJ	−24.3	−5.0	−19.3	0.92
ABULOZ	−39.2	0.8	−40.0	0.01	HUNYAQ	−26.0	−8.2	−17.8	0.53
ACPHDR	−20.2	−17.9	−2.3	0.30	IKARUH	−19.1	−13.9	−5.2	0.44
AHOYEB	−31.1	−23.4	−7.7	0.89	IKUTOX	−11.4	−11.0	−0.4	0.97
AJUROM	−19.5	−15.6	−3.9	0.40	INUJAC^5^	−12.1	−8.8	−3.3	0.41
AJUXOS	−15.4	−8.3	−7.1	0.44	ITAMIZ	−17.9	−15.6	−2.3	0.56
AJUXUY	−21.5	−8.6	−13.0	0.23	IXEKAX	−36.6	−29.0	−7.6	0.08
AJUYAF	−19.6	−9.2	−10.4	0.35	IXEKEB	−28.3	−22.6	−5.6	0.06
ASOKIC	−13.0	−12.6	−0.3	1.65	JAXPON	−11.8	−9.9	−2.0	0.24
ATUKEF	−21.7	−20.2	−1.5	0.12	JEBTAL	−11.0	−10.4	−0.6	0.15
AWUGEE	−16.8	−4.5	−12.3	1.16	JEJWOK	−22.5	−20.4	−2.0	0.34
AXEZIM	−28.2	−9.7	−18.4	0.24	JESCAL	−32.6	−28.3	−4.4	0.64
AYIBEP	−256.4	7.7	−264.2	0.24	JIVMEG	−28.3	1.3	−29.6	0.23
BAFZEN	−128.0	3.0	−131.0	0.49	JIVMUW	−22.0	−4.4	−17.6	1.44
BAHDEU	−11.6	−10.6	−1.0	2.75	JULJAB	−11.0	−10.3	−0.7	2.54
BAKHIE^5^	−19.5	−8.0	−11.6	0.91	JUMYOF	−17.0	−17.0	0.0	1.52
BAMQAH	−23.9	−8.0	−15.9	0.52	KAXPOO	−12.0	−11.2	−0.7	0.83
BAPRAM	−17.9	−11.0	−6.9	0.74	KOHJAS^5^	−11.2	−8.0	−3.2	0.57
BAPREQ	−20.1	−11.2	−8.9	0.72	KOLMAZ	−59.0	−21.0	−38.0	0.08
BAXZAB	−31.5	−12.8	−18.8	0.20	LAYMAZ	−36.2	−10.0	−26.1	0.14
BAYXII	−45.4	−4.3	−41.1	0.24	LODNOH01	−9.9	−9.9	0.0	0.44
BECVEK	−15.7	−7.4	−8.3	0.33	MAFRAN^5^	−15.5	−12.3	−3.3	0.58
BEGVOZ	−51.4	−6.0	−45.4	1.54	MEXPEK	−9.2	−8.5	−0.7	1.08
BEVHER	−29.6	−24.4	−5.2	0.27	MNPOCB01	−37.5	−9.2	−28.3	0.11
BEVWAA	−27.9	−23.0	−4.9	0.55	MODTII	−116.2	−4.2	−112.0	0.31
BIFKIK	−50.3	−0.4	−50.0	0.17	MOZNIY	−10.5	−9.1	−1.5	0.30
BOHWUQ	−21.4	−19.5	−2.0	0.78	MUTFEM	−19.0	−17.2	−1.8	0.49
CACQED	−27.6	−19.5	−8.1	2.74	NEBQOA	−55.7	−31.3	−24.3	0.00
CAWRAT10	−52.3	−6.1	−46.2	0.39	NEPQUU	−16.0	−15.4	−0.6	0.52
CECMEC10	−26.9	−19.3	−7.5	0.48	NETKOM^5^	−13.1	−8.7	−4.5	1.54
CENHAE	−14.4	−4.6	−9.8	0.39	NETKOM01	−14.4	−10.0	−4.3	0.60
CICVUF	−16.7	−7.3	−9.4	0.68	NEXLUX	−23.0	−9.8	−13.2	2.63
CIXCOB	−23.8	−23.4	−0.4	0.06	NOHHOH	−58.6	−33.2	−25.3	0.98
COBTIW	−46.9	−5.4	−41.4	0.03	NOYNAQ	−16.6	−6.1	−10.5	1.36
COXLEG10	−20.6	3.9	−24.5	0.90	NUDHOJ	−45.0	−39.5	−5.5	0.81
COXQEL	−16.7	−15.6	−1.1	0.55	OBOHAO	−12.0	−10.7	−1.3	0.40
COYBOH	−18.3	−10.4	−7.9	0.23	OCAMIO	−14.1	−10.0	−4.1	1.12
CRAMCA10	−36.2	−0.4	−35.9	0.36	QAJKAN	−17.0	−14.2	−2.7	2.29
CRAMCB10	−38.5	1.9	−40.4	0.61	QAKNAR	−25.9	−7.2	−18.7	0.81
CRAMCC10	−32.5	−0.9	−31.6	0.17	QAKNIZ	−25.9	−4.4	−21.5	0.52
CUDXUU	−10.9	−10.4	−0.5	1.27	QAKNOF	−25.6	−6.1	−19.4	0.98
CYCBOB	−15.6	−7.7	−7.9	0.38	QATDIY	−30.8	−22.2	−8.6	0.24
CYCBOF11	−12.4	−7.7	−4.6	0.74	RABJIJ	−16.2	−15.2	−1.0	0.65
DENFOR	−13.5	−12.1	−1.4	0.04	RACKAH	−26.8	−12.2	−14.6	0.79
DERFUB	−9.8	−7.7	−2.1	3.08	RAHWED	−7.0	−6.8	−0.3	0.46
DESHEO	−37.7	−3.6	−34.1	0.53	RALQAW01	−17.0	−7.2	−9.8	0.54
DIZTIP	−13.9	−8.0	−5.9	0.58	RIBBUZ	−15.4	−4.6	−10.9	0.41
DOXWAO	−41.6	−7.3	−34.3	0.83	RUYWIR	−32.0	−26.4	−5.6	0.06
DUGGUH10	−30.3	−2.2	−28.0	0.24	SAKTII	−57.5	−29.5	−28.0	0.31
DUKHUM	−49.5	−8.5	−41.1	0.33	SEPKON	−20.6	−2.9	−17.7	0.35
EBASEF	−13.7	−12.9	−0.8	1.09	SEPNEG	−20.7	−3.6	−17.1	0.97
EGIRIV	−241.4	−6.3	−235.2	0.16	SOVJIW	−28.0	−4.1	−23.9	0.17
EGIROB	−245.3	−4.2	−241.1	0.25	SOVJOC	−29.4	−5.0	−24.4	0.15
EMOZOV	−33.3	−7.1	−26.2	3.30	TONFOR	−21.2	−4.7	−16.5	2.46
EMOZUB	−14.5	−12.6	−1.9	0.70	UBESOJ	−16.4	−13.0	−3.4	0.06
EZAVOQ	−29.4	−28.1	−1.3	1.05	UBETAW	−28.9	−21.5	−7.5	0.39
EZUMER	−16.1	−15.3	−0.8	0.23	UBETEA	−22.1	−19.0	−3.1	0.70
FADCAP	−44.8	1.2	−46.0	0.07	UBEVAY	−30.7	−15.9	−14.8	0.35
FAHDOH	−36.3	−18.2	−18.2	0.27	UFIWAH	−7.0	−6.3	−0.7	1.03
FANJAG	−20.6	−10.5	−10.2	0.11	UJEFIY	−56.3	−34.3	−22.0	0.15
FIKVIE	−41.9	−21.1	−20.8	0.87	VAFRUP	−26.5	−22.8	−3.6	0.00
FIRXOT	−45.3	−26.2	−19.1	0.02	VAKJEX	−24.7	−16.4	−8.3	1.06
FODTIB	−29.1	−12.4	−16.7	0.38	VAVLUZ	−19.8	−11.4	−8.4	0.59
FUCVAA	−84.7	1.7	−85.8	0.58	VOHVIX	−25.6	−9.9	−15.7	0.41
GAMBIF	−136.3	−6.1	−130.2	0.70	VOTNEX	−47.3	−26.8	−20.5	0.52
GIGKEM	−66.8	−30.8	−36.0	0.30	VOTNOH	−63.7	−38.6	−25.2	0.05
GIKKEQ	−70.4	−2.2	−68.2	0.54	XAGLOG	−16.9	−18.2	1.3	4.74
GIXNOQ	−11.1	−9.1	−2.0	0.75	XAGMAT	−19.0	−21.1	2.2	3.52
GIYKOO	−12.6	−10.9	−1.7	0.39	XAQJAA	−143.7	−5.0	−138.8	1.65
GOBYOL	−16.0	−6.5	−9.5	0.44	XAQJEE	−145.3	−15.3	−130.0	1.89
GOKQUS	−14.0	−12.7	−1.2	0.44	XIVVAZ	−77.2	−10.1	−67.1	2.97
GUGGUK	−179.8	−5.4	−174.4	0.28	XOFSUG	−17.5	−15.7	−1.9	0.17
GUQHUV	−24.0	−20.1	−4.0	0.02	XUGPUK	−9.9	−9.3	−0.6	1.62
GUQJEH	−30.9	−25.4	−5.5	0.02	XUTBET	−18.3	−13.7	−4.6	0.92
GUQJIL	−29.4	−23.7	−5.7	0.28	YACVEE	−25.1	−14.3	−10.9	0.14
HASWUT	−95.8	−3.3	−92.5	2.65	YACVII	−12.8	−13.1	0.3	0.43
HIWNIK	−19.9	−16.4	−3.5	0.95	YAWJIP	−22.3	−7.4	−14.8	0.68
HUNWUI^5^	−15.1	−5.9	−9.2	1.09	YOCLUX	−47.0	−26.2	−20.9	0.39
HUNXAP^5^	−14.9	−5.8	−9.1	1.06	YONVAY	−13.9	−7.1	−6.8	0.50
HUNXIX	−32.0	−6.9	−25.1	0.16	ZESFEI	−26.0	2.3	−28.3	0.44
HUNXOD	−24.5	−6.8	−17.7	0.35					

1 Cambridge Structural Database code;

2 Intermolecular interaction energy calculated by the EUDOC program;

3 The van der Waals component of the intermolecular interaction energy;

4 The electrostatic component of the intermolecular interaction energy;

5 The translational increment was set to 0.5 Å.

**Table 2:** The Number and percentage of the EUDOC-generated complexes distributed in four categories of the mass-weighted root mean square deviations (mwRMSDs).

mwRMSD (Å)	Number of complexes (percentage)*		
	Method 1	Method 2	Method 3
≤0.5	84 (52%)	148 (92%)	150 (93%)
≤1.0	130 (81%)	159 (99%)	161 (100%)
≤2.0	149 (93%)	161 (100%)	161 (100%)
>2.0	12 (7%)	0	0
Total	161 (100%)	161 (100%)	161 (100%)

* Method 1: docking without consideration of crystal packing and structural waters; Method 2: docking with consideration of crystal packing; Method 3: docking with consideration of crystal packing and structural waters

### Docking with consideration of the influence of crystal packing

**Figure 2:**
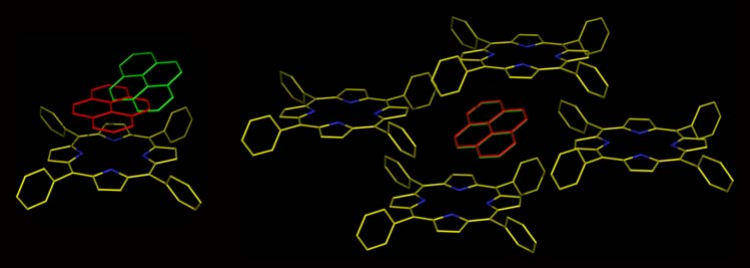
Overlays of the EUDOC-generated and crystal structures of complex XAGMAT. Left: Docking without consideration of the influence of crystal packing. Right: Docking with consideration of the influence of crystal packing. The C atoms of the EUDOC-generated and crystal structure are red and green, respectively. The N atom is blue.

Figure 2 shows the difference (mwRMSD of 3.52 Å) between the EUDOC-generated and crystal structures (CSD code: XAGMAT). Complex XAGMAT is one of the 12 complexes that the EUDOC program failed to reproduce. Despite a favorable π-π interaction between the guest and host structures predicted by the EUDOC program, in the corresponding crystal structure the guest structure surprisingly docks at a region at which it partly interacts with the host via a π-π interaction (see Fig. 2). This discrepancy suggests that the guest might partly interact with host(s) and/or guest(s) in neighboring unit cells of the crystal structure. To confirm this, the docking study with complex XAGMAT was repeated with consideration of the influence of crystal packing—namely, the guest was docked into a multimeric host system that included neighboring host(s) and/or guest(s). These neighboring structures were generated by applying the symmetry of the space group of the crystal structure. The host(s) and/or guest(s) in neighboring unit cells were excluded if these structures were >4.0 Å away from the guest to be docked. Interestingly, when the influence of crystal packing was taken into account, the EUDOC program accurately reproduced complex XAGMAT with an mwRMSD of 0.07 Å, instead of the 3.52 Å obtained without consideration of crystal packing. This result prompted a new docking study that considered the influence of crystal packing.

The results of the docking studies with consideration of the influence of crystal packing are listed in Table 3. The 12 complexes (CSD codes: AJUXUY, ATUKEF, BAXZAB, BIFKIK, CRAMCC10, FANJAG, GUGGUK, KOLMAZ, LAYMAZ, NEBQOA, QAJKAN, and RALQAW01) that were not reproduced previously by the EUDOC program were accurately reproduced after the influence of crystal packing was taken into account. With consideration of the influence of crystal packing, the EUDOC program reproduced all 161 complexes and accurately reproduced 99% of them (see Table 2).

**Table 3:** The intermolecular interaction energies of the 161 small-molecule complexes generated by the EUDOC program and their mass-weighted root mean square deviations (mwRMSDs) relative to their corresponding crystal structures.

CSD code^1^	E_total_ 2 (kcal/mol)	E_vdw_ 3 (kcal/mol)	E_ele_ 4 (kcal/mol)	mwRMSD (Å)	CSD code^1^	E_total_ 2 (kcal/mol)	E_vdw_ 3 (kcal/mol)	E_ele_ 4 (kcal/mol)	mwRMSD (Å)
ABELAU	−28.8	−11.6	−17.3	0.20	HUNXUJ	−29.2	−19.3	−9.9	0.28
ABULOZ	−39.2	0.8	−40.0	0.01	HUNYAQ	−41.2	−15.8	−25.4	0.43
ACPHDR^5^	−20.2	−17.9	−2.3	0.30	IKARUH	−19.1	−13.9	−5.2	0.44
AHOYEB	−31.1	−23.4	−7.7	0.89	IKUTOX	−22.1	−21.7	−0.5	0.46
AJUROM	−23.5	−18.3	−5.2	0.17	INUJAC	−28.9	−7.4	−21.5	0.22
AJUXOS	−30.0	−16.1	−13.9	0.32	ITAMIZ	−24.0	−19.2	−4.8	0.30
AJUXUY	−42.3	−19.4	−22.9	0.26	IXEKAX	−36.6	−29.0	−7.6	0.08
AJUYAF	−42.8	−20.0	−22.8	0.22	IXEKEB	−28.3	−22.6	−5.6	0.06
ASOKIC	−21.2	−13.2	−8.0	0.23	JAXPON	−21.5	−19.2	−2.3	0.13
ATUKEF	−30.1	−27.6	−2.5	0.15	JEBTAL	−18.6	−17.5	−1.1	0.10
AWUGEE	−25.3	−12.5	−12.8	0.27	JEJWOK	−29.3	−23.4	−5.9	0.32
AXEZIM	−39.3	−20.5	−18.8	0.17	JESCAL	−49.3	−40.0	−9.3	0.05
AYIBEP	−384.9	−3.7	−381.2	0.10	JIVMEG	−40.2	−10.5	−29.7	0.28
BAFZEN	−183.8	6.2	−190.0	0.10	JIVMUW	−36.5	−18.3	−18.2	0.26
BAHDEU^6^	−23.4	−21.6	−1.8	0.46	JULJAB	−23.3	−21.7	−1.6	0.03
BAKHIE	−53.0	−38.9	−14.0	0.18	JUMYOF	−26.2	−22.4	−3.9	0.34
BAMQAH	−41.5	−14.1	−27.3	0.18	KAXPOO	−36.2	−24.0	−12.2	0.23
BAPRAM	−53.1	−29.4	−23.8	0.11	KOHJAS	−13.6	−10.7	−2.9	0.34
BAPREQ	−36.8	−25.1	−11.7	0.13	KOLMAZ	−65.4	−28.3	−37.3	0.25
BAXZAB	−31.5	−23.1	−8.3	0.22	LAYMAZ	−49.5	−20.7	−28.8	0.30
BAYXII	−46.2	−8.5	−37.7	0.10	LODNOH01	−9.9	−9.9	0.0	0.44
BECVEK	−43.0	−28.8	−14.2	0.04	MAFRAN	−23.0	−15.4	−7.6	0.36
BEGVOZ	−67.4	−20.8	−46.6	0.24	MEXPEK	−14.7	−13.1	−1.6	0.53
BEVHER	−47.9	−36.1	−11.7	0.09	MNPOCB01	−41.9	−14.0	−27.9	0.08
BEVWAA	−43.6	−32.5	−11.1	0.19	MODTII	−173.7	−6.4	−167.3	0.26
BIFKIK	−48.1	−9.2	−38.9	0.32	MOZNIY	−23.6	−20.7	−2.9	0.22
BOHWUQ	−21.4	−19.5	−2.0	0.78	MUTFEM	−27.3	−24.4	−2.9	0.27
CACQED	−40.6	−30.3	−10.3	0.49	NEBQOA	−67.3	−39.6	−27.7	0.21
CAWRAT10	−61.1	−12.5	−48.6	0.30	NEPQUU	−16.0	−15.4	−0.6	0.52
CECMEC10	−44.9	−24.7	−20.2	0.12	NETKOM	−19.8	−12.8	−7.1	0.15
CENHAE	−16.5	−5.6	−11.0	0.36	NETKOM01	−20.0	−14.3	−5.7	0.14
CICVUF	−50.3	−29.2	−21.1	0.18	NEXLUX	−37.3	−18.6	−18.8	0.15
CIXCOB	−46.5	−45.9	−0.6	0.06	NOHHOH	−82.8	−52.9	−29.9	0.23
COBTIW	−42.0	−12.7	−29.2	0.03	NOYNAQ	−26.2	−13.7	−12.5	0.64
COXLEG10	−43.1	−16.8	−26.3	0.04	NUDHOJ	−75.8	−64.2	−11.6	0.02
COXQEL	−28.1	−25.2	−2.9	0.37	OBOHAO	−18.0	−17.8	−0.1	0.12
COYBOH	−18.3	−10.4	−7.9	0.23	OCAMIO	−25.4	−21.1	−4.3	0.15
CRAMCA10	−38.5	−1.4	−37.1	0.36	QAJKAN	−31.6	−25.8	−5.8	0.10
CRAMCB10	−43.1	−2.2	−40.9	0.23	QAKNAR	−44.6	−13.8	−30.8	0.18
CRAMCC10	−23.2	−0.3	−22.9	0.31	QAKNIZ	−46.9	−12.3	34.6	0.29
CUDXUU	−19.0	−17.1	−1.8	0.12	QAKNOF	−20.1	−8.3	−11.8	0.46
CYCBOB	−18.6	−12.4	−6.2	0.38	QATDIY	−34.9	−26.2	−8.7	0.24
CYCBOF11^5^	−14.6	−10.5	−4.1	0.44	RABJIJ	−22.1	−19.9	−2.2	0.20
DENFOR	−20.2	−18.8	−1.3	0.04	RACKAH	−30.4	−12.9	−17.5	0.29
DERFUB^5^	−10.8	−9.8	−1.0	0.55	RAHWED	−7.0	−6.8	−0.3	0.46
DESHEO	−51.6	−16.9	−34.7	0.12	RALQAW01	−23.0	−13.9	−9.1	0.57
DIZTIP	−22.5	−15.2	−7.3	0.44	RIBBUZ	−19.3	−6.9	−12.4	0.18
DOXWAO	−56.2	−26.9	−29.3	0.10	RUYWIR	−50.9	−40.2	−10.6	0.06
DUGGUH10	−35.0	−5.8	−29.2	0.20	SAKTII	−57.2	−32.2	−25.0	0.31
DUKHUM	−49.8	−9.6	−40.2	0.32	SEPKON	−56.3	−29.5	−26.8	0.24
EBASEF	−34.7	−26.6	−8.1	0.26	SEPNEG	−65.6	−30.1	−35.5	0.09
EGIRIV	−241.4	−6.3	−235.2	0.16	SOVJIW	−41.3	−12.8	−28.6	0.13
EGIROB	−245.3	−4.2	−241.1	0.25	SOVJOC	−38.2	−10.0	−28.2	0.09
EMOZOV	−63.5	−9.7	−53.8	0.22	TONFOR	−27.7	−15.1	−12.6	0.39
EMOZUB	−28.7	−12.8	−15.9	0.22	UBESOJ	−44.8	−36.7	−8.1	0.06
EZAVOQ	−37.6	−34.4	−3.2	0.41	UBETAW	−46.4	−35.5	−10.9	0.39
EZUMER	−16.1	−15.3	−0.8	0.23	UBETEA	−39.2	−33.3	−5.9	0.12
FADCAP	−45.9	−0.5	−45.4	0.07	UBEVAY	−52.8	−32.9	−19.9	0.05
FAHDOH	−39.4	−26.4	−13.0	0.26	UFIWAH	−19.0	−18.3	−0.6	0.06
FANJAG	−29.0	−18.0	−11.1	0.16	UJEFIY	−52.4	−67.4	15.0	0.15
FIKVIE	−54.7	−29.8	−24.9	0.43	VAFRUP^5^	−26.5	−22.8	−3.6	0.00
FIRXOT	−48.0	−30.7	−17.3	0.02	VAKJEX	−44.7	−35.9	−8.7	0.25
FODTIB	−53.6	−36.0	−17.5	0.14	VAVLUZ	−36.1	−23.3	−12.8	0.16
FUCVAA	−100.9	0.1	−101.0	0.66	VOHVIX	−32.4	−18.1	−14.2	0.30
GAMBIF	−136.3	−6.1	−130.2	0.70	VOTNEX	−50.9	−30.3	−20.6	0.38
GIGKEM	−77.4	−34.6	−42.9	0.22	VOTNOH	−84.2	−47.2	−37.0	0.05
GIKKEQ	−135.2	−4.5	−130.7	0.00	XAGLOG	−32.0	−31.4	−0.6	0.33
GIXNOQ	−27.1	−16.4	−10.7	0.27	XAGMAT	−36.2	−36.9	0.6	0.07
GIYKOO	−30.7	−22.7	−7.9	0.33	XAQJAA	−143.7	−5.0	−138.8	1.65
GOBYOL	−38.5	−27.2	−11.3	0.19	XAQJEE	−145.3	−15.3	−130.0	1.89
GOKQUS^5^	−16.7	−15.8	−0.9	0.28	XIVVAZ	−79.7	−20.0	−59.7	0.45
GUGGUK	−223.9	−3.5	−220.5	0.66	XOFSUG	−37.6	−34.4	−3.2	0.05
GUQHUV^5^	−24.0	−20.1	−4.0	0.02	XUGPUK	−18.5	−17.8	−0.7	0.31
GUQJEH	−30.9	−25.4	−5.5	0.02	XUTBET	−28.7	−23.3	−5.4	0.84
GUQJIL	−50.7	−41.0	−9.7	0.05	YACVEE	−30.8	−19.7	−11.1	0.14
HASWUT	−215.3	−16.1	−199.2	0.22	YACVII	−18.0	−18.1	0.1	0.27
HIWNIK	−28.5	−25.2	−3.3	0.36	YAWJIP	−33.0	−17.8	−15.1	0.16
HUNWUI	−35.6	−21.5	−14.1	0.08	YOCLUX	−47.0	−26.2	−20.9	0.39
HUNXAP	−33.7	−18.5	−15.2	0.13	YONVAY	−28.6	−20.6	−8.0	0.46
HUNXIX	−42.1	−14.4	−27.7	0.13	ZESFEI	−38.1	−9.2	−28.9	0.33
HUNXOD	−39.7	−16.7	−23.0	0.28					

1 Cambridge Structural Database code;

2 Intermolecular interaction energy calculated by the EUDOC program;

3 The van der Waals component of the intermolecular interaction energy;

4 The electrostatic component of the intermolecular interaction energy.

5 EUDOC identified one alternative binding mode that is energetically indistinguishable from the binding mode of the crystal structure.

6 EUDOC identified three alternative binding modes that are energetically indistinguishable from the binding mode of the crystal structure.

### Docking with consideration of the influences of crystal packing and structural waters

Two crystal structures (CSD codes: XAQJAA and XAQJEE) could not be accurately reproduced by the EUDOC program even after consideration of the influence of crystal packing (XAQJAA: mwRMSD = 1.65 Å; XAQJEE: mwRMSD = 1.89 Å). Visual inspection of these structures revealed that the binding between the guest and host structures was mediated by crystallographically determined water molecules. This mediation suggested that, similar to the crystal packing, water molecules might also play an important role in guest-host complexation, and it might be necessary to include them in the multimeric host system for docking. Accordingly, the docking studies with the 161 complexes were repeated with consideration of the influences of both crystal packing and structural waters. The results are listed in Table 4. Indeed, the EUDOC program accurately reproduced complexes XAQJAA and XAQJEE with mwRMSDs of 0.03 and 0.27 Å, respectively. Taking into account the influences of both crystal packing and structural waters, the EUDOC program accurately reproduced all 161 complexes (see Table 2).

**Table 4:** The intermolecular interaction energies of the 161 small-molecule complexes generated by the EUDOC program and their mass-weighted root mean square deviations (mwRMSDs) relative to their corresponding crystal structures.

CSD code^1^	E_total_ 2 (kcal/mol)	E_vdw_ 3 (kcal/mol)	E_ele_ 4 (kcal/mol)	mwRMSD (Å)	CSD code^1^	E_total_ 2 (kcal/mol)	E_vdw_ 3 (kcal/mol)	E_ele_ 4 (kcal/mol)	mwRMSD (Å)
ABELAU	−28.8	−11.6	−17.3	0.20	HUNXUJ	−29.2	−19.3	−9.9	0.28
ABULOZ^5^	−46.1	2.3	−48.3	0.03	HUNYAQ	−41.2	−15.8	−25.4	0.43
ACPHDR^6^	−20.2	−17.9	−2.3	0.30	IKARUH^5^	−30.4	−16.8	−13.6	0.63
AHOYEB	−31.1	−23.4	−7.7	0.89	IKUTOX	−22.1	−21.7	−0.5	0.46
AJUROM	−23.5	−18.3	−5.2	0.17	INUJAC	−28.9	−7.4	−21.5	0.22
AJUXOS	−30.0	−16.1	−13.9	0.32	ITAMIZ^5^	−24.4	−20.1	−4.3	0.40
AJUXUY	−42.3	−19.4	−22.9	0.26	IXEKAX	−36.6	−29.0	−7.6	0.08
AJUYAF	−42.8	−20.0	−22.8	0.22	IXEKEB	−28.3	−22.6	−5.6	0.06
ASOKIC	−21.2	−13.2	−8.0	0.23	JAXPON	−21.5	−19.2	−2.3	0.13
ATUKEF^5^	−29.7	−27.8	−1.8	0.12	JEBTAL	−18.6	−17.5	−1.1	0.10
AWUGEE	−25.3	−12.5	−12.8	0.27	JEJWOK	−29.3	−23.4	−5.9	0.32
AXEZIM	−39.3	−20.5	−18.8	0.17	JESCAL	−49.3	−40.0	−9.3	0.05
AYIBEP^5^	−423.2	−1.2	−422.0	0.07	JIVMEG	−40.2	−10.5	−29.7	0.28
BAFZEN^5^	−205.0	4.9	−210.0	0.04	JIVMUW	−36.5	−18.3	−18.2	0.26
BAHDEU^7^	−23.4	−21.6	−1.8	0.46	JULJAB	−23.3	−21.7	−1.6	0.03
BAKHIE	−53.0	−38.9	−14.0	0.18	JUMYOF	−26.2	−22.4	−3.9	0.34
BAMQAH	−41.5	−14.1	−27.3	0.18	KAXPOO	−36.2	−24.0	−12.2	0.23
BAPRAM	−53.1	−29.4	−23.8	0.11	KOHJAS	−13.6	−10.7	−2.9	0.34
BAPREQ	−36.8	−25.1	−11.7	0.13	KOLMAZ	−65.4	−28.3	−37.3	0.25
BAXZAB	−31.5	−23.1	−8.3	0.22	LAYMAZ	−49.5	−20.7	−28.8	0.30
BAYXII	−46.2	−8.5	−37.7	0.10	LODNOH01	−9.9	−9.9	0.0	0.44
BECVEK	−43.0	−28.8	−14.2	0.04	MAFRAN	−23.0	−15.4	−7.6	0.36
BEGVOZ	−67.4	−20.8	−46.6	0.24	MEXPEK	−14.7	−13.1	−1.6	0.53
BEVHER	−47.9	−36.1	−11.7	0.09	MNPOCB01	−41.9	−14.0	−27.9	0.08
BEVWAA	−43.6	−32.5	−11.1	0.19	MODTII	−173.7	−6.4	−167.3	0.26
BIFKIK	−48.1	−9.2	−38.9	0.32	MOZNIY	−23.6	−20.7	−2.9	0.22
BOHWUQ	−21.4	−19.5	−2.0	0.78	MUTFEM	−27.3	−24.4	−2.9	0.27
CACQED^5^	−34.7	−24.2	−10.5	0.32	NEBQOA	−67.3	−39.6	−27.7	0.21
CAWRAT10	−61.1	−12.5	−48.6	0.30	NEPQUU	−16.0	−15.4	−0.6	0.52
CECMEC10^5^	−48.9	−25.0	−23.8	0.12	NETKOM	−19.8	−12.8	−7.1	0.15
CENHAE	−16.5	−5.6	−11.0	0.36	NETKOM01	−20.0	−14.3	−5.7	0.14
CICVUF	−50.3	−29.2	−21.1	0.18	NEXLUX	−37.3	−18.6	−18.8	0.15
CIXCOB	−46.5	−45.9	−0.6	0.06	NOHHOH	−82.8	−52.9	−29.9	0.23
COBTIW	−42.0	−12.7	−29.2	0.03	NOYNAQ	−26.2	−13.7	−12.5	0.64
COXLEG10	−43.1	−16.8	−26.3	0.04	NUDHOJ	−75.8	−64.2	−11.6	0.02
COXQEL	−28.1	−25.2	−2.9	0.37	OBOHAO	−18.0	−17.8	−0.1	0.12
COYBOH	−18.3	−10.4	−7.9	0.23	OCAMIO	−25.4	−21.1	−4.3	0.15
CRAMCA10	−38.5	−1.4	−37.1	0.36	QAJKAN	−31.6	−25.8	−5.8	0.10
CRAMCB10^5^	−53.7	2.1	−55.9	0.23	QAKNAR	−44.6	−13.8	−30.8	0.18
CRAMCC10	−23.2	−0.3	−22.9	0.31	QAKNIZ	−46.9	−12.3	34.6	0.29
CUDXUU	−19.0	−17.1	−1.8	0.12	QAKNOF	−20.1	−8.3	−11.8	0.46
CYCBOB	−18.6	−12.4	−6.2	0.38	QATDIY	−34.9	−26.2	−8.7	0.24
CYCBOF11^6^	−14.6	−10.5	−4.1	0.44	RABJIJ	−22.1	−19.9	−2.2	0.20
DENFOR	−20.2	−18.8	−1.3	0.04	RACKAH	−30.4	−12.9	−17.5	0.29
DERFUB^6^	−10.8	−9.8	−1.0	0.55	RAHWED	−7.0	−6.8	−0.3	0.46
DESHEO	−51.6	−16.9	−34.7	0.12	RALQAW01	−23.0	−13.9	−9.1	0.57
DIZTIP	−22.5	−15.2	−7.3	0.44	RIBBUZ	−19.3	−6.9	−12.4	0.18
DOXWAO	−56.2	−26.9	−29.3	0.10	RUYWIR	−50.9	−40.2	−10.6	0.06
DUGGUH10	−35.0	−5.8	−29.2	0.20	SAKTII	−57.2	−32.2	−25.0	0.31
DUKHUM	−49.8	−9.6	−40.2	0.32	SEPKON	−56.3	−29.5	−26.8	0.24
EBASEF	−34.7	−26.6	−8.1	0.26	SEPNEG	−65.6	−30.1	−35.5	0.09
EGIRIV^5^	−289.8	−3.8	−285.9	0.22	SOVJIW	−41.3	−12.8	−28.6	0.13
EGIROB^5^	−271.6	9.1	−280.7	0.43	SOVJOC	−38.2	−10.0	−28.2	0.09
EMOZOV	−63.5	−9.7	−53.8	0.22	TONFOR	−27.7	−15.1	−12.6	0.39
EMOZUB	−28.7	−12.8	−15.9	0.22	UBESOJ	−44.8	−36.7	−8.1	0.06
EZAVOQ^5^	−40.6	−33.3	−7.4	0.35	UBETAW^5^	−45.5	−34.5	−11.0	0.39
EZUMER	−16.1	−15.3	−0.8	0.23	UBETEA	−39.2	−33.3	−5.9	0.12
FADCAP	−45.9	−0.5	−45.4	0.07	UBEVAY	−52.8	−32.9	−19.9	0.05
FAHDOH	−39.4	−26.4	−13.0	0.26	UFIWAH	−19.0	−18.3	−0.6	0.06
FANJAG	−29.0	−18.0	−11.1	0.16	UJEFIY	−52.4	−67.4	15.0	0.15
FIKVIE^5^	−59.0	−26.6	−32.5	0.15	VAFRUP^6^	−26.5	−22.8	−3.6	0.00
FIRXOT	−48.0	−30.7	−17.3	0.02	VAKJEX	−44.7	−35.9	−8.7	0.25
FODTIB	−53.6	−36.0	−17.5	0.14	VAVLUZ	−36.1	−23.3	−12.8	0.16
FUCVAA	−100.9	0.1	−101.0	0.66	VOHVIX	−32.4	−18.1	−14.2	0.30
GAMBIF	−136.3	−6.1	−130.2	0.70	VOTNEX	−50.9	−30.3	−20.6	0.38
GIGKEM	−77.4	−34.6	−42.9	0.22	VOTNOH	−84.2	−47.2	−37.0	0.05
GIKKEQ	−135.2	−4.5	−130.7	0.00	XAGLOG	−32.0	−31.4	−0.6	0.33
GIXNOQ	−27.1	−16.4	−10.7	0.27	XAGMAT	−36.2	−36.9	0.6	0.07
GIYKOO	−30.7	−22.7	−7.9	0.33	XAQJAA^5^	−193.7	−14.3	−179.4	0.03
GOBYOL	−38.5	−27.2	−11.3	0.19	XAQJEE^5^	−196.7	−15.9	−180.8	0.27
GOKQUS^6^	−16.7	−15.8	−0.9	0.28	XIVVAZ^5^	−118.7	−11.0	−107.7	0.43
GUGGUK^5^	−240.6	−6.7	−233.9	0.26	XOFSUG	−37.6	−34.4	−3.2	0.05
GUQHUV^6^	−24.0	−20.1	−4.0	0.02	XUGPUK	−18.5	−17.8	−0.7	0.31
GUQJEH	−30.9	−25.4	−5.5	0.02	XUTBET	−28.7	−23.3	−5.4	0.84
GUQJIL	−50.7	−41.0	−9.7	0.05	YACVEE	−30.8	−19.7	−11.1	0.14
HASWUT^5^	−231.0	−11.4	−219.6	0.22	YACVII	−18.0	−18.1	0.1	0.27
HIWNIK	−28.5	−25.2	−3.3	0.36	YAWJIP	−33.0	−17.8	−15.1	0.16
HUNWUI	−35.6	−21.5	−14.1	0.08	YOCLUX^5^	−52.9	−22.2	−30.8	0.32
HUNXAP	−33.7	−18.5	−15.2	0.13	YONVAY	−28.6	−20.6	−8.0	0.46
HUNXIX	−42.1	−14.4	−27.7	0.13	ZESFEI	−38.1	−9.2	−28.9	0.33
HUNXOD	−39.7	−16.7	−23.0	0.28					

1 Cambridge Structural Database code;

2 Intermolecular interaction energy calculated by the EUDOC program;

3 The van der Waals component of the intermolecular interaction energy;

4 The electrostatic component of the intermolecular interaction energy;

5 Structural water molecules were present in the multimeric host system.

6 EUDOC identified one alternative binding mode that is energetically indistinguishable from the binding mode of the crystal structure.

7 EUDOC identified three alternative binding modes that are energetically indistinguishable from the binding mode of the crystal structure.

## Discussion

### The influences of crystal packing and structural water on docking

This study shows that 31 (19%) of the 161 complexes could not be accurately reproduced with mwRMSDs of <1.0 Å by the EUDOC program if neighboring host(s) and/or guest(s) in the crystal structure were not included as part of the multimeric host system, whereas only 2 (1%) of these complexes could not be accurately reproduced with mwRMSDs of <1.0 Å if neighboring structures were included but water molecules were excluded from the host system. These results show that the influence of crystal packing or crystal environment on crystal structures of guest-host complexes is significant, which is consistent with the reported influence of crystal packing on protein structures [14]. These results also show that the influence of structural waters on guest–host complex crystal structures is insignificant, which is consistent with our reported finding that complexation between a small molecule and a protein is not commonly mediated by water molecules [3]. This study therefore suggests that crystal packing should be taken into account when reproducing crystal structures of small-molecule guest-host complexes through docking studies, whereas water molecules, counterions or other companying molecules such as ethanol can be excluded from the host system. This study also suggests that, *ideally*, to perform prospective and accurate docking of a small molecule into another small molecule, repetitive docking of a guest or host into a guest-host complex that is generated by the previous docking is preferred, because a host is sometimes too small to prevent a guest from interacting with nearby guest(s) and/or host(s). It is worth noting, however, that the success rate of docking a small molecule into another small molecule is about 93% if the influence of crystal packing is ignored.

### Demonstration of the accuracy of the nonbonded parameters of the AMBER force field

In the crystal structure of Rebek's acridine diacid in complex with quinoxaline (CSD code: YAWJIP) there are two crystallographically independent forms of the complex in the asymmetric unit [15]. This structure was used to benchmark the all-atom AMBER/OPLS force field [16]. In this study, complex YAWJIP was accurately reproduced by the EUDOC program using the nonbonded force field parameters of the second-generation AMBER force field [3], [17]; the mwRMSDs of the guest position between the EUDOC-generated and crystal complexes for forms A and B are both 0.16 Å. The accurate reproduction of the remaining crystal structures (see Table 2) further demonstrates the accuracy of the nonbonded force field parameters of the second-generation AMBER force field (parm99.dat) for reproducing crystal structures [3], [17].

### Application to supramolecular chemistry

The above results suggest that the EUDOC program can predict small-molecule guest-host complexes with a reasonable success rate (93%), without consideration to the mechanism of small-molecule complex aggregation—namely, without being given the multimeric host system. To demonstrate this ability herein, the crystal structure of complex YAWJIP is used as a model system. Based on the NMR spectroscopic data of complex YAWJIP, the guest structure quinoxaline was proposed to have face-to-face π-stacking with the acridine portion of Rebek's acridine diacids in an early report of the complex [18]. However, the face-to-face π-stacking was found in neither the crystal structure of complex YAWJIP [15] nor the Monte Carlo statistical mechanics calculations of the complex [16].

To perform a prospective docking study, the two-dimensional structures of quinoxaline and Rebek's acridine diacid were converted to 3D structures using the PCModel program (Serena Software LLC, Bloomington, IN), respectively. Both 3D structures were refined with energy minimization monitored with a normal-mode (NMODE) analysis to ensure that the energy minimization stopped when the minimized conformation reached a local potential energy minimum. The energy minimization was performed by using the SANDER module of the AMBER5 program [19] with the second-generation AMBER force field [17], and the NMODE analysis was carried out using the NMODE module of the AMBER8 program [19]. Given the refined 3D structures of quinoxaline and Rebek's acridine diacid, the EUDOC program generated a complex nearly identical to the crystal structure of form A (mwRMSD: 0.7 Å) but not the proposed complex with nearly face-to-face π-stacking. This perspective docking result suggests that the EUDOC program is a useful tool for predicting 3D models of guest-host complexes to aid the design of new molecular entities according to the principles of supramolecular chemistry.

Visual inspection of 154 reported crystal structures of proteins in complex with small molecules [3] and the 161 crystal structures of guest-host complexes reported herein suggested that the noncovalent interactions of the guest-host complexes are in general weaker than those of the protein complexes. The average of the interaction energies of the guest-host complexes listed in Table 4 (−50.1 kcal/mol) and the average of those of the protein complexes in Table VI (excluding 1PHA) of reference 3 (−108.5 kcal/mol) quantitatively confirm the relatively weak noncovalent interactions of the guest-host complex. This confirmation suggests that to design high-affinity guest-host complexes it is of advantage to incorporate the entropic energy into the binding, because the number of functional groups that can be introduced onto the guest and host structures to confer the nonbonded interactions is limited by the size of the two partners. This “saturation” problem is more apparent for small-molecule complexes than for protein complexes. It is therefore conceivable that the EUDOC program is also a useful tool for estimating the interaction energies of guest-host complexes to aid the design of new molecular entities according to the principles of supramolecular chemistry.

## Methods

### Preparation of the host and guest structures

The guest and host structures were taken from the crystal structures of their corresponding complexes obtained from CSD [1]. Water molecules, counterions, and solvent molecules such as ethanol were removed from the guest or host structure. Hydrogen atoms were added by using the QUANTA97 program (Accelrys Software, Inc, San Diego, California) followed by energy minimization of the hydrogen atoms using the SANDER module of the AMBER5 program [19] with the second-generation AMBER force field (parm99.dat) [17] and a positional constraint on all non-hydrogen atoms. The protonation states of the guest and host structures shown in Figures S1, S2, S3, S4, S5, S6, S7, S8, S9, S10, S11, S12, S13, S14, S15, S16 of Supporting Information were determined according to p*K*a values of functional groups at pH of 7.0. The atomic charges of the guest and host structures listed in Table S1 of Supporting Information were generated according to the RESP procedure [20] with ab initio calculations at the HF/6-31G* level using the Gaussian03 program [21]. The AMBER atom types of the guest and host structures listed in Table S1 of Supporting Information were assigned by the ANTECHAMBER module of AMBER7 [19].

### Docking studies using the EUDOC program

The algorithm of the EUDOC program has been reported elsewhere [3]. Briefly, it uses a systematic search protocol, translating and rotating a guest in a putative binding pocket of a host to search for energetically favorable orientations and positions of the guest relative to the host. A docking box is defined within the binding pocket to confine the translation of the ligand. The intermolecular interaction energy is the potential energy of the guest-host complex relative to the potential energies of the two partners in their free state. This energy was calculated according to equations 1 and 2 using the second-generation AMBER force field [17].
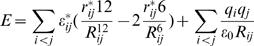
(1)

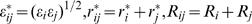
(2)


In calculating the intermolecular interaction energy, the dielectric constant was set to 1.0, and the distance cutoffs for steric and electrostatic interactions were set to 10^9^ Å. A docking box was defined to enclose the guest structure in the crystal structure of the guest-host complex. The size of the docking box and the cutoff for the interaction energy used by the EUDOC program are listed in Table S2 of Supporting Information. The complex-prediction module of the EUDOC program (Version 41, executable available from YPP) was used to translate and rotate the guest around the host at increments of 1.0 Å and 10° of arc, respectively, unless noted otherwise in Table 1.

To consider the influence of crystal packing, the PyMOL program (DeLano Scientific LLC, South San Francisco, California) was used to generate a multimeric host system by applying the symmetry of the space group of the crystal structure. The host or guest structure was excluded from the multimeric host system if the shortest distance between a heavy atom of the guest structure to be docked and the heavy atom of the host/guest structure in neighboring unit cells was >4.0 Å.

### Energy minimizations monitored with normal-mode analysis

Energy minimization was performed with the SANDER module of AMBER5 [19] using (1) maxcyc = 10^6^, (2) dielc = 80, (3) scnb = 2.0, (4) scee = 1.2, (5) ntr = 0, (6) ntmin = 1 or 2, (7) ncyc = 0, (8) cut = 12, and (9) drms = 10^−7^. NMODE analysis was performed with the NMODE module of AMBER8 [19] using (1) ilevel = 0 or 1, (2) cut = 12, (3) scnb = 2.0, (4) scee = 1.2, (5) dielc = 80, and (6) idiel = 1.

### Mass-weighted root mean square deviations

The mwRMSDs were calculated by superimposing the host portion of the EUDOC-generated complex over the corresponding host portion of the crystal structure followed by a calculation for the mwRMSD of all atoms of the guest portion in the two superimposed complexes using the PTRAJ module of AMBER8 [19].

## Supporting Information

Table S1 The RESP charges and the AMBER atom types of 161 host-guest complexes
(2.63 MB DOC)

Table S2 The docking box size and the interaction energy cutoff used by the EUDOC program for reproducing the 161 host-guest complexes
(0.24 MB DOC)

Figure S1 Chemical structures of the 161 host-guest complexes.
(0.10 MB TIF)

Figure S2 Chemical structures of the 161 host-guest complexes.
(0.09 MB TIF)

Figure S3 Chemical structures of the 161 host-guest complexes.
(0.11 MB TIF)

Figure S4 Chemical structures of the 161 host-guest complexes.
(0.10 MB TIF)

Figure S5 Chemical structures of the 161 host-guest complexes.
(0.09 MB TIF)

Figure S6 Chemical structures of the 161 host-guest complexes.
(0.11 MB TIF)

Figure S7 Chemical structures of the 161 host-guest complexes.
(0.12 MB TIF)

Figure S8 Chemical structures of the 161 host-guest complexes.
(0.12 MB TIF)

Figure S9 Chemical structures of the 161 host-guest complexes.
(0.12 MB TIF)

Figure S10 Chemical structures of the 161 host-guest complexes.
(0.12 MB TIF)

Figure S11 Chemical structures of the 161 host-guest complexes.
(0.20 MB TIF)

Figure S12 Chemical structures of the 161 host-guest complexes.
(0.12 MB TIF)

Figure S13 Chemical structures of the 161 host-guest complexes.
(0.10 MB TIF)

Figure S14 Chemical structures of the 161 host-guest complexes.
(0.21 MB TIF)

Figure S15 Chemical structures of the 161 host-guest complexes.
(0.11 MB TIF)

Figure S16 Chemical structures of the 161 host-guest complexes.
(0.12 MB TIF)
