# Supplementary material for: Accurate Reproduction of 161 Small-Molecule Complex Crystal Structures using the EUDOC Program: Expanding the Use of EUDOC to Supramolecular Chemistry
Source: PLoS One. 2007 Jun 13;2(6):e531. doi: 10.1371/journal.pone.0000531 (PMC1888730; doi:10.1371/journal.pone.0000531)
Supplement: Table S1 — The RESP charges and the AMBER atom types of 161 host-guest complexes (2.63 MB DOC) [file pone.0000531.s001.doc]

Table S1. The RESP charges and the AMBER atom types of 161 host-guest complexes. Atom names, the AMBER atom types, Cartesian coordinates x,y and z, and the RESP charges are at columns 3, 4, 6, 7, 8 and 9. Suffixes “h” and “g” specify the host and guest of the complex crystal structure, respectively.

ABELAUh

1 0 C3 CA M 2.6990 3.8590 0.9130 -0.2530

2 1 H3 HA E 2.5350 4.6790 1.3640 0.1100

3 1 C1 CA M 3.9670 3.3190 0.9010 0.2800

4 3 H1 H4 E 4.6600 3.7900 1.3490 0.0710

5 3 N1 NC M 4.2760 2.1710 0.2980 -0.5660

6 5 C2 CA M 3.2560 1.5300 -0.2850 0.2800

7 6 H2 H4 E 3.4340 0.6920 -0.6960 0.0710

8 6 C4 CA M 1.9770 2.0010 -0.3250 -0.2530

9 8 H4 HA E 1.3020 1.4980 -0.7640 0.1100

10 8 C5 CA M 1.6630 3.2050 0.2670 0.0650

11 10 C6 CT M 0.3010 3.8120 0.0880 0.0190

12 11 H5 HC E -0.3870 3.0940 -0.0310 0.0630

13 11 C7 CT M -0.1940 4.8640 1.0970 0.0190

14 13 C9 CA S -1.4650 4.6440 1.8650 0.0650

15 14 C12 CA B -1.7090 5.3970 3.0030 -0.2530

16 15 C17 CA B -2.9360 5.3500 3.6080 0.2800

17 16 N2 NC S -3.9500 4.5990 3.1930 -0.5660

18 17 C18 CA B -3.6870 3.8200 2.1370 0.2800

19 18 C13 CA S -2.4870 3.8050 1.4670 -0.2530

20 19 H10 HA E -2.3600 3.2170 0.7310 0.1100

21 18 H14 H4 E -4.3750 3.2400 1.8300 0.0710

22 16 H13 H4 E -3.0710 5.8910 4.3760 0.0710

23 15 H9 HA E -1.0240 5.9480 3.3640 0.1100

24 13 H6 HC E 0.5460 5.1140 1.7220 0.0630

25 13 C10 CT M -0.3010 5.9370 -0.0880 0.0190

26 25 C14 CA S -1.6630 6.5440 -0.2670 0.0650

27 26 C19 CA B -2.6990 5.8900 -0.9130 -0.2530

28 27 C23 CA B -3.9670 6.4300 -0.9010 0.2800

29 28 N4 NC S -4.2760 7.5780 -0.2980 -0.5660

30 29 C24 CA B -3.2560 8.2190 0.2850 0.2800

31 30 C20 CA S -1.9770 7.7480 0.3250 -0.2530

32 31 H16 HA E -1.3020 8.2510 0.7640 0.1100

33 30 H20 H4 E -3.4340 9.0570 0.6960 0.0710

34 28 H19 H4 E -4.6600 5.9590 -1.3490 0.0710

35 27 H15 HA E -2.5350 5.0700 -1.3640 0.1100

36 25 H8 HC E 0.3870 6.6550 0.0310 0.0630

37 25 C8 CT M 0.1940 4.8850 -1.0970 0.0190

38 37 H7 HC E -0.5460 4.6350 -1.7220 0.0630

39 37 C11 CA M 1.4650 5.1050 -1.8650 0.0650

40 39 C15 CA M 1.7090 4.3520 -3.0030 -0.2530

41 40 H11 HA E 1.0240 3.8010 -3.3640 0.1100

42 40 C21 CA M 2.9360 4.3990 -3.6080 0.2800

43 42 H17 H4 E 3.0710 3.8580 -4.3760 0.0710

44 42 N3 NC M 3.9500 5.1500 -3.1930 -0.5660

45 44 C22 CA M 3.6870 5.9290 -2.1370 0.2800

46 45 H18 H4 E 4.3750 6.5090 -1.8300 0.0710

47 45 C16 CA M 2.4870 5.9440 -1.4670 -0.2530

48 47 H12 HA E 2.3600 6.5320 -0.7310 0.1100

ABELAUg

1 0 O1 OH M 6.8320 1.4680 -0.3000 -0.5930

2 1 H1 HO E 6.0480 1.7350 -0.1680 0.4510

3 1 C1 CA M 7.3420 2.0820 -1.3980 0.2820

4 3 C2 CA M 6.6250 3.0100 -2.1460 -0.3370

5 4 H2 HA E 5.7330 3.2270 -1.9010 0.1410

6 4 C4 CA M 7.2030 3.6140 -3.2430 0.2820

7 6 O2 OH S 6.5480 4.5440 -4.0020 -0.5930

8 7 H5 HO E 5.7670 4.6430 -3.7100 0.4510

9 6 C6 CA M 8.5080 3.3140 -3.6010 -0.1770

10 9 H6 HA E 8.9110 3.7380 -4.3490 0.1620

11 9 C5 CA M 9.2160 2.3910 -2.8550 -0.2370

12 11 H4 HA E 10.1070 2.1770 -3.1010 0.1820

13 11 C3 CA M 8.6490 1.7720 -1.7540 -0.1770

14 13 H3 HA E 9.1470 1.1430 -1.2460 0.1620

ABULOZh

1 0 C1 CT M 1.3980 0.7120 6.9160 0.0830

2 1 H1 H1 E 1.9290 1.2290 7.5420 0.0370

3 1 H2 H1 E 0.4950 1.0610 6.9420 0.0370

4 1 C2 CT M 1.9350 0.9210 5.5710 0.0830

5 4 H3 H1 E 2.0070 1.8690 5.3810 0.0370

6 4 H4 H1 E 2.8160 0.5230 5.4960 0.0370

7 4 O2 OS M 1.0000 0.2770 4.6170 -0.3150

8 7 C3 CT M 1.4410 0.3430 3.3570 0.0830

9 8 H5 H1 E 2.2750 -0.1410 3.2810 0.0370

10 8 H6 H1 E 1.6060 1.2690 3.1200 0.0370

11 8 C4 CT M 0.4430 -0.2370 2.4170 0.0830

12 11 H7 H1 E 0.7390 -0.1120 1.5040 0.0370

13 11 H8 H1 E -0.4090 0.2150 2.5230 0.0370

14 11 O3 OS M 0.2870 -1.6590 2.6980 -0.3150

15 14 C5 CT M -0.6130 -2.2580 1.9490 0.0830

16 15 H9 H1 E -0.4140 -2.0860 1.0150 0.0370

17 15 H10 H1 E -1.4880 -1.8870 2.1410 0.0370

18 15 C6 CT M -0.6450 -3.7060 2.1750 0.0830

19 18 H11 H1 E 0.2310 -4.0840 1.9940 0.0370

20 18 H12 H1 E -1.2800 -4.1170 1.5690 0.0370

21 18 O4 OS M -1.0320 -3.9890 3.5770 -0.3150

22 21 C7 CT M -0.9540 -5.2730 3.9250 0.0830

23 22 H13 H1 E -0.0310 -5.5630 3.8690 0.0370

24 22 H14 H1 E -1.4720 -5.8040 3.2990 0.0370

25 22 C8 CT M -1.4530 -5.5090 5.2870 0.0830

26 25 H15 H1 E -2.3700 -5.2020 5.3510 0.0370

27 25 H16 H1 E -1.4400 -6.4600 5.4740 0.0370

28 25 O5 OS M -0.6420 -4.8120 6.2750 -0.3150

29 28 C9 CT M -1.1530 -4.8460 7.5400 0.0830

30 29 H17 H1 E -1.2910 -5.7670 7.8100 0.0370

31 29 H18 H1 E -2.0110 -4.3940 7.5560 0.0370

32 29 C10 CT M -0.2380 -4.1940 8.4750 0.0830

33 32 H19 H1 E 0.6300 -4.6220 8.4240 0.0370

34 32 H20 H1 E -0.5730 -4.3050 9.3790 0.0370

35 32 O6 OS M -0.0970 -2.7940 8.1900 -0.3150

36 35 C11 CT M 0.7510 -2.1550 8.9720 0.0830

37 36 H21 H1 E 1.6330 -2.5510 8.8770 0.0370

38 36 H22 H1 E 0.4780 -2.2640 9.8960 0.0370

39 36 C12 CT M 0.8290 -0.7470 8.6640 0.0830

40 39 H23 H1 E -0.0560 -0.3540 8.7140 0.0370

41 39 H24 H1 E 1.3930 -0.3010 9.3160 0.0370

42 39 O1 OS M 1.3740 -0.5550 7.3330 -0.3150

ABULOZg

1 0 N1 N3 M -0.7000 -1.8540 5.5210 -0.7080

2 1 H1 H E -0.0800 -1.1910 5.1860 0.4270

3 1 H2 H E -0.4220 -2.1790 6.3700 0.4270

4 1 H3 H E -1.5560 -1.4730 5.6650 0.4270

5 1 H4 H E -0.7530 -2.5830 4.8800 0.4270

ACPHDRh

1 0 N1 N3 M 6.0160 4.5820 8.0400 -0.1590

2 1 H1 H E 6.4200 5.2840 7.4390 0.3210

3 1 H2 H E 5.8420 5.0810 8.8970 0.3210

4 1 C1 CT M 6.9690 3.4460 8.3080 0.0220

5 4 H3 HP E 7.0380 2.9580 7.3820 0.0870

6 4 H4 HP E 6.5560 2.8450 9.0040 0.0870

7 4 C3 CT M 8.3150 3.9920 8.7290 -0.0060

8 7 H5 HC E 8.8060 4.4490 7.8980 0.0360

9 7 H6 HC E 8.2060 4.6970 9.5280 0.0360

10 7 C6 CT M 9.2390 2.8970 9.2340 -0.0060

11 10 H9 HC E 9.2510 2.1680 8.6380 0.0360

12 10 H10 HC E 8.9120 2.5970 10.1600 0.0360

13 10 C9 CT M 10.6300 3.4660 9.3860 0.0220

14 13 H13 HP E 11.0550 3.6360 8.5230 0.0870

15 13 H14 HP E 10.5560 4.2680 9.9310 0.0870

16 13 N2 N3 M 11.5670 2.5720 10.1550 -0.1590

17 16 H15 H E 11.3440 2.5970 11.1800 0.3210

18 16 H16 H E 12.4440 2.9360 10.0810 0.3210

19 16 C12 CA M 11.6320 1.1770 9.7090 0.0410

20 19 C15 CA B 12.0500 0.8920 8.4350 -0.1790

21 20 C19 CA S 12.1090 -0.4430 8.0290 -0.1250

22 21 H24 HA E 12.3930 -0.6770 7.1520 0.1680

23 20 H20 HA E 12.2450 1.7160 7.7480 0.1870

24 19 C14 CA M 11.2810 0.1780 10.5990 -0.1790

25 24 H19 HA E 10.9910 0.4740 11.5530 0.1870

26 24 C18 CA M 11.3630 -1.1520 10.1720 -0.1250

27 26 H23 HA E 11.1160 -1.8970 10.7990 0.1680

28 26 C22 CA M 11.7610 -1.4680 8.8910 0.0500

29 28 C24 CT M 11.8130 -2.9090 8.4140 -0.2020

30 29 H27 HC E 12.0600 -3.4320 9.2050 0.1630

31 29 H28 HC E 12.5260 -3.0490 7.7120 0.1630

32 29 C25 CA M 10.4720 -3.3600 7.8680 0.0500

33 32 C28 CA B 10.2070 -3.3150 6.5160 -0.1250

34 33 H34 HA E 10.9010 -3.0260 5.9530 0.1680

35 33 C31 CA S 8.9840 -3.7100 5.9990 -0.1790

36 35 H38 HA E 8.8100 -3.6360 4.9980 0.1870

37 32 C27 CA M 9.4770 -3.8280 8.7130 -0.1250

38 37 H33 HA E 9.6540 -3.9520 9.7010 0.1680

39 37 C30 CA M 8.2340 -4.2160 8.2190 -0.1790

40 39 H37 HA E 7.5120 -4.5620 8.8100 0.1870

41 39 C33 CA M 8.0130 -4.1550 6.8650 0.0410

42 41 N4 N3 M 6.7140 -4.5820 6.3210 -0.1590

43 42 H43 H E 6.8880 -5.0810 5.4640 0.3210

44 42 H44 H E 6.3110 -5.2840 6.9220 0.3210

45 42 C34 CT M 5.7620 -3.4460 6.0530 0.0220

46 45 H41 HP E 5.6920 -2.9580 6.9790 0.0870

47 45 H42 HP E 6.1740 -2.8450 5.3570 0.0870

48 45 C32 CT M 4.4150 -3.9920 5.6320 -0.0060

49 48 H39 HC E 3.9240 -4.4490 6.4620 0.0360

50 48 H40 HC E 4.5240 -4.6970 4.8320 0.0360

51 48 C29 CT M 3.4910 -2.8970 5.1270 -0.0060

52 51 H35 HC E 3.8180 -2.5970 4.2010 0.0360

53 51 H36 HC E 3.4800 -2.1680 5.7230 0.0360

54 51 C26 CT M 2.1000 -3.4660 4.9750 0.0220

55 54 H31 HP E 2.1740 -4.2680 4.4300 0.0870

56 54 H32 HP E 1.6750 -3.6360 5.8380 0.0870

57 54 N3 N3 M 1.1630 -2.5720 4.2060 -0.1590

58 57 H29 H E 1.3860 -2.5970 3.1810 0.3210

59 57 H30 H E 0.2870 -2.9360 4.2800 0.3210

60 57 C23 CA M 1.0980 -1.1770 4.6510 0.0410

61 60 C21 CA B 0.6800 -0.8920 5.9260 -0.1790

62 61 C17 CA S 0.6210 0.4430 6.3320 -0.1250

63 62 H22 HA E 0.3370 0.6770 7.2090 0.1680

64 61 H26 HA E 0.4850 -1.7160 6.6130 0.1870

65 60 C20 CA M 1.4490 -0.1780 3.7620 -0.1790

66 65 H25 HA E 1.7390 -0.4740 2.8080 0.1870

67 65 C16 CA M 1.3680 1.1520 4.1890 -0.1250

68 67 H21 HA E 1.6150 1.8970 3.5620 0.1680

69 67 C13 CA M 0.9690 1.4680 5.4700 0.0500

70 69 C11 CT M 0.9180 2.9090 5.9470 -0.2020

71 70 H17 HC E 0.6700 3.4320 5.1560 0.1630

72 70 H18 HC E 0.2040 3.0490 6.6490 0.1630

73 70 C10 CA M 2.2580 3.3600 6.4930 0.0500

74 73 C7 CA M 2.5230 3.3150 7.8450 -0.1250

75 74 H11 HA E 1.8290 3.0260 8.4080 0.1680

76 74 C4 CA M 3.7460 3.7100 8.3620 -0.1790

77 76 H7 HA E 3.9200 3.6360 9.3630 0.1870

78 76 C2 CA M 4.7170 4.1550 7.4960 0.0410

79 78 C5 CA M 4.4960 4.2160 6.1410 -0.1790

80 79 H8 HA E 5.2190 4.5620 5.5500 0.1870

81 79 C8 CA M 3.2530 3.8280 5.6480 -0.1250

82 81 H12 HA E 3.0760 3.9520 4.6600 0.1680

ACPHDRg

1 0 C9 CT M 8.8670 0.4160 5.7510 -0.2240

2 1 H9 HC E 8.9230 -0.2940 4.9110 0.0720

3 1 H10 HC E 9.5730 0.2710 6.4410 0.0720

4 1 H11 HC E 8.8050 1.3550 5.2700 0.0720

5 1 C7 CA M 7.5660 0.2010 6.4970 0.0850

6 5 C3 CA M 7.5280 -0.4830 7.7080 -0.3420

7 6 H1 HA E 8.4320 -0.8360 8.0920 0.1860

8 6 C1 CA M 6.3670 -0.6930 8.4050 0.0850

9 8 C4 CT 3 6.4090 -1.4840 9.6680 -0.2240

10 9 H2 HC E 5.9820 -1.0390 10.5410 0.0720

11 9 H3 HC E 5.8810 -2.2130 9.6220 0.0720

12 9 H4 HC E 7.3090 -1.5810 10.0670 0.0720

13 8 C2 CA M 5.1640 -0.2010 7.8640 0.0850

14 13 C5 CT 3 3.8630 -0.4160 8.6100 -0.2240

15 14 H5 HC E 3.8070 0.2940 9.4490 0.0720

16 14 H6 HC E 3.1570 -0.2710 7.9200 0.0720

17 14 H7 HC E 3.9250 -1.3550 9.0900 0.0720

18 13 C6 CA M 5.2020 0.4830 6.6530 -0.3420

19 18 H8 HA E 4.2980 0.8360 6.2690 0.1860

20 18 C8 CA M 6.3630 0.6930 5.9560 0.0850

21 20 C10 CT M 6.3210 1.4840 4.6920 -0.2240

22 21 H12 HC E 5.4220 1.5810 4.2940 0.0720

23 21 H13 HC E 6.7480 1.0390 3.8200 0.0720

24 21 H14 HC E 6.8490 2.2130 4.7390 0.0720

AHOYEBh

1 0 O10 O M -4.3250 11.2780 19.9670 -0.2640

2 1 C65 C M -4.7040 10.1240 19.7950 0.3790

3 2 C69 CT 3 -6.1430 9.7240 19.9880 -0.2650

4 3 H67 HC E -6.4210 9.9400 20.9020 0.0700

5 3 H68 HC E -6.2380 8.7600 19.8360 0.0950

6 3 H69 HC E -6.7060 10.2110 19.3510 0.1020

7 2 N6 N M -3.8640 9.1340 19.4380 -0.3450

8 7 H60 H E -4.2030 8.3370 19.2890 0.3030

9 7 C58 CA M -2.4550 9.3040 19.2860 -0.0020

10 9 C53 CA B -1.9030 10.1880 18.3840 -0.2640

11 10 C48 CA S -0.5220 10.3400 18.2940 -0.0190

12 11 H46 HA E -0.1540 10.9200 17.6360 0.1090

13 10 H52 HA E -2.4710 10.6980 17.8180 0.1810

14 9 C52 CA M -1.6110 8.5560 20.0850 -0.0090

15 14 H51 HA E -1.9790 7.9160 20.6840 0.1100

16 14 C47 CA M -0.2500 8.7230 20.0250 -0.2050

17 16 H45 HA E 0.3070 8.1960 20.5860 0.1330

18 16 C38 CA M 0.3300 9.6490 19.1570 0.0790

19 18 N3 NH M 1.7200 9.9060 19.2230 -0.2330

20 19 C37 CT 3 2.2730 10.8550 18.2990 -0.0440

21 20 O4 OS S 2.2930 10.3760 16.9380 -0.1560

22 21 C26 CA S 2.7500 9.0790 16.8000 0.0230

23 22 C22 CA B 2.9750 8.6170 15.4990 -0.0510

24 23 C16 CA S 3.4750 7.3280 15.3640 -0.2950

25 24 H12 HA E 3.6380 6.9960 14.4870 0.1890

26 23 C28 CT B 2.7270 9.5330 14.2960 0.0540

27 26 H24 HC E 2.6850 10.4650 14.6520 0.0400

28 26 C33 CT 3 3.9020 9.4980 13.2900 0.0040

29 28 H30 HC E 3.9390 8.6000 12.8720 0.0050

30 28 C41 CT 3 3.7870 10.5410 12.2050 -0.0510

31 30 H34 HC E 2.9760 10.3780 11.6770 0.0120

32 30 H35 HC E 4.5710 10.4900 11.6170 0.0120

33 30 H36 HC E 3.7400 11.4310 12.6090 0.0120

34 28 H80 HC E 4.7500 9.6380 13.7800 0.0090

35 20 H31 H2 E 3.1980 11.0730 18.5770 0.1160

36 20 H32 H2 E 1.7420 11.6900 18.3390 0.1170

37 19 C27 CT M 2.6240 8.7350 19.3190 -0.0360

38 37 H22 H1 E 2.1790 8.0080 19.8230 0.1420

39 37 H23 H1 E 3.4520 8.9850 19.7990 0.0710

40 37 C21 CA M 2.9620 8.2620 17.9090 0.0740

41 40 C15 CA M 3.4570 6.9760 17.7190 0.1460

42 41 O3 OH S 3.6730 6.2560 18.8710 -0.5770

43 42 H19 HO E 3.9710 5.4960 18.6750 0.4020

44 41 C10 CA M 3.7550 6.4940 16.4490 -0.0090

45 44 C5 CT M 4.4020 5.1100 16.2550 0.1040

46 45 H3 HC E 4.8190 4.8670 17.1310 0.0400

47 45 C11 CT 3 5.5410 5.1470 15.2210 0.0040

48 47 H7 HC E 5.9000 4.1240 15.1070 0.0050

49 47 H8 HC E 5.1890 5.5090 14.3700 0.0050

50 47 C17 CT 3 6.6530 5.9340 15.6310 -0.0510

51 50 H13 HC E 6.3910 6.8800 15.6560 0.0120

52 50 H14 HC E 7.3890 5.8160 14.9950 0.0120

53 50 H15 HC E 6.9410 5.6540 16.5230 0.0120

54 45 C3 CA M 3.3520 4.0450 15.9660 -0.1170

55 54 C6 CA S 2.9440 3.7290 14.6740 -0.2950

56 55 H4 HA E 3.3270 4.2220 13.9560 0.1890

57 54 C1 CA M 2.7440 3.3130 17.0040 0.0870

58 57 O1 OS M 3.1560 3.6130 18.3060 -0.1900

59 58 C2 CT M 2.7660 2.5720 19.2660 -0.1920

60 59 H1 H2 E 3.3100 1.7610 19.0980 0.1690

61 59 H2 H2 E 2.9710 2.8890 20.1810 0.1820

62 59 N1 NH M 1.4010 2.2260 19.1980 -0.2900

63 62 C9 CA S 0.4590 3.1100 19.7630 0.0670

64 63 C13 CA B -0.8780 2.6950 19.9270 -0.1010

65 64 H10 HA E -1.1540 1.8560 19.5740 0.1100

66 64 C19 CA B -1.7960 3.4880 20.5930 -0.2050

67 66 H17 HA E -2.6940 3.1910 20.6820 0.1550

68 66 C25 CA B -1.4220 4.7130 21.1350 0.0220

69 68 C20 CA B -0.1110 5.1490 20.9560 -0.2060

70 69 C14 CA S 0.7990 4.3730 20.2660 -0.0330

71 70 H11 HA E 1.6780 4.7030 20.1280 0.0980

72 69 H18 HA E 0.1580 5.9880 21.3120 0.1730

73 68 N2 N B -2.4000 5.4720 21.8320 -0.0420

74 73 H29 H E -3.2390 5.2880 21.6480 -0.0100

75 73 C36 C B -2.1890 6.4520 22.7520 0.4300

76 75 O6 O E -1.0770 6.8020 23.1090 -0.5480

77 75 C46 CT 3 -3.4430 7.0740 23.2920 -0.0450

78 77 H42 HC E -3.7150 6.6040 24.1060 -0.0050

79 77 H43 HC E -4.1530 7.0080 22.6200 0.0290

80 77 H44 HC E -3.2760 8.0170 23.4970 0.0430

81 62 C8 CT M 1.1020 1.6000 17.9010 -0.0060

82 81 H5 H1 E 0.1240 1.5990 17.7520 0.1070

83 81 H6 H1 E 1.4120 0.6600 17.9100 0.0770

84 81 C4 CA M 1.7870 2.3480 16.7720 0.0740

85 84 C7 CA M 1.4560 2.0330 15.4410 0.1580

86 85 O2 OH S 0.5450 1.0210 15.3140 -0.5820

87 86 H9 HO E 0.3780 0.8950 14.5010 0.3860

88 85 C12 CA M 2.0150 2.7350 14.3620 -0.0090

89 88 C18 CT M 1.6260 2.4450 12.9160 0.0620

90 89 H16 HC E 1.2860 1.5050 12.8900 0.0400

91 89 C24 CT 3 2.8140 2.5170 11.9350 0.0040

92 91 H20 HC E 3.1920 3.4330 11.9580 0.0050

93 91 H21 HC E 2.4800 2.3540 11.0190 0.0050

94 91 C31 CT 3 3.9200 1.5230 12.2400 -0.0510

95 94 H26 HC E 4.3190 1.7350 13.1100 0.0120

96 94 H27 HC E 4.6070 1.5770 11.5440 0.0120

97 94 H28 HC E 3.5480 0.6170 12.2620 0.0120

98 89 C23 CA M 0.4770 3.3460 12.4530 -0.0510

99 98 C30 CA S 0.6840 4.6460 12.0270 -0.2950

100 99 H25 HA E 1.5680 4.9930 12.0640 0.1890

101 98 C29 CA M -0.8390 2.8530 12.3830 0.0230

102 101 O5 OS M -1.0380 1.5390 12.7740 -0.1570

103 102 C42 CT M -2.3780 1.0360 12.5610 -0.0190

104 103 H37 H2 E -2.4730 0.7720 11.6100 0.1140

105 103 H38 H2 E -2.5080 0.2230 13.1120 0.1080

106 103 N4 NH M -3.4070 1.9820 12.8840 -0.2900

107 106 C56 CA S -3.6440 2.2860 14.2470 0.1210

108 107 C59 CA B -4.7200 3.1120 14.5760 -0.2200

109 108 H58 HA E -5.2280 3.5220 13.8840 0.1330

110 108 C63 CA B -5.0560 3.3380 15.9060 -0.0050

111 110 H63 HA E -5.7870 3.9070 16.1180 0.1090

112 110 C68 CA B -4.3300 2.7390 16.9270 0.0030

113 112 C64 CA B -3.2370 1.9450 16.6050 -0.2760

114 113 C60 CA S -2.8850 1.7390 15.2810 -0.0170

115 114 H59 HA E -2.1190 1.2170 15.0750 0.1090

116 113 H64 HA E -2.7280 1.5410 17.2980 0.1840

117 112 N7 N B -4.7210 2.9390 18.2950 -0.3490

118 117 H72 H E -4.6670 3.7470 18.6370 0.3020

119 117 C73 C B -5.1660 1.9390 19.0720 0.3830

120 119 O11 O E -5.2610 0.7820 18.6650 -0.2740

121 119 C74 CT 3 -5.5410 2.3140 20.4700 -0.2520

122 121 H74 HC E -5.3700 3.2680 20.6090 0.0660

123 121 H75 HC E -5.0070 1.7900 21.1050 0.0910

124 121 H76 HC E -6.4920 2.1290 20.6150 0.1020

125 106 C44 CT M -3.3090 3.1220 11.9560 0.0320

126 125 H39 H1 E -3.9360 3.8370 12.2360 0.1200

127 125 H40 H1 E -3.5570 2.8330 11.0430 0.0650

128 125 C34 CA M -1.8990 3.6510 11.9590 0.0460

129 128 C43 CA M -1.6260 4.9620 11.5560 0.1520

130 129 O8 OH S -2.7310 5.6770 11.1540 -0.5710

131 130 H48 HO E -2.4960 6.4520 10.9300 0.3860

132 129 C35 CA M -0.3350 5.4820 11.5420 -0.0090

133 132 C45 CT M -0.0450 6.8660 10.9670 0.0960

134 133 H41 HC E -0.8380 7.1000 10.4040 0.0290

135 133 C51 CT 3 1.1540 6.8470 10.0150 0.0040

136 135 H49 HC E 1.0410 6.1040 9.3700 0.0050

137 135 H50 HC E 1.9760 6.6670 10.5380 0.0050

138 135 C57 CT 3 1.3420 8.1390 9.2420 -0.0510

139 138 H55 HC E 0.5320 8.3290 8.7220 0.0110

140 138 H56 HC E 2.1050 8.0510 8.6350 0.0120

141 138 H57 HC E 1.5070 8.8740 9.8700 0.0120

142 133 C50 CA M 0.0380 7.9390 12.0470 -0.0510

143 142 C40 CA M 1.2330 8.2310 12.7000 -0.2950

144 143 H33 HA E 1.9970 7.7080 12.4890 0.1890

145 143 C32 CA M 1.3720 9.2530 13.6490 -0.0080

146 145 C39 CA M 0.2220 9.9950 13.9480 0.1580

147 146 O7 OH S 0.1780 11.0330 14.8450 -0.5790

148 147 H47 HO E 0.9480 11.1670 15.1570 0.3850

149 146 C49 CA M -1.0150 9.6940 13.3750 0.0740

150 149 C55 CT B -2.2410 10.4870 13.7830 -0.0040

151 150 H53 H1 E -2.2770 10.5570 14.7700 0.1070

152 150 H54 H1 E -2.1810 11.4040 13.4130 0.0790

153 149 C54 CA M -1.0770 8.6950 12.4100 0.0230

154 153 O9 OS M -2.2820 8.4130 11.7590 -0.1700

155 154 C61 CT M -3.2970 9.4650 11.9370 -0.0840

156 155 H61 H2 E -3.0300 10.2620 11.4150 0.1420

157 155 H62 H2 E -4.1610 9.1450 11.5770 0.1460

158 155 N5 NH M -3.4690 9.8370 13.2920 -0.3510

159 158 C62 CA M -4.1830 8.9750 14.1570 0.1520

160 159 C67 CA B -4.7010 7.7510 13.7190 -0.0570

161 160 C71 CA S -5.4780 6.9660 14.5660 -0.2340

162 161 H71 HA E -5.8150 6.1330 14.2560 0.1880

163 160 H66 HA E -4.5220 7.4540 12.8340 0.1030

164 159 C66 CA M -4.4730 9.3740 15.4600 -0.1390

165 164 H65 HA E -4.1270 10.1990 15.7820 0.1190

166 164 C70 CA M -5.2510 8.5950 16.2950 -0.2080

167 166 H70 HA E -5.4330 8.8910 17.1780 0.1590

168 166 C72 CA M -5.7660 7.3820 15.8570 0.0390

169 168 N8 N M -6.5340 6.5880 16.7490 -0.0360

170 169 H73 H E -6.2400 6.4960 17.5740 -0.0180

171 169 C75 C M -7.6870 5.9650 16.4210 0.4240

172 171 C76 CT 3 -8.3310 5.1870 17.5620 -0.0470

173 172 H77 HC E -9.1440 5.6490 17.8560 -0.0020

174 172 H78 HC E -7.7030 5.1260 18.3120 0.0360

175 172 H79 HC E -8.5610 4.2840 17.2550 0.0410

176 171 O12 O M -8.1990 6.0340 15.3210 -0.5470

AHOYEBg

1 0 C1 CT M -1.5720 5.4700 17.4400 -0.2420

2 1 H1 HP E -1.5670 4.4900 17.3910 0.1520

3 1 H2 HP E -0.9870 5.7630 18.1700 0.1520

4 1 H3 HP E -2.4870 5.7820 17.6090 0.1520

5 1 N1 N3 M -1.1060 6.0090 16.2160 0.1390

6 5 C3 CT 3 -0.8490 7.3500 16.2120 -0.2420

7 6 H7 HP E -0.5780 7.6280 15.3110 0.1520

8 6 H8 HP E -0.1270 7.5480 16.8440 0.1520

9 6 H9 HP E -1.6560 7.8380 16.4740 0.1520

10 5 C4 CT 3 -1.9490 5.6750 15.1130 -0.2420

11 10 H10 HP E -1.7470 6.2660 14.3560 0.1520

12 10 H11 HP E -2.8880 5.7840 15.3730 0.1520

13 10 H12 HP E -1.7900 4.7440 14.8500 0.1520

14 5 C2 CT M 0.0110 5.4210 15.9250 -0.2420

15 14 H4 HP E -0.0340 4.4800 16.1940 0.1520

16 14 H5 HP E 0.7460 5.8620 16.4010 0.1520

17 14 H6 HP E 0.1670 5.4770 14.9590 0.1520

AJUROMh

1 0 C16 CT M -3.9180 15.0560 14.6610 -0.2410

2 1 H13 HC E -4.8220 14.7030 14.8040 0.0500

3 1 H14 HC E -3.7240 15.0770 13.7020 0.0500

4 1 H15 HC E -3.8590 15.9620 15.0280 0.0500

5 1 C12 CT M -2.9090 14.1580 15.3630 0.2720

6 5 H9 HC E -1.9990 14.5360 15.1870 -0.0270

7 5 C17 CT 3 -3.1200 14.1700 16.8560 -0.2410

8 7 H16 HC E -4.0210 13.8460 17.0590 0.0500

9 7 H17 HC E -3.0150 15.0830 17.1930 0.0500

10 7 H18 HC E -2.4580 13.5860 17.2840 0.0500

11 5 C8 CT M -2.9310 12.7320 14.7940 -0.0990

12 11 H5 HC E -2.3190 12.1650 15.3260 0.0220

13 11 H6 HC E -3.8450 12.3620 14.8890 0.0220

14 11 C4 CT M -2.5200 12.6560 13.3250 0.0210

15 14 H3 HC E -3.1280 13.2750 12.8270 0.0730

16 14 C2 CA M -2.6870 11.2850 12.6980 0.0100

17 16 C5 CA S -2.5460 10.0940 13.4040 -0.2970

18 17 H4 HA E -2.3530 10.1490 14.3330 0.1740

19 16 C1 CA M -2.9870 11.1680 11.3420 0.1790

20 19 O1 OH S -3.1950 12.3340 10.6240 -0.5440

21 20 H1 HO E -3.3500 12.1850 9.8400 0.4120

22 19 C3 CA M -3.0630 9.9350 10.7230 -0.2650

23 22 H2 HA E -3.2410 9.8830 9.7920 0.1750

24 22 C6 CA M -2.8810 8.7700 11.4590 0.1790

25 24 O2 OH S -2.9520 7.5990 10.7440 -0.5440

26 25 H7 HO E -2.7040 6.9270 11.1650 0.4120

27 24 C9 CA M -2.6670 8.8280 12.8400 0.0100

28 27 C13 CT M -2.5680 7.5450 13.6580 0.0210

29 28 H10 HC E -3.1580 6.8740 13.2100 0.0730

30 28 C19 CT 3 -3.0910 7.7070 15.0890 -0.0990

31 30 H19 HC E -3.9860 8.1280 15.0520 0.0220

32 30 H20 HC E -2.4910 8.3260 15.5760 0.0220

33 30 C24 CT 3 -3.1960 6.4010 15.8830 0.2720

34 33 H23 HC E -2.2920 5.9740 15.9040 -0.0270

35 33 C29 CT 3 -3.6140 6.6990 17.3230 -0.2410

36 35 H29 HC E -2.9480 7.2840 17.7400 0.0500

37 35 H30 HC E -3.6730 5.8600 17.8260 0.0500

38 35 H31 HC E -4.4880 7.1430 17.3250 0.0500

39 33 C30 CT 3 -4.1790 5.4020 15.2690 -0.2410

40 39 H32 HC E -4.1800 4.5760 15.7990 0.0500

41 39 H33 HC E -3.9080 5.2000 14.3490 0.0500

42 39 H34 HC E -5.0800 5.7890 15.2670 0.0500

43 28 C18 CA M -1.1490 6.9840 13.6110 0.0100

44 43 C23 CA S -0.1150 7.5630 14.3360 -0.2970

45 44 H22 HA E -0.3110 8.3450 14.8380 0.1740

46 43 C22 CA M -0.8190 5.8680 12.8410 0.1790

47 46 O5 OH S -1.8200 5.3240 12.0530 -0.5440

48 47 H27 HO E -1.5620 4.4770 11.8940 0.4120

49 46 C27 CA M 0.4670 5.3460 12.8290 -0.2650

50 49 H28 HA E 0.6710 4.5890 12.2930 0.1750

51 49 C34 CA M 1.4510 5.9400 13.6020 0.1790

52 51 O7 OH S 2.7430 5.4560 13.6270 -0.5440

53 52 H46 HO E 2.8160 4.6910 13.3190 0.4120

54 51 C28 CA M 1.1870 7.0750 14.3760 0.0100

55 54 C35 CT M 2.2490 7.7080 15.2690 0.0210

56 55 H37 HC E 3.0040 7.0540 15.3140 0.0730

57 55 C40 CT 3 1.7330 7.8660 16.6980 -0.0990

58 57 H47 HC E 0.9460 8.4670 16.6870 0.0220

59 57 H48 HC E 2.4350 8.3020 17.2430 0.0220

60 57 C42 CT 3 1.3380 6.5490 17.3690 0.2720

61 60 H49 HC E 0.6870 6.0840 16.7690 -0.0270

62 60 C43 CT 3 0.6430 6.7990 18.7000 -0.2410

63 62 H51 HC E 0.3930 5.9430 19.1040 0.0500

64 62 H52 HC E -0.1630 7.3380 18.5520 0.0500

65 62 H53 HC E 1.2510 7.2790 19.3000 0.0500

66 60 C44 CT 3 2.5330 5.6240 17.5620 -0.2410

67 66 H54 HC E 3.2110 6.0760 18.1060 0.0500

68 66 H55 HC E 2.9150 5.3970 16.6880 0.0500

69 66 H56 HC E 2.2440 4.8060 18.0150 0.0500

70 55 C37 CA M 2.8220 8.9850 14.6460 0.0100

71 70 C41 CA B 3.8510 8.9040 13.6970 0.1790

72 71 C36 CA B 4.4120 10.0570 13.1680 -0.2650

73 72 C31 CA S 3.9330 11.3070 13.5190 0.1790

74 73 O6 OH S 4.5380 12.3790 12.8930 -0.5440

75 74 H38 HO E 4.0430 13.0420 12.9960 0.4120

76 72 H39 HA E 5.1350 9.9860 12.5560 0.1750

77 71 O8 OH S 4.3740 7.7250 13.2280 -0.5440

78 77 H50 HO E 3.9220 7.0380 13.5020 0.4120

79 70 C32 CA M 2.3800 10.2620 14.9850 -0.2970

80 79 H35 HA E 1.6840 10.3350 15.6280 0.1740

81 79 C25 CA M 2.8950 11.4430 14.4430 0.0100

82 81 C21 CT M 2.3600 12.8240 14.8020 0.0210

83 82 H21 HC E 3.1190 13.4600 14.6680 0.0730

84 82 C26 CT 3 1.9370 12.9650 16.2660 -0.0990

85 84 H25 HC E 2.6800 12.6530 16.8400 0.0220

86 84 H26 HC E 1.1620 12.3710 16.4300 0.0220

87 84 C33 CT 3 1.5570 14.3970 16.6880 0.2720

88 87 H36 HC E 0.7640 14.6820 16.1520 -0.0270

89 87 C38 CT 3 2.6760 15.3790 16.4410 -0.2410

90 89 H40 HC E 2.4350 16.2510 16.8190 0.0500

91 89 H41 HC E 2.8220 15.4720 15.4740 0.0500

92 89 H42 HC E 3.4970 15.0540 16.8650 0.0500

93 87 C39 CT 3 1.1700 14.4120 18.1630 -0.2410

94 93 H43 HC E 1.9450 14.1550 18.7070 0.0500

95 93 H44 HC E 0.4390 13.7770 18.3160 0.0500

96 93 H45 HC E 0.8790 15.3110 18.4160 0.0500

97 82 C15 CA M 1.2590 13.2630 13.8320 0.0100

98 97 C11 CA M -0.0350 12.7680 13.9290 -0.2970

99 98 H8 HA E -0.2120 12.1080 14.5900 0.1740

100 98 C7 CA M -1.0920 13.1820 13.1140 0.0100

101 100 C10 CA M -0.7880 14.1180 12.1260 0.1790

102 101 O3 OH S -1.7430 14.6020 11.2460 -0.5440

103 102 H11 HO E -2.3510 14.0060 11.0720 0.4120

104 101 C14 CA M 0.4980 14.6260 11.9860 -0.2650

105 104 H12 HA E 0.6860 15.2660 11.3090 0.1750

106 104 C20 CA M 1.5040 14.1990 12.8340 0.1790

107 106 O4 OH M 2.8030 14.6620 12.6860 -0.5440

108 107 H24 HO E 2.8290 15.1970 11.9810 0.4120

AJUROMg

1 0 C5 CT M -0.1040 12.0870 9.4120 -0.0570

2 1 H9 HC E 0.3930 12.9170 9.5600 0.0550

3 1 H10 HC E -0.4500 11.7560 10.2680 0.0550

4 1 H11 HC E -0.8510 12.2580 8.8010 0.0550

5 1 C1 CT M 0.8120 11.0540 8.8080 0.0020

6 5 H1 HP E 0.2750 10.2550 8.5760 0.0750

7 5 H2 HP E 1.1830 11.4190 7.9660 0.0750

8 5 N1 N3 M 1.9660 10.6170 9.6920 -0.0440

9 8 C3 CT 3 2.7930 9.6670 8.8620 0.0020

10 9 H5 HP E 2.2130 8.9190 8.5710 0.0750

11 9 H6 HP E 3.0930 10.1430 8.0480 0.0750

12 9 C7 CT 3 4.0100 9.0850 9.5490 -0.0570

13 12 H15 HC E 3.7320 8.6060 10.3550 0.0550

14 12 H16 HC E 4.6240 9.8090 9.7940 0.0550

15 12 H17 HC E 4.4640 8.4660 8.9400 0.0550

16 8 C4 CT 3 1.4510 9.9420 10.9610 0.0020

17 16 H7 HP E 0.8770 10.5870 11.4470 0.0750

18 16 H8 HP E 2.2260 9.7380 11.5430 0.0750

19 16 C8 CT 3 0.6650 8.6690 10.7480 -0.0570

20 19 H18 HC E 1.2370 8.0020 10.3130 0.0550

21 19 H19 HC E -0.1110 8.8560 10.1790 0.0550

22 19 H20 HC E 0.3620 8.3240 11.6120 0.0550

23 8 C2 CT M 2.7820 11.8040 10.1550 0.0020

24 23 H3 HP E 2.2100 12.3720 10.7300 0.0750

25 23 H4 HP E 3.5280 11.4720 10.7140 0.0750

26 23 C6 CT M 3.3590 12.6680 9.0500 -0.0570

27 26 H12 HC E 2.6290 13.0360 8.5100 0.0550

28 26 H13 HC E 3.9450 12.1240 8.4830 0.0550

29 26 H14 HC E 3.8750 13.4000 9.4460 0.0550

AJUXOSh

1 0 O1 OS M 9.0440 5.0560 -0.9840 -0.3170

2 1 C1 CT M 9.3500 3.7500 -0.5150 0.1680

3 2 H1 H1 E 10.2350 3.4890 -0.8140 0.0180

4 2 H2 H1 E 8.7080 3.1130 -0.8650 0.0180

5 2 C3 CT M 9.3000 3.7550 0.9830 0.0470

6 5 H4 H1 E 9.6370 2.9110 1.3230 0.0410

7 5 H5 H1 E 9.8650 4.4650 1.3260 0.0410

8 5 O2 OS M 7.9600 3.9500 1.4280 -0.2800

9 8 C8 CT M 7.8730 3.9710 2.8460 0.0470

10 9 H13 H1 E 8.4590 4.6590 3.1990 0.0410

11 9 H14 H1 E 8.1610 3.1170 3.2040 0.0410

12 9 C11 CT M 6.4660 4.2440 3.2660 0.1680

13 12 H19 H1 E 6.4200 4.3300 4.2300 0.0180

14 12 H20 H1 E 6.1580 5.0750 2.8710 0.0180

15 12 O4 OS M 5.6460 3.1720 2.8370 -0.3170

16 15 C13 CT M 4.2810 3.2790 3.2490 0.0150

17 16 H23 H1 E 3.9660 4.1950 3.1000 0.0770

18 16 C15 CT M 4.1070 2.9140 4.7290 -0.0660

19 18 H26 HC E 4.6630 3.4970 5.2690 0.0340

20 18 H27 HC E 3.1830 3.0610 4.9870 0.0340

21 18 C18 CT M 4.4750 1.4820 5.0110 0.0320

22 21 H31 HC E 4.3190 1.2880 5.9500 -0.0010

23 21 H32 HC E 5.4190 1.3510 4.8310 -0.0010

24 21 C20 CT M 3.6620 0.5280 4.1590 0.0320

25 24 H35 HC E 2.7260 0.5870 4.4060 -0.0010

26 24 H36 HC E 3.9580 -0.3820 4.3160 -0.0010

27 24 C19 CT M 3.8260 0.8740 2.6770 -0.0660

28 27 H33 HC E 4.7460 0.7190 2.4120 0.0340

29 27 H34 HC E 3.2600 0.2910 2.1470 0.0340

30 27 C16 CT M 3.4610 2.3140 2.3970 0.0150

31 30 H28 H1 E 2.5160 2.4380 2.6230 0.0770

32 30 O6 OS M 3.6100 2.5140 0.9840 -0.3170

33 32 C17 CT M 3.3040 3.8200 0.5150 0.1680

34 33 H29 H1 E 2.4190 4.0810 0.8140 0.0180

35 33 H30 H1 E 3.9460 4.4570 0.8650 0.0180

36 33 C14 CT M 3.3540 3.8150 -0.9830 0.0470

37 36 H24 H1 E 3.0170 4.6590 -1.3230 0.0410

38 36 H25 H1 E 2.7890 3.1050 -1.3260 0.0410

39 36 O5 OS M 4.6940 3.6200 -1.4280 -0.2800

40 39 C12 CT M 4.7810 3.5990 -2.8460 0.0470

41 40 H21 H1 E 4.1950 2.9110 -3.1990 0.0410

42 40 H22 H1 E 4.4930 4.4530 -3.2040 0.0410

43 40 C10 CT M 6.1880 3.3260 -3.2660 0.1680

44 43 H17 H1 E 6.2340 3.2400 -4.2300 0.0180

45 43 H18 H1 E 6.4960 2.4950 -2.8710 0.0180

46 43 O3 OS M 7.0080 4.3980 -2.8370 -0.3170

47 46 C5 CT M 8.3730 4.2910 -3.2490 0.0150

48 47 H8 H1 E 8.6880 3.3750 -3.1000 0.0770

49 47 C2 CT M 9.1930 5.2560 -2.3970 0.0150

50 49 H3 H1 E 10.1380 5.1320 -2.6230 0.0770

51 49 C4 CT M 8.8280 6.6960 -2.6770 -0.0660

52 51 H6 HC E 7.9080 6.8510 -2.4120 0.0340

53 51 H7 HC E 9.3940 7.2790 -2.1470 0.0340

54 51 C6 CT M 8.9920 7.0420 -4.1590 0.0320

55 54 H9 HC E 9.9280 6.9830 -4.4060 -0.0010

56 54 H10 HC E 8.6960 7.9520 -4.3160 -0.0010

57 54 C9 CT M 8.1790 6.0880 -5.0110 0.0320

58 57 H15 HC E 8.3350 6.2820 -5.9500 -0.0010

59 57 H16 HC E 7.2350 6.2190 -4.8310 -0.0010

60 57 C7 CT M 8.5470 4.6560 -4.7290 -0.0660

61 60 H11 HC E 7.9910 4.0730 -5.2690 0.0340

62 60 H12 HC E 9.4710 4.5090 -4.9870 0.0340

AJUXOSg

1 0 N1 N M 6.3750 1.7500 0.1800 -0.5460

2 1 H1 H E 6.6830 2.3690 0.6400 0.3360

3 1 H2 H E 5.7790 1.9230 -0.3700 0.3360

4 1 C1 CM M 6.7090 0.5170 0.4160 0.2610

5 4 S1 SS E 7.8570 0.0570 1.5440 -0.3870

6 4 C2 CM M 5.9450 -0.5170 -0.4160 0.2610

7 6 S2 SS E 4.7970 -0.0570 -1.5440 -0.3870

8 6 N2 N M 6.2790 -1.7500 -0.1800 -0.5460

9 8 H3 H E 5.9710 -2.3690 -0.6400 0.3360

10 8 H4 H E 6.8750 -1.9230 0.3700 0.3360

AJUXUYh

1 0 C1 CT M 12.4450 7.2400 -5.3850 0.0830

2 1 H1 H1 E 12.6790 8.0740 -5.8240 0.0370

3 1 H2 H1 E 12.3750 6.5480 -6.0610 0.0370

4 1 C2 CT M 13.4950 6.8760 -4.3860 0.0830

5 4 H3 H1 E 13.2800 6.0270 -3.9700 0.0370

6 4 H4 H1 E 14.3570 6.7940 -4.8240 0.0370

7 4 O2 OS M 13.5400 7.9080 -3.4030 -0.3150

8 7 C3 CT M 14.4500 7.6130 -2.3510 0.0830

9 8 H5 H1 E 15.3580 7.5970 -2.6920 0.0370

10 8 H6 H1 E 14.2500 6.7430 -1.9730 0.0370

11 8 C4 CT M 14.3140 8.6710 -1.3030 0.0830

12 11 H7 H1 E 15.0330 8.5920 -0.6560 0.0370

13 11 H8 H1 E 14.3630 9.5500 -1.7100 0.0370

14 11 O3 OS M 13.0540 8.5050 -0.6530 -0.3150

15 14 C5 CT M 12.7300 9.6030 0.1850 0.0830

16 15 H9 H1 E 12.6830 10.4170 -0.3400 0.0370

17 15 H10 H1 E 13.4170 9.7160 0.8600 0.0370

18 15 C6 CT M 11.4090 9.3430 0.8400 0.0830

19 18 H11 H1 E 11.4580 8.5350 1.3750 0.0370

20 18 H12 H1 E 11.1780 10.0830 1.4220 0.0370

21 18 O4 OS M 10.4210 9.1950 -0.1750 -0.3150

22 21 C7 CT M 9.1120 9.0990 0.3820 0.0830

23 22 H13 H1 E 8.8000 9.9790 0.6450 0.0370

24 22 H14 H1 E 9.1300 8.5350 1.1700 0.0370

25 22 C8 CT M 8.1870 8.5170 -0.6370 0.0830

26 25 H15 H1 E 8.4720 7.6180 -0.8660 0.0370

27 25 H16 H1 E 7.2890 8.4660 -0.2740 0.0370

28 25 O5 OS M 8.1900 9.3350 -1.8060 -0.3150

29 28 C9 CT M 7.1740 8.9620 -2.7350 0.0830

30 29 H17 H1 E 7.2010 9.5810 -3.4820 0.0370

31 29 H18 H1 E 6.3110 9.0680 -2.3020 0.0370

32 29 C10 CT M 7.2630 7.5450 -3.2840 0.0830

33 32 H19 H1 E 7.0820 6.9260 -2.5590 0.0370

34 32 H20 H1 E 6.5610 7.4300 -3.9450 0.0370

35 32 O6 OS M 8.5070 7.1810 -3.8840 -0.3150

36 35 C11 CT M 8.9250 8.0890 -4.8970 0.0830

37 36 H21 H1 E 9.1500 8.9440 -4.4960 0.0370

38 36 H22 H1 E 8.2010 8.2320 -5.5250 0.0370

39 36 C12 CT M 10.1180 7.5430 -5.6190 0.0830

40 39 H23 H1 E 9.8970 6.6880 -6.0200 0.0370

41 39 H24 H1 E 10.3780 8.1510 -6.3280 0.0370

42 39 O1 OS M 11.1980 7.3810 -4.7010 -0.3150

AJUXUYg

1 0 S1 SS M 11.0570 3.8660 -5.4410 -0.4160

2 1 C1 CM M 10.7400 3.7840 -3.7920 0.0080

3 2 N1 N B 11.3020 2.9130 -2.9600 -0.3480

4 3 H1 H E 11.8000 2.3360 -3.2760 0.2800

5 3 H2 H E 10.9890 2.7960 -2.1510 0.2800

6 2 N2 N M 9.8200 4.6350 -3.2550 -0.0330

7 6 H3 H E 9.5370 5.2250 -3.6950 0.2290

8 6 N3 N M 9.6340 4.7560 -1.8840 -0.0330

9 8 H4 H E 9.2140 4.1610 -1.5430 0.2290

10 8 C2 CM M 10.3780 5.6150 -1.1330 0.0080

11 10 N4 N B 11.1620 6.4610 -1.7870 -0.3480

12 11 H5 H E 11.1090 6.5550 -2.6280 0.2800

13 11 H6 H E 11.6010 7.0720 -1.3320 0.2800

14 10 S2 SS M 10.2450 5.5770 0.5470 -0.4160

AJUYAFh

1 0 O1 OS M 14.7810 7.2560 12.8140 -0.3170

2 1 C1 CT M 15.5480 6.2180 13.4190 0.1680

3 2 H1 H1 E 16.3520 6.0610 12.9010 0.0180

4 2 H2 H1 E 15.0320 5.3970 13.4350 0.0180

5 2 C3 CT M 15.9180 6.6170 14.8270 0.0470

6 5 H4 H1 E 16.5240 5.9610 15.2060 0.0410

7 5 H5 H1 E 16.3660 7.4770 14.8190 0.0410

8 5 O2 OS M 14.7330 6.6950 15.6160 -0.2800

9 8 C8 CT M 15.0240 6.9720 16.9830 0.0470

10 9 H13 H1 E 15.5880 7.7580 17.0500 0.0410

11 9 H14 H1 E 15.4960 6.2230 17.3790 0.0410

12 9 C11 CT M 13.7290 7.2060 17.7150 0.1680

13 12 H19 H1 E 13.9010 7.3670 18.6550 0.0180

14 12 H20 H1 E 13.2740 7.9800 17.3490 0.0180

15 12 O4 OS M 12.9280 6.0470 17.5560 -0.3170

16 15 C13 CT M 11.6010 6.1770 18.0930 0.0150

17 16 H23 H1 E 11.1890 7.0000 17.7570 0.0770

18 16 C15 CT M 11.6110 6.1960 19.6250 -0.0660

19 18 H26 HC E 12.1250 6.9560 19.9380 0.0340

20 18 H27 HC E 10.7030 6.2890 19.9560 0.0340

21 18 C18 CT M 12.2230 4.9040 20.1660 0.0320

22 21 H31 HC E 12.1950 4.9150 21.1350 -0.0010

23 21 H32 HC E 13.1520 4.8490 19.8930 -0.0010

24 21 C20 CT M 11.4720 3.6900 19.6500 0.0320

25 24 H35 HC E 10.5720 3.6940 20.0110 -0.0010

26 24 H36 HC E 11.9140 2.8840 19.9600 -0.0010

27 24 C19 CT M 11.4020 3.6700 18.1170 -0.0660

28 27 H33 HC E 12.2920 3.5480 17.7510 0.0340

29 27 H34 HC E 10.8510 2.9260 17.8240 0.0340

30 27 C16 CT M 10.8120 4.9770 17.6080 0.0150

31 30 H28 H1 E 9.9010 5.0540 17.9610 0.0770

32 30 O6 OS M 10.7370 5.0630 16.1800 -0.3170

33 32 C17 CT M 9.8080 4.1650 15.5750 0.1680

34 33 H29 H1 E 8.9700 4.1770 16.0640 0.0180

35 33 H30 H1 E 10.1600 3.2620 15.5930 0.0180

36 33 C14 CT M 9.5800 4.6010 14.1490 0.0470

37 36 H24 H1 E 9.2660 5.5180 14.1310 0.0410

38 36 H25 H1 E 8.9040 4.0400 13.7360 0.0410

39 36 O5 OS M 10.8040 4.4930 13.4260 -0.2800

40 39 C12 CT M 10.5950 4.8090 12.0480 0.0470

41 40 H21 H1 E 9.9500 4.1930 11.6660 0.0410

42 40 H22 H1 E 10.2380 5.7080 11.9700 0.0410

43 40 C10 CT M 11.8970 4.7130 11.2880 0.1680

44 43 H17 H1 E 11.7190 4.6530 10.3370 0.0180

45 43 H18 H1 E 12.3790 3.9160 11.5590 0.0180

46 43 O3 OS M 12.6790 5.8620 11.5590 -0.3170

47 46 C5 CT M 13.9690 5.8490 10.9400 0.0150

48 47 H8 H1 E 14.4770 5.0720 11.2560 0.0770

49 47 C2 CT M 14.6680 7.1340 11.3880 0.0150

50 49 H3 H1 E 15.5700 7.1410 11.0070 0.0770

51 49 C4 CT M 13.9330 8.3640 10.8900 -0.0660

52 51 H6 HC E 13.0370 8.3820 11.2630 0.0340

53 51 H7 HC E 14.3990 9.1620 11.1840 0.0340

54 51 C6 CT M 13.8550 8.3460 9.3630 0.0320

55 54 H9 HC E 14.7490 8.4150 8.9920 -0.0010

56 54 H10 HC E 13.3440 9.1120 9.0570 -0.0010

57 54 C9 CT M 13.1970 7.0660 8.8640 0.0320

58 57 H15 HC E 13.2170 7.0480 7.8950 -0.0010

59 57 H16 HC E 12.2690 7.0480 9.1470 -0.0010

60 57 C7 CT M 13.9200 5.8420 9.4110 -0.0660

61 60 H11 HC E 13.4650 5.0400 9.1090 0.0340

62 60 H12 HC E 14.8250 5.8220 9.0630 0.0340

AJUYAFg

1 0 S1 SS M 9.7790 0.9360 13.7720 -0.4160

2 1 C1 CM M 11.4200 0.8850 14.1890 0.0080

3 2 N1 N B 11.9850 -0.1280 14.8230 -0.3480

4 3 H1 H E 11.5140 -0.8330 15.0660 0.2800

5 3 H2 H E 12.8730 -0.1580 14.8890 0.2800

6 2 N2 N M 12.2040 1.9370 13.8600 -0.0330

7 6 H3 H E 11.9260 2.6130 13.4700 0.2290

8 6 N3 N M 13.5680 1.9590 14.1500 -0.0330

9 8 H4 H E 14.0450 1.3970 13.7390 0.2290

10 8 C2 CM M 14.1420 2.9400 14.8680 0.0080

11 10 N4 N B 13.3720 3.9410 15.2770 -0.3480

12 11 H5 H E 13.7850 4.6480 15.6910 0.2800

13 11 H6 H E 12.5250 3.9570 15.0630 0.2800

14 10 S2 SS M 15.7960 2.8710 15.1960 -0.4160

ASOKICh

1 0 C3 CA M 1.4240 0.4200 13.9640 -0.2964

2 1 H3 HA E 1.3360 1.3630 13.9030 0.1453

3 1 C1 CA M 0.5730 -0.3190 14.7520 0.3296

4 3 H1 H4 E -0.1120 0.1390 15.2260 0.0642

5 3 N1 NC M 0.6570 -1.6410 14.8870 -0.5755

6 5 C2 CA M 1.6190 -2.2640 14.2100 0.3296

7 6 H2 H4 E 1.6950 -3.2070 14.2960 0.0642

8 6 C4 CA M 2.5100 -1.6080 13.3920 -0.2964

9 8 H4 HA E 3.1830 -2.0920 12.9260 0.1453

10 8 C5 CA M 2.4130 -0.2460 13.2590 0.0260

11 10 C6 C M 3.3680 0.4330 12.3460 0.7849

12 11 O1 O E 4.3230 -0.0970 11.8730 -0.5604

13 11 O2 OS M 3.0450 1.7170 12.1360 -0.3997

14 13 C7 CT M 3.9370 2.4320 11.2450 0.0844

15 14 H5 H1 E 4.0690 1.9370 10.4450 0.0775

16 14 H6 H1 E 4.7750 2.5810 11.6680 0.0775

17 14 C8 CT M 3.2640 3.7500 10.9390 0.0844

18 17 H7 H1 E 3.9090 4.3840 10.6490 0.0775

19 17 H8 H1 E 2.8250 4.0790 11.7150 0.0775

20 17 O3 OS M 2.2990 3.5090 9.8880 -0.3997

21 20 C9 C M 1.6740 4.5640 9.3850 0.7849

22 21 O4 O E 1.8460 5.6890 9.7840 -0.5604

23 21 C10 CA M 0.7880 4.2070 8.2500 0.0260

24 23 C11 CA M 0.6830 2.9020 7.7920 -0.2964

25 24 H9 HA E 1.1760 2.2020 8.2040 0.1453

26 24 C13 CA M -0.1550 2.6370 6.7210 0.3296

27 26 H11 H4 E -0.2180 1.7460 6.3980 0.0642

28 26 N2 NC M -0.8800 3.5980 6.1260 -0.5755

29 28 C14 CA M -0.7770 4.8460 6.5730 0.3296

30 29 H12 H4 E -1.2900 5.5270 6.1550 0.0642

31 29 C12 CA M 0.0460 5.1850 7.6230 -0.2964

32 31 H10 HA E 0.1030 6.0880 7.9130 0.1453

33 31 C3 CA M -3.8500 -2.9020 12.6350 -0.2964

34 33 H3 HA E -4.3440 -2.2020 12.2220 0.1453

35 33 C1 CA M -3.0120 -2.6370 13.7050 0.3296

36 35 H1 H4 E -2.9490 -1.7460 14.0290 0.0642

37 35 N1 NC M -2.2880 -3.5980 14.3010 -0.5755

38 37 C2 CA M -2.3900 -4.8460 13.8540 0.3296

39 38 H2 H4 E -1.8770 -5.5270 14.2720 0.0642

40 38 C4 CA M -3.2130 -5.1850 12.8040 -0.2964

41 40 H4 HA E -3.2710 -6.0880 12.5140 0.1453

42 40 C5 CA M -3.9560 -4.2070 12.1760 0.0260

43 42 C6 C M -4.8420 -4.5640 11.0420 0.7849

44 43 O1 O E -5.0130 -5.6890 10.6420 -0.5604

45 43 O2 OS M -5.4660 -3.5090 10.5390 -0.3997

46 45 C7 CT M -6.4310 -3.7500 9.4880 0.0844

47 46 H5 H1 E -5.9930 -4.0790 8.7120 0.0775

48 46 H6 H1 E -7.0760 -4.3840 9.7780 0.0775

49 46 C8 CT M -7.1040 -2.4320 9.1820 0.0844

50 49 H7 H1 E -7.9420 -2.5810 8.7590 0.0775

51 49 H8 H1 E -7.2360 -1.9370 9.9820 0.0775

52 49 O3 OS M -6.2130 -1.7170 8.2910 -0.3997

53 52 C9 C M -6.5360 -0.4330 8.0810 0.7849

54 53 O4 O E -7.4900 0.0970 8.5540 -0.5604

55 53 C10 CA M -5.5800 0.2460 7.1680 0.0260

56 55 C11 CA M -4.5910 -0.4200 6.4630 -0.2964

57 56 H9 HA E -4.5030 -1.3630 6.5240 0.1453

58 56 C13 CA M -3.7400 0.3190 5.6750 0.3296

59 58 H11 H4 E -3.0550 -0.1390 5.2010 0.0642

60 58 N2 NC M -3.8250 1.6410 5.5400 -0.5755

61 60 C14 CA M -4.7870 2.2640 6.2170 0.3296

62 61 H12 H4 E -4.8620 3.2070 6.1310 0.0642

63 61 C12 CA M -5.6770 1.6080 7.0350 -0.2964

64 63 H10 HA E -6.3500 2.0920 7.5010 0.1453

65 63 Cl- IM E -0.9190 1.1480 3.4560 -1.0000

66 0 Cl- IM E -3.3260 4.8980 3.5910 -1.0000

67 0 Cl- IM E -2.2490 -1.1480 16.9710 -1.0000

68 0 Cl- IM E 0.1580 -4.8980 16.8360 -1.0000

69 0 Hg1 Hg M -2.2490 2.9280 4.2820 2.0000

70 69 Hg1 Hg M -0.9190 -2.9280 16.1450 2.0000

ASOKICg

1 0 Cl1 Cl M 0.0460 1.2490 10.8330 -0.0282

2 1 C1 CT M -0.9280 -0.0810 10.1330 -0.2698

3 2 Cl2 Cl E -0.5000 -1.6500 11.0490 -0.0282

4 2 H1 H2 E -0.7030 -0.1060 9.0670 0.3261

5 2 C2 CT M -2.2390 0.0810 10.2940 -0.2698

6 5 Cl4 Cl E -2.6680 1.6500 9.3780 -0.0282

7 5 H2 H2 E -2.4640 0.1060 11.3600 0.3261

8 5 Cl3 Cl M -3.2140 -1.2490 9.5930 -0.0282

ATUKEFh

1 0 C5 CT M 7.4430 2.0740 5.7980 0.0220

2 1 H3 HC E 7.3870 2.9910 6.1660 -0.0130

3 1 H4 HC E 6.7360 1.9790 5.1130 -0.0130

4 1 C4 CT M 8.8110 1.8850 5.1340 0.0220

5 4 H1 HC E 8.8790 0.9540 4.8040 -0.0130

6 4 H2 HC E 8.8700 2.4880 4.3500 -0.0130

7 4 C3 CM M 9.9630 2.1590 6.0380 0.2710

8 7 C2 CT B 11.3070 1.4940 5.9060 0.4590

9 8 C16 CT 3 11.2120 -0.0340 6.0030 -0.2070

10 9 H13 HC E 10.7730 -0.3870 5.2020 0.0320

11 9 H14 HC E 10.6890 -0.2790 6.7960 0.0320

12 9 H15 HC E 12.1120 -0.4140 6.0760 0.0320

13 8 C17 CT 3 11.9930 1.9100 4.5840 -0.2070

14 13 H16 HC E 11.5400 1.4770 3.8300 0.0320

15 13 H17 HC E 12.9340 1.6330 4.6030 0.0320

16 13 H18 HC E 11.9410 2.8830 4.4810 0.0320

17 7 N1 NB M 9.9200 2.9930 7.0090 -0.3860

18 17 N2 NB M 11.2180 2.9830 7.6860 -0.3860

19 18 C1 CM M 11.9690 2.1340 7.0930 0.2710

20 19 C15 CT M 13.3480 1.8030 7.5520 0.0220

21 20 H11 HC E 13.3830 0.8400 7.7750 -0.0130

22 20 H12 HC E 13.9740 1.9580 6.8010 -0.0130

23 20 C42 CT M 13.8250 2.5930 8.7480 0.0220

24 23 H57 HC E 13.1100 2.5970 9.4320 -0.0130

25 23 H58 HC E 13.9790 3.5310 8.4690 -0.0130

26 23 C38 CM M 15.0770 2.0660 9.3660 0.2710

27 26 C39 CT B 15.8670 2.8050 10.4070 0.4590

28 27 C40 CT 3 15.0290 3.2440 11.6160 -0.2070

29 28 H51 HC E 14.4020 3.9450 11.3430 0.0320

30 28 H52 HC E 14.5290 2.4750 11.9630 0.0320

31 28 H53 HC E 15.6210 3.5890 12.3160 0.0320

32 27 C41 CT 3 16.5790 4.0200 9.7910 -0.2070

33 32 H54 HC E 15.9210 4.7200 9.5930 0.0320

34 32 H55 HC E 17.2430 4.3660 10.4240 0.0320

35 32 H56 HC E 17.0280 3.7500 8.9620 0.0320

36 26 N12 NB M 15.5600 0.9170 9.0940 -0.3860

37 36 N11 NB M 16.7140 0.7020 9.9670 -0.3860

38 37 C37 CM M 16.8630 1.7300 10.7120 0.2710

39 38 C36 CT M 17.9830 1.8570 11.7090 0.0220

40 39 H49 HC E 18.7530 2.2890 11.2640 -0.0130

41 39 H50 HC E 17.6870 2.4540 12.4410 -0.0130

42 39 C35 CT M 18.4510 0.5380 12.3200 0.0220

43 42 H47 HC E 19.3880 0.6450 12.6200 -0.0130

44 42 H48 HC E 18.4410 -0.1580 11.6150 -0.0130

45 42 C31 CM M 17.6340 0.0590 13.4730 0.2710

46 45 C32 CT B 17.8790 -1.2470 14.1800 0.4590

47 46 C33 CT 3 17.5920 -2.4520 13.2770 -0.2070

48 47 H41 HC E 17.7620 -3.2800 13.7730 0.0320

49 47 H42 HC E 16.6550 -2.4290 12.9900 0.0320

50 47 H43 HC E 18.1750 -2.4160 12.4900 0.0320

51 46 C34 CT 3 19.2800 -1.3600 14.7740 -0.2070

52 51 H44 HC E 19.3770 -2.2280 15.2190 0.0320

53 51 H45 HC E 19.9450 -1.2840 14.0590 0.0320

54 51 H46 HC E 19.4180 -0.6420 15.4260 0.0320

55 45 N10 NB M 16.6720 0.7380 13.9720 -0.3860

56 55 N9 NB M 16.1360 -0.0080 15.1110 -0.3860

57 56 C30 CM M 16.8070 -1.0900 15.2360 0.2710

58 57 C29 CT M 16.5550 -2.0740 16.3410 0.0220

59 58 H39 HC E 16.6110 -2.9910 15.9730 -0.0130

60 58 H40 HC E 17.2620 -1.9790 17.0260 -0.0130

61 58 C28 CT M 15.1870 -1.8850 17.0050 0.0220

62 61 H37 HC E 15.1200 -0.9540 17.3350 -0.0130

63 61 H38 HC E 15.1280 -2.4880 17.7890 -0.0130

64 61 C24 CM M 14.0350 -2.1590 16.1010 0.2710

65 64 C25 CT B 12.6910 -1.4940 16.2330 0.4590

66 65 C26 CT 3 12.7860 0.0340 16.1360 -0.2070

67 66 H31 HC E 13.2260 0.3870 16.9370 0.0320

68 66 H32 HC E 13.3090 0.2790 15.3430 0.0320

69 66 H33 HC E 11.8860 0.4140 16.0630 0.0320

70 65 C27 CT 3 12.0050 -1.9100 17.5550 -0.2070

71 70 H34 HC E 12.4580 -1.4770 18.3090 0.0320

72 70 H35 HC E 11.0640 -1.6330 17.5360 0.0320

73 70 H36 HC E 12.0570 -2.8830 17.6580 0.0320

74 64 N8 NB M 14.0790 -2.9930 15.1300 -0.3860

75 74 N7 NB M 12.7810 -2.9830 14.4530 -0.3860

76 75 C23 CM M 12.0290 -2.1340 15.0460 0.2710

77 76 C22 CT M 10.6500 -1.8030 14.5870 0.0220

78 77 H59 HC E 10.6150 -0.8400 14.3640 -0.0130

79 77 H60 HC E 10.0240 -1.9580 15.3380 -0.0130

80 77 C14 CT M 10.1730 -2.5930 13.3910 0.0220

81 80 H9 HC E 10.8880 -2.5970 12.7070 -0.0130

82 80 H10 HC E 10.0190 -3.5310 13.6700 -0.0130

83 80 C12 CM M 8.9210 -2.0660 12.7730 0.2710

84 83 C13 CT B 8.1310 -2.8050 11.7310 0.4590

85 84 C20 CT 3 8.9700 -3.2440 10.5230 -0.2070

86 85 H25 HC E 9.5960 -3.9450 10.7960 0.0320

87 85 H26 HC E 9.4700 -2.4750 10.1760 0.0320

88 85 H27 HC E 8.3770 -3.5890 9.8230 0.0320

89 84 C21 CT 3 7.4190 -4.0200 12.3480 -0.2070

90 89 H28 HC E 8.0770 -4.7200 12.5460 0.0320

91 89 H29 HC E 6.7550 -4.3660 11.7150 0.0320

92 89 H30 HC E 6.9700 -3.7500 13.1770 0.0320

93 83 N6 NB M 8.4380 -0.9170 13.0450 -0.3860

94 93 N5 NB M 7.2840 -0.7020 12.1720 -0.3860

95 94 C11 CM M 7.1350 -1.7300 11.4270 0.2710

96 95 C10 CT M 6.0150 -1.8570 10.4300 0.0220

97 96 H7 HC E 5.2450 -2.2890 10.8750 -0.0130

98 96 H8 HC E 6.3110 -2.4540 9.6980 -0.0130

99 96 C9 CT M 5.5470 -0.5380 9.8190 0.0220

100 99 H5 HC E 4.6100 -0.6450 9.5190 -0.0130

101 99 H6 HC E 5.5580 0.1580 10.5240 -0.0130

102 99 C7 CM M 6.3640 -0.0590 8.6660 0.2710

103 102 N4 NB M 7.3260 -0.7380 8.1660 -0.3860

104 103 N3 NB M 7.8620 0.0080 7.0280 -0.3860

105 104 C6 CM M 7.1910 1.0900 6.9030 0.2710

106 105 C8 CT M 6.1190 1.2470 7.9590 0.4590

107 106 C19 CT 3 4.7180 1.3600 7.3650 -0.2070

108 107 H22 HC E 4.6210 2.2280 6.9200 0.0320

109 107 H23 HC E 4.0530 1.2840 8.0800 0.0320

110 107 H24 HC E 4.5800 0.6420 6.7130 0.0320

111 106 C18 CT M 6.4060 2.4520 8.8620 -0.2070

112 111 H19 HC E 6.2360 3.2800 8.3660 0.0320

113 111 H20 HC E 7.3430 2.4290 9.1490 0.0320

114 111 H21 HC E 5.8230 2.4160 9.6490 0.0320

ATUKEFg

1 0 C4 CT M 14.4490 -1.4010 11.6900 -0.1970

2 1 H3 HC E 14.2660 -2.0870 12.3670 0.0630

3 1 H4 HC E 14.8100 -1.8250 10.8850 0.0630

4 1 H5 HC E 15.1020 -0.7600 12.0420 0.0630

5 1 C1 CA M 13.1970 -0.6950 11.3560 0.1390

6 5 C2 CA M 12.6990 0.2790 12.2010 -0.2100

7 6 H1 HA E 13.1800 0.4820 12.9960 0.1450

8 6 C5 CA M 11.5410 0.9640 11.9370 -0.2100

9 8 H10 HA E 11.2390 1.6300 12.5450 0.1450

10 8 C6 CA M 10.8010 0.6950 10.7830 0.1390

11 10 C8 CT 3 9.5490 1.4010 10.4480 -0.1970

12 11 H7 HC E 9.7320 2.0870 9.7720 0.0630

13 11 H8 HC E 8.8960 0.7600 10.0960 0.0630

14 11 H9 HC E 9.1890 1.8250 11.2540 0.0630

15 10 C7 CA M 11.2990 -0.2790 9.9380 -0.2100

16 15 H6 HA E 10.8180 -0.4820 9.1430 0.1450

17 15 C3 CA M 12.4570 -0.9640 10.2020 -0.2100

18 17 H2 HA E 12.7590 -1.6300 9.5940 0.1450

AWUGEEh

1 0 C15 CA M 11.3240 -0.5220 1.6230 -0.1410

2 1 C11 CA B 11.1980 -0.1600 2.9190 -0.1710

3 2 C6 CA S 10.9740 1.1720 3.2670 -0.0730

4 3 H3 HA E 10.8950 1.4060 4.1640 0.1080

5 2 H7 HA E 11.2620 -0.8060 3.5860 0.1410

6 1 H10 HA E 11.4660 -1.4120 1.3950 0.1400

7 1 C10 CA M 11.2380 0.4260 0.6620 -0.1710

8 7 H6 HA E 11.3370 0.1860 -0.2300 0.1410

9 7 C5 CA M 11.0050 1.7460 0.9930 -0.0730

10 9 H2 HA E 10.9370 2.3830 0.3180 0.1080

11 9 C2 CA M 10.8730 2.1340 2.3060 -0.0060

12 11 C1 CT M 10.6580 3.6160 2.6630 0.2600

13 12 O1 OH S 10.0740 4.3190 1.5740 -0.5290

14 13 H1 HO E 9.1830 3.9480 1.3640 0.3840

15 12 C4 CA S 12.0220 4.2750 2.9290 -0.0060

16 15 C8 CA B 12.7410 4.8240 1.8970 -0.0730

17 16 H4 HA E 12.3800 4.8360 1.0400 0.1080

18 16 C13 CA B 13.9870 5.3580 2.1140 -0.1710

19 18 H8 HA E 14.4610 5.7250 1.4030 0.1410

20 18 C18 CA B 14.5340 5.3510 3.3600 -0.1410

21 20 C14 CA B 13.8290 4.8190 4.3990 -0.1710

22 21 C9 CA S 12.5790 4.2850 4.1900 -0.0730

23 22 H5 HA E 12.1040 3.9280 4.9050 0.1080

24 21 H9 HA E 14.1960 4.8150 5.2530 0.1410

25 20 H12 HA E 15.3810 5.7080 3.5020 0.1400

26 12 C3 CZ M 9.7720 3.7260 3.8510 -0.0970

27 26 C7 CZ M 9.0230 3.7960 4.7710 -0.1930

28 27 C12 CA M 8.0860 3.9360 5.8540 0.1770

29 28 C17 CA B 8.1320 5.0470 6.7050 -0.1850

30 29 H11 HA E 8.8050 5.6790 6.6020 0.1730

31 29 C20 CA B 7.1840 5.2100 7.6930 -0.1650

32 31 C21 CA B 6.1910 4.2830 7.8650 -0.1540

33 32 C19 CA S 6.1520 3.1580 7.0760 -0.0860

34 33 H13 HA E 5.4900 2.5190 7.2120 0.0840

35 32 H15 HA E 5.5400 4.4140 8.5160 0.1510

36 31 H14 HA E 7.2200 5.9550 8.2470 0.1580

37 28 C16 CA M 7.0950 2.9730 6.0800 0.0740

38 37 N1 N2 M 7.1190 1.8490 5.2110 -0.0600

39 38 N2 N2 M 6.5020 0.8570 5.6580 -0.0600

40 39 C22 CA M 6.5260 -0.2670 4.7890 0.0740

41 40 C24 CA B 7.4690 -0.4520 3.7930 -0.0860

42 41 C27 CA B 7.4300 -1.5770 3.0050 -0.1540

43 42 C29 CA B 6.4370 -2.5040 3.1760 -0.1650

44 43 C26 CA S 5.4900 -2.3410 4.1650 -0.1850

45 44 H17 HA E 4.8160 -2.9730 4.2670 0.1730

46 43 H19 HA E 6.4010 -3.2500 2.6220 0.1580

47 42 H18 HA E 8.0810 -1.7080 2.3530 0.1510

48 41 H16 HA E 8.1310 0.1870 3.6570 0.0840

49 40 C23 CA M 5.5350 -1.2310 5.0150 0.1770

50 49 C25 CZ M 4.5980 -1.0900 6.0980 -0.1930

51 50 C28 CZ M 3.8490 -1.0210 7.0190 -0.0970

52 51 C30 CT M 2.9630 -0.9110 8.2060 0.2600

53 52 O2 OH S 3.5480 -1.6130 9.2950 -0.5290

54 53 H20 HO E 4.4380 -1.2420 9.5050 0.3840

55 52 C32 CA S 1.5990 -1.5690 7.9400 -0.0060

56 55 C35 CA B 0.8800 -2.1180 8.9720 -0.0730

57 56 H23 HA E 1.2410 -2.1300 9.8290 0.1080

58 56 C39 CA B -0.3660 -2.6520 8.7550 -0.1710

59 58 H27 HA E -0.8400 -3.0190 9.4660 0.1410

60 58 C42 CA B -0.9130 -2.6460 7.5090 -0.1410

61 60 C40 CA B -0.2080 -2.1130 6.4700 -0.1710

62 61 C36 CA S 1.0430 -1.5800 6.6800 -0.0730

63 62 H24 HA E 1.5170 -1.2220 5.9640 0.1080

64 61 H28 HA E -0.5750 -2.1090 5.6160 0.1410

65 60 H30 HA E -1.7600 -3.0030 7.3670 0.1400

66 52 C31 CA M 2.7480 0.5710 8.5640 -0.0060

67 66 C33 CA M 2.6160 0.9590 9.8760 -0.0730

68 67 H21 HA E 2.6840 0.3220 10.5510 0.1080

69 67 C37 CA M 2.3830 2.2800 10.2070 -0.1710

70 69 H25 HA E 2.2840 2.5200 11.1000 0.1410

71 69 C41 CA M 2.2970 3.2270 9.2460 -0.1410

72 71 H29 HA E 2.1550 4.1170 9.4750 0.1400

73 71 C38 CA M 2.4230 2.8650 7.9500 -0.1710

74 73 H26 HA E 2.3600 3.5110 7.2830 0.1410

75 73 C34 CA M 2.6470 1.5340 7.6020 -0.0730

76 75 H22 HA E 2.7260 1.3000 6.7050 0.1080

AWUGEEg

1 0 C2 CT M 4.8970 1.5250 2.0940 -0.1050

2 1 H2 H1 E 5.3030 1.5060 2.9640 0.0630

3 1 H3 H1 E 4.7940 0.6290 1.7690 0.0630

4 1 H4 H1 E 4.0360 1.9470 2.1520 0.0630

5 1 N1 N M 5.7410 2.2740 1.1860 -0.0580

6 5 C3 CT 3 5.2400 2.5630 -0.1410 -0.1050

7 6 H5 H1 E 5.7220 3.3060 -0.5110 0.0630

8 6 H6 H1 E 5.3590 1.7940 -0.7030 0.0630

9 6 H7 H1 E 4.3060 2.7820 -0.0900 0.0630

10 5 C1 C M 6.9140 2.7310 1.5750 0.3640

11 10 H1 HA E 7.1760 2.5580 2.4500 0.0660

12 10 O1 O M 7.6810 3.3670 0.8700 -0.5380

AXEZIMh

1 0 C57 CA M 13.8870 0.6860 2.6710 -0.1150

2 1 C44 CA B 13.3700 1.2210 1.4960 -0.1870

3 2 C32 CA S 12.1820 1.9220 1.5270 -0.0860

4 3 H22 HA E 11.8250 2.1680 0.6840 0.1130

5 2 H34 HA E 13.8310 1.1760 0.6580 0.1460

6 1 H49 HA E 14.7700 0.3110 2.6290 0.1330

7 1 C43 CA M 13.2000 0.8600 3.8570 -0.1870

8 7 H33 HA E 13.6470 0.5710 4.6900 0.1460

9 7 C31 CA M 12.0030 1.5530 3.8830 -0.0860

10 9 H21 HA E 11.4320 1.5750 4.6960 0.1130

11 9 C23 CA M 11.4630 2.0830 2.7120 0.0410

12 11 C20 CT M 10.1240 2.8480 2.6660 -0.0490

13 12 C24 CA S 10.4010 4.2630 2.1200 0.0410

14 13 C33 CA B 11.5730 4.9170 2.5000 -0.0860

15 14 C45 CA B 11.8740 6.1900 2.0390 -0.1870

16 15 C58 CA B 11.0010 6.8360 1.1850 -0.1150

17 16 C46 CA B 9.8430 6.2080 0.8000 -0.1870

18 17 C34 CA S 9.5350 4.9230 1.2600 -0.0860

19 18 H24 HA E 8.6050 4.6780 0.9510 0.1130

20 17 H36 HA E 9.2420 6.6670 0.2650 0.1460

21 16 H50 HA E 11.2800 7.6590 0.8360 0.1330

22 15 H35 HA E 12.7260 6.6860 2.3240 0.1460

23 14 H23 HA E 12.1880 4.3830 3.1060 0.1130

24 12 C25 CA S 9.4670 2.8760 4.0600 0.0410

25 24 C35 CA B 8.9280 1.6880 4.5710 -0.0860

26 25 C47 CA B 8.3760 1.6450 5.8370 -0.1870

27 26 C59 CA B 8.3600 2.7830 6.6320 -0.1150

28 27 C48 CA B 8.8960 3.9580 6.1380 -0.1870

29 28 C36 CA S 9.4420 4.0070 4.8520 -0.0860

30 29 H26 HA E 9.7210 4.8850 4.4310 0.1130

31 28 H38 HA E 8.7220 4.7860 6.6580 0.1460

32 27 H51 HA E 7.7740 2.7100 7.4860 0.1330

33 26 H37 HA E 7.9780 0.8110 6.1450 0.1460

34 25 H25 HA E 9.0360 0.8660 4.0400 0.1130

35 12 N1 N M 9.2760 2.0520 1.7300 -0.1720

36 35 H15 H E 9.6030 1.4220 1.2310 0.1440

37 35 C13 C M 7.9420 2.1010 1.5980 0.6520

38 37 O5 O E 7.2160 2.9450 2.1250 -0.5140

39 37 C10 CA M 7.3380 1.0020 0.7500 -0.2490

40 39 C14 CA S 6.7770 -0.1200 1.3620 0.0170

41 40 C15 CA S 6.1200 -1.1050 0.6320 0.0170

42 41 C19 CT 3 5.6500 -2.2300 1.5190 -0.0190

43 42 C21 CT 3 5.8320 -1.6640 2.9580 0.0030

44 43 C18 CT B 6.8610 -0.4960 2.8180 -0.0190

45 44 H16 HC E 7.7540 -0.7600 3.0730 0.0470

46 44 H17 HC E 6.6300 0.2590 3.4380 0.0470

47 43 C26 C B 4.5400 -1.0380 3.4330 0.5230

48 47 O7 O E 3.6900 -0.5980 2.6910 -0.5090

49 47 O8 OS S 4.4730 -0.9490 4.7480 -0.3170

50 49 C49 CT 3 3.3210 -0.2440 5.3190 0.1150

51 50 C60 CT 3 3.7280 0.2350 6.6730 -0.0830

52 51 H52 HC E 2.9900 0.7380 7.0560 0.0320

53 51 H53 HC E 4.0590 -0.4100 7.2770 0.0320

54 51 H54 HC E 4.5450 0.7650 6.5550 0.0320

55 50 H39 H1 E 2.6160 -0.9200 5.3270 0.0630

56 50 H40 H1 E 3.1410 0.4120 4.6960 0.0630

57 43 C27 C B 6.2940 -2.7440 3.9290 0.5230

58 57 O9 O E 5.9270 -3.8870 3.8680 -0.5090

59 57 O10 OS S 7.1520 -2.2670 4.8210 -0.3170

60 59 C50 CT 3 7.6890 -3.2030 5.7980 0.1150

61 60 C61 CT 3 8.0100 -2.4190 7.0290 -0.0830

62 61 H55 HC E 8.6620 -1.8340 6.8380 0.0320

63 61 H56 HC E 7.2110 -1.9590 7.3390 0.0320

64 61 H57 HC E 8.4190 -2.9720 7.7000 0.0320

65 60 H41 H1 E 8.5370 -3.5210 5.3920 0.0630

66 60 H42 H1 E 7.0110 -3.8590 6.0220 0.0630

67 42 H18 HC E 6.2300 -3.0130 1.3900 0.0470

68 42 H19 HC E 4.7230 -2.4480 1.3560 0.0470

69 39 C8 CA M 7.2230 1.1050 -0.6320 0.0170

70 69 C4 CT M 7.6930 2.2300 -1.5190 -0.0190

71 70 H3 HC E 8.6200 2.4480 -1.3560 0.0470

72 70 H4 HC E 7.1130 3.0130 -1.3900 0.0470

73 70 C2 CT M 7.5110 1.6640 -2.9580 0.0030

74 73 C1 C B 8.8030 1.0380 -3.4330 0.5230

75 74 O1 O E 9.6530 0.5980 -2.6910 -0.5090

76 74 O2 OS S 8.8700 0.9490 -4.7480 -0.3170

77 76 C3 CT 3 10.0220 0.2440 -5.3190 0.1150

78 77 C7 CT 3 9.6150 -0.2350 -6.6730 -0.0830

79 78 H7 HC E 10.3530 -0.7380 -7.0560 0.0320

80 78 H8 HC E 9.2840 0.4100 -7.2770 0.0320

81 78 H9 HC E 8.7980 -0.7650 -6.5550 0.0320

82 77 H1 H1 E 10.7270 0.9200 -5.3270 0.0630

83 77 H2 H1 E 10.2020 -0.4120 -4.6960 0.0630

84 73 C6 C B 7.0490 2.7440 -3.9290 0.5230

85 84 O3 O E 7.4160 3.8870 -3.8680 -0.5090

86 84 O4 OS S 6.1910 2.2670 -4.8210 -0.3170

87 86 C12 CT 3 5.6540 3.2030 -5.7980 0.1150

88 87 C17 CT 3 5.3330 2.4190 -7.0290 -0.0830

89 88 H12 HC E 4.9240 2.9720 -7.7000 0.0320

90 88 H13 HC E 6.1320 1.9590 -7.3390 0.0320

91 88 H14 HC E 4.6810 1.8340 -6.8380 0.0320

92 87 H10 H1 E 4.8060 3.5210 -5.3920 0.0630

93 87 H11 H1 E 6.3320 3.8590 -6.0220 0.0630

94 73 C5 CT M 6.4820 0.4960 -2.8180 -0.0190

95 94 H5 HC E 5.5890 0.7600 -3.0730 0.0470

96 94 H6 HC E 6.7130 -0.2590 -3.4380 0.0470

97 94 C9 CA M 6.5660 0.1200 -1.3620 0.0170

98 97 C11 CA M 6.0050 -1.0020 -0.7500 -0.2490

99 98 C16 C M 5.4010 -2.1010 -1.5980 0.6520

100 99 O6 O E 6.1270 -2.9450 -2.1250 -0.5140

101 99 N2 N M 4.0670 -2.0520 -1.7300 -0.1720

102 101 H20 H E 3.7400 -1.4220 -1.2310 0.1440

103 101 C22 CT M 3.2190 -2.8480 -2.6660 -0.0490

104 103 C29 CA S 2.9420 -4.2630 -2.1200 0.0410

105 104 C39 CA B 1.7700 -4.9170 -2.5000 -0.0860

106 105 C53 CA B 1.4690 -6.1900 -2.0390 -0.1870

107 106 C63 CA B 2.3420 -6.8360 -1.1850 -0.1150

108 107 C54 CA B 3.5000 -6.2080 -0.8000 -0.1870

109 108 C40 CA S 3.8080 -4.9230 -1.2600 -0.0860

110 109 H30 HA E 4.7380 -4.6780 -0.9510 0.1130

111 108 H46 HA E 4.1010 -6.6670 -0.2650 0.1460

112 107 H59 HA E 2.0630 -7.6590 -0.8360 0.1330

113 106 H45 HA E 0.6170 -6.6860 -2.3240 0.1460

114 105 H29 HA E 1.1550 -4.3830 -3.1060 0.1130

115 103 C30 CA S 3.8760 -2.8760 -4.0600 0.0410

116 115 C41 CA B 4.4150 -1.6880 -4.5710 -0.0860

117 116 C55 CA B 4.9670 -1.6450 -5.8370 -0.1870

118 117 C64 CA B 4.9830 -2.7830 -6.6320 -0.1150

119 118 C56 CA B 4.4470 -3.9580 -6.1380 -0.1870

120 119 C42 CA S 3.9010 -4.0070 -4.8520 -0.0860

121 120 H32 HA E 3.6220 -4.8850 -4.4310 0.1130

122 119 H48 HA E 4.6210 -4.7860 -6.6580 0.1460

123 118 H60 HA E 5.5690 -2.7100 -7.4860 0.1330

124 117 H47 HA E 5.3650 -0.8110 -6.1450 0.1460

125 116 H31 HA E 4.3070 -0.8660 -4.0400 0.1130

126 103 C28 CA M 1.8800 -2.0830 -2.7120 0.0410

127 126 C37 CA M 1.3400 -1.5530 -3.8830 -0.0860

128 127 H27 HA E 1.9110 -1.5750 -4.6960 0.1130

129 127 C51 CA M 0.1430 -0.8600 -3.8570 -0.1870

130 129 H43 HA E -0.3040 -0.5710 -4.6900 0.1460

131 129 C62 CA M -0.5440 -0.6860 -2.6710 -0.1150

132 131 H58 HA E -1.4270 -0.3110 -2.6290 0.1330

133 131 C52 CA M -0.0270 -1.2210 -1.4960 -0.1870

134 133 H44 HA E -0.4880 -1.1760 -0.6580 0.1460

135 133 C38 CA M 1.1610 -1.9220 -1.5270 -0.0860

136 135 H28 HA E 1.5180 -2.1680 -0.6840 0.1130

AXEZIMg

1 0 O1 OH M 7.9120 -4.0640 -0.3450 -0.5930

2 1 H1 HO E 7.5440 -3.6690 -1.1100 0.4510

3 1 C1 CA M 8.7730 -3.2100 0.2820 0.2820

4 3 C2 CA M 9.1610 -2.0070 -0.2940 -0.3370

5 4 H2 HA E 8.7940 -1.7230 -1.1410 0.1410

6 4 C4 CA M 10.0320 -1.1780 0.3850 0.2820

7 6 O2 OH S 10.4160 0.0380 -0.1330 -0.5930

8 7 H5 HO E 9.9690 0.1970 -0.8550 0.4510

9 6 C6 CA M 10.5410 -1.5330 1.6210 -0.1770

10 9 H6 HA E 11.1590 -1.0410 2.0110 0.1620

11 9 C5 CA M 10.1550 -2.7460 2.1790 -0.2370

12 11 H4 HA E 10.5890 -3.0350 3.0770 0.1820

13 11 C3 CA M 9.2650 -3.5780 1.5310 -0.1770

14 13 H3 HA E 8.9420 -4.4550 1.9340 0.1620

AYIBEPh

1 0 C1 CT M 8.8780 -1.1150 8.4910 -0.1990

2 1 H7 HP E 8.2050 -1.3150 7.8560 0.2630

3 1 H8 HP E 9.7300 -1.1350 8.0970 0.2630

4 1 N1 N3 M 8.6290 0.3260 8.8640 -0.3470

5 4 H3 H E 9.1840 0.5410 9.3860 0.3530

6 4 H4 H E 8.6490 0.8290 8.0700 0.3530

7 4 C12 CT M 7.2830 0.5500 9.4540 0.0430

8 7 H21 HP E 6.6560 0.1980 8.9030 0.0600

9 7 H22 HP E 7.2250 0.0360 10.3000 0.0600

10 7 C24 CT M 7.0470 2.0250 9.6460 0.1660

11 10 H27 HP E 7.7930 2.5050 10.1120 0.0660

12 10 H28 HP E 6.8970 2.4510 8.8900 0.0660

13 10 N6 N3 M 5.8280 2.2290 10.4850 -0.0370

14 13 H25 H E 5.9460 1.8310 11.2840 0.2800

15 13 H26 H E 5.1210 1.8430 10.0830 0.2800

16 13 C23 CT M 5.5530 3.6780 10.6880 0.1660

17 16 H29 HP E 5.5610 4.0550 9.8560 0.0660

18 16 H30 HP E 6.2430 4.0000 11.1720 0.0660

19 16 C22 CT M 4.1930 3.9390 11.3080 0.0430

20 19 H31 HP E 4.0410 4.8470 11.2660 0.0600

21 19 H32 HP E 3.4500 3.4780 10.8100 0.0600

22 19 N5 N3 M 4.1330 3.5360 12.7340 -0.3470

23 22 H33 H E 4.8070 3.9640 13.0790 0.3530

24 22 H34 H E 4.1100 2.5590 12.7570 0.3530

25 22 C21 CT M 2.8410 3.9680 13.3930 -0.1990

26 25 H35 HP E 2.9520 4.9010 13.5220 0.2630

27 25 H36 HP E 2.1280 3.7120 12.7840 0.2630

28 25 C18 CA M 2.6740 3.3140 14.7210 0.0430

29 28 C19 CA B 1.5430 2.5530 14.9910 -0.1570

30 29 C20 CA S 1.4180 1.9010 16.1910 -0.1570

31 30 H23 HA E 0.6640 1.3520 16.4090 0.1830

32 29 H24 HA E 0.9190 2.3790 14.2740 0.1830

33 28 C17 CA M 3.6450 3.4530 15.7070 -0.1570

34 33 H37 HA E 4.4220 4.0000 15.5100 0.1830

35 33 C16 CA M 3.5280 2.7710 16.9010 -0.1570

36 35 H38 HA E 4.1020 2.8110 17.5780 0.1830

37 35 C15 CA M 2.4190 1.9790 17.1410 0.0430

38 37 C14 CT M 2.3520 1.1150 18.3660 -0.1990

39 38 H39 HP E 3.0260 1.3150 19.0010 0.2630

40 38 H40 HP E 1.5010 1.1350 18.7590 0.2630

41 38 N4 N3 M 2.6010 -0.3260 17.9930 -0.3470

42 41 H41 H E 2.0470 -0.5410 17.4700 0.3530

43 41 H42 H E 2.5820 -0.8290 18.7860 0.3530

44 41 C13 CT M 3.9480 -0.5500 17.4030 0.0430

45 44 H43 HP E 4.5740 -0.1980 17.9540 0.0600

46 44 H44 HP E 4.0060 -0.0360 16.5570 0.0600

47 44 C11 CT M 4.1840 -2.0250 17.2110 0.1660

48 47 H19 HP E 3.4380 -2.5050 16.7450 0.0660

49 47 H20 HP E 4.3340 -2.4510 17.9670 0.0660

50 47 N3 N3 M 5.4030 -2.2290 16.3720 -0.0370

51 50 H1 H E 6.1100 -1.8430 16.7730 0.2800

52 50 H2 H E 5.2850 -1.8310 15.5730 0.2800

53 50 C10 CT M 5.6770 -3.6780 16.1690 0.1660

54 53 H17 HP E 5.6690 -4.0540 17.0000 0.0660

55 53 H18 HP E 4.9880 -4.0000 15.6840 0.0660

56 53 C9 CT M 7.0380 -3.9390 15.5490 0.0430

57 56 H15 HP E 7.1890 -4.8470 15.5900 0.0600

58 56 H16 HP E 7.7810 -3.4780 16.0470 0.0600

59 56 N2 N3 M 7.0980 -3.5360 14.1230 -0.3470

60 59 H5 H E 6.4240 -3.9640 13.7770 0.3530

61 59 H6 H E 7.1200 -2.5590 14.1000 0.3530

62 59 C8 CT M 8.3890 -3.9680 13.4630 -0.1990

63 62 H13 HP E 8.2780 -4.9010 13.3340 0.2630

64 62 H14 HP E 9.1030 -3.7120 14.0730 0.2630

65 62 C5 CA M 8.5570 -3.3140 12.1350 0.0430

66 65 C4 CA M 7.5860 -3.4530 11.1500 -0.1570

67 66 H10 HA E 6.8080 -4.0000 11.3470 0.1830

68 66 C3 CA M 7.7020 -2.7710 9.9560 -0.1570

69 68 H9 HA E 7.1290 -2.8110 9.2790 0.1830

70 68 C2 CA M 8.8120 -1.9790 9.7150 0.0430

71 70 C7 CA M 9.8130 -1.9010 10.6660 -0.1570

72 71 H12 HA E 10.5660 -1.3510 10.4470 0.1830

73 71 C6 CA M 9.6870 -2.5530 11.8650 -0.1570

74 73 H11 HA E 10.3120 -2.3790 12.5820 0.1830

AYIBEPg

1 0 O1 O M 7.0040 -0.7490 14.1720 -0.8670

2 1 C1 C M 6.3900 0.0540 13.4200 0.7340

3 2 O3 O E 6.9100 0.9220 12.7060 -0.8670

4 2 C2 C M 4.8410 -0.0540 13.4360 0.7340

5 4 O4 O E 4.2270 0.7490 12.6840 -0.8670

6 4 O2 O M 4.3210 -0.9220 14.1510 -0.8670

BAFZENh

1 0 O11 O M 6.3840 1.9050 4.5390 -0.5790

2 1 C15 C M 6.8250 1.3290 5.5170 0.6250

3 2 O12 OH S 7.2860 1.8860 6.5680 -0.6090

4 3 H22 HO E 7.5140 1.2130 7.2140 0.3480

5 2 C13 CT M 6.8080 -0.2070 5.5850 0.2000

6 5 H19 H1 E 7.8010 -0.5250 5.8630 0.0520

7 5 O9 OS M 6.4640 -0.8050 4.3480 -0.4110

8 7 C11 CT M 7.4890 -0.7630 3.3570 0.0920

9 8 H15 H1 E 7.5880 0.3700 3.6870 0.0330

10 8 H16 H1 E 8.3370 -1.3450 3.6870 0.0330

11 8 C9 CT M 6.9530 -1.3320 2.0820 0.0330

12 11 H11 H1 E 6.5020 -2.2940 2.2790 0.0400

13 11 H12 H1 E 7.7590 -1.4500 1.3730 0.0400

14 11 O7 OS M 5.9710 -0.4510 1.5460 -0.2870

15 14 C7 CT M 5.3220 -1.0320 0.3900 0.0080

16 15 H7 H1 E 6.0640 -1.3330 -0.3350 0.0500

17 15 H8 H1 E 4.7420 -1.8910 0.6910 0.0500

18 15 C5 CT M 4.4110 0.0120 -0.2130 0.1650

19 18 H3 H1 E 3.9200 -0.3880 -1.0880 0.0040

20 18 H4 H1 E 4.9850 0.8850 -0.4900 0.0040

21 18 O1 OS M 3.4500 0.3570 0.7670 -0.3090

22 21 C1 CT M 2.2030 0.8380 0.3090 0.1530

23 22 H1 H1 E 2.3240 1.2330 -0.6890 0.0480

24 22 C3 C B 1.1590 -0.2640 0.2020 0.6020

25 24 O3 O E 0.0000 0.0000 0.0000 -0.6930

26 24 O4 O E 1.6370 -1.4680 0.3170 -0.6930

27 22 C2 CT M 1.7390 1.9330 1.2720 0.2000

28 27 H2 H1 E 0.7750 2.3420 1.0070 0.0520

29 27 C4 C B 2.7650 3.0770 1.2090 0.6250

30 29 O5 OH S 2.7220 3.7680 0.1620 -0.6090

31 30 H21 HO E 2.0040 3.4600 -0.3960 0.3480

32 29 O6 O E 3.5600 3.2200 2.1240 -0.5790

33 27 O2 OS M 1.6080 1.3640 2.5650 -0.4110

34 33 C6 CT M 0.9760 2.2440 3.5020 0.0920

35 34 H5 H1 E 1.4850 3.1970 3.4950 0.0330

36 34 H6 H1 E -0.0560 2.3850 3.2180 0.0330

37 34 C8 CT M 1.0330 1.6640 4.8830 0.0330

38 37 H9 H1 E 0.5630 0.6920 4.8840 0.0400

39 37 H10 H1 E 0.5110 2.3170 5.5680 0.0400

40 37 O8 OS M 2.3960 1.5350 5.2940 -0.2870

41 40 C10 CT M 2.4960 1.1320 6.6650 0.0080

42 41 H13 H1 E 2.0790 1.9040 7.2950 0.0500

43 41 H14 H1 E 1.9470 0.2130 6.8090 0.0500

44 41 C12 CT M 3.9320 0.9130 7.0370 0.1650

45 44 H17 H1 E 3.9990 0.6230 8.0740 0.0040

46 44 H18 H1 E 4.4920 1.8230 6.8810 0.0040

47 44 O10 OS M 4.4630 -0.1280 6.2100 -0.3090

48 47 C14 CT M 5.7320 -0.6370 6.5970 0.1530

49 48 H20 H1 E 5.9620 -0.2370 7.5740 0.0480

50 48 C16 C M 5.7090 -2.1730 6.6690 0.6020

51 50 O14 O E 6.7460 -2.7800 6.9030 -0.6930

52 50 O13 O M 4.5680 -2.7200 6.5030 -0.6930

BAFZENg

1 0 N1 N3 M 1.7870 -3.1520 4.5380 -0.3350

2 1 H1 H E 0.9750 -3.2160 5.2470 0.3540

3 1 H2 H E 2.6120 -3.7570 4.8820 0.3540

4 1 H3 H E 1.4540 -3.5090 3.5750 0.3540

5 1 C1 CT M 2.2240 -1.7470 4.4190 -0.0190

6 5 H4 HP E 1.3920 -1.1480 4.0810 0.1470

7 5 H5 HP E 2.5500 -1.3970 5.3870 0.1470

8 5 C2 CT M 3.3620 -1.6010 3.4380 -0.0190

9 8 H6 HP E 3.0190 -1.8600 2.4470 0.1470

10 8 H7 HP E 4.1680 -2.2600 3.7230 0.1470

11 8 N2 N3 M 3.8420 -0.2010 3.4430 -0.3350

12 11 H8 H E 4.6570 -0.0970 2.7410 0.3540

13 11 H9 H E 4.1860 0.0580 4.4330 0.3540

14 11 H10 H E 3.0360 0.4570 3.1570 0.3540

BAHDEUh

1 0 C29 CT M 11.4660 -5.0500 11.2560 -0.1770

2 1 H14 HC E 10.8110 -4.4180 10.9550 0.0450

3 1 H15 HC E 12.2660 -4.9570 10.7330 0.0450

4 1 H16 HC E 11.1210 -5.9410 11.1560 0.0450

5 1 C22 CT M 11.7810 -4.7940 12.7070 0.0950

6 5 H9 HC E 10.9640 -4.9280 13.2290 0.0370

7 5 C30 CT 3 12.7970 -5.8240 13.1410 -0.1770

8 7 H17 HC E 12.4430 -6.7050 12.9940 0.0450

9 7 H18 HC E 13.6030 -5.7140 12.6330 0.0450

10 7 H19 HC E 12.9900 -5.7100 14.0750 0.0450

11 5 C16 CT M 12.2610 -3.3690 12.9270 -0.0250

12 11 H5 H1 E 13.0520 -3.2170 12.3680 0.1110

13 11 C23 CT 3 12.6030 -2.9860 14.3610 0.0950

14 13 H10 HC E 13.3040 -3.5980 14.6690 0.0370

15 13 C31 CT 3 13.1720 -1.5750 14.4150 -0.1770

16 15 H20 HC E 13.9180 -1.5080 13.8140 0.0450

17 15 H21 HC E 12.4940 -0.9460 14.1560 0.0450

18 15 H22 HC E 13.4630 -1.3800 15.3100 0.0450

19 13 C32 CT 3 11.4360 -3.1320 15.3000 -0.1770

20 19 H23 HC E 11.0980 -4.0290 15.2530 0.0450

21 19 H24 HC E 11.7230 -2.9450 16.1970 0.0450

22 19 H25 HC E 10.7460 -2.5160 15.0470 0.0450

23 11 O1 OS M 11.2170 -2.4760 12.4930 -0.2360

24 23 C7 CA M 11.3320 -1.6760 11.3990 0.0820

25 24 C11 CA B 12.4850 -1.4420 10.6810 -0.1580

26 25 C10 CA B 12.4760 -0.6530 9.5380 -0.1970

27 26 C6 CA B 11.3460 -0.0320 9.0830 -0.1330

28 27 C3 CB E 10.1840 -0.2280 9.8210 -0.0560

29 27 H2 HA E 11.3550 0.4960 8.3160 0.1200

30 26 H3 HA E 13.2680 -0.5420 9.0660 0.1610

31 25 H4 HA E 13.2850 -1.8200 10.9680 0.1490

32 24 C4 CB M 10.1590 -1.0370 10.9620 0.0900

33 32 C2 CR M 8.7980 -1.0240 11.4390 0.0860

34 33 N3 N2 E 8.3610 -1.6750 12.5220 -0.2290

35 33 N1 NA M 8.0370 -0.2480 10.5970 0.1700

36 35 H1 H E 6.9730 0.0090 10.6770 -0.1170

37 35 C1 CR M 8.8420 0.2490 9.6130 0.0830

38 37 N2 N2 M 8.5120 1.0260 8.5880 -0.2290

39 38 C5 CC M 7.2690 1.4290 8.3430 0.0790

40 39 C9 CB S 6.8930 2.3020 7.2400 0.1000

41 40 C14 CB S 5.5090 2.4420 7.3090 -0.0140

42 41 C20 CA B 4.8020 3.2020 6.3860 -0.1070

43 42 H7 HA E 3.8780 3.2960 6.4430 0.1120

44 42 C27 CA B 5.5290 3.8150 5.3780 -0.1920

45 44 C21 CA B 6.8980 3.6820 5.3080 -0.2380

46 45 C15 CA S 7.6300 2.9590 6.2310 0.1880

47 46 O2 OS S 8.9680 2.7880 6.2250 -0.3160

48 47 C28 CT 3 9.8740 3.7510 5.6100 -0.0190

49 48 H13 H1 E 9.3530 4.3960 5.0880 0.1210

50 48 C36 CT 3 10.7660 2.9810 4.6890 0.0820

51 50 H28 HC E 11.1300 2.2140 5.1780 0.0460

52 50 C41 CT 3 11.8770 3.7030 4.0520 -0.1870

53 52 H31 HC E 12.4540 4.0650 4.7270 0.0500

54 52 H32 HC E 11.5300 4.4160 3.5110 0.0500

55 52 H33 HC E 12.3760 3.0970 3.4960 0.0500

56 50 C42 CT 3 9.9280 2.4620 3.5260 -0.1870

57 56 H34 HC E 10.4870 1.9560 2.9300 0.0500

58 56 H35 HC E 9.5450 3.2040 3.0520 0.0500

59 56 H36 HC E 9.2270 1.9000 3.8620 0.0500

60 48 C37 CT 3 10.5610 4.4750 6.7550 0.0820

61 60 H29 HC E 11.1440 5.1470 6.3450 0.0460

62 60 C43 CT 3 11.4740 3.5370 7.5480 -0.1870

63 62 H37 HC E 12.0850 3.1030 6.9480 0.0500

64 62 H38 HC E 10.9430 2.8770 7.9970 0.0500

65 62 H39 HC E 11.9680 4.0450 8.1960 0.0500

66 60 C44 CT 3 9.5990 5.2200 7.6080 -0.1870

67 66 H40 HC E 9.0770 5.8090 7.0610 0.0500

68 66 H41 HC E 10.0810 5.7320 8.2620 0.0500

69 66 H42 HC E 9.0190 4.5980 8.0530 0.0500

70 45 H8 HA E 7.3500 4.0960 4.6080 0.1670

71 44 H12 HA E 5.0840 4.3230 4.7380 0.1660

72 39 N4 NB M 6.1430 1.0910 9.0510 0.0450

73 72 C13 CC M 5.0670 1.6900 8.4630 0.0300

74 73 N6 N2 M 3.8010 1.6750 8.8490 -0.2290

75 74 C26 CR M 3.3640 1.0240 9.9320 0.0860

76 75 C35 CB S 2.0030 1.0370 10.4090 0.0900

77 76 C38 CB S 1.9780 0.2280 11.5500 -0.0560

78 77 C45 CA B 0.8160 0.0320 12.2880 -0.1330

79 78 H43 HA E 0.8070 -0.4960 13.0560 0.1200

80 78 C49 CA B -0.3140 0.6530 11.8330 -0.1970

81 80 C48 CA B -0.3230 1.4420 10.6900 -0.1580

82 81 C40 CA S 0.8300 1.6760 9.9720 0.0820

83 82 O4 OS S 0.9450 2.4760 8.8780 -0.2360

84 83 C54 CT 3 -0.0990 3.3690 8.4440 -0.0250

85 84 H60 H1 E -0.8900 3.2170 9.0040 0.1110

86 84 C55 CT 3 -0.4410 2.9860 7.0100 0.0950

87 86 H61 HC E -1.1420 3.5980 6.7020 0.0370

88 86 C57 CT 3 0.7260 3.1320 6.0720 -0.1770

89 88 H63 HC E 1.4160 2.5160 6.3240 0.0450

90 88 H64 HC E 0.4390 2.9450 5.1740 0.0450

91 88 H65 HC E 1.0640 4.0290 6.1190 0.0450

92 86 C58 CT 3 -1.0100 1.5750 6.9560 -0.1770

93 92 H66 HC E -1.3010 1.3800 6.0610 0.0450

94 92 H67 HC E -0.3320 0.9460 7.2150 0.0450

95 92 H68 HC E -1.7560 1.5080 7.5570 0.0450

96 84 C56 CT 3 0.3810 4.7940 8.6640 0.0950

97 96 H62 HC E 1.1990 4.9280 8.1420 0.0370

98 96 C59 CT 3 0.6970 5.0500 10.1150 -0.1770

99 98 H69 HC E 1.3510 4.4180 10.4160 0.0450

100 98 H70 HC E -0.1040 4.9570 10.6390 0.0450

101 98 H71 HC E 1.0410 5.9410 10.2150 0.0450

102 96 C60 CT 3 -0.6350 5.8240 8.2300 -0.1770

103 102 H72 HC E -0.2810 6.7050 8.3780 0.0450

104 102 H73 HC E -1.4410 5.7140 8.7390 0.0450

105 102 H74 HC E -0.8280 5.7100 7.2960 0.0450

106 81 H46 HA E -1.1230 1.8200 10.4040 0.1490

107 80 H47 HA E -1.1060 0.5420 12.3060 0.1610

108 75 N8 NA M 4.1250 0.2480 10.7740 0.1700

109 108 H27 H E 5.1890 -0.0090 10.6940 -0.1170

110 108 C33 CR M 3.3200 -0.2490 11.7580 0.0830

111 110 N7 N2 M 3.6500 -1.0260 12.7830 -0.2290

112 111 C17 CC M 4.8930 -1.4290 13.0280 0.0790

113 112 C19 CB E 5.2690 -2.3020 14.1310 0.1000

114 112 N5 NB M 6.0190 -1.0910 12.3200 0.0450

115 114 C8 CC M 7.0950 -1.6900 12.9090 0.0300

116 115 C12 CB M 6.6530 -2.4420 14.0630 -0.0140

117 116 C18 CA M 7.3600 -3.2020 14.9850 -0.1070

118 117 H6 HA E 8.2840 -3.2960 14.9280 0.1120

119 117 C24 CA M 6.6340 -3.8150 15.9940 -0.1920

120 119 H11 HA E 7.0780 -4.3230 16.6330 0.1660

121 119 C34 CA M 5.2640 -3.6820 16.0640 -0.2380

122 121 H26 HA E 4.8120 -4.0960 16.7640 0.1670

123 121 C25 CA M 4.5320 -2.9590 15.1410 0.1880

124 123 O3 OS M 3.1940 -2.7880 15.1460 -0.3160

125 124 C39 CT M 2.2880 -3.7510 15.7610 -0.0190

126 125 H30 H1 E 2.8090 -4.3960 16.2830 0.1210

127 125 C47 CT 3 1.3960 -2.9810 16.6820 0.0820

128 127 H45 HC E 1.0320 -2.2140 16.1930 0.0460

129 127 C52 CT 3 2.2340 -2.4620 17.8450 -0.1870

130 129 H54 HC E 1.6750 -1.9560 18.4410 0.0500

131 129 H55 HC E 2.6170 -3.2040 18.3190 0.0500

132 129 H56 HC E 2.9350 -1.9000 17.5090 0.0500

133 127 C53 CT 3 0.2850 -3.7030 17.3190 -0.1870

134 133 H57 HC E -0.2920 -4.0650 16.6440 0.0500

135 133 H58 HC E 0.6330 -4.4160 17.8600 0.0500

136 133 H59 HC E -0.2140 -3.0970 17.8750 0.0500

137 125 C46 CT M 1.6020 -4.4750 14.6160 0.0820

138 137 H44 HC E 1.0180 -5.1470 15.0260 0.0460

139 137 C51 CT 3 2.5630 -5.2200 13.7630 -0.1870

140 139 H51 HC E 3.0860 -5.8090 14.3100 0.0500

141 139 H52 HC E 2.0810 -5.7320 13.1090 0.0500

142 139 H53 HC E 3.1430 -4.5980 13.3190 0.0500

143 137 C50 CT M 0.6880 -3.5370 13.8230 -0.1870

144 143 H48 HC E 0.0770 -3.1030 14.4230 0.0500

145 143 H49 HC E 1.2190 -2.8770 13.3740 0.0500

146 143 H50 HC E 0.1940 -4.0450 13.1750 0.0500

BAHDEUg

1 0 O1 O M 4.2950 5.0100 10.4380 -0.5400

2 1 C1 C M 5.4870 4.7300 10.2300 0.7200

3 2 O2 OH S 6.5490 5.6720 9.5190 -0.6590

4 3 H1 HO E 6.6810 6.0610 8.5440 0.4790

5 2 C2 C M 6.6760 4.0840 11.1410 0.7200

6 5 O4 OH S 5.6130 3.1420 11.8520 -0.6590

7 6 H2 HO E 5.4810 2.7540 12.8270 0.4790

8 5 O3 O M 7.8670 3.8040 10.9340 -0.5400

BAKHIEh

1 0 O1 OS M 1.8780 6.4880 1.8270 -0.3880

2 1 C1 CT M 2.8120 6.6490 0.7980 0.1740

3 2 H1 H1 E 2.3860 7.0630 0.0450 0.0100

4 2 H2 H1 E 3.5300 7.2070 1.1070 0.0100

5 2 C2 CT M 3.3560 5.3460 0.3910 0.1740

6 5 H3 H1 E 3.6260 4.8540 1.1710 0.0100

7 5 H4 H1 E 4.1130 5.4800 -0.1830 0.0100

8 5 O2 OS M 2.3590 4.6100 -0.3090 -0.3880

9 8 C3 CT M 2.8760 3.9340 -1.4100 0.1740

10 9 H5 H1 E 3.2480 4.5680 -2.0270 0.0100

11 9 H6 H1 E 3.5630 3.3310 -1.1160 0.0100

12 9 C4 CT M 1.7960 3.1590 -2.0890 0.1740

13 12 H7 H1 E 1.3390 2.6160 -1.4420 0.0100

14 12 H8 H1 E 2.1840 2.5960 -2.7620 0.0100

15 12 O3 OS M 0.8760 4.0460 -2.6910 -0.3880

16 15 C5 CT M -0.2590 3.3840 -3.2310 0.1740

17 16 H9 H1 E -0.6330 3.9350 -3.9230 0.0100

18 16 H10 H1 E 0.0240 2.5470 -3.6070 0.0100

19 16 C7 CT M -1.3160 3.1150 -2.2120 0.1740

20 19 H13 H1 E -0.9310 2.6810 -1.4470 0.0100

21 19 H14 H1 E -1.9960 2.5520 -2.5900 0.0100

22 19 O4 OS M -1.8780 4.3550 -1.8270 -0.3880

23 22 C9 CT M -2.8120 4.1940 -0.7980 0.1740

24 23 H17 H1 E -2.3860 3.7800 -0.0450 0.0100

25 23 H18 H1 E -3.5300 3.6360 -1.1070 0.0100

26 23 C11 CT M -3.3560 5.4970 -0.3910 0.1740

27 26 H21 H1 E -3.6260 5.9890 -1.1710 0.0100

28 26 H22 H1 E -4.1130 5.3630 0.1830 0.0100

29 26 O6 OS M -2.3590 6.2330 0.3090 -0.3880

30 29 C12 CT M -2.8760 6.9090 1.4100 0.1740

31 30 H23 H1 E -3.2480 6.2750 2.0270 0.0100

32 30 H24 H1 E -3.5630 7.5120 1.1160 0.0100

33 30 C10 CT M -1.7960 7.6840 2.0890 0.1740

34 33 H19 H1 E -1.3390 8.2270 1.4420 0.0100

35 33 H20 H1 E -2.1840 8.2470 2.7620 0.0100

36 33 O5 OS M -0.8760 6.7970 2.6910 -0.3880

37 36 C8 CT M 0.2590 7.4590 3.2310 0.1740

38 37 H15 H1 E 0.6330 6.9080 3.9230 0.0100

39 37 H16 H1 E -0.0240 8.2960 3.6070 0.0100

40 37 C6 CT M 1.3160 7.7280 2.2120 0.1740

41 40 H11 H1 E 0.9310 8.1620 1.4470 0.0100

42 40 H12 H1 E 1.9960 8.2910 2.5900 0.0100

BAKHIEg

1 0 O1 O1 M -1.1020 9.0170 -0.8770 -0.4460

2 1 N2 NO M -0.3720 9.7230 -1.5500 0.7220

3 2 O2 O1 E -0.4890 10.9400 -1.5910 -0.4460

4 2 C2 CA M 0.6720 9.1060 -2.3300 0.0580

5 4 C3 CA S 1.5220 9.9430 -3.0110 -0.2050

6 5 H3 HA E 1.4110 10.8940 -2.9380 0.2270

7 4 C1 CA M 0.7910 7.6990 -2.4120 0.1800

8 7 N1 NH B 0.0150 6.8140 -1.8030 -0.7530

9 8 H1 H E 0.2590 5.8020 -1.9110 0.3960

10 8 H2 H E -0.6560 7.1000 -0.8960 0.3960

11 7 C6 CA M 1.8350 7.2270 -3.2440 -0.2030

12 11 H5 HA E 1.9580 6.2780 -3.3340 0.1700

13 11 C5 CA M 2.6720 8.0540 -3.9190 -0.1350

14 13 H4 HA E 3.3610 7.6950 -4.4820 0.1920

15 13 C4 CA M 2.5140 9.4420 -3.7850 0.0000

16 15 N3 NO M 3.4050 10.3460 -4.4860 0.7560

17 16 O4 O1 E 4.2820 9.8610 -5.1800 -0.4540

18 16 O3 O1 M 3.2630 11.5470 -4.3290 -0.4540

BAMQAHh

1 0 C1 CT M -2.6450 -7.6860 -4.8120 0.1360

2 1 H1 H1 E -2.1980 -7.8120 -5.4630 0.0410

3 1 H2 H1 E -3.5800 -7.3980 -5.1890 0.0410

4 1 C2 CT M -2.7120 -8.8840 -3.9240 0.0260

5 4 H3 H1 E -3.1450 -8.6100 -3.1130 0.0560

6 4 H4 H1 E -3.2220 -9.6790 -4.3870 0.0560

7 4 O2 OS M -1.3930 -9.3380 -3.6890 -0.3020

8 7 C3 CT M -1.3630 -10.5800 -2.9900 0.0260

9 8 H5 H1 E -1.8850 -11.2470 -3.4340 0.0560

10 8 H6 H1 E -1.6450 -10.5200 -2.0760 0.0560

11 8 C4 CT M 0.0310 -11.1020 -2.9830 0.1360

12 11 H7 H1 E -0.1090 -12.0310 -2.6040 0.0410

13 11 H8 H1 E 0.4110 -11.2330 -3.8680 0.0410

14 11 O3 OS M 0.8280 -10.1750 -2.2330 -0.3030

15 14 C5 CA M 2.2020 -10.3860 -2.2300 0.2010

16 15 C6 CA M 2.8200 -11.4850 -2.8070 -0.1690

17 16 H9 HA E 2.3100 -12.2170 -3.2740 0.1220

18 16 C7 CA M 4.1960 -11.5860 -2.7490 -0.2240

19 18 H10 HA E 4.5440 -12.3450 -3.1980 0.1680

20 18 C8 CA M 4.9390 -10.6030 -2.1380 -0.2240

21 20 H11 HA E 5.8040 -10.7770 -2.1510 0.1680

22 20 C9 CA M 4.3290 -9.5020 -1.5850 -0.1690

23 22 H12 HA E 4.7990 -8.6670 -1.1420 0.1220

24 22 C10 CA M 2.9570 -9.3970 -1.6140 0.2010

25 24 O4 OS M 2.2260 -8.3610 -1.0760 -0.3030

26 25 C11 CT M 2.9660 -7.3200 -0.4190 0.1360

27 26 H13 H1 E 3.6250 -6.8990 -1.0570 0.0410

28 26 H14 H1 E 3.4570 -7.7830 0.3870 0.0410

29 26 C12 CT M 1.9880 -6.2960 0.0160 0.0260

30 29 H15 H1 E 1.0450 -6.5290 0.4620 0.0560

31 29 H16 H1 E 2.5960 -5.5590 0.4810 0.0560

32 29 O5 OS M 1.3760 -5.7230 -1.1530 -0.3020

33 32 C13 CT M 0.6140 -4.5640 -0.8530 0.0260

34 33 H17 H1 E 1.1650 -4.0340 -0.4430 0.0560

35 33 H18 H1 E -0.1980 -4.6760 -0.2360 0.0560

36 33 C14 CT M 0.1810 -3.9120 -2.1360 0.1360

37 36 H19 H1 E -0.2220 -2.9790 -2.0100 0.0410

38 36 H20 H1 E 1.0820 -3.7780 -2.6890 0.0410

39 36 O6 OS M -0.7080 -4.7980 -2.8240 -0.3030

40 39 C15 CA M -0.9280 -4.5200 -4.1560 0.2010

41 40 C16 CA M -0.4910 -3.3980 -4.8200 -0.1690

42 41 H21 HA E 0.0090 -2.7800 -4.3120 0.1220

43 41 C17 CA M -0.7840 -3.2360 -6.1840 -0.2240

44 43 H22 HA E -0.3490 -2.3380 -6.6330 0.1680

45 43 C18 CA M -1.4960 -4.2010 -6.8560 -0.2240

46 45 H23 HA E -1.7740 -4.1910 -8.0010 0.1680

47 45 C19 CA M -1.9530 -5.3430 -6.1890 -0.1690

48 47 H24 HA E -2.4130 -6.1440 -6.7650 0.1220

49 47 C20 CA M -1.6670 -5.5080 -4.8380 0.2010

50 49 O1 OS M -2.0300 -6.5990 -4.0890 -0.3030

BAMQAHg

1 0 C1 CR M 1.1590 -7.6480 -4.6840 0.0290

2 1 H1 H5 E 0.4120 -8.2820 -4.6510 0.2580

3 1 N1 NA M 1.5960 -6.8100 -3.7490 -0.1410

4 3 H4 H E 1.1920 -6.5430 -2.9530 0.3750

5 3 C3 CW M 2.5550 -6.0530 -4.2400 -0.1350

6 5 H3 H4 E 2.9930 -5.3740 -3.7170 0.2580

7 5 C2 CW M 2.7430 -6.3680 -5.4990 -0.1350

8 7 H2 H4 E 3.3550 -6.1580 -6.0480 0.2580

9 7 N2 NA M 1.8640 -7.3770 -5.7710 -0.1410

10 9 H5 H E 1.8150 -7.8690 -6.6230 0.3750

BAPRAMh

1 0 O1 OS M 7.8410 9.1120 0.0670 -0.3070

2 1 C1 CT M 8.5310 8.7830 1.2770 0.0720

3 2 H1 H1 E 9.3870 9.2380 1.3030 0.0410

4 2 H2 H1 E 8.6920 7.8280 1.3150 0.0410

5 2 C2 CT M 7.6960 9.2050 2.4450 0.0720

6 5 H3 H1 E 8.2050 9.1060 3.2650 0.0410

7 5 H4 H1 E 7.4460 10.1370 2.3530 0.0410

8 5 O2 OS M 6.5270 8.3930 2.5010 -0.3070

9 8 C3 CT M 5.6400 8.8230 3.5240 0.0720

10 9 H5 H1 E 5.3400 9.7270 3.3430 0.0410

11 9 H6 H1 E 6.0950 8.8170 4.3800 0.0410

12 9 C4 CT M 4.4630 7.8940 3.5660 0.0720

13 12 H7 H1 E 4.7700 6.9830 3.6980 0.0410

14 12 H8 H1 E 3.8860 8.1290 4.3080 0.0410

15 12 O3 OS M 3.7340 7.9860 2.3390 -0.3070

16 15 C5 CT M 2.6820 7.0240 2.3150 0.0720

17 16 H9 H1 E 2.1210 7.1370 3.0970 0.0410

18 16 H10 H1 E 3.0580 6.1300 2.3390 0.0410

19 16 C7 CT M 1.8550 7.1870 1.0750 0.0720

20 19 H23 H1 E 1.0480 6.6550 1.1510 0.0410

21 19 H24 H1 E 1.5960 8.1160 0.9760 0.0410

22 19 O4 OS M 2.5960 6.7720 -0.0670 -0.3070

23 22 C8 CT M 1.9060 7.1010 -1.2770 0.0720

24 23 H13 H1 E 1.0500 6.6460 -1.3030 0.0410

25 23 H14 H1 E 1.7450 8.0560 -1.3150 0.0410

26 23 C9 CT M 2.7410 6.6790 -2.4450 0.0720

27 26 H15 H1 E 2.2320 6.7780 -3.2650 0.0410

28 26 H16 H1 E 2.9910 5.7470 -2.3530 0.0410

29 26 O5 OS M 3.9100 7.4910 -2.5010 -0.3070

30 29 C10 CT M 4.7970 7.0610 -3.5240 0.0720

31 30 H17 H1 E 5.0970 6.1570 -3.3430 0.0410

32 30 H18 H1 E 4.3420 7.0670 -4.3800 0.0410

33 30 C11 CT M 5.9740 7.9900 -3.5660 0.0720

34 33 H19 H1 E 5.6670 8.9010 -3.6980 0.0410

35 33 H20 H1 E 6.5510 7.7550 -4.3080 0.0410

36 33 O6 OS M 6.7030 7.8980 -2.3390 -0.3070

37 36 C12 CT M 7.7550 8.8600 -2.3150 0.0720

38 37 H21 H1 E 8.3160 8.7470 -3.0970 0.0410

39 37 H22 H1 E 7.3790 9.7540 -2.3390 0.0410

40 37 C6 CT M 8.5820 8.6970 -1.0750 0.0720

41 40 H11 H1 E 9.3890 9.2290 -1.1510 0.0410

42 40 H12 H1 E 8.8410 7.7680 -0.9760 0.0410

BAPRAMg

1 0 S1 SS M 6.0350 4.6750 2.4590 -0.4480

2 1 C8 CM M 6.3840 5.1210 0.8470 0.1320

3 2 N3 NT B 6.2200 6.3440 0.3870 -0.3450

4 3 H3 H E 6.3840 6.5440 -0.4570 0.2630

5 3 H4 H E 6.0180 6.9410 0.9320 0.2630

6 2 N2 NT M 6.8300 4.1770 0.0030 -0.3130

7 6 H2 H E 6.9680 3.4070 0.2670 0.3050

8 6 N1 N M 7.2540 4.4840 -1.2830 -0.1880

9 8 H1 H E 6.7500 4.2170 -1.8660 0.2600

10 8 C7 C M 8.6060 4.6440 -1.4930 0.6110

11 10 O1 O E 9.3650 4.7500 -0.5410 -0.4890

12 10 C1 CA M 9.0450 4.7020 -2.9020 -0.1840

13 12 C2 CA M 8.1800 5.1460 -3.9110 -0.1480

14 13 H7 HA E 7.3070 5.3730 -3.6880 0.1020

15 13 C3 CA M 8.5820 5.2560 -5.2220 -0.1470

16 15 H8 HA E 7.9940 5.5550 -5.8770 0.1430

17 15 C4 CA M 9.8900 4.9070 -5.5450 -0.1620

18 17 H9 HA E 10.1810 4.9870 -6.4260 0.1650

19 17 C5 CA M 10.7580 4.4490 -4.5840 -0.1680

20 19 H10 HA E 11.6230 4.2140 -4.8310 0.1400

21 19 C6 CA M 10.3730 4.3250 -3.2360 0.2890

22 21 N4 NH M 11.2450 3.8080 -2.3330 -0.8620

23 22 H5 H E 11.9900 3.6220 -2.5900 0.3900

24 22 H6 H E 11.0960 3.8040 -1.4750 0.3900

BAPREQh

1 0 O1 OS M 1.8450 0.2580 -1.0180 -0.3180

2 1 C1 CT M 1.2410 -0.9900 -1.3610 0.0850

3 2 H1 H1 E 1.7940 -1.4560 -2.0080 0.0370

4 2 H2 H1 E 0.3720 -0.8360 -1.7600 0.0370

5 2 C2 CT M 1.0900 -1.8320 -0.1160 0.0850

6 5 H3 H1 E 0.5220 -1.3560 0.5100 0.0370

7 5 H4 H1 E 0.6370 -2.6560 -0.3540 0.0370

8 5 O2 OS M 2.3150 -2.1610 0.5420 -0.3180

9 8 C3 CT M 3.2240 -2.9140 -0.2570 0.0850

10 9 H5 H1 E 3.5060 -2.3920 -1.0230 0.0370

11 9 H6 H1 E 2.7940 -3.7230 -0.5780 0.0370

12 9 C4 CT M 4.4090 -3.2630 0.5940 0.0850

13 12 H7 H1 E 4.1200 -3.7410 1.3880 0.0370

14 12 H8 H1 E 5.0190 -3.8320 0.0990 0.0370

15 12 O3 OS M 5.0610 -2.0480 0.9640 -0.3180

16 15 C5 CT M 6.2240 -2.2720 1.7570 0.0850

17 16 H9 H1 E 5.9960 -2.7920 2.5430 0.0370

18 16 H10 H1 E 6.8850 -2.7650 1.2450 0.0370

19 16 C7 CT M 6.7800 -0.9380 2.1660 0.0850

20 19 H23 H1 E 7.5000 -1.0640 2.8040 0.0370

21 19 H24 H1 E 6.0860 -0.4100 2.5920 0.0370

22 19 O4 OS M 7.2670 -0.2580 1.0180 -0.3180

23 22 C8 CT M 7.8710 0.9900 1.3610 0.0850

24 23 H13 H1 E 7.3180 1.4560 2.0080 0.0370

25 23 H14 H1 E 8.7400 0.8360 1.7600 0.0370

26 23 C9 CT M 8.0220 1.8320 0.1160 0.0850

27 26 H15 H1 E 8.5900 1.3560 -0.5100 0.0370

28 26 H16 H1 E 8.4750 2.6560 0.3540 0.0370

29 26 O5 OS M 6.7970 2.1610 -0.5420 -0.3180

30 29 C10 CT M 5.8880 2.9140 0.2570 0.0850

31 30 H17 H1 E 5.6060 2.3920 1.0230 0.0370

32 30 H18 H1 E 6.3180 3.7230 0.5780 0.0370

33 30 C11 CT M 4.7030 3.2630 -0.5940 0.0850

34 33 H19 H1 E 4.9920 3.7410 -1.3880 0.0370

35 33 H20 H1 E 4.0930 3.8320 -0.0990 0.0370

36 33 O6 OS M 4.0510 2.0480 -0.9640 -0.3180

37 36 C12 CT M 2.8880 2.2720 -1.7570 0.0850

38 37 H21 H1 E 3.1160 2.7920 -2.5430 0.0370

39 37 H22 H1 E 2.2270 2.7650 -1.2450 0.0370

40 37 C6 CT M 2.3320 0.9380 -2.1660 0.0850

41 40 H11 H1 E 1.6120 1.0640 -2.8040 0.0370

42 40 H12 H1 E 3.0260 0.4100 -2.5920 0.0370

BAPREQg

1 0 S1 SS M 2.6280 2.8620 2.6090 -0.4270

2 1 C4 C M 2.9750 1.1970 2.6310 0.0450

3 2 N3 N B 3.3090 0.5270 1.5530 -0.3050

4 3 H3 H E 3.3920 -0.3070 1.5750 0.2600

5 3 H4 H E 3.3080 0.9310 0.7920 0.2600

6 2 N2 N M 2.9170 0.5370 3.8110 -0.1820

7 6 H2 H E 2.6120 0.9370 4.5060 0.2780

8 6 N1 N M 3.0290 -0.8430 3.8570 -0.1620

9 8 H1 H E 3.7290 -1.1560 4.1540 0.2150

10 8 C3 C M 1.9440 -1.6280 3.6160 0.4940

11 10 O1 O E 0.8580 -1.1850 3.2830 -0.4730

12 10 C1 CV M 2.1830 -3.0810 3.7720 0.1040

13 12 N5 NB M 3.3410 -3.5740 4.1990 -0.3710

14 13 S2 S M 3.1830 -5.1980 4.2620 0.2750

15 14 N6 NB M 1.6400 -5.3200 3.7460 -0.4640

16 15 C2 CR M 1.1960 -4.0870 3.4970 0.5740

17 16 N4 NH M -0.0290 -3.8460 2.9960 -0.9390

18 17 H5 H E -0.6090 -4.4820 2.9780 0.4090

19 17 H6 H E -0.2930 -2.9950 2.9310 0.4090

BAXZABh

1 0 O1 OS M 2.1130 2.0110 9.8650 -0.3150

2 1 C1 CT M 3.3870 2.4830 10.2800 0.0860

3 2 H1 H1 E 3.6030 3.3070 9.8520 0.0360

4 2 H2 H1 E 4.0840 1.7840 10.0280 0.0360

5 2 C2 CT M 3.3810 2.6490 11.7660 0.0860

6 5 H3 H1 E 4.3250 2.8370 12.1120 0.0360

7 5 H4 H1 E 3.0800 1.7990 12.1850 0.0360

8 5 O2 OS M 2.5140 3.7140 12.1300 -0.3150

9 8 C3 CT M 2.3850 3.8000 13.5430 0.0860

10 9 H5 H1 E 3.2960 3.8600 13.9600 0.0360

11 9 H6 H1 E 1.9300 2.9850 13.8940 0.0360

12 9 C4 CT M 1.6060 5.0180 13.9160 0.0860

13 12 H7 H1 E 2.0330 5.8400 13.5150 0.0360

14 12 H8 H1 E 1.6550 5.1600 14.8980 0.0360

15 12 O3 OS M 0.2560 4.8930 13.4890 -0.3150

16 15 C5 CT M -0.5970 5.7520 14.2390 0.0860

17 16 H9 H1 E -0.2220 6.6890 14.1970 0.0360

18 16 H10 H1 E -0.6420 5.4550 15.1750 0.0360

19 16 C6 CT M -1.9690 5.7240 13.6710 0.0860

20 19 H11 H1 E -2.5890 6.2550 14.2520 0.0360

21 19 H12 H1 E -2.3010 4.7920 13.6220 0.0360

22 19 O4 OS M -1.9430 6.3010 12.3770 -0.3150

23 22 C7 CT M -3.2410 6.6090 11.8890 0.0860

24 23 H13 H1 E -3.6510 7.3440 12.4530 0.0360

25 23 H14 H1 E -3.8320 5.8120 11.9530 0.0360

26 23 C8 CT M -3.1220 7.0270 10.4590 0.0860

27 26 H15 H1 E -2.3990 7.6800 10.3490 0.0360

28 26 H16 H1 E -3.9860 7.4620 10.1840 0.0360

29 26 O5 OS M -2.8700 5.8700 9.6850 -0.3150

30 29 C9 CT M -2.6420 6.1510 8.3080 0.0860

31 30 H17 H1 E -1.7120 6.5160 8.1510 0.0360

32 30 H18 H1 E -3.2680 6.8530 7.9840 0.0360

33 30 C10 CT M -2.8870 4.9000 7.5300 0.0860

34 33 H19 H1 E -2.7710 5.0990 6.5570 0.0360

35 33 H20 H1 E -3.8430 4.5960 7.7030 0.0360

36 33 O6 OS M -1.9820 3.8900 7.9660 -0.3150

37 36 C11 CT M -2.2050 2.6580 7.2920 0.0860

38 37 H21 H1 E -1.9150 2.7540 6.3320 0.0360

39 37 H22 H1 E -3.1820 2.4100 7.3540 0.0360

40 37 C12 CT M -1.4150 1.5780 7.9520 0.0860

41 40 H23 H1 E -1.5610 0.7020 7.4710 0.0360

42 40 H24 H1 E -1.7160 1.4720 8.9140 0.0360

43 40 O7 OS M -0.0360 1.9230 7.9390 -0.3150

44 43 C13 CT M 0.7910 0.8210 8.3020 0.0860

45 44 H25 H1 E 0.8510 0.1530 7.5490 0.0360

46 44 H26 H1 E 0.4030 0.3660 9.1090 0.0360

47 44 C14 CT M 2.1570 1.3250 8.6190 0.0860

48 47 H27 H1 E 2.4570 1.9490 7.8820 0.0360

49 47 H28 H1 E 2.8020 0.5760 8.6810 0.0360

BAXZABg

1 0 N1 N1 M -0.7430 3.4490 10.9320 0.0370

2 1 N2 N1 M -0.1870 4.2350 10.4020 0.4540

3 2 C1 CA M 0.5010 5.2300 9.7200 -0.0270

4 3 C2 CA M 0.5050 6.5050 10.2600 -0.1560

5 4 H4 HA E 0.0680 6.6550 11.0990 0.2260

6 4 C3 CA M 1.1260 7.5050 9.5500 -0.1870

7 6 H5 HA E 1.1240 8.4100 9.8810 0.1710

8 6 C4 CA M 1.7300 7.2080 8.3270 0.4630

9 8 O1 OS S 2.3550 8.1040 7.5500 -0.3110

10 9 C7 CT 3 2.3630 9.4880 7.9470 -0.0470

11 10 H1 H1 E 1.4310 9.8250 8.1930 0.1080

12 10 H2 H1 E 2.9870 9.6300 8.7330 0.1080

13 10 H3 H1 E 2.7140 9.9390 7.2000 0.1080

14 8 C5 CA M 1.7150 5.9000 7.8190 -0.1870

15 14 H6 HA E 2.1630 5.7390 6.9800 0.1710

16 14 C6 CA M 1.0870 4.8990 8.5000 -0.1560

17 16 H7 HA E 1.0170 4.0110 8.1470 0.2260

BAYXIIh

1 0 C1 CA M 26.9770 2.8520 -2.3420 0.2240

2 1 O1 OS M 27.0200 4.0860 -2.9460 -0.3260

3 2 C2 CT M 27.2070 4.0580 -4.3870 0.0830

4 3 H1 H1 E 28.0560 3.7380 -4.5580 0.0650

5 3 H2 H1 E 26.4380 3.6600 -4.7460 0.0650

6 3 C3 CT M 27.3210 5.4670 -4.8730 -0.0060

7 6 H3 H1 E 28.0560 5.9910 -4.3470 0.0580

8 6 H4 H1 E 27.5450 5.4560 -5.8500 0.0580

9 6 O2 OS M 26.0740 6.1410 -4.7080 -0.2290

10 9 C4 CT M 26.0340 7.3440 -5.4860 0.0610

11 10 H5 H1 E 26.0970 7.0700 -6.4380 0.0470

12 10 H6 H1 E 26.9200 7.7870 -5.2160 0.0470

13 10 C5 CT M 24.7760 8.0820 -5.2090 0.0300

14 13 H7 H1 E 24.7620 8.2620 -4.3230 0.0600

15 13 H8 H1 E 24.7910 8.9960 -5.6860 0.0600

16 13 O3 OS M 23.6410 7.2920 -5.5680 -0.3510

17 16 C6 CT M 22.5760 8.0850 -6.0970 0.0610

18 17 H9 H1 E 22.9160 8.4520 -6.9780 0.0510

19 17 H10 H1 E 22.3770 8.8570 -5.5450 0.0510

20 17 C7 CT M 21.3690 7.2220 -6.2920 0.0960

21 20 H11 H1 E 21.6390 6.3710 -6.8140 0.0400

22 20 H12 H1 E 20.6450 7.6060 -6.7900 0.0400

23 20 O4 OS M 20.7890 6.7780 -5.0610 -0.3390

24 23 C8 CT M 20.0510 7.7870 -4.3770 0.0510

25 24 H13 H1 E 20.6730 8.4170 -4.0180 0.0520

26 24 H14 H1 E 19.4240 8.2960 -5.0510 0.0520

27 24 C9 CT M 19.1850 7.1050 -3.3530 0.0010

28 27 H15 H1 E 18.4860 6.5010 -3.8060 0.0660

29 27 H16 H1 E 18.6000 7.8470 -2.9370 0.0660

30 27 O5 OS M 20.0400 6.3980 -2.4510 -0.2400

31 30 C10 CT M 19.3530 5.7210 -1.3930 0.0010

32 31 H17 H1 E 18.3730 5.5250 -1.6210 0.0660

33 31 H18 H1 E 19.7930 4.8260 -1.2690 0.0660

34 31 C11 CT M 19.2470 6.5760 -0.1530 0.0510

35 34 H19 H1 E 18.8560 7.4590 -0.3760 0.0520

36 34 H20 H1 E 18.6850 6.1640 0.5400 0.0520

37 34 O6 OS M 20.5480 6.8230 0.3450 -0.3390

38 37 C12 CT M 20.5200 7.5200 1.6050 0.0960

39 38 H21 H1 E 20.0480 8.4000 1.5270 0.0400

40 38 H22 H1 E 20.0200 6.9840 2.2790 0.0400

41 38 C13 CT M 21.9110 7.9080 1.9920 0.0610

42 41 H23 H1 E 22.3200 8.3570 1.2690 0.0510

43 41 H24 H1 E 21.8090 8.5470 2.7720 0.0510

44 41 O7 OS M 22.6550 6.7470 2.3610 -0.3510

45 44 C14 CT M 23.9360 7.0710 2.9250 0.0300

46 45 H25 H1 E 24.2230 6.2240 3.5010 0.0600

47 45 H26 H1 E 23.8820 7.8650 3.4540 0.0600

48 45 C15 CT M 24.9950 7.3310 1.8770 0.0610

49 48 H27 H1 E 23.8820 7.8650 3.4540 0.0470

50 48 H28 H1 E 25.8980 7.5710 2.3260 0.0470

51 48 O8 OS M 25.2930 6.1070 1.1720 -0.2290

52 51 C16 CT M 26.5400 5.5540 1.5580 -0.0060

53 52 H29 H1 E 27.2330 6.0170 1.1280 0.0580

54 52 H30 H1 E 26.6080 5.5340 2.6780 0.0580

55 52 C17 CT M 26.6190 4.1330 1.0900 0.0830

56 55 H31 H1 E 27.4320 3.7730 1.3860 0.0650

57 55 H32 H1 E 25.8410 3.7550 1.3160 0.0650

58 55 O9 OS M 26.6650 4.1220 -0.3620 -0.3260

59 58 C18 CA M 26.7950 2.8650 -0.9230 0.2240

60 59 C19 CA M 26.7930 1.6790 -0.2350 -0.1840

61 60 H33 HA E 26.6930 1.6750 0.7990 0.1240

62 60 C20 CA M 26.9520 0.4700 -0.9000 -0.2180

63 62 H34 HA E 26.9770 -0.3020 -0.4460 0.1650

64 62 C21 CA M 27.1280 0.4520 -2.2770 -0.2180

65 64 H35 HA E 27.2900 -0.4230 -2.7250 0.1650

66 64 C22 CA M 27.1280 1.6390 -2.9860 -0.1840

67 66 H36 HA E 27.2900 1.6660 -3.9940 0.1240

BAYXIIg

1 0 N1 NH M 23.1180 5.9850 -0.6510 -0.9250

2 1 H1 H E 23.7110 5.8880 0.0940 0.4670

3 1 H2 H E 22.2350 6.1470 -0.5170 0.4670

4 1 C1 C2 M 23.6410 6.1820 -1.8610 0.9730

5 4 N3 NH B 24.9350 6.0350 -2.0510 -0.9250

6 5 H5 H E 25.5010 5.8190 -1.4330 0.4670

7 5 H6 H E 25.3020 6.0340 -2.8190 0.4670

8 4 N2 NH M 22.8430 6.5230 -2.8690 -0.9250

9 8 H3 H E 21.8660 6.5010 -2.7490 0.4670

10 8 H4 H E 23.1150 6.5520 -3.6180 0.4670

BECVEKh

1 0 O1 OS M 5.7370 -1.2450 12.6960 -0.3150

2 1 C1 CT M 6.8030 -0.3230 12.5200 0.0770

3 2 H1 H1 E 7.6200 -0.8190 12.4340 0.0400

4 2 H2 H1 E 6.6440 0.1730 11.7060 0.0400

5 2 C2 CT M 6.9620 0.6550 13.6560 0.0770

6 5 H3 H1 E 7.7450 1.1920 13.5120 0.0400

7 5 H4 H1 E 7.0480 0.1780 14.4840 0.0400

8 5 O2 OS M 5.8200 1.4900 13.7070 -0.3150

9 8 C3 CT M 5.8190 2.3010 14.8750 0.0770

10 9 H5 H1 E 5.7980 1.7400 15.6540 0.0400

11 9 H6 H1 E 6.6150 2.8400 14.8900 0.0400

12 9 C4 CT M 4.6210 3.1860 14.8700 0.0770

13 12 H7 H1 E 4.6110 3.7060 14.0630 0.0400

14 12 H8 H1 E 4.6530 3.7730 15.6280 0.0400

15 12 O3 OS M 3.4400 2.3960 14.9350 -0.3150

16 15 C5 CT M 2.2960 3.1360 15.3190 0.0770

17 16 H9 H1 E 2.4500 3.5460 16.1750 0.0400

18 16 H10 H1 E 2.1170 3.8160 14.6670 0.0400

19 16 C7 CT M 1.1400 2.2130 15.4100 0.0770

20 19 H13 H1 E 1.0130 1.7730 14.5670 0.0400

21 19 H14 H1 E 0.3490 2.7080 15.6350 0.0400

22 19 O4 OS M 1.3950 1.2450 16.4170 -0.3150

23 22 C9 CT M 0.3290 0.3230 16.5930 0.0770

24 23 H17 H1 E -0.4880 0.8190 16.6790 0.0400

25 23 H18 H1 E 0.4880 -0.1730 17.4070 0.0400

26 23 C11 CT M 0.1690 -0.6550 15.4580 0.0770

27 26 H21 H1 E -0.6140 -1.1920 15.6020 0.0400

28 26 H22 H1 E 0.0830 -0.1780 14.6290 0.0400

29 26 O6 OS M 1.3120 -1.4900 15.4070 -0.3150

30 29 C12 CT M 1.3120 -2.3010 14.2380 0.0770

31 30 H23 H1 E 1.3330 -1.7400 13.4590 0.0400

32 30 H24 H1 E 0.5170 -2.8400 14.2230 0.0400

33 30 C10 CT M 2.5110 -3.1860 14.2440 0.0770

34 33 H19 H1 E 2.5200 -3.7060 15.0500 0.0400

35 33 H20 H1 E 2.4780 -3.7730 13.4850 0.0400

36 33 O5 OS M 3.6910 -2.3960 14.1780 -0.3150

37 36 C8 CT M 4.8350 -3.1360 13.7940 0.0770

38 37 H15 H1 E 4.6820 -3.5460 12.9380 0.0400

39 37 H16 H1 E 5.0140 -3.8160 14.4460 0.0400

40 37 C6 CT M 5.9910 -2.2130 13.7040 0.0770

41 40 H11 H1 E 6.1190 -1.7730 14.5460 0.0400

42 40 H12 H1 E 6.7820 -2.7080 13.4780 0.0400

BECVEKg

1 0 O1 O M -1.1610 -1.3900 8.5400 -0.4850

2 1 N1 NO M -1.1660 -0.3880 9.2460 0.8330

3 2 O2 O E -2.1230 0.3760 9.2990 -0.4850

4 2 C1 CA M -0.0050 -0.1020 10.0690 -0.0650

5 4 C2 CA M 1.0450 -1.0030 10.0630 -0.2660

6 5 H5 HA E 0.9940 -1.7910 9.5190 0.2010

7 5 C3 CA M 2.1610 -0.7770 10.8320 0.3010

8 7 N2 NH B 3.2250 -1.6510 10.8010 -0.9220

9 8 H1 H E 3.8650 -1.5900 11.2680 0.3790

10 8 H2 H E 3.1500 -2.3830 10.3000 0.3790

11 7 C4 CA M 2.2100 0.3850 11.6470 0.3290

12 11 N3 NH B 3.3120 0.6320 12.4150 -0.8750

13 12 H3 H E 3.8480 -0.0530 12.6600 0.3820

14 12 H4 H E 3.2080 1.2330 13.0330 0.3820

15 11 C5 CA M 1.1480 1.2760 11.6080 -0.2930

16 15 H6 HA E 1.5060 2.0730 12.1400 0.1870

17 15 C6 CA M 0.0340 1.0470 10.8260 -0.1820

18 17 H7 HA E -0.6940 1.6730 10.8100 0.2010

BEGVOZh

1 0 O1 OS M -1.1100 1.1860 -2.0620 -0.3650

2 1 C1 CT M -1.0500 2.5400 -2.4690 0.0290

3 2 H1 H1 E -0.3690 2.9930 -1.9480 0.0900

4 2 H2 H1 E -1.9000 2.9630 -2.2730 0.0900

5 2 C2 CT M -0.7440 2.7230 -3.9320 -0.0520

6 5 H3 HP E -1.5090 2.4390 -4.4570 0.1200

7 5 H4 HP E -0.5920 3.6640 -4.1130 0.1200

8 5 N1 N3 M 0.4540 1.9440 -4.3470 0.0030

9 8 H5 H E 1.0950 1.9920 -3.7100 0.2340

10 8 H6 H E 0.1800 1.0890 -4.4620 0.2340

11 8 C3 CT M 1.0420 2.4240 -5.6240 -0.0520

12 11 H7 HP E 1.3610 3.3330 -5.5080 0.1200

13 11 H8 HP E 0.3560 2.4330 -6.3100 0.1200

14 11 C4 CT M 2.1850 1.5510 -6.0760 0.0290

15 14 H9 H1 E 1.9050 0.6220 -6.0960 0.0900

16 14 H10 H1 E 2.4640 1.8060 -6.9690 0.0900

17 14 O2 OS M 3.2690 1.7170 -5.1580 -0.3650

18 17 C5 CT M 4.3670 0.8130 -5.4080 -0.0160

19 18 H11 H1 E 5.1980 1.2570 -5.1780 0.1240

20 18 H12 H1 E 4.3950 0.6090 -6.3550 0.1240

21 18 C7 CT M 4.2720 -0.4630 -4.6400 -0.0160

22 21 H27 H1 E 4.1850 -0.2740 -3.6920 0.1240

23 21 H28 H1 E 5.0740 -0.9920 -4.7750 0.1240

24 21 O3 OS M 3.1260 -1.1860 -5.1010 -0.3650

25 24 C8 CT M 3.0660 -2.5400 -4.6940 0.0290

26 25 H15 H1 E 2.3850 -2.9930 -5.2140 0.0900

27 25 H16 H1 E 3.9160 -2.9630 -4.8900 0.0900

28 25 C9 CT M 2.7600 -2.7230 -3.2300 -0.0520

29 28 H17 HP E 2.6080 -3.6640 -3.0500 0.1200

30 28 H18 HP E 3.5250 -2.4390 -2.7050 0.1200

31 28 N2 N3 M 1.5630 -1.9440 -2.8160 0.0030

32 31 H19 H E 0.9210 -1.9920 -3.4520 0.2340

33 31 H20 H E 1.8360 -1.0890 -2.7000 0.2340

34 31 C10 CT M 0.9740 -2.4240 -1.5390 -0.0520

35 34 H21 HP E 0.6550 -3.3330 -1.6550 0.1200

36 34 H22 HP E 1.6600 -2.4330 -0.8530 0.1200

37 34 C11 CT M -0.1690 -1.5510 -1.0870 0.0290

38 37 H23 H1 E 0.1110 -0.6220 -1.0670 0.0900

39 37 H24 H1 E -0.4480 -1.8060 -0.1930 0.0900

40 37 O4 OS M -1.2530 -1.7170 -2.0050 -0.3650

41 40 C12 CT M -2.3510 -0.8130 -1.7550 -0.0160

42 41 H25 H1 E -3.1820 -1.2570 -1.9850 0.1240

43 41 H26 H1 E -2.3790 -0.6090 -0.8070 0.1240

44 41 C6 CT M -2.2560 0.4630 -2.5230 -0.0160

45 44 H13 H1 E -2.1690 0.2740 -3.4700 0.1240

46 44 H14 H1 E -3.0580 0.9920 -2.3880 0.1240

BEGVOZg

1 0 O4 O M 2.6170 0.4090 -1.8820 -0.3720

2 1 N4 N2 M 2.3570 1.6320 -2.1960 -0.1970

3 2 C1 CM M 3.1190 2.5900 -1.7030 0.3270

4 3 C2 C M 2.7030 3.9340 -2.0880 0.2070

5 4 O1 O E 1.7930 4.1940 -2.8640 -0.5870

6 4 N1 N M 3.3990 4.9750 -1.5010 -0.2440

7 6 H2 H E 3.1080 5.7610 -1.6040 0.2960

8 6 C3 C M 4.4490 4.8700 -0.6240 0.3450

9 8 O2 O E 4.9800 5.8410 -0.1330 -0.5840

10 8 N2 N M 4.8560 3.5980 -0.3600 -0.1670

11 10 H3 H E 5.4980 3.5110 0.1580 0.2790

12 10 C4 CM M 4.2810 2.4340 -0.8300 0.1940

13 12 N3 N2 M 4.7350 1.2830 -0.4940 -0.3770

14 13 O3 OH M 5.8370 1.4180 0.3800 -0.5620

15 14 H1 HO E 5.9120 0.4970 0.6370 0.4420

BEVHERh

1 0 C20 CT M 3.5600 -6.1570 13.7130 0.0790

2 1 C18 CA S 4.6550 -5.1560 14.0030 0.0030

3 2 C4 CA S 5.0080 -4.2450 13.0250 -0.2310

4 3 H1 HA E 4.5250 -4.2500 12.1950 0.1610

5 1 H7 H1 E 3.7440 -6.9530 14.2160 0.0800

6 1 H8 H1 E 3.5710 -6.3610 12.7750 0.0800

7 1 O6 OS M 2.2250 -5.6910 14.0720 -0.3690

8 7 C25 CA M 2.0070 -5.1140 15.2760 0.2480

9 8 C14 CA M 0.9670 -4.1940 15.3290 -0.1520

10 9 H4 HA E 0.4330 -4.0290 14.5480 0.1480

11 9 C13 CA M 0.6970 -3.5170 16.4960 -0.2270

12 11 C17 C B -0.3040 -2.3990 16.4810 0.8900

13 12 O7 O E -0.0900 -1.3060 16.9130 -0.5780

14 12 O8 OS S -1.4420 -2.7600 15.9110 -0.4120

15 14 C34 CT 3 -2.4330 -1.6940 15.7410 -0.0160

16 15 H24 H1 E -3.2230 -2.0460 15.3220 0.0820

17 15 H25 H1 E -2.6610 -1.3340 16.6010 0.0820

18 15 H26 H1 E -2.0620 -1.0000 15.1910 0.0820

19 11 C15 CA M 1.4630 -3.7640 17.6170 -0.2270

20 19 C7 C B 1.2620 -2.9830 18.8540 0.8900

21 20 O3 O E 2.1370 -2.3880 19.4090 -0.5780

22 20 O4 OS S -0.0100 -3.0380 19.2570 -0.4120

23 22 C33 CT 3 -0.3460 -2.1730 20.3530 -0.0160

24 23 H21 H1 E -1.2740 -2.2810 20.5770 0.0820

25 23 H22 H1 E 0.1940 -2.3990 21.1140 0.0820

26 23 H23 H1 E -0.1820 -1.2610 20.1030 0.0820

27 19 C12 CA M 2.4670 -4.7160 17.5720 -0.1520

28 27 H3 HA E 2.9850 -4.8970 18.3600 0.1480

29 27 C26 CA M 2.7310 -5.4050 16.4130 0.2480

30 29 O5 OS M 3.6470 -6.4450 16.4040 -0.3690

31 30 C29 CT M 5.0370 -6.0360 16.3370 0.0790

32 31 H16 H1 E 5.2640 -5.5990 17.1610 0.0800

33 31 H17 H1 E 5.5810 -6.8200 16.2310 0.0800

34 31 C19 CA M 5.3300 -5.0980 15.2060 0.0030

35 34 C8 CA M 6.3400 -4.1650 15.3820 -0.2310

36 35 H2 HA E 6.7960 -4.1340 16.2260 0.1610

37 35 C10 CA M 6.7240 -3.2750 14.3910 0.0470

38 37 C21 CT 3 7.8580 -2.3140 14.6760 0.0620

39 38 O2 OS E 7.3960 -1.0300 15.1620 -0.2800

40 38 H9 H1 E 8.3520 -2.1750 13.8650 0.0610

41 38 H10 H1 E 8.4330 -2.7050 15.3380 0.0610

42 37 C6 CA M 6.0490 -3.3150 13.1920 0.0470

43 42 C16 CT M 6.3750 -2.4530 12.0190 0.0620

44 43 H5 H1 E 5.5540 -2.2100 11.5850 0.0610

45 43 H6 H1 E 6.9180 -2.9650 11.4150 0.0610

46 43 O1 OS M 7.0790 -1.2390 12.3430 -0.2800

47 46 C1 CA M 6.3720 -0.3250 13.1050 0.0960

48 47 C2 CA M 6.6040 -0.2240 14.4170 0.0960

49 48 C5 CA M 6.0110 0.8560 15.1650 0.0390

50 49 C23 CA B 6.3330 1.0220 16.5110 -0.1380

51 50 C24 CA B 5.7710 2.0460 17.2210 -0.1380

52 51 C22 CA B 4.8850 2.9270 16.6250 -0.1680

53 52 C28 CA S 4.5610 2.7960 15.3150 -0.1500

54 53 H15 HA E 3.9470 3.4100 14.9040 0.1360

55 52 H11 HA E 4.4990 3.6380 17.1420 0.1440

56 51 H13 HA E 5.9880 2.1570 18.1500 0.1400

57 50 H12 HA E 6.9500 0.4210 16.9330 0.1220

58 49 C11 CA M 5.1230 1.7570 14.5470 0.0140

59 58 C9 CA M 4.8270 1.5940 13.1530 0.0140

60 59 C3 CA M 5.4840 0.5790 12.4370 0.0390

61 60 C27 CA M 5.2780 0.4720 11.0630 -0.1380

62 61 H14 HA E 5.7510 -0.1960 10.5630 0.1220

63 61 C30 CA M 4.4080 1.3160 10.4330 -0.1380

64 63 H18 HA E 4.2600 1.2240 9.4890 0.1400

65 63 C31 CA M 3.7370 2.3080 11.1310 -0.1680

66 65 H19 HA E 3.1260 2.8910 10.6740 0.1440

67 65 C32 CA M 3.9410 2.4480 12.4650 -0.1500

68 67 H20 HA E 3.4790 3.1340 12.9530 0.1360

BEVHERg

1 0 N1 N1 M 5.3200 -2.7750 18.1460 -0.4000

2 1 C8 CZ M 4.6320 -2.1290 17.5140 0.3600

3 2 C4 CA M 3.7670 -1.3470 16.6860 0.0110

4 3 C2 CA M 3.5890 -1.7270 15.3780 -0.1400

5 4 H1 HA E 4.0190 -2.5130 15.0340 0.2000

6 4 C1 CA M 2.7840 -0.9540 14.5680 0.0110

7 6 C9 CZ S 2.6240 -1.2900 13.1900 0.3600

8 7 N2 N1 E 2.5040 -1.5060 12.0960 -0.4000

9 6 C6 CA M 2.1400 0.1890 15.0670 0.0110

10 9 C10 CZ S 1.3120 0.9910 14.2250 0.3600

11 10 N4 N1 E 0.6660 1.6360 13.5590 -0.4000

12 9 C3 CA M 2.3180 0.5640 16.3730 -0.1400

13 12 H2 HA E 1.8780 1.3430 16.7220 0.2000

14 12 C5 CA M 3.1390 -0.1990 17.1760 0.0110

15 14 C7 CZ M 3.3490 0.2360 18.5120 0.3600

16 15 N3 N1 M 3.5230 0.6340 19.5480 -0.4000

BEVWAAh

1 0 O1 OS M 6.4120 6.0840 -0.4090 -0.2770

2 1 C1 CT M 5.0170 5.5400 -0.9200 -0.0070

3 2 H4 H1 E 4.6780 6.4880 -1.1770 0.0910

4 2 H5 H1 E 5.6430 4.9010 -1.7890 0.0910

5 2 C2 CA M 4.3630 4.8000 0.1000 0.0660

6 5 C3 CA M 3.4170 5.4710 0.8620 -0.2530

7 6 H6 HA E 3.2540 6.5970 0.7200 0.1620

8 6 C4 CA M 2.5700 4.8060 1.7490 0.0160

9 8 C5 CT M 1.6100 5.6280 2.5380 0.0740

10 9 H7 H1 E 0.6350 5.8220 1.9330 0.0780

11 9 H8 H1 E 2.1060 6.8320 2.5590 0.0780

12 9 O2 OS M 1.2440 5.0100 3.8210 -0.3690

13 12 C6 CA M 2.2850 4.6610 4.6550 0.2310

14 13 C7 CA M 2.8630 5.6200 5.4830 -0.1880

15 14 H9 HA E 2.4910 6.7560 5.1730 0.1370

16 14 C8 CA M 3.7660 5.2040 6.4900 -0.2110

17 16 C31 C B 4.3620 6.1750 7.4460 0.8030

18 17 O5 O E 5.1400 5.8670 8.3050 -0.5880

19 17 O6 OS S 3.9300 7.4320 7.2720 -0.3380

20 19 C32 CT 3 4.4360 8.4600 8.1710 -0.0860

21 20 H1 H1 E 3.9160 9.3260 7.7630 0.0960

22 20 H2 H1 E 4.2570 8.1760 9.2220 0.0960

23 20 H3 H1 E 5.5870 8.7290 7.6170 0.0960

24 16 C9 CA M 4.0980 3.8590 6.6380 -0.0930

25 24 H10 HA E 4.8290 3.6400 7.4930 0.1550

26 24 C10 CA M 3.5750 2.9110 5.7550 -0.2380

27 26 H11 HA E 3.7230 1.9050 5.8540 0.1570

28 26 C11 CA M 2.6600 3.3080 4.7670 0.2630

29 28 O3 OS M 2.1690 2.3030 3.9970 -0.3500

30 29 C12 CT M 1.6790 2.5840 2.6410 0.0470

31 30 H12 H1 E 1.6500 1.6010 2.0820 0.0900

32 30 H13 H1 E 0.5360 3.0760 2.6900 0.0900

33 30 C13 CA M 2.6580 3.4010 1.8340 0.0210

34 33 C14 CA M 3.6700 2.7330 1.1540 -0.2410

35 34 H14 HA E 3.8690 1.5910 1.3110 0.1660

36 34 C15 CA M 4.5200 3.4090 0.2810 0.0140

37 36 C16 CT M 5.5450 2.5830 -0.4550 0.0860

38 37 H15 H1 E 5.2060 2.3450 -1.4600 0.0700

39 37 H16 H1 E 5.7750 1.6000 0.3140 0.0700

40 37 O4 OS M 6.7680 3.2660 -0.7660 -0.3060

41 40 C17 CA M 7.4120 3.9590 0.2470 0.0310

42 41 C18 CA M 8.3510 3.2660 1.0810 0.0350

43 42 C19 CA M 8.5050 1.8470 0.9780 -0.1210

44 43 H17 HA E 7.8730 1.2070 0.4980 0.1150

45 43 C20 CA M 9.4300 1.1790 1.7490 -0.1540

46 45 H18 HA E 9.4020 -0.2360 1.5680 0.1420

47 45 C21 CA M 10.2220 1.8630 2.6170 -0.1540

48 47 H19 HA E 11.0640 1.3050 3.4610 0.1410

49 47 C22 CA M 10.1300 3.2600 2.7430 -0.1710

50 49 H20 HA E 10.9120 3.9590 3.6350 0.1450

51 49 C23 CA M 9.1970 3.9840 1.9750 0.0210

52 51 C24 CA M 9.0510 5.4230 2.0390 0.0160

53 52 C25 CA M 9.8570 6.2230 2.8960 -0.1530

54 53 H21 HA E 10.5960 5.7180 3.7630 0.1350

55 53 C26 CA M 9.7120 7.5880 2.9530 -0.1550

56 55 H22 HA E 10.2870 8.1340 3.8670 0.1440

57 55 C27 CA M 8.7150 8.2240 2.1570 -0.1500

58 57 H23 HA E 8.5730 9.3170 2.1100 0.1450

59 57 C28 CA M 7.9330 7.4850 1.3280 -0.1340

60 59 H24 HA E 7.2780 7.7920 0.6460 0.1220

61 59 C29 CA M 8.0770 6.0740 1.2500 0.0170

62 61 C30 CA M 7.2620 5.3120 0.3550 0.1830

BEVWAAg

1 0 N1 N1 M 9.6730 1.2840 6.5970 -0.4300

2 1 C1 CZ M 8.8210 1.5420 5.8850 0.4130

3 2 C2 CM M 7.7130 1.9090 5.0080 -0.2120

4 3 C3 CZ S 7.0090 0.8050 4.4050 0.4130

5 4 N2 N1 E 6.4280 -0.0450 4.0020 -0.4300

6 3 C4 CA M 7.3560 3.2390 4.8020 0.1940

7 6 C9 CA B 8.0430 4.2670 5.5400 -0.1250

8 7 C8 CA S 7.6810 5.5550 5.4030 -0.1250

9 8 H3 HA E 8.0400 6.4580 5.9940 0.1510

10 7 H4 HA E 9.0240 4.0890 6.3460 0.1510

11 6 C5 CA M 6.3070 3.6040 3.9080 -0.1250

12 11 H1 HA E 5.6910 2.8410 3.4060 0.1510

13 11 C6 CA M 5.9590 4.9090 3.7330 -0.1250

14 13 H2 HA E 5.1630 5.1640 3.1070 0.1510

15 13 C7 CA M 6.6170 5.9320 4.5030 0.1940

16 15 C11 CM M 6.2480 7.2570 4.3940 -0.2120

17 16 C12 CZ S 5.2460 7.6710 3.4830 0.4130

18 17 N4 N1 E 4.4470 8.0120 2.7240 -0.4300

19 16 C10 CZ M 6.8970 8.2920 5.2020 0.4130

20 19 N3 N1 M 7.4170 9.0480 5.8420 -0.4300

BIFKIKh

1 0 C1 CA M 3.2060 2.5850 5.6860 0.2250

2 1 O1 OS M 4.4840 2.5610 6.1720 -0.3460

3 2 C2 CT M 5.0130 1.3290 6.6660 0.0570

4 3 H1 H1 E 4.3880 0.8910 7.1130 0.0450

5 3 H2 H1 E 5.2420 0.7270 5.9070 0.0450

6 3 C3 CT M 6.1390 1.6290 7.5720 0.2400

7 6 H3 H1 E 5.8590 2.3360 8.1760 0.0160

8 6 H4 H1 E 6.2910 0.9100 8.2210 0.0160

9 6 O2 OS M 7.3370 2.0460 6.9230 -0.4190

10 9 C4 CT M 8.2320 0.9830 6.6330 0.2750

11 10 H5 H1 E 7.9050 0.4280 5.9250 -0.0030

12 10 H6 H1 E 8.3580 0.4280 7.4590 -0.0030

13 10 C5 CT M 9.5290 1.5450 6.2220 0.0460

14 13 H7 H1 E 9.4230 2.1800 5.4750 0.0280

15 13 H8 H1 E 10.1630 0.8470 5.7780 0.0280

16 13 O3 OS M 10.1640 2.1750 7.3030 -0.3190

17 16 C6 CT M 11.2890 2.9030 6.8780 0.0230

18 17 H9 H1 E 10.9830 3.5950 6.3390 0.0600

19 17 H10 H1 E 11.8870 2.2830 6.4340 0.0600

20 17 C7 CT M 11.9690 3.5330 8.0220 0.1620

21 20 H11 H1 E 12.7650 4.0870 7.6820 0.0330

22 20 H12 H1 E 12.2090 2.9110 8.7250 0.0330

23 20 O4 OS M 11.0990 4.5180 8.6050 -0.4200

24 23 C8 CT M 11.7630 5.3160 9.5660 0.1790

25 24 H13 H1 E 12.1150 4.7250 10.2180 0.0090

26 24 H14 H1 E 12.5390 5.8350 9.1360 0.0090

27 24 C9 CT M 10.7800 6.1630 10.2790 0.0830

28 27 H15 H1 E 10.0550 5.6330 10.6410 0.0610

29 27 H16 H1 E 11.2440 6.7260 11.0390 0.0610

30 27 O5 OS M 10.1500 7.0950 9.4280 -0.3910

31 30 C10 CT M 10.8030 8.3420 9.3690 0.0830

32 31 H17 H1 E 11.7740 8.2610 9.1570 0.0610

33 31 H18 H1 E 10.6620 8.7460 10.2840 0.0610

34 31 C11 CT M 10.1300 9.2180 8.3850 0.1790

35 34 H19 H1 E 10.1040 8.7510 7.6020 0.0090

36 34 H20 H1 E 10.5880 10.0780 8.2670 0.0090

37 34 O6 OS M 8.7890 9.4600 8.7710 -0.4200

38 37 C12 CT M 8.0770 10.2430 7.8550 0.1620

39 38 H21 H1 E 8.2650 9.7650 6.9120 0.0330

40 38 H22 H1 E 8.4470 11.0430 7.7560 0.0330

41 38 C13 CT M 6.6710 10.3790 8.2300 0.0230

42 41 H23 H1 E 6.5150 10.7860 9.1100 0.0600

43 41 H24 H1 E 6.1910 11.0540 7.6120 0.0600

44 41 O7 OS M 6.0340 9.1240 8.1940 -0.3190

45 44 C14 CT M 4.6160 9.1710 8.1030 0.0460

46 45 H25 H1 E 4.3960 9.6400 7.3190 0.0280

47 45 H26 H1 E 4.2640 9.7520 8.8250 0.0280

48 45 C15 CT M 4.0840 7.7880 8.1020 0.2750

49 48 H27 H1 E 3.1490 7.7820 8.0490 -0.0030

50 48 H28 H1 E 4.3770 7.3430 8.9550 -0.0030

51 48 O8 OS M 4.6700 6.9770 7.0890 -0.4190

52 51 C16 CT M 4.1880 7.1440 5.7400 0.2400

53 52 H29 H1 E 3.8900 8.0560 5.6490 0.0160

54 52 H30 H1 E 5.0210 6.9920 5.1330 0.0160

55 52 C17 CT M 3.0760 6.2020 5.4580 0.0570

56 55 H31 H1 E 2.7760 6.4000 4.5640 0.0450

57 55 H32 H1 E 2.2900 6.3660 6.1470 0.0450

58 55 O9 OS M 3.6060 4.8830 5.5430 -0.3460

59 58 C18 CA M 2.7180 3.8560 5.3580 0.2250

60 59 C19 CA M 1.4290 3.9920 4.9110 -0.1960

61 60 H33 HA E 1.0970 4.8370 4.8280 0.1460

62 60 C20 CA M 0.6160 2.8790 4.8020 -0.2000

63 62 H34 HA E -0.2190 2.9960 4.5590 0.1630

64 62 C21 CA M 1.0840 1.6550 5.1140 -0.2000

65 64 H35 HA E 0.5620 0.8360 5.0480 0.1630

66 64 C22 CA M 2.3750 1.4890 5.5630 -0.1960

67 66 H36 HA E 2.8000 0.6240 5.7320 0.1460

BIFKIKg

1 0 O1 OH M 6.2600 5.0310 7.4240 -0.4690

2 1 H1 HO E 5.6970 5.6980 7.3900 0.4620

3 1 C1 CM M 7.4140 5.4300 7.8670 0.8620

4 3 N2 NT B 7.6290 6.6710 8.1820 -0.8710

5 4 H4 H E 8.2890 6.9120 8.4850 0.4710

6 4 H5 H E 6.9770 7.3110 8.1710 0.4710

7 3 N1 NT M 8.3180 4.5020 7.9810 -0.8710

8 7 H2 H E 9.1350 4.6770 8.2080 0.4710

9 7 H3 H E 8.1830 3.7320 7.7190 0.4710

BOHWUQh

1 0 C9 CT M -3.9760 7.9670 4.2530 0.0030

2 1 H13 H1 E -4.8600 8.4550 3.8420 0.0550

3 1 H64 H1 E -4.2480 7.4260 5.1590 0.0550

4 1 H82 H1 E -3.5740 7.2680 3.5190 0.0550

5 1 O5 OS M -3.1510 8.7920 4.5130 -0.3000

6 5 C6 CT M -2.6420 9.1440 5.7250 -0.0170

7 6 H6 H1 E -3.1790 10.0090 6.1150 0.0860

8 6 H7 H1 E -2.7490 8.3220 6.4320 0.0860

9 6 C5 CT M -1.0910 9.5090 5.5280 0.0200

10 9 O4 OS E -1.1210 10.8270 4.8650 -0.3150

11 9 H5 H1 E -0.5760 8.7320 4.9620 0.1480

12 9 C4 CT M -0.3380 9.6210 6.8420 -0.0070

13 12 O3 OS E -0.0480 8.2980 7.2990 -0.2360

14 12 H4 H1 E -0.9620 10.1990 7.5240 0.1440

15 12 C3 CT M 1.0310 10.3100 6.6800 -0.0350

16 15 O2 OS S 1.5180 10.6170 8.0420 -0.3450

17 16 C8 CT 3 2.9470 10.4960 8.2530 -0.0150

18 17 H12 H1 E 3.4310 11.4420 8.0090 0.0650

19 17 H63 H1 E 3.3440 9.7090 7.6120 0.0650

20 17 H81 H1 E 3.1410 10.2480 9.2970 0.0650

21 15 H3 H1 E 1.7330 9.6780 6.1370 0.1660

22 15 C2 CT M 0.8280 11.6490 5.9120 0.0310

23 22 O1 OS S 2.1400 12.2310 5.6550 -0.2340

24 23 C7 CT 3 2.1940 13.6030 5.7700 -0.1270

25 24 H11 H1 E 2.3230 13.8760 6.8170 0.0890

26 24 H62 H1 E 1.2680 14.0370 5.3920 0.0890

27 24 H80 H1 E 3.0350 13.9830 5.1900 0.0890

28 22 H2 H1 E 0.2080 12.3100 6.5170 0.1470

29 22 C1 CT M 0.1100 11.4130 4.5350 0.0060

30 29 H1 H2 E 0.0040 12.3270 3.9500 0.1690

31 29 O8 OS M 0.8970 10.4030 3.7870 -0.2360

32 31 C13 CT M 0.9840 10.6290 2.3550 -0.0070

33 32 C14 CT 3 0.0100 9.6690 1.6180 0.0200

34 33 O9 OS E 0.3090 9.6420 0.2420 -0.3150

35 33 C15 CT 3 -1.4330 10.3000 1.6530 -0.0170

36 35 O10 OS S -2.2590 9.3160 1.0960 -0.3000

37 36 C18 CT 3 -3.6570 9.8040 1.0380 0.0030

38 37 H16 H1 E -4.0080 9.7810 0.0060 0.0550

39 37 H67 H1 E -4.2910 9.1630 1.6510 0.0550

40 37 H85 H1 E -3.7020 10.8260 1.4150 0.0550

41 35 H33 H1 E -1.7400 10.5770 2.6620 0.0860

42 35 H34 H1 E -1.4690 11.2150 1.0610 0.0860

43 33 H32 H1 E 0.0850 8.6890 2.0890 0.1480

44 32 H31 H1 E 0.7170 11.6370 2.0370 0.1440

45 32 C12 CT M 2.4450 10.2720 2.0580 -0.0350

46 45 O7 OS S 3.2580 11.3940 2.4790 -0.3450

47 46 C17 CT 3 4.3370 10.9440 3.4380 -0.0150

48 47 H15 H1 E 4.6600 9.9360 3.1760 0.0650

49 47 H66 H1 E 3.9450 10.9470 4.4550 0.0650

50 47 H84 H1 E 5.1860 11.6250 3.3740 0.0650

51 45 H30 H1 E 2.7220 9.3310 2.5330 0.1660

52 45 C11 CT M 2.5970 10.2810 0.4340 0.0310

53 52 O6 OS S 3.9450 9.9310 0.1330 -0.2340

54 53 C16 CT 3 4.5980 10.7650 -0.7460 -0.1270

55 54 H14 H1 E 4.6890 11.7580 -0.3060 0.0890

56 54 H65 H1 E 4.0320 10.8290 -1.6750 0.0890

57 54 H83 H1 E 5.5920 10.3670 -0.9530 0.0890

58 52 H9 H1 E 2.3580 11.2520 0.0000 0.1470

59 52 C10 CT M 1.6260 9.1900 -0.1310 0.0060

60 59 H8 H2 E 1.7400 9.0560 -1.2070 0.1690

61 59 O13 OS M 1.8840 7.9360 0.5050 -0.2360

62 61 C22 CT M 2.0020 6.8020 -0.3590 -0.0070

63 62 C23 CT 3 0.6620 5.9910 -0.3510 0.0200

64 63 O14 OS E 0.8860 4.7930 -1.1520 -0.3150

65 63 C24 CT 3 -0.3810 6.8140 -1.1650 -0.0170

66 65 O15 OS S -1.6580 6.0460 -1.1060 -0.3000

67 66 C27 CT 3 -2.7120 6.6010 -0.6590 0.0030

68 67 H19 H1 E -2.5380 6.9450 0.3600 0.0550

69 67 H70 H1 E -2.9720 7.4510 -1.2910 0.0550

70 67 H88 H1 E -3.5310 5.8820 -0.6640 0.0550

71 65 H40 H1 E -0.5020 7.8160 -0.7540 0.0860

72 65 H41 H1 E -0.0530 6.9230 -2.1990 0.0860

73 63 H39 H1 E 0.3400 5.7780 0.6690 0.1480

74 62 H38 H1 E 2.2400 7.1150 -1.3760 0.1440

75 62 C21 CT M 3.0840 5.8940 0.2690 -0.0350

76 75 O12 OS S 4.3490 6.5800 0.0770 -0.3450

77 76 C26 CT 3 5.0100 6.9110 1.3780 -0.0150

78 77 H18 H1 E 5.2840 5.9900 1.8920 0.0650

79 77 H69 H1 E 4.3220 7.4840 2.0000 0.0650

80 77 H87 H1 E 5.9060 7.5020 1.1890 0.0650

81 75 H37 H1 E 2.8420 5.6940 1.3130 0.1660

82 75 C20 CT M 3.1910 4.6000 -0.4940 0.0310

83 82 O11 OS S 4.0730 3.7300 0.1400 -0.2340

84 83 C25 CT 3 5.3880 3.5720 -0.5420 -0.1270

85 84 H17 H1 E 6.1510 3.3230 0.1950 0.0890

86 84 H68 H1 E 5.6540 4.5050 -1.0400 0.0890

87 84 H86 H1 E 5.3220 2.7720 -1.2790 0.0890

88 82 H36 H1 E 3.5350 4.8800 -1.4900 0.1470

89 82 C19 CT M 1.8290 3.8420 -0.6010 0.0060

90 89 H35 H2 E 1.9160 2.9480 -1.2180 0.1690

91 89 O18 OS M 1.3940 3.4560 0.6990 -0.2360

92 91 C31 CT M 0.6930 2.1690 0.8060 -0.0070

93 92 C32 CT 3 -0.7200 2.5050 1.3880 0.0200

94 93 O19 OS E -1.3150 1.3080 1.8540 -0.3150

95 93 C33 CT 3 -1.6410 3.0170 0.2760 -0.0170

96 95 O20 OS S -1.7250 2.0500 -0.7850 -0.3000

97 96 C36 CT 3 -2.6260 2.5620 -1.9090 0.0030

98 97 H22 H1 E -2.9210 1.7290 -2.5470 0.0550

99 97 H73 H1 E -2.0850 3.3000 -2.5020 0.0550

100 97 H91 H1 E -3.5150 3.0230 -1.4790 0.0550

101 95 H47 H1 E -1.2440 3.9520 -0.1190 0.0860

102 95 H48 H1 E -2.6370 3.1800 0.6870 0.0860

103 93 H46 H1 E -0.5990 3.2490 2.1750 0.1480

104 92 H45 H1 E 0.5640 1.6340 -0.1350 0.1440

105 92 C30 CT M 1.5340 1.2850 1.7300 -0.0350

106 105 O17 OS S 2.6380 0.8460 0.9430 -0.3450

107 106 C35 CT 3 3.9220 0.8650 1.6400 -0.0150

108 107 H21 H1 E 4.3000 -0.1530 1.7340 0.0650

109 107 H72 H1 E 3.7910 1.2980 2.6320 0.0650

110 107 H90 H1 E 4.6340 1.4650 1.0730 0.0650

111 105 H44 H1 E 1.8590 1.8310 2.6150 0.1660

112 105 C29 CT M 0.7520 0.1310 2.2060 0.0310

113 112 O16 OS S 1.5340 -0.6240 3.1300 -0.2340

114 113 C34 CT 3 1.1670 -2.0020 3.3140 -0.1270

115 114 H20 H1 E 1.9210 -2.6430 2.8570 0.0890

116 114 H71 H1 E 1.0990 -2.2210 4.3800 0.0890

117 114 H89 H1 E 0.2010 -2.1860 2.8440 0.0890

118 112 H43 H1 E 0.4910 -0.5040 1.3590 0.1470

119 112 C28 CT M -0.5620 0.6170 2.8770 0.0060

120 119 H42 H2 E -1.1410 -0.2070 3.2950 0.1690

121 119 O23 OS M -0.2230 1.5230 3.9190 -0.2360

122 121 C40 CT M -1.0300 1.4750 5.1920 -0.0070

123 122 C41 CT 3 -1.8250 2.7790 5.2550 0.0200

124 123 O24 OS E -2.4370 2.7860 6.5440 -0.3150

125 123 C42 CT 3 -3.0190 2.9740 4.2320 -0.0170

126 125 O25 OS S -3.7280 1.7750 4.3430 -0.3000

127 126 C45 CT 3 -4.8250 1.6830 3.2880 0.0030

128 127 H27 H1 E -4.4370 2.0260 2.3290 0.0550

129 127 H76 H1 E -5.6660 2.3100 3.5840 0.0550

130 127 H94 H1 E -5.1570 0.6490 3.1980 0.0550

131 125 H24 H1 E -3.6270 3.8530 4.4450 0.0860

132 125 H53 H1 E -2.6470 3.1170 3.2180 0.0860

133 123 H52 H1 E -1.1070 3.5650 5.0200 0.1480

134 122 H51 H1 E -1.7340 0.6470 5.2790 0.1440

135 122 C39 CT M 0.0110 1.4060 6.2570 -0.0350

136 135 O22 OS S 0.5960 0.1380 6.2500 -0.3450

137 136 C44 CT 3 2.1580 0.1310 6.4090 -0.0150

138 137 H26 H1 E 2.5770 1.0040 5.9090 0.0650

139 137 H75 H1 E 2.5650 -0.7750 5.9600 0.0650

140 137 H93 H1 E 2.4160 0.1600 7.4680 0.0650

141 135 H50 H1 E 0.7560 2.1820 6.0830 0.1660

142 135 C38 CT M -0.6890 1.5730 7.6530 0.0310

143 142 O21 OS S 0.3250 1.6560 8.6240 -0.2340

144 143 C43 CT 3 0.0020 1.0750 9.8750 -0.1270

145 144 H25 H1 E 0.6700 0.2360 10.0700 0.0890

146 144 H74 H1 E 0.1150 1.8200 10.6620 0.0890

147 144 H92 H1 E -1.0290 0.7220 9.8550 0.0890

148 142 H23 H1 E -1.3560 0.7380 7.8670 0.1470

149 142 C37 CT M -1.5090 2.9050 7.6410 0.0060

150 149 H49 H2 E -2.0100 3.0550 8.5980 0.1690

151 149 O28 OS M -0.6770 4.0200 7.4320 -0.2360

152 151 C49 CT M -0.7920 5.1020 8.3630 -0.0070

153 152 H57 H1 E -1.1500 4.8270 9.3550 0.1440

154 152 C48 CT M 0.6300 5.6670 8.4040 -0.0350

155 154 O27 OS S 1.5580 4.7710 9.1780 -0.3450

156 155 C53 CT 3 2.5350 4.2090 8.4020 -0.0150

157 156 H29 H1 E 2.7390 4.8560 7.5490 0.0650

158 156 H78 H1 E 2.2020 3.2330 8.0480 0.0650

159 156 H96 H1 E 3.4430 4.0900 8.9920 0.0650

160 154 H56 H1 E 0.9710 5.7920 7.3760 0.1660

161 154 C47 CT M 0.5830 6.9970 9.2630 0.0310

162 161 O26 OS S 1.9040 7.5810 9.2110 -0.2340

163 162 C52 CT 3 2.5800 7.6620 10.3610 -0.1270

164 163 H28 H1 E 2.8650 6.6620 10.6870 0.0890

165 163 H77 H1 E 1.9510 8.1270 11.1210 0.0890

166 163 H95 H1 E 3.4760 8.2650 10.2130 0.0890

167 161 H55 H1 E 0.2840 6.7980 10.2920 0.1470

168 161 C46 CT M -0.3900 7.9340 8.6350 0.0060

169 168 H54 H2 E -0.3710 8.8450 9.2330 0.1690

170 168 O29 OS M -1.7070 7.3330 8.6310 -0.3150

171 170 C50 CT M -1.8000 6.1300 7.7860 0.0200

172 171 H58 H1 E -1.6600 6.3270 6.7230 0.1480

173 171 C51 CT M -3.3020 5.4720 8.2860 -0.0170

174 173 H59 H1 E -3.3960 5.4060 9.3700 0.0860

175 173 H60 H1 E -3.5250 4.4480 7.9860 0.0860

176 173 O30 OS M -4.0540 6.4470 7.7570 -0.3000

177 176 C54 CT M -5.4640 5.6580 8.2190 0.0030

178 177 H10 H1 E -6.2670 5.9150 7.5280 0.0550

179 177 H61 H1 E -5.7410 5.9680 9.2270 0.0550

180 177 H79 H1 E -5.3010 4.5800 8.2010 0.0550

BOHWUQg

1 0 O1 O M -0.4280 5.5220 3.3910 -0.4560

2 1 C4 C M 0.4160 6.1060 3.8760 0.4220

3 2 H3 HA E 0.2110 7.1650 4.0310 -0.0050

4 2 C1 CA M 1.5420 5.7270 4.2600 -0.0150

5 4 C2 CA M 2.3930 6.6990 4.7210 -0.1100

6 5 H1 HA E 2.0280 7.7070 4.9180 0.1320

7 5 C5 CA M 3.6390 6.3990 4.9170 -0.1490

8 7 H4 HA E 4.2750 7.2800 4.8250 0.1370

9 7 C7 CA M 4.2980 5.4070 5.1750 -0.1010

10 9 H6 HA E 5.2210 5.3560 5.7520 0.1340

11 9 C6 CA M 3.6600 4.4160 4.6110 -0.1490

12 11 H5 HA E 4.2010 3.4870 4.4290 0.1370

13 11 C3 CA M 2.3380 4.4880 4.2360 -0.1100

14 13 H2 HA E 1.8500 3.5730 3.9010 0.1320

CACQEDh

1 0 O5 OH M 0.9640 -6.4620 1.9410 -0.6510

2 1 H13 HO E 1.2470 -7.3740 2.0440 0.4170

3 1 C6 CT M -0.0030 -6.3780 0.8900 0.2020

4 3 H6 H1 E 0.4360 -6.1450 0.0550 0.0410

5 3 H7 H1 E -0.6540 -5.6890 1.0940 0.0410

6 3 C5 CT M -0.6940 -7.7210 0.7520 0.0610

7 6 O4 OS E 0.3130 -8.6660 0.3500 -0.3330

8 6 H5 H1 E -1.3730 -7.6600 0.0480 0.1170

9 6 C4 CT M -1.3530 -8.1830 2.0310 -0.0280

10 9 O3 OS E -2.4400 -7.3260 2.3750 -0.2950

11 9 H4 H1 E -0.6890 -8.1520 2.7520 0.1460

12 9 C3 CT M -1.8920 -9.5830 1.9320 0.0930

13 12 O2 OH S -2.3830 -9.9780 3.1990 -0.5700

14 13 H12 HO E -2.3730 -9.2260 3.7950 0.4020

15 12 H3 H1 E -2.6320 -9.5910 1.2890 0.1140

16 12 C2 CT M -0.8080 -10.4920 1.4330 0.1760

17 16 O1 OH S -1.2860 -11.8400 1.2400 -0.6220

18 17 H11 HO E -1.4110 -12.2610 2.0940 0.4030

19 16 H2 H1 E -0.1160 -10.5240 2.1260 0.1110

20 16 C1 CT M -0.1610 -9.9880 0.1820 0.0040

21 20 H1 H2 E 0.6030 -10.5660 -0.0290 0.1710

22 20 O33 OS M -1.0800 -10.0360 -0.9110 -0.2950

23 22 C40 CT M -0.5850 -10.5770 -2.1480 -0.0280

24 23 C41 CT 3 -1.0590 -9.7110 -3.3050 0.0610

25 24 O34 OS E -0.6300 -10.3350 -4.5260 -0.3330

26 24 C42 CT 3 -0.3860 -8.3380 -3.2920 0.2020

27 26 O35 OH S -0.8200 -7.5420 -4.3510 -0.6510

28 27 H10 HO E -1.0390 -8.0990 -5.1020 0.4170

29 26 H69 H1 E -0.5860 -7.8930 -2.4540 0.0410

30 26 H70 H1 E 0.5740 -8.4520 -3.3490 0.0410

31 24 H68 H1 E -2.0330 -9.6180 -3.2900 0.1170

32 23 H67 H1 E 0.3940 -10.5950 -2.1300 0.1460

33 23 C39 CT M -1.1120 -11.9890 -2.2830 0.0930

34 33 O32 OH S -0.5670 -12.8330 -1.2710 -0.5700

35 34 H30 HO E -0.3570 -13.6910 -1.6480 0.4020

36 33 H66 H1 E -2.0890 -11.9780 -2.2030 0.1140

37 33 C38 CT M -0.7320 -12.5560 -3.6200 0.1760

38 37 O31 OH S -1.3120 -13.8740 -3.8210 -0.6220

39 38 H29 HO E -1.4560 -14.2940 -2.9700 0.4030

40 37 H65 H1 E 0.2440 -12.6440 -3.6490 0.1110

41 37 C37 CT M -1.1560 -11.6290 -4.7370 0.0040

42 41 H64 H2 E -0.8170 -11.9780 -5.5880 0.1710

43 41 O28 OS M -2.5770 -11.6190 -4.7740 -0.2950

44 43 C34 CT M -3.2310 -11.9820 -6.0160 -0.0280

45 44 C35 CT 3 -3.7280 -10.7200 -6.6970 0.0610

46 45 O29 OS E -4.5870 -11.0850 -7.8080 -0.3330

47 45 C36 CT 3 -2.6100 -9.8940 -7.2890 0.2020

48 47 O30 OH S -3.0770 -8.7150 -7.9070 -0.6510

49 48 H28 HO E -3.2490 -8.8870 -8.8360 0.4170

50 47 H62 H1 E -1.9840 -9.6580 -6.5860 0.0410

51 47 H63 H1 E -2.1340 -10.4290 -7.9440 0.0410

52 45 H61 H1 E -4.2340 -10.1790 -6.0560 0.1170

53 44 H60 H1 E -2.6050 -12.4570 -6.6020 0.1460

54 44 C33 CT M -4.3730 -12.8940 -5.6180 0.0930

55 54 O27 OH S -3.8080 -14.1010 -5.0910 -0.5700

56 55 H27 HO E -3.6380 -14.7150 -5.8090 0.4020

57 54 H59 H1 E -4.9230 -12.4590 -4.9340 0.1140

58 54 C32 CT M -5.2080 -13.1760 -6.8460 0.1760

59 58 O26 OH S -6.3250 -14.0080 -6.5270 -0.6220

60 59 H26 HO E -6.5560 -13.8930 -5.6020 0.4030

61 58 H58 H1 E -4.6500 -13.6320 -7.5110 0.1110

62 58 C31 CT M -5.6980 -11.8610 -7.4340 0.0040

63 62 H57 H2 E -6.2340 -12.0520 -8.2330 0.1710

64 62 O23 OS M -6.5070 -11.2150 -6.4860 -0.2950

65 64 C28 CT M -7.8350 -10.7890 -6.8330 -0.0280

66 65 C29 CT 3 -7.8740 -9.2650 -6.7750 0.0610

67 66 O24 OS E -9.2270 -8.8050 -6.8740 -0.3330

68 66 C30 CT 3 -7.0810 -8.5800 -7.8280 0.2020

69 68 O25 OH S -7.5550 -8.8600 -9.1490 -0.6510

70 69 H25 HO E -7.7920 -9.7880 -9.2150 0.4170

71 68 H55 H1 E -6.1550 -8.8600 -7.7590 0.0410

72 68 H56 H1 E -7.1140 -7.6220 -7.6770 0.0410

73 66 H54 H1 E -7.5240 -8.9860 -5.9020 0.1170

74 65 H53 H1 E -8.0610 -11.0940 -7.7360 0.1460

75 65 C27 CT M -8.7710 -11.4070 -5.8310 0.0930

76 75 O22 OH S -8.7870 -12.8380 -5.9410 -0.5700

77 76 H24 HO E -9.0720 -13.2200 -5.1080 0.4020

78 75 H52 H1 E -8.4360 -11.1840 -4.9380 0.1140

79 75 C26 CT M -10.1370 -10.8180 -5.9560 0.1760

80 79 O21 OH S -11.0530 -11.3200 -4.9750 -0.6220

81 80 H23 HO E -11.1970 -10.6530 -4.3000 0.4030

82 79 H51 H1 E -10.4840 -11.0490 -6.8420 0.1110

83 79 C25 CT M -10.0680 -9.3010 -5.8580 0.0040

84 83 H50 H2 E -10.9690 -8.9350 -5.9860 0.1710

85 83 O18 OS M -9.6100 -8.9420 -4.5760 -0.2950

86 85 C22 CT M -10.2390 -7.8610 -3.8800 -0.0280

87 86 C23 CT 3 -9.3000 -6.6640 -3.8050 0.0610

88 87 O19 OS E -9.9690 -5.6470 -3.0640 -0.3330

89 87 C24 CT 3 -8.8480 -6.0580 -5.0910 0.2020

90 89 O20 OH S -9.9470 -5.7310 -5.9390 -0.6510

91 90 H22 HO E -10.2500 -6.5220 -6.3900 0.4170

92 89 H48 H1 E -8.3410 -5.2520 -4.9040 0.0410

93 89 H49 H1 E -8.2640 -6.6800 -5.5510 0.0410

94 87 H47 H1 E -8.5030 -6.9350 -3.3010 0.1170

95 86 H46 H1 E -11.0710 -7.6070 -4.3330 0.1460

96 86 C21 CT M -10.5360 -8.3450 -2.4840 0.0930

97 96 O17 OH S -11.4010 -9.4880 -2.4910 -0.5700

98 97 H21 HO E -11.5950 -9.7490 -1.5880 0.4020

99 96 H45 H1 E -9.6890 -8.6080 -2.0680 0.1140

100 96 C20 CT M -11.1190 -7.2210 -1.6760 0.1760

101 100 O16 OH S -11.2510 -7.6180 -0.2910 -0.6220

102 101 H20 HO E -10.8630 -6.9450 0.2740 0.4030

103 100 H44 H1 E -12.0100 -7.0120 -2.0280 0.1110

104 100 C19 CT M -10.2650 -5.9740 -1.7440 0.0040

105 104 H43 H2 E -10.7680 -5.2310 -1.3460 0.1710

106 104 O13 OS M -9.0730 -6.1840 -0.9690 -0.2950

107 106 C16 CT M -8.6340 -5.0900 -0.1240 -0.0280

108 107 C17 CT 3 -7.1990 -4.7450 -0.4850 0.0610

109 108 O14 OS E -6.7370 -3.7150 0.4320 -0.3330

110 108 C18 CT 3 -7.0040 -4.1590 -1.8500 0.2020

111 110 O15 OH S -7.8050 -3.0130 -2.0760 -0.6510

112 111 H19 HO E -8.0030 -2.9400 -3.0130 0.4170

113 110 H41 H1 E -6.0710 -3.9190 -1.9600 0.0410

114 110 H42 H1 E -7.2190 -4.8310 -2.5150 0.0410

115 108 H40 H1 E -6.6390 -5.5420 -0.3890 0.1170

116 107 H39 H1 E -9.2070 -4.3080 -0.2730 0.1460

117 107 C15 CT M -8.7510 -5.5410 1.3150 0.0930

118 117 O12 OH S -10.1250 -5.7500 1.6710 -0.5700

119 118 H18 HO E -10.3950 -5.0870 2.3100 0.4020

120 117 H38 H1 E -8.2590 -6.3780 1.4300 0.1140

121 117 C14 CT M -8.1510 -4.4890 2.2110 0.1760

122 121 O11 OH S -8.1000 -4.9550 3.5820 -0.6220

123 122 H17 HO E -7.3670 -5.5660 3.6860 0.4030

124 121 H37 H1 E -8.7090 -3.6840 2.1710 0.1110

125 121 C13 CT M -6.7560 -4.1340 1.7700 0.0040

126 125 H36 H2 E -6.4320 -3.3980 2.3290 0.1710

127 125 O8 OS M -5.9030 -5.2450 1.9600 -0.2950

128 127 C10 CT M -4.7780 -5.1190 2.8660 -0.0280

129 128 H32 H1 E -4.9540 -4.3860 3.4930 0.1460

130 128 C9 CT M -4.7440 -6.4220 3.6280 0.0930

131 130 O7 OH S -5.8890 -6.4720 4.4940 -0.5700

132 131 H15 HO E -6.0880 -5.5890 4.8130 0.4020

133 130 H31 H1 E -4.8150 -7.1570 2.9830 0.1140

134 130 C8 CT M -3.4700 -6.5780 4.3750 0.1760

135 134 O6 OH S -3.3810 -7.8990 4.9820 -0.6220

136 135 H14 HO E -3.0800 -8.5330 4.3270 0.4030

137 134 H9 H1 E -3.4270 -5.8990 5.0800 0.1110

138 134 C7 CT M -2.2850 -6.3930 3.4320 0.0040

139 138 H8 H2 E -1.4390 -6.5420 3.9040 0.1710

140 138 O9 OS M -2.3540 -5.0600 2.9270 -0.3330

141 140 C11 CT M -3.5150 -4.8270 2.1130 0.0610

142 141 H33 H1 E -3.4730 -5.4070 1.3240 0.1170

143 141 C12 CT M -3.3900 -3.3710 1.6690 0.2020

144 143 H34 H1 E -3.2810 -2.8030 2.4480 0.0410

145 143 H35 H1 E -4.2040 -3.1020 1.2130 0.0410

146 143 O10 OH M -2.2690 -3.1960 0.7900 -0.6510

147 146 H16 HO E -2.0760 -2.2600 0.6990 0.4170

CACQEDg

1 0 O1 OH M -3.1000 -5.8230 -2.8350 -0.5930

2 1 H1 HO E -2.9070 -5.4160 -1.9870 0.4380

3 1 C2 CA M -4.0010 -6.8860 -2.6510 0.3220

4 3 C1 CA M -4.4550 -7.1720 -1.4280 -0.2970

5 4 H3 HA E -4.1270 -6.7120 -0.6890 0.1510

6 4 C9 CA M -5.4640 -8.2120 -1.2550 0.0090

7 6 C8 CA M -6.0300 -8.5510 -0.0070 -0.2970

8 7 H8 HA E -5.6990 -8.1620 0.7700 0.1510

9 7 C7 CA M -7.0510 -9.4380 0.0690 0.3220

10 9 O2 OH S -7.6180 -9.8260 1.2660 -0.5930

11 10 H2 HO E -7.6470 -9.0760 1.8650 0.4380

12 9 C6 CA M -7.5010 -10.1420 -1.1020 -0.2110

13 12 H7 HA E -8.1640 -10.7890 -1.0400 0.1720

14 12 C5 CA M -6.9310 -9.8410 -2.3080 -0.1880

15 14 H6 HA E -7.2210 -10.2870 -3.0700 0.1560

16 14 C10 CA M -5.8910 -8.8430 -2.4260 0.0920

17 16 C4 CA M -5.3590 -8.5630 -3.7000 -0.1880

18 17 H5 HA E -5.6200 -9.0510 -4.4480 0.1560

19 17 C3 CA M -4.4380 -7.5420 -3.8010 -0.2110

20 19 H4 HA E -4.1100 -7.2920 -4.6340 0.1720

CAWRAT10h

1 0 C1 CT M 2.2010 2.7820 -9.1050 0.0160

2 1 H23 H1 E 2.3060 2.8550 -10.1770 0.0750

3 1 H24 H1 E 1.2380 2.3490 -8.8680 0.0750

4 1 O1 OS M 3.2500 1.9530 -8.5670 -0.3790

5 4 C2 CT M 3.0660 0.5700 -8.9060 0.0340

6 5 H25 H1 E 2.4720 0.5080 -9.8070 0.0780

7 5 H26 H1 E 4.0370 0.1270 -9.0830 0.0780

8 5 C3 CT M 2.3990 -0.1790 -7.8600 0.1040

9 8 H27 H1 E 2.1500 -1.1590 -8.2290 0.0670

10 8 H28 H1 E 1.4920 0.3400 -7.5860 0.0670

11 8 O2 OS M 3.2130 -0.3240 -6.6990 -0.4640

12 11 C4 CT M 2.6980 -1.1070 -5.6600 0.1040

13 12 H29 H1 E 1.8280 -0.6120 -5.2610 0.0670

14 12 H30 H1 E 2.4170 -2.0670 -6.0650 0.0670

15 12 C5 CT M 3.6320 -1.3250 -4.5780 0.0340

16 15 H31 H1 E 4.5350 -1.7580 -4.9880 0.0780

17 15 H32 H1 E 3.1860 -2.0150 -3.8770 0.0780

18 15 O3 OS M 3.9910 -0.1240 -3.8660 -0.3790

19 18 C6 CT M 3.3880 0.0100 -2.5710 0.0160

20 19 H33 H1 E 2.3290 -0.1890 -2.6390 0.0750

21 19 H34 H1 E 3.8440 -0.6850 -1.8810 0.0750

22 19 C7 CA M 3.6140 1.4110 -2.1000 0.0280

23 22 C13 CM S 2.8640 2.4660 -2.6160 0.0270

24 23 O4 OS S 1.8920 2.2090 -3.5690 -0.1920

25 24 C14 CT 3 0.6540 1.8130 -2.9660 -0.0250

26 25 H6 H1 E 0.0140 1.3220 -3.7170 0.0650

27 25 H7 H1 E 0.1520 2.7070 -2.6380 0.0650

28 25 H8 H1 E 0.7920 1.1380 -2.1480 0.0650

29 22 C8 CA M 4.5620 1.7060 -1.1230 -0.1970

30 29 H1 HA E 5.0960 0.8920 -0.6530 0.1620

31 29 C9 CA M 4.8520 2.9920 -0.7320 0.0280

32 31 C10 CT 3 5.7960 3.3110 0.4100 -0.1390

33 32 H2 HC E 5.9480 4.3560 0.5460 0.0530

34 32 H3 HC E 5.3960 2.8780 1.3200 0.0530

35 32 H4 HC E 6.7540 2.8340 0.1980 0.0530

36 31 C11 CM M 4.1570 4.0450 -1.3600 -0.1160

37 36 H5 HA E 4.3830 5.0670 -1.0890 0.1290

38 36 C12 CA M 3.1950 3.7940 -2.3110 -0.0380

39 38 N1 N M 2.5040 4.8570 -2.9770 0.0150

40 39 C18 C S 2.8130 4.9840 -4.2980 0.2700

41 40 O5 O E 3.8630 4.4680 -4.7490 -0.4620

42 39 C15 CT M 1.3300 5.4790 -2.3710 -0.0390

43 42 H9 H1 E 0.4970 4.7940 -2.4100 0.0410

44 42 H10 H1 E 1.5470 5.7280 -1.3410 0.0410

45 42 C16 CT M 0.9850 6.7440 -3.1370 0.0050

46 45 H11 HC E 1.7860 7.4600 -3.0300 0.0300

47 45 H12 HC E 0.0740 7.1670 -2.7400 0.0300

48 45 C17 CT M 0.7920 6.4200 -4.5970 -0.0390

49 48 H13 H1 E 0.6490 7.3360 -5.1540 0.0410

50 48 H14 H1 E -0.0740 5.7880 -4.7150 0.0410

51 48 N2 N M 1.9980 5.7180 -5.1000 0.0150

52 51 C19 CA M 2.1820 5.6030 -6.5390 -0.0380

53 52 C20 CM M 2.4770 6.6900 -7.2990 -0.1160

54 53 H15 HA E 2.5780 7.6600 -6.8320 0.1290

55 53 C21 CA M 2.6520 6.5600 -8.6870 0.0280

56 55 C22 CT 3 2.9420 7.7630 -9.5410 -0.1390

57 56 H16 HC E 2.5040 8.6370 -9.0590 0.0530

58 56 H17 HC E 4.0100 7.8830 -9.5850 0.0530

59 56 H18 HC E 2.5410 7.6880 -10.5420 0.0530

60 55 C23 CA M 2.5870 5.2790 -9.2360 -0.1970

61 60 H19 HA E 2.7670 5.1600 -10.2930 0.1620

62 60 C24 CA M 2.3020 4.1640 -8.4900 0.0280

63 62 C25 CM M 2.0620 4.3350 -7.1060 0.0270

64 63 O6 OS M 1.7360 3.2310 -6.3550 -0.1920

65 64 C26 CT M 0.3220 2.9640 -6.2710 -0.0250

66 65 H20 H1 E 0.1430 1.9860 -5.8700 0.0650

67 65 H21 H1 E -0.1430 3.0440 -7.2490 0.0650

68 65 H22 H1 E -0.1340 3.7050 -5.6140 0.0650

CAWRAT10g

1 0 N1 N3 M 4.4930 2.1310 -5.8420 -0.5860

2 1 H10 H E 4.4200 1.2710 -5.3630 0.3660

3 1 H11 H E 4.0650 2.0230 -6.6160 0.3660

4 1 H12 H E 4.1990 2.8520 -5.1340 0.3660

5 1 C1 CT M 5.9760 2.3310 -6.0610 0.4640

6 5 C3 CT 3 6.4820 1.2080 -6.9570 -0.4360

7 6 H4 HC E 7.5450 1.2780 -7.1720 0.1480

8 6 H5 HC E 5.9200 1.4050 -7.8430 0.1480

9 6 H6 HC E 6.2660 0.2130 -6.5920 0.1480

10 5 C4 CT 3 6.6200 2.2740 -4.6730 -0.4360

11 10 H7 HC E 7.6650 2.5100 -4.8000 0.1480

12 10 H8 HC E 6.5100 1.3040 -4.2300 0.1480

13 10 H9 HC E 6.1800 3.0230 -4.0050 0.1480

14 5 C2 CT M 6.1640 3.6900 -6.7120 -0.4360

15 14 H1 HC E 7.1730 3.8660 -7.0340 0.1480

16 14 H2 HC E 5.8420 4.4860 -6.0580 0.1480

17 14 H3 HC E 5.5110 3.6640 -7.5820 0.1480

CECMEC10h

1 0 C9 CT M -2.8580 7.2210 3.7660 0.0030

2 1 H13 H1 E -1.9790 7.3590 3.5280 0.0550

3 1 H64 H1 E -3.2380 6.4100 4.4000 0.0550

4 1 H82 H1 E -3.3700 7.1690 2.6560 0.0550

5 1 O5 OS M -3.2650 8.4870 4.2700 -0.3000

6 5 C6 CT M -2.6750 8.7830 5.5490 -0.0170

7 6 H6 H1 E -3.2330 9.4720 5.9860 0.0860

8 6 H7 H1 E -2.6720 7.8810 6.1310 0.0860

9 6 C5 CT M -1.2380 9.2940 5.4060 0.0200

10 9 O4 OS E -1.3460 10.6070 4.7910 -0.3150

11 9 H5 H1 E -0.6630 8.6650 4.8100 0.1480

12 9 C4 CT M -0.6000 9.4270 6.7840 -0.0070

13 12 O3 OS E -0.2240 8.1280 7.2370 -0.2360

14 12 H4 H1 E -1.3100 9.8520 7.4920 0.1440

15 12 C3 CT M 0.6440 10.3100 6.7930 -0.0350

16 15 O2 OS S 0.8160 10.6920 8.1700 -0.3450

17 16 C8 CT 3 2.1100 11.0200 8.5770 -0.0150

18 17 H12 H1 E 2.7510 11.8460 8.4170 0.0650

19 17 H63 H1 E 3.0130 10.5880 7.8490 0.0650

20 17 H81 H1 E 2.4750 10.7060 9.6600 0.0650

21 15 H3 H1 E 1.4460 9.8750 6.3960 0.1660

22 15 C2 CT M 0.5390 11.5210 5.9110 0.0310

23 22 O1 OS S 1.8620 12.0550 5.7810 -0.2340

24 23 C7 CT 3 1.8960 13.4430 5.4830 -0.1270

25 24 H11 H1 E 0.9400 13.8400 5.9860 0.0890

26 24 H62 H1 E 1.4110 13.5790 4.4800 0.0890

27 24 H80 H1 E 2.7440 13.5550 5.3520 0.0890

28 22 H2 H1 E -0.1080 12.1780 6.4220 0.1470

29 22 C1 CT M -0.0890 11.2190 4.5360 0.0060

30 29 H1 H2 E -0.2490 12.0830 3.9250 0.1690

31 29 O8 OS M 0.7440 10.4140 3.7570 -0.2360

32 31 C13 CT M 0.7880 10.6900 2.3530 -0.0070

33 32 C14 CT 3 -0.1730 9.7800 1.5780 0.0200

34 33 O9 OS E 0.1510 9.7190 0.1760 -0.3150

35 33 C15 CT 3 -1.5930 10.2880 1.6160 -0.0170

36 35 O10 OS S -2.4320 9.3700 0.9670 -0.3000

37 36 C18 CT 3 -3.7900 9.8230 0.9580 0.0030

38 37 H16 H1 E -4.2440 9.2580 0.3960 0.0550

39 37 H67 H1 E -4.0850 9.9700 2.1270 0.0550

40 37 H85 H1 E -3.8040 10.6590 0.7930 0.0550

41 35 H33 H1 E -1.9020 10.3740 2.6430 0.0860

42 35 H34 H1 E -1.6670 11.2520 1.1100 0.0860

43 33 H32 H1 E -0.0620 8.7360 1.9690 0.1480

44 32 H31 H1 E 0.4330 11.6560 2.1010 0.1440

45 32 C12 CT M 2.2720 10.4550 1.9490 -0.0350

46 45 O7 OS S 3.0120 11.5470 2.4590 -0.3450

47 46 C17 CT 3 4.1200 11.1690 3.2650 -0.0150

48 47 H15 H1 E 4.6310 10.4690 2.6030 0.0650

49 47 H66 H1 E 3.5170 11.0860 3.9640 0.0650

50 47 H84 H1 E 4.3430 11.8930 3.7660 0.0650

51 45 H30 H1 E 2.6830 9.6380 2.3920 0.1660

52 45 C11 CT M 2.3880 10.3980 0.4480 0.0310

53 52 O6 OS S 3.7560 10.1340 0.0910 -0.2340

54 53 C16 CT 3 4.2630 11.0550 -0.8140 -0.1270

55 54 H14 H1 E 4.2940 12.0410 -0.3510 0.0890

56 54 H65 H1 E 3.6240 11.0880 -1.6970 0.0890

57 54 H83 H1 E 5.2710 10.7600 -1.1060 0.0890

58 52 H9 H1 E 2.0960 11.3000 0.0260 0.1470

59 52 C10 CT M 1.4930 9.3390 -0.1150 0.0060

60 59 H8 H2 E 1.5780 9.2340 -1.1630 0.1690

61 59 O13 OS M 1.7820 8.0830 0.4990 -0.2360

62 61 C22 CT M 1.9680 6.9370 -0.3360 -0.0070

63 62 C23 CT 3 0.7030 6.0910 -0.4070 0.0200

64 63 O14 OS E 0.9960 4.8690 -1.1340 -0.3150

65 63 C24 CT 3 -0.3870 6.7750 -1.2160 -0.0170

66 65 O15 OS S -1.5870 6.0130 -1.0600 -0.3000

67 66 C27 CT 3 -2.7650 6.6870 -1.2920 0.0030

68 67 H19 H1 E -3.5250 6.3400 -0.5920 0.0550

69 67 H70 H1 E -2.6060 7.7560 -1.1540 0.0550

70 67 H88 H1 E -3.0980 6.4970 -2.3120 0.0550

71 65 H40 H1 E -0.5620 7.8340 -0.8460 0.0860

72 65 H41 H1 E -0.0950 6.8130 -2.3390 0.0860

73 63 H39 H1 E 0.3690 5.8400 0.7140 0.1480

74 62 H38 H1 E 2.2380 7.2400 -1.3870 0.1440

75 62 C21 CT M 3.0910 6.1200 0.3400 -0.0350

76 75 O12 OS S 4.3220 6.8440 0.2660 -0.3450

77 76 C26 CT 3 4.8520 7.1690 1.5500 -0.0150

78 77 H18 H1 E 4.7450 6.1720 2.2600 0.0650

79 77 H69 H1 E 3.9550 7.6440 2.2200 0.0650

80 77 H87 H1 E 5.5650 7.1690 1.6910 0.0650

81 75 H37 H1 E 2.8300 6.0060 1.4270 0.1660

82 75 C20 CT M 3.2720 4.7910 -0.4060 0.0310

83 82 O11 OS S 4.2200 3.9930 0.3030 -0.2340

84 83 C25 CT 3 5.4780 3.8480 -0.3580 -0.1270

85 84 H17 H1 E 6.0970 3.0390 0.3700 0.0890

86 84 H68 H1 E 6.1260 4.7070 -0.5310 0.0890

87 84 H86 H1 E 5.0890 3.3710 -1.3610 0.0890

88 82 H36 H1 E 3.7410 4.9380 -1.4400 0.1470

89 82 C19 CT M 1.9560 4.0330 -0.5020 0.0060

90 89 H35 H2 E 1.9810 3.0390 -1.1500 0.1690

91 89 O18 OS M 1.5200 3.6130 0.7690 -0.2360

92 91 C31 CT M 0.9070 2.3070 0.8420 -0.0070

93 92 C32 CT 3 -0.4920 2.4880 1.4600 0.0200

94 93 O19 OS E -1.0240 1.2010 1.8620 -0.3150

95 93 C33 CT 3 -1.4980 3.0740 0.5190 -0.0170

96 95 O20 OS S -1.5970 2.3030 -0.6610 -0.3000

97 96 C36 CT 3 -2.7380 2.6940 -1.4600 0.0030

98 97 H22 H1 E -2.5830 1.8040 -2.2600 0.0550

99 97 H73 H1 E -3.5880 2.6350 -0.9510 0.0550

100 97 H91 H1 E -2.7960 3.7980 -1.3870 0.0550

101 95 H47 H1 E -1.2950 4.1070 0.2510 0.0860

102 95 H48 H1 E -2.5070 3.0860 1.0040 0.0860

103 93 H46 H1 E -0.4200 3.0620 2.3520 0.1480

104 92 H45 H1 E 0.8070 1.8990 -0.1720 0.1440

105 92 C30 CT M 1.8070 1.4390 1.7260 -0.0350

106 105 O17 OS S 2.9540 1.1160 0.9220 -0.3450

107 106 C35 CT 3 4.3080 1.1130 1.4140 -0.0150

108 107 H21 H1 E 5.0440 0.8310 0.6610 0.0650

109 107 H72 H1 E 4.2570 0.4750 2.1800 0.0650

110 107 H90 H1 E 4.6100 2.1060 1.7470 0.0650

111 105 H44 H1 E 2.1630 1.9470 2.6560 0.1660

112 105 C29 CT M 1.0930 0.1780 2.1820 0.0310

113 112 O16 OS S 1.9370 -0.5130 3.0830 -0.2340

114 113 C34 CT 3 2.0660 -1.8750 2.8370 -0.1270

115 114 H20 H1 E 2.3720 -2.3500 3.5550 0.0890

116 114 H71 H1 E 1.3060 -1.9940 3.5810 0.0890

117 114 H89 H1 E 1.2500 -2.3500 2.2070 0.0890

118 112 H43 H1 E 0.8780 -0.5220 1.3480 0.1470

119 112 C28 CT M -0.2310 0.5340 2.8240 0.0060

120 119 H42 H2 E -0.7880 -0.3320 3.0790 0.1690

121 119 O23 OS M 0.0190 1.3670 3.9470 -0.2360

122 121 C40 CT M -0.7850 1.2110 5.1350 -0.0070

123 122 C41 CT 3 -1.7510 2.4000 5.2740 0.0200

124 123 O24 OS E -2.3230 2.4050 6.5830 -0.3150

125 123 C42 CT 3 -2.9350 2.2960 4.3100 -0.0170

126 125 O25 OS S -3.5770 3.5660 4.3090 -0.3000

127 126 C45 CT 3 -4.7690 3.4830 3.5630 0.0030

128 127 H27 H1 E -4.6980 3.2290 2.3920 0.0550

129 127 H76 H1 E -5.2430 2.6830 3.9510 0.0550

130 127 H94 H1 E -5.1250 4.7480 3.3560 0.0550

131 125 H24 H1 E -3.6810 1.5430 4.6780 0.0860

132 125 H53 H1 E -2.7140 2.1130 3.3040 0.0860

133 123 H52 H1 E -1.2220 3.3710 5.1670 0.1480

134 122 H51 H1 E -1.3690 0.3320 5.0350 0.1440

135 122 C39 CT M 0.1970 1.1630 6.2990 -0.0350

136 135 O22 OS S 0.8180 -0.1420 6.2480 -0.3450

137 136 C44 CT 3 2.2880 -0.0620 6.2750 -0.0150

138 137 H26 H1 E 2.8960 0.2920 5.4420 0.0650

139 137 H75 H1 E 2.3990 -1.1630 6.4750 0.0650

140 137 H93 H1 E 2.6100 0.6880 7.0170 0.0650

141 135 H50 H1 E 0.9430 1.9700 6.2110 0.1660

142 135 C38 CT M -0.4890 1.3150 7.6260 0.0310

143 142 O21 OS S 0.5280 1.4030 8.6260 -0.2340

144 143 C43 CT 3 0.0980 1.2040 9.9380 -0.1270

145 144 H25 H1 E -0.5880 1.7090 10.0690 0.0890

146 144 H74 H1 E 0.1720 0.1190 9.9370 0.0890

147 144 H92 H1 E 0.7160 1.1630 10.5710 0.0890

148 142 H23 H1 E -1.1350 0.4620 7.8320 0.1470

149 142 C37 CT M -1.3740 2.5470 7.6220 0.0060

150 149 H49 H2 E -1.8720 2.7300 8.4840 0.1690

151 149 O28 OS M -0.6030 3.7220 7.4620 -0.2360

152 151 C49 CT M -0.8590 4.8380 8.2990 -0.0070

153 152 H57 H1 E -1.2740 4.4630 9.2100 0.1440

154 152 C48 CT M 0.4790 5.4880 8.5380 -0.0350

155 154 O27 OS S 1.2500 4.6720 9.4120 -0.3450

156 155 C53 CT 3 2.4460 4.1570 8.8930 -0.0150

157 156 H29 H1 E 3.0000 4.9850 8.2060 0.0650

158 156 H78 H1 E 2.2300 3.9640 7.9420 0.0650

159 156 H96 H1 E 2.8610 3.5610 9.9240 0.0650

160 154 H56 H1 E 0.9720 5.6020 7.5590 0.1660

161 154 C47 CT M 0.3150 6.8720 9.2140 0.0310

162 161 O26 OS S 1.5800 7.5160 9.2640 -0.2340

163 162 C52 CT 3 1.9530 7.8910 10.5290 -0.1270

164 163 H28 H1 E 1.6070 7.5960 11.4830 0.0890

165 163 H77 H1 E 1.1050 8.3100 11.0700 0.0890

166 163 H95 H1 E 2.7740 8.2610 10.3730 0.0890

167 161 H55 H1 E -0.0460 6.7890 10.2810 0.1470

168 161 C46 CT M -0.7000 7.7480 8.5300 0.0060

169 168 H54 H2 E -0.8480 8.6650 9.1440 0.1690

170 168 O29 OS M -1.9220 7.0340 8.4150 -0.3150

171 170 C50 CT M -1.8130 5.8260 7.6300 0.0200

172 171 H58 H1 E -1.3680 6.0300 6.6340 0.1480

173 171 C51 CT M -3.2550 5.3220 7.4280 -0.0170

174 173 H59 H1 E -3.8530 6.1010 7.0170 0.0860

175 173 H60 H1 E -3.2360 4.4630 6.6600 0.0860

176 173 O30 OS M -3.7740 4.9120 8.7120 -0.3000

177 176 C54 CT M -5.1580 5.0710 8.7990 0.0030

178 177 H10 H1 E -5.5720 6.0060 8.3780 0.0550

179 177 H61 H1 E -5.7380 4.7720 9.2500 0.0550

180 177 H79 H1 E -5.5620 4.3440 7.9550 0.0550

CECMEC10g

1 0 O2 OH M 5.6440 8.4530 4.6570 -0.6900

2 1 H8 HO E 6.8750 8.4270 4.3210 0.4930

3 1 C7 C M 5.4050 8.0620 5.8510 0.6970

4 3 O3 O E 6.2360 7.8860 6.7420 -0.5760

5 3 C4 CT M 3.8830 7.9740 6.2010 0.2270

6 5 O1 OH S 3.8050 7.8270 7.5890 -0.6540

7 6 H6 HO E 2.6450 7.5490 7.9550 0.4230

8 5 H3 H1 E 3.4320 8.9970 5.8140 0.0640

9 5 C1 CA M 3.2080 6.8650 5.4460 0.0760

10 9 C2 CA M 2.0960 7.1240 4.6200 -0.1430

11 10 H1 HA E 1.7020 8.1900 4.3610 0.1300

12 10 C5 CA M 1.4410 6.1010 3.9850 -0.1760

13 12 H4 HA E 0.6380 6.4570 3.2110 0.1470

14 12 C8 CA M 1.8580 4.8000 4.1740 -0.1100

15 14 H7 HA E 1.2710 3.9410 3.7000 0.1330

16 14 C6 CA M 2.9570 4.5130 4.9610 -0.1760

17 16 H5 HA E 3.3960 3.3230 5.0740 0.1470

18 16 C3 CA M 3.6320 5.5670 5.5790 -0.1430

19 18 H2 HA E 4.4770 5.4120 6.2500 0.1300

CENHAEh

1 0 C1 CT M 1.5980 3.2010 -0.1280 0.0830

2 1 H1 H1 E 2.0430 2.8650 -1.0700 0.0370

3 1 H2 H1 E 2.0670 3.9070 0.2540 0.0370

4 1 C2 CT M 0.1820 3.5940 -0.3230 0.0830

5 4 H3 H1 E -0.2850 3.8050 0.5710 0.0370

6 4 H4 H1 E 0.1170 4.4710 -0.8320 0.0370

7 4 O2 OS M -0.4720 2.5610 -1.0530 -0.3150

8 7 C3 CT M -1.8260 2.8490 -1.3260 0.0830

9 8 H5 H1 E -2.3790 2.9370 -0.4360 0.0370

10 8 H6 H1 E -1.8810 3.7190 -1.8160 0.0370

11 8 C4 CT M -2.3920 1.7300 -2.1340 0.0830

12 11 H7 H1 E -3.3520 1.9240 -2.3940 0.0370

13 11 H8 H1 E -1.8060 1.5340 -2.9730 0.0370

14 11 O3 OS M -2.4550 0.5720 -1.3070 -0.3150

15 14 C5 CT M -2.9570 -0.5590 -2.0120 0.0830

16 15 H9 H1 E -3.8360 -0.3470 -2.4820 0.0370

17 15 H10 H1 E -2.4650 -0.7230 -2.8380 0.0370

18 15 C6 CT M -2.9890 -1.7120 -1.0800 0.0830

19 18 H11 H1 E -3.4380 -1.5190 -0.1190 0.0370

20 18 H12 H1 E -3.4550 -2.4600 -1.5460 0.0370

21 18 O4 OS M -1.6550 -2.1150 -0.7950 -0.3150

22 21 C7 CT M -1.5980 -3.2010 0.1280 0.0830

23 22 H13 H1 E -2.0670 -3.9070 -0.2540 0.0370

24 22 H14 H1 E -2.0430 -2.8650 1.0700 0.0370

25 22 C8 CT M -0.1820 -3.5940 0.3230 0.0830

26 25 H15 H1 E 0.2850 -3.8050 -0.5710 0.0370

27 25 H16 H1 E -0.1170 -4.4710 0.8320 0.0370

28 25 O5 OS M 0.4720 -2.5610 1.0530 -0.3150

29 28 C9 CT M 1.8260 -2.8490 1.3260 0.0830

30 29 H17 H1 E 2.3790 -2.9370 0.4360 0.0370

31 29 H18 H1 E 1.8810 -3.7190 1.8160 0.0370

32 29 C10 CT M 2.3920 -1.7300 2.1340 0.0830

33 32 H19 H1 E 1.8060 -1.5340 2.9730 0.0370

34 32 H20 H1 E 3.3520 -1.9240 2.3940 0.0370

35 32 O6 OS M 2.4550 -0.5720 1.3070 -0.3150

36 35 C11 CT M 2.9570 0.5590 2.0120 0.0830

37 36 H21 H1 E 2.4650 0.7230 2.8380 0.0370

38 36 H22 H1 E 3.8360 0.3470 2.4820 0.0370

39 36 C12 CT M 2.9890 1.7120 1.0800 0.0830

40 39 H23 H1 E 3.4380 1.5190 0.1190 0.0370

41 39 H24 H1 E 3.4550 2.4600 1.5460 0.0370

42 39 O1 OS M 1.6550 2.1150 0.7950 -0.3150

CENHAEg

1 0 N1 NT M -0.9460 0.6680 1.3130 -0.8930

2 1 H1 H E -0.1200 0.7380 1.4590 0.4130

3 1 H2 H E -1.3590 0.5350 0.4920 0.4130

4 1 C1 CZ M -1.5110 -0.0940 2.2140 0.6400

5 4 N2 N1 M -2.0470 -0.6950 2.9800 -0.5730

CICVUFh

1 0 O1 OS M 8.6770 1.2100 -1.9000 -0.3030

2 1 C1 CT M 9.3730 1.6910 -3.0490 0.0650

3 2 H1 H1 E 9.6840 0.8650 -3.6790 0.0430

4 2 H2 H1 E 8.7190 2.3430 -3.6060 0.0430

5 2 C2 CT M 10.5560 2.4690 -2.5990 0.0650

6 5 H3 H1 E 11.0310 2.9400 -3.4590 0.0430

7 5 H4 H1 E 10.2560 3.2250 -1.8850 0.0430

8 5 O2 OS M 11.4560 1.5560 -2.0010 -0.3030

9 8 C3 CT M 12.6840 2.1110 -1.6930 0.0650

10 9 H5 H1 E 13.1570 2.4690 -2.5990 0.0430

11 9 H6 H1 E 12.5290 2.9400 -1.0250 0.0430

12 9 C4 CT M 13.5910 1.1530 -1.0290 0.0650

13 12 H7 H1 E 13.6690 0.2600 -1.6290 0.0430

14 12 H8 H1 E 14.5710 1.5960 -0.9150 0.0430

15 12 O3 OS M 13.1210 0.8060 0.2360 -0.3030

16 15 C5 CT M 14.0160 0.0230 0.9960 0.0650

17 16 H9 H1 E 14.3310 -0.8400 0.4210 0.0430

18 16 H10 H1 E 14.8720 0.6220 1.2450 0.0430

19 16 C7 CT M 13.3190 -0.4230 2.2550 0.0650

20 19 H13 H1 E 13.9910 -1.0080 2.8550 0.0430

21 19 H14 H1 E 12.9900 0.4370 2.8190 0.0430

22 19 O4 OS M 12.2170 -1.2100 1.9000 -0.3030

23 22 C9 CT M 11.5210 -1.6910 3.0490 0.0650

24 23 H17 H1 E 11.2100 -0.8650 3.6790 0.0430

25 23 H18 H1 E 12.1750 -2.3430 3.6060 0.0430

26 23 C11 CT M 10.3380 -2.4690 2.5990 0.0650

27 26 H21 H1 E 9.8630 -2.9400 3.4590 0.0430

28 26 H22 H1 E 10.6380 -3.2250 1.8850 0.0430

29 26 O6 OS M 9.4380 -1.5560 2.0010 -0.3030

30 29 C12 CT M 8.2100 -2.1110 1.6930 0.0650

31 30 H23 H1 E 7.7370 -2.4690 2.5990 0.0430

32 30 H24 H1 E 8.3650 -2.9400 1.0250 0.0430

33 30 C10 CT M 7.3030 -1.1530 1.0290 0.0650

34 33 H19 H1 E 7.2250 -0.2600 1.6290 0.0430

35 33 H20 H1 E 6.3230 -1.5960 0.9150 0.0430

36 33 O5 OS M 7.7730 -0.8060 -0.2360 -0.3030

37 36 C8 CT M 6.8780 -0.0230 -0.9960 0.0650

38 37 H15 H1 E 6.5630 0.8400 -0.4210 0.0430

39 37 H16 H1 E 6.0220 -0.6220 -1.2450 0.0430

40 37 C6 CT M 7.5750 0.4230 -2.2550 0.0650

41 40 H11 H1 E 6.9030 1.0080 -2.8550 0.0430

42 40 H12 H1 E 7.9040 -0.4370 -2.8190 0.0430

CICVUFg

1 0 N1 NH M 8.2910 2.6140 0.8400 -0.9750

2 1 H1 H E 8.3600 2.4610 -0.0370 0.4120

3 1 H2 H E 7.6170 2.9560 1.3360 0.4120

4 1 C1 C2 M 9.2830 2.0660 1.5520 1.0110

5 4 N2 NH B 10.2780 1.4890 0.8820 -0.9750

6 5 H3 H E 10.8560 1.0920 1.3180 0.4120

7 5 H4 H E 10.2160 1.4110 0.0180 0.4120

8 4 N3 NE M 9.3600 2.0800 2.8770 -0.8970

9 8 C2 CR M 8.3820 2.6710 3.6170 0.7800

10 9 N4 NB M 7.2990 3.3440 3.2360 -0.6370

11 10 C3 CB M 6.7060 3.7750 4.4310 0.2340

12 11 C4 CM M 5.5870 4.5670 4.6440 -0.2020

13 12 H6 HA E 4.9970 4.9300 3.8070 0.1550

14 12 C5 CA M 5.2300 4.8480 5.9580 -0.1890

15 14 H7 HA E 4.3690 5.4680 6.1500 0.1510

16 14 C6 CA M 5.9640 4.3600 7.0320 -0.2050

17 16 H8 HA E 5.6460 4.5770 8.0350 0.1580

18 16 C7 CA M 7.1020 3.5930 6.8440 -0.2160

19 18 H9 HA E 7.6930 3.2170 7.6690 0.1670

20 18 C8 CN M 7.4510 3.3190 5.5260 0.0830

21 20 N5 NA M 8.5090 2.6110 4.9840 -0.4410

22 21 H5 H E 9.2170 2.3520 5.3630 0.3480

CIXCOBh

1 0 C7 CT M 0.3860 4.1520 2.3230 -0.0400

2 1 H1 HC E 1.0240 4.8900 2.4170 0.0480

3 1 H2 HC E -0.5240 4.4860 2.4700 0.0480

4 1 H3 HC E 0.4540 3.7770 1.4200 0.0480

5 1 C5 CA M 0.6960 3.0920 3.3280 0.0380

6 5 C6 CA M 0.7330 3.1600 4.7010 -0.0450

7 6 S4 S M 0.4580 4.4450 5.8630 -0.1330

8 7 C2 CA M 0.8340 3.4280 7.2700 0.0600

9 8 S3 S M 1.1640 1.7130 6.9560 -0.1330

10 9 C3 CA M 1.0560 1.8830 5.2120 -0.0450

11 10 C4 CA M 1.2080 1.0200 4.1570 0.0380

12 11 C8 CT 3 1.5290 -0.4390 4.1390 -0.0400

13 12 H4 HC E 1.5650 -0.7550 3.2120 0.0480

14 12 H5 HC E 0.8360 -0.9320 4.6250 0.0480

15 12 H6 HC E 2.3970 -0.5880 4.5690 0.0480

16 11 N1 N* M 0.9830 1.7720 3.0020 -0.1070

17 16 C9 CT M 1.1220 1.2440 1.6290 -0.0400

18 17 H7 H1 E 0.8530 0.2910 1.6200 0.0670

19 17 H8 H1 E 0.5080 1.7370 1.0300 0.0670

20 17 C10 CT M 2.5420 1.3640 1.0970 -0.0040

21 20 H9 H1 E 2.6090 0.9520 0.2000 0.0650

22 20 H10 H1 E 3.1760 0.9070 1.7030 0.0650

23 20 O1 OS M 2.8420 2.7550 1.0270 -0.2400

24 23 C11 CT M 4.1940 3.0550 0.7190 -0.0340

25 24 H11 H1 E 4.7950 2.6110 1.3690 0.0770

26 24 H12 H1 E 4.4160 2.7280 -0.1870 0.0770

27 24 C12 CT M 4.3650 4.5390 0.7880 0.0570

28 27 H13 H1 E 3.7650 4.9730 0.1330 0.0530

29 27 H14 H1 E 5.2970 4.7760 0.5570 0.0530

30 27 O2 OS M 4.0620 5.0180 2.1110 -0.2980

31 30 C13 CT M 3.7700 6.4130 2.0900 0.0640

32 31 H15 H1 E 4.5670 6.9150 1.7830 0.0410

33 31 H16 H1 E 3.0310 6.5900 1.4540 0.0410

34 31 C14 CT M 3.3750 6.8870 3.4720 0.1060

35 34 H17 H1 E 3.0330 7.8150 3.4270 0.0570

36 34 H18 H1 E 2.6610 6.3070 3.8370 0.0570

37 34 O3 OS M 4.5390 6.8280 4.3130 -0.3420

38 37 C15 CA M 4.4070 7.3370 5.5970 0.2220

39 38 C20 CA B 5.5890 7.4550 6.3140 -0.1980

40 39 C19 CA S 5.5760 7.9080 7.6370 -0.1980

41 40 H21 HA E 6.3860 7.9820 8.1290 0.1510

42 39 H22 HA E 6.4140 7.2270 5.9030 0.1510

43 38 C16 CA M 3.1970 7.6720 6.1940 -0.1980

44 43 H19 HA E 2.3850 7.5900 5.7060 0.1510

45 43 C17 CA M 3.1810 8.1250 7.5070 -0.1980

46 45 H20 HA E 2.3550 8.3500 7.9170 0.1510

47 45 C18 CA M 4.3550 8.2520 8.2260 0.2220

48 47 O4 OS M 4.2290 8.6980 9.5230 -0.3420

49 48 C21 CT M 5.4360 9.0700 10.2170 0.1060

50 49 H23 H1 E 5.9710 8.2660 10.4290 0.0570

51 49 H24 H1 E 5.9830 9.6700 9.6500 0.0570

52 49 C22 CT M 5.0360 9.7780 11.4850 0.0640

53 52 H25 H1 E 4.3520 10.4650 11.2820 0.0410

54 52 H26 H1 E 5.8240 10.2350 11.8740 0.0410

55 52 O5 OS M 4.5130 8.8460 12.4230 -0.2980

56 55 C23 CT M 3.8930 9.5330 13.5120 0.0570

57 56 H27 H1 E 4.5440 10.1460 13.9340 0.0530

58 56 H28 H1 E 3.1320 10.0700 13.1760 0.0530

59 56 C24 CT M 3.4010 8.5340 14.5290 -0.0340

60 59 H29 H1 E 4.1560 7.9860 14.8620 0.0770

61 59 H30 H1 E 2.9930 9.0040 15.3000 0.0770

62 59 O6 OS M 2.4360 7.7060 13.8930 -0.2400

63 62 C25 CT M 1.8320 6.7730 14.7790 -0.0040

64 63 H31 H1 E 2.4650 6.0410 14.9870 0.0650

65 63 H32 H1 E 1.5780 7.2190 15.6250 0.0650

66 63 C26 CT M 0.6040 6.2190 14.0910 -0.0400

67 66 H33 H1 E -0.0820 6.9320 14.0450 0.0670

68 66 H34 H1 E 0.2360 5.4850 14.6420 0.0670

69 66 N2 N* M 0.8490 5.7130 12.7340 -0.1070

70 69 C27 CA M 1.1850 4.3910 12.4310 0.0380

71 70 C32 CT 3 1.4330 3.3510 13.4590 -0.0400

72 71 H38 HC E 2.3220 2.9600 13.3210 0.0480

73 71 H39 HC E 0.7530 2.6490 13.3860 0.0480

74 71 H40 HC E 1.3900 3.7560 14.3510 0.0480

75 70 C28 CA M 1.1480 4.2970 11.0600 -0.0450

76 75 S2 S M 1.3610 2.9850 9.9160 -0.1330

77 76 C1 CA M 0.8950 3.9590 8.5100 0.0600

78 77 S1 S M 0.5820 5.6790 8.7950 -0.1330

79 78 C29 CA M 0.7790 5.5560 10.5330 -0.0450

80 79 C30 CA M 0.5960 6.4330 11.5740 0.0380

81 80 C31 CT M 0.1910 7.8770 11.5540 -0.0400

82 81 H35 HC E 0.1570 8.2180 12.4730 0.0480

83 81 H36 HC E -0.6930 7.9620 11.1410 0.0480

84 81 H37 HC E 0.8450 8.3920 11.0380 0.0480

CIXCOBg

1 0 C2 CA M 5.1590 2.3440 7.7240 -0.2430

2 1 H2 HA E 5.6040 1.7940 8.3600 0.1270

3 1 C1 CA M 5.0700 1.9290 6.4200 -0.0450

4 3 H1 H4 E 5.4490 1.0980 6.1590 0.1280

5 3 N1 N* M 4.4480 2.6970 5.5130 -0.0950

6 5 C6 CT 3 4.3640 2.2640 4.1110 -0.1340

7 6 H5 H1 E 3.8810 2.9380 3.5900 0.0780

8 6 H6 H1 E 3.8880 1.4100 4.0620 0.0780

9 6 H7 H1 E 5.2680 2.1550 3.7490 0.0780

10 5 C5 CA M 3.9120 3.8790 5.8620 -0.0450

11 10 H4 H4 E 3.4900 4.4150 5.2010 0.1280

12 10 C4 CA M 3.9680 4.3220 7.1530 -0.2430

13 12 H3 HA E 3.5680 5.1510 7.3870 0.1270

14 12 C3 CA M 4.6060 3.5610 8.1340 0.0580

15 14 C7 CA M 4.6970 4.0300 9.5330 0.0580

16 15 C8 CA M 4.1430 5.2480 9.9430 -0.2430

17 16 H14 HA E 3.6990 5.7970 9.3070 0.1270

18 16 C9 CA M 4.2320 5.6620 11.2470 -0.0450

19 18 H8 H4 E 3.8530 6.4940 11.5080 0.1280

20 18 N2 N* M 4.8540 4.8950 12.1540 -0.0950

21 20 C12 CT 3 4.9390 5.3280 13.5560 -0.1340

22 21 H11 H1 E 5.4210 4.6540 14.0770 0.0780

23 21 H12 H1 E 5.4150 6.1820 13.6050 0.0780

24 21 H13 H1 E 4.0350 5.4370 13.9180 0.0780

25 20 C10 CA M 5.3910 3.7120 11.8050 -0.0450

26 25 H10 H4 E 5.8130 3.1760 12.4660 0.1280

27 25 C11 CA M 5.3350 3.2690 10.5140 -0.2430

28 27 H9 HA E 5.7340 2.4400 10.2800 0.1270

COBTIWh

1 0 C1 CA M 4.6420 8.6960 1.4640 -0.1330

2 1 H1 HA E 4.2150 8.7300 0.6620 0.1430

3 1 C2 CA M 4.4850 9.7760 2.3240 -0.0230

4 3 C4 CT M 3.5280 10.8710 1.9690 0.0730

5 4 H2 H1 E 3.3680 11.5200 2.7600 0.0440

6 4 H3 H1 E 3.7900 11.3730 1.1480 0.0440

7 4 O1 OS M 2.2560 10.2430 1.7040 -0.3040

8 7 C9 CT M 1.2100 11.1800 1.4770 0.1160

9 8 H9 H1 E 1.4560 11.8100 0.7370 0.0260

10 8 H10 H1 E 1.1220 11.7080 2.3040 0.0260

11 8 C11 CT M -0.0320 10.4180 1.1440 0.1030

12 11 H13 H1 E 0.1600 9.7590 0.3370 0.0290

13 11 H14 H1 E -0.7190 11.0010 0.9040 0.0290

14 11 O3 OS M -0.4810 9.6190 2.2440 -0.3330

15 14 C13 CT M -1.5420 10.2280 2.9720 0.1210

16 15 H17 H1 E -1.2200 11.0630 3.3440 0.0330

17 15 H18 H1 E -2.3520 10.3690 2.3430 0.0330

18 15 C15 CT M -1.8980 9.3660 4.1250 0.0720

19 18 H21 H1 E -2.4990 9.7970 4.6820 0.0400

20 18 H22 H1 E -1.1510 9.0470 4.6670 0.0400

21 18 O5 OS M -2.4870 8.1490 3.6680 -0.3620

22 21 C17 CT M -2.6510 7.2170 4.7190 0.1780

23 22 H25 H1 E -1.8040 6.8930 5.0630 0.0150

24 22 H26 H1 E -3.1130 7.6110 5.4280 0.0150

25 22 C19 CT M -3.4340 6.0230 4.2690 0.0780

26 25 H29 H1 E -4.2670 6.3020 3.8740 0.0310

27 25 H30 H1 E -3.6110 5.4590 5.0450 0.0310

28 25 O7 OS M -2.6830 5.3030 3.3010 -0.3670

29 28 C21 CT M -3.3070 4.0810 2.9080 0.0530

30 29 H33 H1 E -3.3170 3.4530 3.7010 0.0780

31 29 H34 H1 E -4.2100 4.2660 2.6420 0.0780

32 29 C22 CT M -2.5530 3.5390 1.7510 0.0530

33 32 H35 H1 E -2.5240 4.2070 1.0350 0.0780

34 32 H36 H1 E -2.9360 2.7360 1.4420 0.0780

35 32 O8 OS M -1.1710 3.2850 2.0540 -0.3670

36 35 C20 CT M -0.9160 1.9970 2.6010 0.0780

37 36 H31 H1 E -1.5100 1.8310 3.3550 0.0310

38 36 H32 H1 E -1.0830 1.3290 1.8680 0.0310

39 36 C18 CT M 0.5110 1.9720 3.0540 0.1780

40 39 H27 H1 E 0.6600 2.7510 3.6730 0.0150

41 39 H28 H1 E 0.6730 1.1180 3.5880 0.0150

42 39 O6 OS M 1.3550 2.0560 1.9180 -0.3620

43 42 C16 CT M 2.7200 2.1800 2.2760 0.0720

44 43 H23 H1 E 2.8380 2.9520 2.8400 0.0400

45 43 H24 H1 E 2.9900 1.3580 2.7740 0.0400

46 43 C14 CT M 3.5650 2.3020 1.0530 0.1210

47 46 H19 H1 E 3.3710 1.5360 0.4090 0.0330

48 46 H20 H1 E 4.5150 2.2200 1.3270 0.0330

49 46 O4 OS M 3.3510 3.5810 0.4600 -0.3330

50 49 C12 CT M 4.3750 3.9250 -0.4780 0.1030

51 50 H15 H1 E 4.3380 3.3480 -1.2490 0.0290

52 50 H16 H1 E 5.2490 3.7470 -0.0350 0.0290

53 50 C10 CT M 4.1760 5.3490 -0.8800 0.1160

54 53 H11 H1 E 3.2820 5.4560 -1.3430 0.0260

55 53 H12 H1 E 4.8210 5.6180 -1.4940 0.0260

56 53 O2 OS M 4.1830 6.2590 0.2250 -0.3040

57 56 C6 CT M 5.4450 6.3950 0.8780 0.0730

58 57 H5 H1 E 5.6950 5.5170 1.3750 0.0440

59 57 H6 H1 E 6.0910 6.6120 0.1950 0.0440

60 57 C3 CA M 5.3590 7.5740 1.8180 -0.0230

61 60 C7 CA M 5.9920 7.5670 3.0520 -0.1220

62 61 H7 HA E 6.4440 6.8290 3.2710 0.1350

63 61 C8 CA M 5.9240 8.6500 3.8890 -0.1690

64 63 H8 HA E 6.3700 8.6520 4.7710 0.1370

65 63 C5 CA M 5.1580 9.7440 3.5370 -0.1220

66 65 H4 HA E 5.0670 10.4130 4.0940 0.1350

COBTIWg

1 0 N1 NH M 1.6840 7.2100 1.2600 -0.9020

2 1 H1 H E 1.6010 8.0160 1.4920 0.4650

3 1 H2 H E 2.4670 6.9600 0.9710 0.4650

4 1 C1 C2 M 0.7660 6.3190 1.5970 0.9160

5 4 N3 NH B -0.3850 6.7190 2.1140 -0.9020

6 5 H5 H E -0.9720 6.1310 2.3930 0.4650

7 5 H6 H E -0.5570 7.5700 2.2240 0.4650

8 4 N2 NH M 1.0020 5.0370 1.4030 -0.9020

9 8 H3 H E 1.7450 4.7320 1.0570 0.4650

10 8 H4 H E 0.3850 4.4430 1.5430 0.4650

COXLEG10h

1 0 O2 OH M 0.9700 12.1050 5.3370 -0.5970

2 1 H2 HO E 0.0230 12.1510 5.1810 0.4320

3 1 C8 CA M 1.4420 10.8450 5.4460 0.3200

4 3 C7 CA M 0.7510 9.6670 5.2810 -0.0900

5 4 C1 CA M -0.5450 8.9250 4.9840 -0.0900

6 5 C2 CA M -1.8720 8.9430 4.6400 0.3200

7 6 O1 OH S -2.6560 10.0150 4.3970 -0.5970

8 7 H1 HO E -2.2380 10.8150 4.5690 0.4320

9 6 C3 CA M -2.4920 7.6740 4.5130 -0.2040

10 9 H3 HA E -3.4680 7.6400 4.2830 0.1730

11 9 C4 CA M -1.8190 6.4930 4.6940 -0.2470

12 11 H4 HA E -2.3170 5.6240 4.5960 0.1820

13 11 C5 CA M -0.4490 6.4650 5.0160 -0.1480

14 13 H5 HA E 0.0560 5.6050 5.1350 0.1570

15 13 C6 CA M 0.1390 7.6920 5.1520 0.0230

16 15 C12 CA M 1.4160 8.4190 5.4330 0.0230

17 16 C11 CA M 2.7400 8.2870 5.7200 -0.1480

18 17 H8 HA E 3.1870 7.3960 5.8060 0.1570

19 17 C10 CA M 3.4400 9.5020 5.8870 -0.2470

20 19 H7 HA E 4.4190 9.4820 6.0990 0.1820

21 19 C9 CA M 2.8110 10.7150 5.7630 -0.2040

22 21 H6 HA E 3.3490 11.5480 5.9060 0.1730

COXLEG10g

1 0 O1 O M -1.5760 12.3070 4.9140 -0.5650

2 1 C1 C M -2.1550 13.4250 4.8630 0.6410

3 2 C2 CA M -1.4380 14.6470 5.0270 -0.4430

4 3 H1 HA E -0.4470 14.6200 5.1740 0.1790

5 3 C4 CA M -2.0810 15.8220 4.9840 0.3530

6 5 C6 CT 3 -1.4880 17.1670 5.1520 -0.3670

7 6 H3 HC E -1.7220 17.7430 4.3900 0.1230

8 6 H4 HC E -0.5200 17.1260 5.2680 0.1230

9 6 H5 HC E -1.8650 17.5620 5.9190 0.1230

10 5 O2 OS M -3.4180 15.8920 4.7730 -0.2570

11 10 C5 CA M -4.1380 14.7510 4.5970 0.3530

12 11 C7 CT 3 -5.5670 15.0460 4.3490 -0.3670

13 12 H6 HC E -5.9270 15.6210 5.0550 0.1230

14 12 H7 HC E -5.6450 15.4730 3.5120 0.1230

15 12 H8 HC E -6.0890 14.2110 4.3300 0.1230

16 11 C3 CA M -3.5540 13.5450 4.6360 -0.4430

17 16 H2 HA E -4.1080 12.7280 4.5030 0.1790

COXQELh

1 0 C1 CT M 0.7200 3.1720 24.8180 0.0690

2 1 H1 H2 E 0.6170 3.8780 25.5300 0.0490

3 1 H2 H2 E 1.5110 2.5790 25.1180 0.0490

4 1 N1 NT M -0.4670 2.3440 24.6710 -0.2480

5 4 C3 CT B -1.6680 3.1800 24.6980 0.1450

6 5 H5 H2 E -1.6030 3.8960 23.9760 0.0270

7 5 H6 H2 E -1.7340 3.6610 25.5930 0.0270

8 4 C2 CT M -0.5430 1.3140 25.7100 -0.0050

9 8 H3 H1 E 0.2830 0.7730 25.6440 0.0440

10 8 H4 H1 E -0.5460 1.7230 26.6040 0.0440

11 8 C4 CT M -1.7970 0.4840 25.4940 -0.2670

12 11 H7 HC E -1.8640 -0.2080 26.1820 0.1140

13 11 H8 HC E -1.7500 0.0170 24.6080 0.1140

14 11 C7 CT M -3.0260 1.3810 25.5350 -0.0020

15 14 C10 CT 3 -4.3400 0.6340 25.3390 -0.2670

16 15 C13 CT B -5.5090 1.6210 25.3010 -0.0050

17 16 H23 H1 E -6.3550 1.1760 25.1350 0.0440

18 16 H24 H1 E -5.5820 2.1020 26.1870 0.0440

19 15 H18 HC E -4.2760 0.0740 24.5020 0.1140

20 15 H19 HC E -4.4830 -0.0120 26.0920 0.1140

21 14 H13 H1 E -3.0500 1.8100 26.4430 0.0970

22 14 N3 NT M -2.8830 2.4070 24.4910 -0.2540

23 22 C8 CT M -4.0280 3.3320 24.5130 0.1450

24 23 H14 H2 E -4.1050 3.7990 25.4400 0.0270

25 23 H15 H2 E -3.8760 4.0100 23.8280 0.0270

26 23 N5 NT M -5.3170 2.6860 24.3030 -0.2480

27 26 C14 CT M -5.5050 2.1660 22.9450 0.0690

28 27 H25 H2 E -4.8590 1.4260 22.7210 0.0490

29 27 H26 H2 E -6.4430 1.7990 22.9260 0.0490

30 27 N7 NT M -5.3200 3.2090 21.9500 -0.2480

31 30 C19 CT B -5.3710 2.6190 20.6220 0.1450

32 31 H35 H2 E -4.7700 1.8660 20.5890 0.0270

33 31 H36 H2 E -6.3320 2.2480 20.4040 0.0270

34 30 C18 CT M -6.2920 4.3040 22.0400 -0.0050

35 34 H33 H1 E -7.2490 3.9720 21.9500 0.0440

36 34 H34 H1 E -6.2450 4.6930 22.9500 0.0440

37 34 C20 CT M -5.9760 5.3410 20.9740 -0.2670

38 37 H37 HC E -5.1140 5.7940 21.2030 0.1140

39 37 H38 HC E -6.6630 6.0340 20.9550 0.1140

40 37 C23 CT M -5.9150 4.7170 19.5860 -0.0020

41 40 C26 CT 3 -5.4720 5.6970 18.5030 -0.2670

42 41 H48 HC E -6.1240 6.4180 18.4190 0.1140

43 41 H49 HC E -4.6430 6.1120 18.7840 0.1140

44 41 C29 CT B -5.3050 4.9920 17.1640 -0.0050

45 44 H53 H1 E -4.9090 5.6040 16.5160 0.0440

46 44 H54 H1 E -6.1870 4.6600 16.7940 0.0440

47 40 H43 H1 E -6.8160 4.3770 19.3430 0.0970

48 40 N9 NT M -4.9660 3.5950 19.6240 -0.2540

49 48 C24 CT M -4.8780 2.9510 18.3230 0.1450

50 49 H44 H2 E -5.8270 2.6070 18.0180 0.0270

51 49 H45 H2 E -4.2930 2.1800 18.3610 0.0270

52 49 N11 NT M -4.3680 3.8810 17.3250 -0.2480

53 52 C30 CT M -4.1040 3.1720 16.0820 0.0690

54 53 H55 H2 E -4.0010 3.8780 15.3700 0.0490

55 53 H56 H2 E -4.8950 2.5790 15.7820 0.0490

56 53 N12 NT M -2.9180 2.3440 16.2290 -0.2480

57 56 C32 CT 3 -2.8410 1.3140 15.1900 -0.0050

58 57 C31 CT 3 -1.5870 0.4840 15.4060 -0.2670

59 58 C27 CT B -0.3580 1.3810 15.3650 -0.0020

60 59 C25 CT 3 0.9560 0.6340 15.5610 -0.2670

61 60 C21 CT B 2.1250 1.6210 15.5990 -0.0050

62 61 H39 H1 E 2.9710 1.1760 15.7660 0.0440

63 61 H40 H1 E 2.1980 2.1020 14.7130 0.0440

64 60 H46 HC E 0.8920 0.0740 16.3980 0.1140

65 60 H47 HC E 1.0990 -0.0120 14.8090 0.1140

66 59 H50 H1 E -0.3340 1.8100 14.4570 0.0970

67 58 H57 HC E -1.5210 -0.2080 14.7190 0.1140

68 58 H58 HC E -1.6340 0.0170 16.2920 0.1140

69 57 H59 H1 E -3.6670 0.7730 15.2560 0.0440

70 57 H60 H1 E -2.8380 1.7230 14.2960 0.0440

71 56 C28 CT M -1.7170 3.1800 16.2020 0.1450

72 71 H51 H2 E -1.7810 3.8960 16.9250 0.0270

73 71 H52 H2 E -1.6500 3.6610 15.3080 0.0270

74 71 N10 NT M -0.5010 2.4070 16.4090 -0.2540

75 74 C22 CT M 0.6440 3.3320 16.3870 0.1450

76 75 H41 H2 E 0.7200 3.7990 15.4600 0.0270

77 75 H42 H2 E 0.4920 4.0100 17.0720 0.0270

78 75 N8 NT M 1.9320 2.6860 16.5970 -0.2480

79 78 C17 CT M 2.1200 2.1660 17.9550 0.0690

80 79 H31 H2 E 1.4740 1.4260 18.1790 0.0490

81 79 H32 H2 E 3.0590 1.7990 17.9740 0.0490

82 79 N6 NT M 1.9360 3.2090 18.9500 -0.2480

83 82 C12 CT M 1.9870 2.6190 20.2780 0.1450

84 83 H21 H2 E 1.3860 1.8660 20.3110 0.0270

85 83 H22 H2 E 2.9480 2.2480 20.4960 0.0270

86 83 N4 NT M 1.5820 3.5950 21.2760 -0.2540

87 86 C6 CT M 1.4940 2.9510 22.5770 0.1450

88 87 H11 H2 E 2.4430 2.6070 22.8820 0.0270

89 87 H12 H2 E 0.9090 2.1800 22.5390 0.0270

90 87 N2 NT M 0.9840 3.8810 23.5750 -0.2480

91 90 C5 CT M 1.9200 4.9920 23.7360 -0.0050

92 91 H9 H1 E 1.5250 5.6040 24.3850 0.0440

93 91 H10 H1 E 2.8030 4.6600 24.1070 0.0440

94 91 C9 CT M 2.0870 5.6970 22.3970 -0.2670

95 94 H16 HC E 2.7400 6.4180 22.4810 0.1140

96 94 H17 HC E 1.2590 6.1120 22.1160 0.1140

97 94 C11 CT M 2.5310 4.7170 21.3140 -0.0020

98 97 H20 H1 E 3.4310 4.3770 21.5570 0.0970

99 97 C15 CT M 2.5920 5.3410 19.9270 -0.2670

100 99 H27 HC E 1.7300 5.7940 19.6980 0.1140

101 99 H28 HC E 3.2790 6.0340 19.9460 0.1140

102 99 C16 CT M 2.9070 4.3040 18.8600 -0.0050

103 102 H29 H1 E 3.8640 3.9720 18.9500 0.0440

104 102 H30 H1 E 2.8610 4.6930 17.9500 0.0440

COXQELg

1 0 O1 OS M -0.5560 0.3080 19.6160 -0.4240

2 1 C1 CT M -1.3540 -0.8690 19.7820 0.1010

3 2 H1 H1 E -0.7370 -1.6810 19.6510 0.0560

4 2 H2 H1 E -2.0290 -0.9340 19.0980 0.0560

5 2 C3 CT M -2.0310 -0.8690 21.1180 0.1010

6 5 H5 H1 E -2.6470 -1.6810 21.2490 0.0560

7 5 H6 H1 E -1.3550 -0.9340 21.8030 0.0560

8 5 O2 OS M -2.8280 0.3080 21.2840 -0.4240

9 8 C4 CT M -2.0070 1.4740 21.1290 0.1010

10 9 H7 H1 E -2.5960 2.2750 21.2870 0.0560

11 9 H8 H1 E -1.2790 1.4480 21.7750 0.0560

12 9 C2 CT M -1.3770 1.4740 19.7710 0.1010

13 12 H3 H1 E -0.7880 2.2750 19.6130 0.0560

14 12 H4 H1 E -2.1050 1.4480 19.1250 0.0560

COYBOHh

1 0 C1 CT M -0.6530 2.9740 -1.9830 0.0830

2 1 H1 H1 E -0.9040 3.4800 -2.9150 0.0370

3 1 H2 H1 E 0.1330 3.5330 -1.4760 0.0370

4 1 C2 CT M -1.8640 2.8970 -1.1100 0.0830

5 4 H3 H1 E -2.2830 3.8950 -0.9820 0.0370

6 4 H4 H1 E -2.6030 2.2470 -1.5770 0.0370

7 4 O2 OS M -1.5100 2.3680 0.1570 -0.3150

8 7 C3 CT M -2.6100 2.3030 1.0410 0.0830

9 8 H5 H1 E -3.3900 1.6730 0.6140 0.0370

10 8 H6 H1 E -3.0100 3.3040 1.2030 0.0370

11 8 C4 CT M -2.1510 1.7280 2.3430 0.0830

12 11 H7 H1 E -2.9670 1.7730 3.0640 0.0370

13 11 H8 H1 E -1.3020 2.3030 2.7140 0.0370

14 11 O3 OS M -1.7620 0.3770 2.1600 -0.3150

15 14 C5 CT M -1.3500 -0.2340 3.3650 0.0830

16 15 H9 H1 E -2.1680 -0.2200 4.0850 0.0370

17 15 H10 H1 E -0.5020 0.3100 3.7800 0.0370

18 15 C6 CT M -0.9510 -1.6470 3.0800 0.0830

19 18 H11 H1 E -1.7660 -2.1540 2.5630 0.0370

20 18 H12 H1 E -0.7370 -2.1600 4.0180 0.0370

21 18 O4 OS M 0.2050 -1.6620 2.2600 -0.3150

22 21 C7 CT M 0.6530 -2.9740 1.9830 0.0830

23 22 H13 H1 E -0.1330 -3.5330 1.4760 0.0370

24 22 H14 H1 E 0.9040 -3.4800 2.9150 0.0370

25 22 C8 CT M 1.8640 -2.8970 1.1100 0.0830

26 25 H15 H1 E 2.6080 -2.2510 1.5780 0.0370

27 25 H16 H1 E 2.2770 -3.8970 0.9790 0.0370

28 25 O5 OS M 1.5100 -2.3680 -0.1570 -0.3150

29 28 C9 CT M 2.6100 -2.3030 -1.0410 0.0830

30 29 H17 H1 E 3.0100 -3.3040 -1.2030 0.0370

31 29 H18 H1 E 3.3900 -1.6730 -0.6140 0.0370

32 29 C10 CT M 2.1510 -1.7280 -2.3430 0.0830

33 32 H19 H1 E 1.2990 -2.3010 -2.7090 0.0370

34 32 H20 H1 E 2.9640 -1.7760 -3.0680 0.0370

35 32 O6 OS M 1.7620 -0.3770 -2.1600 -0.3150

36 35 C11 CT M 1.3500 0.2340 -3.3650 0.0830

37 36 H21 H1 E 0.5020 -0.3100 -3.7800 0.0370

38 36 H22 H1 E 2.1680 0.2200 -4.0850 0.0370

39 36 C12 CT M 0.9510 1.6470 -3.0800 0.0830

40 39 H23 H1 E 1.7660 2.1580 -2.5670 0.0370

41 39 H24 H1 E 0.7330 2.1560 -4.0190 0.0370

42 39 O1 OS M -0.2050 1.6620 -2.2600 -0.3150

COYBOHg

1 0 C17 CA M 1.1440 5.8110 3.5100 -0.0590

2 1 C12 CA B 2.2950 5.2820 3.9470 -0.1380

3 2 C6 CA S 2.8050 4.1160 3.3960 -0.1530

4 3 H5 HA E 3.7250 3.6850 3.7910 0.1660

5 2 H11 HA E 2.8400 5.7780 4.7500 0.1630

6 1 H16 HA E 0.7610 6.7350 3.9430 0.1560

7 1 C11 CA M 0.4520 5.1720 2.5050 -0.1380

8 7 H10 HA E -0.5020 5.5760 2.1650 0.1630

9 7 C5 CA M 0.9550 4.0240 1.9240 -0.1530

10 9 H4 HA E 0.4050 3.5280 1.1240 0.1660

11 9 C1 CA M 2.1590 3.5080 2.3630 0.0200

12 11 P1 P M 2.8300 2.0330 1.5880 0.3450

13 12 C2 CT 3 1.5140 1.0870 0.8500 -0.3650

14 13 H1 HC E 1.9270 0.1940 0.3820 0.1500

15 13 H2 HC E 1.0100 1.6920 0.0970 0.1500

16 13 H3 HC E 0.8000 0.7960 1.6200 0.1500

17 12 C4 CA S 4.0140 2.5170 0.3270 0.0200

18 17 C9 CA B 4.1180 1.7750 -0.8340 -0.1530

19 18 C15 CA B 5.0450 2.1350 -1.7920 -0.1380

20 19 C19 CA B 5.8720 3.2200 -1.5970 -0.0590

21 20 C16 CA B 5.7380 3.9480 -0.4800 -0.1380

22 21 C10 CA S 4.7960 3.6200 0.4830 -0.1530

23 22 H9 HA E 4.6820 4.2470 1.3680 0.1660

24 21 H15 HA E 6.3810 4.8150 -0.3260 0.1630

25 20 H18 HA E 6.6250 3.4820 -2.3410 0.1560

26 19 H14 HA E 5.1240 1.5550 -2.7110 0.1630

27 18 H8 HA E 3.4730 0.9110 -0.9920 0.1660

28 12 C3 CA M 3.6660 1.0430 2.8320 0.0200

29 28 C7 CA M 2.9960 -0.0020 3.4390 -0.1530

30 29 H6 HA E 1.9710 -0.2370 3.1520 0.1660

31 29 C13 CA M 3.6330 -0.7470 4.4120 -0.1380

32 31 H12 HA E 3.1090 -1.5770 4.8860 0.1630

33 31 C18 CA M 4.9250 -0.4520 4.7890 -0.0590

34 33 H17 HA E 5.4130 -1.0210 5.5800 0.1560

35 33 C14 CA M 5.5730 0.5450 4.1700 -0.1380

36 35 H13 HA E 6.5990 0.7760 4.4570 0.1630

37 35 C8 CA M 4.9620 1.2890 3.1720 -0.1530

38 37 H7 HA E 5.5190 2.0730 2.6590 0.1660

CRAMCA10h

1 0 O1 OS M 4.0400 13.1570 3.9920 -0.3080

2 1 C1 CT M 4.6840 12.0220 4.5660 0.0700

3 2 H1 H1 E 4.6810 12.0930 5.4880 0.0420

4 2 H2 H1 E 4.1710 11.3090 4.3930 0.0420

5 2 C3 CT M 6.0810 11.9220 4.0220 0.0700

6 5 H5 H1 E 6.6780 11.2930 4.6000 0.0420

7 5 H6 H1 E 6.6340 12.7960 4.0500 0.0420

8 5 O2 OS M 6.0030 11.4750 2.6570 -0.3080

9 8 C5 CT M 7.2870 11.1710 2.1150 0.0700

10 9 H9 H1 E 7.8850 11.8980 2.3070 0.0420

11 9 H10 H1 E 7.7450 10.4250 2.4980 0.0420

12 9 C7 CT M 7.1450 10.9660 0.6330 0.0700

13 12 H13 H1 E 6.3220 10.4180 0.4250 0.0420

14 12 H14 H1 E 7.8960 10.5730 0.2790 0.0420

15 12 O4 OS M 6.8810 12.2310 0.0430 -0.3080

16 15 C9 CT M 6.7900 12.1830 -1.3840 0.0700

17 16 H17 H1 E 7.5790 11.9670 -1.6460 0.0420

18 16 H18 H1 E 6.1340 11.5240 -1.6510 0.0420

19 16 C11 CT M 6.3980 13.5500 -1.8750 0.0700

20 19 H21 H1 E 6.5240 13.5500 -2.8280 0.0420

21 19 H22 H1 E 6.9550 14.1900 -1.4730 0.0420

22 19 O6 OS M 5.0140 13.7630 -1.5790 -0.3080

23 22 C12 CT M 4.5030 15.0110 -2.0650 0.0700

24 23 H23 H1 E 5.0550 15.7500 -1.7210 0.0420

25 23 H24 H1 E 4.6650 15.0940 -2.9840 0.0420

26 23 C10 CT M 3.0490 15.1150 -1.7180 0.0700

27 26 H19 H1 E 2.5870 14.3810 -1.9300 0.0420

28 26 H20 H1 E 2.6380 15.8570 -2.2070 0.0420

29 26 O5 OS M 2.9070 15.2950 -0.3000 -0.3080

30 29 C8 CT M 1.5340 15.1800 0.0990 0.0700

31 30 H15 H1 E 0.9950 15.7740 -0.3480 0.0420

32 30 H16 H1 E 1.1870 14.3240 -0.1240 0.0420

33 30 C6 CT M 1.4400 15.3940 1.5940 0.0700

34 33 H11 H1 E 0.5500 15.5440 1.7910 0.0420

35 33 H12 H1 E 1.9390 16.2120 1.8180 0.0420

36 33 O3 OS M 2.0320 14.2620 2.2510 -0.3080

37 36 C4 CT M 2.0110 14.4090 3.6740 0.0700

38 37 H7 H1 E 2.4680 15.2380 3.9310 0.0420

39 37 H8 H1 E 1.2190 14.4630 3.9330 0.0420

40 37 C2 CT M 2.6410 13.1800 4.2900 0.0700

41 40 H3 H1 E 2.2670 12.3810 3.9310 0.0420

42 40 H4 H1 E 2.4830 13.1850 5.2230 0.0420

CRAMCA10g

1 0 N1 N3 M 4.5320 13.4630 1.1910 -0.0920

2 1 H1 H E 3.7200 13.4160 1.3140 0.3250

3 1 H2 H E 4.6520 13.4810 0.3230 0.3250

4 1 H3 H E 4.9700 12.8080 1.4510 0.3250

5 1 N2 NT M 5.2220 14.6690 1.6070 -0.7190

6 5 H4 H E 5.1180 14.6830 2.4620 0.4180

7 5 H5 H E 4.8400 15.2060 1.2000 0.4180

CRAMCB10h

1 0 O1 OS M 0.7080 4.7400 13.6960 -0.3080

2 1 C1 CT M 0.7040 5.1110 15.0740 0.0700

3 2 H1 H1 E -0.1660 5.5100 15.2980 0.0420

4 2 H2 H1 E 1.4190 5.8100 15.1830 0.0420

5 2 C3 CT M 1.0560 3.9160 15.9100 0.0700

6 5 H5 H1 E 1.0760 4.1370 16.7910 0.0420

7 5 H6 H1 E 1.9500 3.6060 15.6670 0.0420

8 5 O2 OS M 0.0550 2.9090 15.7460 -0.3080

9 8 C5 CT M 0.2900 1.7990 16.6210 0.0700

10 9 H9 H1 E 0.2380 2.0940 17.5370 0.0420

11 9 H10 H1 E 1.1640 1.4160 16.4840 0.0420

12 9 C7 CT M -0.7380 0.7390 16.3760 0.0700

13 12 H13 H1 E -1.6580 1.1100 16.5410 0.0420

14 12 H14 H1 E -0.6000 0.0330 17.0560 0.0420

15 12 O4 OS M -0.6220 0.2920 15.0260 -0.3080

16 15 C9 CT M -1.4360 -0.8590 14.7700 0.0700

17 16 H17 H1 E -2.3460 -0.6970 15.0310 0.0420

18 16 H18 H1 E -1.1350 -1.6200 15.3360 0.0420

19 16 C11 CT M -1.3970 -1.1590 13.3050 0.0700

20 19 H21 H1 E -1.9850 -1.9940 13.1870 0.0420

21 19 H22 H1 E -0.5300 -1.2750 13.0080 0.0420

22 19 O6 OS M -1.9680 -0.0580 12.6000 -0.3080

23 22 C12 CT M -2.1470 -0.3400 11.2130 0.0700

24 23 H23 H1 E -2.8160 -0.9870 11.1550 0.0420

25 23 H24 H1 E -1.3260 -0.5880 10.8490 0.0420

26 23 C10 CT M -2.7190 0.8730 10.5540 0.0700

27 26 H19 H1 E -3.5450 1.1390 11.0180 0.0420

28 26 H20 H1 E -2.9580 0.7060 9.6910 0.0420

29 26 O5 OS M -1.7560 1.9230 10.6240 -0.3080

30 29 C8 CT M -2.1660 3.1010 9.9310 0.0700

31 30 H15 H1 E -3.0350 3.5080 10.3440 0.0420

32 30 H16 H1 E -2.3330 2.8610 9.0490 0.0420

33 30 C6 CT M -1.0660 4.1070 9.9770 0.0700

34 33 H11 H1 E -1.3240 4.8930 9.5130 0.0420

35 33 H12 H1 E -0.2460 3.7280 9.5690 0.0420

36 33 O3 OS M -0.7990 4.4480 11.3420 -0.3080

37 36 C4 CT M 0.2650 5.3950 11.4410 0.0700

38 37 H7 H1 E 0.1660 6.1430 10.8620 0.0420

39 37 H8 H1 E 1.1210 4.9610 11.2010 0.0420

40 37 C2 CT M 0.3690 5.8450 12.8680 0.0700

41 40 H3 H1 E -0.4680 6.2550 13.1850 0.0420

42 40 H4 H1 E 1.0690 6.5000 12.8960 0.0420

CRAMCB10g

1 0 N1 N3 M -0.1310 2.1220 12.9710 -0.2270

2 1 H1 H E -0.0170 2.6420 13.6010 0.3780

3 1 H2 H E -0.5950 1.6710 13.3680 0.3780

4 1 H3 H E -0.4490 2.4320 12.3840 0.3780

5 1 O1 OH M 1.0930 1.5510 12.5490 -0.3700

6 5 H4 HO E 1.4240 1.0300 13.1700 0.4640

CRAMCC10h

1 0 O1 OS M 18.5510 -0.1440 7.8440 -0.3080

2 1 C1 CT M 18.7100 -1.5410 8.1360 0.0700

3 2 H1 H1 E 19.1410 -1.6670 8.9970 0.0420

4 2 H2 H1 E 17.4860 -2.1140 8.1570 0.0420

5 2 C3 CT M 19.6730 -2.1320 7.1920 0.0700

6 5 H5 H1 E 19.9650 -3.0160 7.5260 0.0420

7 5 H6 H1 E 20.5430 -1.5730 7.1630 0.0420

8 5 O2 OS M 19.0930 -2.1160 5.8930 -0.3080

9 8 C5 CT M 19.9140 -2.7460 4.9190 0.0700

10 9 H9 H1 E 20.8500 -2.2430 4.9280 0.0420

11 9 H10 H1 E 20.1010 -3.5840 5.1960 0.0420

12 9 C7 CT M 19.2420 -2.6800 3.6160 0.0700

13 12 H13 H1 E 18.3940 -2.8880 3.4960 0.0420

14 12 H14 H1 E 19.9030 -3.0590 2.9420 0.0420

15 12 O4 OS M 19.1830 -1.3080 3.1710 -0.3080

16 15 C9 CT M 18.6050 -1.1580 1.8720 0.0700

17 16 H17 H1 E 19.0640 -1.6930 1.2230 0.0420

18 16 H18 H1 E 17.6490 -1.6160 1.8340 0.0420

19 16 C11 CT M 18.5510 0.3030 1.5070 0.0700

20 19 H21 H1 E 18.2970 0.5240 0.4200 0.0420

21 19 H22 H1 E 19.4520 0.6270 1.4710 0.0420

22 19 O6 OS M 17.7380 0.9670 2.4470 -0.3080

23 22 C12 CT M 17.5660 2.3640 2.1300 0.0700

24 23 H23 H1 E 18.5470 2.8270 2.0440 0.0420

25 23 H24 H1 E 17.2300 2.4750 1.2610 0.0420

26 23 C10 CT M 16.6580 2.9830 3.1180 0.0700

27 26 H19 H1 E 15.7550 2.4240 3.0950 0.0420

28 26 H20 H1 E 16.3130 3.9620 2.8650 0.0420

29 26 O5 OS M 17.2550 2.9650 4.3880 -0.3080

30 29 C8 CT M 16.4400 3.5910 5.3930 0.0700

31 30 H15 H1 E 16.2410 4.5120 5.1200 0.0420

32 30 H16 H1 E 15.4280 3.0940 5.3680 0.0420

33 30 C6 CT M 17.0990 3.4860 6.7050 0.0700

34 33 H11 H1 E 16.5670 3.9700 7.4120 0.0420

35 33 H12 H1 E 18.1090 3.8760 6.6480 0.0420

36 33 O3 OS M 17.0880 2.1280 7.1060 -0.3080

37 36 C4 CT M 17.6550 1.9570 8.4070 0.0700

38 37 H7 H1 E 18.7030 2.3550 8.2710 0.0420

39 37 H8 H1 E 17.2680 2.3630 9.1120 0.0420

40 37 C2 CT M 17.6620 0.5290 8.7390 0.0700

41 40 H3 H1 E 16.6730 0.0600 8.5960 0.0420

42 40 H4 H1 E 17.9370 0.3610 9.8190 0.0420

CRAMCC10g

1 0 N1 N3 M 18.9150 0.7560 5.1460 -0.2120

2 1 H1 H E 18.8730 0.2840 4.6230 0.3010

3 1 H2 H E 18.7200 0.5410 5.7880 0.3010

4 1 H3 H E 18.4140 1.4010 5.1390 0.3010

5 1 C1 CT M 20.2460 1.3290 5.2190 -0.0370

6 5 H4 HP E 20.3390 1.6330 4.5850 0.1150

7 5 H5 HP E 21.0630 0.7130 5.5210 0.1150

8 5 H6 HP E 20.2550 1.9940 5.9980 0.1150

CUDXUUh

1 0 C38 CT M 1.5550 2.0590 -3.1660 0.0610

2 1 H36 H1 E 2.3350 2.6610 -3.5480 0.0780

3 1 H37 H1 E 1.8030 1.0200 -3.3200 0.0780

4 1 O5 OS M 1.3410 2.3270 -1.7920 -0.2880

5 4 C33 CA M 2.4470 2.2290 -0.9720 0.2050

6 5 C29 CA B 3.5850 1.5310 -1.2730 -0.2000

7 6 C26 CA S 4.6390 1.4970 -0.3550 -0.1490

8 7 H23 HA E 5.5010 0.9090 -0.5870 0.1430

9 6 H25 HA E 3.6680 1.0140 -2.2070 0.1390

10 5 C28 CA M 2.3300 2.9200 0.2290 -0.2000

11 10 H24 HA E 1.4370 3.4680 0.4510 0.1390

12 10 C25 CA M 3.4120 2.8900 1.1230 -0.1490

13 12 H22 HA E 3.3200 3.4300 2.0340 0.1430

14 12 C21 CA M 4.5710 2.1740 0.8510 0.0100

15 14 C18 CT M 5.7220 2.2010 1.8680 0.1410

16 15 C22 CT 3 6.8960 1.3010 1.4140 -0.2060

17 16 H16 HC E 7.5910 1.2320 2.2010 0.0570

18 16 H17 HC E 7.4060 1.7170 0.5630 0.0570

19 16 H18 HC E 6.5600 0.3320 1.1560 0.0570

20 15 C23 CT 3 5.2040 1.6670 3.2150 -0.2060

21 20 H19 HC E 4.3580 2.2310 3.5730 0.0570

22 20 H20 HC E 5.9770 1.7140 3.9260 0.0570

23 20 H21 HC E 4.8960 0.6720 3.1030 0.0570

24 15 C15 CA M 6.2600 3.6370 1.9790 0.0100

25 24 C13 CA B 6.5790 4.3480 0.8400 -0.1490

26 25 C11 CA S 7.1440 5.6120 0.9030 -0.2000

27 26 H9 HA E 7.3650 6.1430 0.0250 0.1390

28 25 H11 HA E 6.3820 3.9100 -0.1050 0.1430

29 24 C12 CA M 6.4930 4.2680 3.2130 -0.1490

30 29 H10 HA E 6.2200 3.7580 4.1110 0.1430

31 29 C10 CA M 7.0660 5.5440 3.2860 -0.2000

32 31 H8 HA E 7.2280 6.0140 4.2100 0.1390

33 31 C8 CA M 7.4100 6.1890 2.1360 0.2050

34 33 O1 OS M 8.0190 7.4280 2.0800 -0.2880

35 34 C4 CT M 8.4040 8.0440 3.3190 0.0610

36 35 H2 H1 E 8.8130 7.3030 3.9810 0.0780

37 35 H3 H1 E 9.1730 8.7740 3.1030 0.0780

38 35 C2 CA M 7.2320 8.7360 3.9790 -0.0020

39 38 C5 CA S 6.0430 9.0170 3.2880 -0.0960

40 39 H4 HA E 5.9420 8.6840 2.2690 0.1640

41 38 C1 CA M 7.3830 9.1570 5.2810 -0.1630

42 41 H1 HA E 8.2740 8.8990 5.8170 0.1660

43 41 C3 CA M 6.3490 9.8700 5.8660 -0.0210

44 43 N1 NO B 6.5110 10.4050 7.2550 0.7770

45 44 O2 O E 7.5920 10.1820 7.7940 -0.4560

46 44 O3 O E 5.6130 11.0430 7.7450 -0.4560

47 43 C6 CA M 5.1520 10.1860 5.2250 -0.1630

48 47 H5 HA E 4.3590 10.7530 5.7120 0.1660

49 47 C7 CA M 5.0280 9.7320 3.8940 -0.0020

50 49 C9 CT M 3.7360 10.0560 3.1660 0.0610

51 50 H6 H1 E 2.9560 9.4540 3.5480 0.0780

52 50 H7 H1 E 3.4880 11.0960 3.3200 0.0780

53 50 O4 OS M 3.9500 9.7890 1.7920 -0.2880

54 53 C14 CA M 2.8440 9.8860 0.9720 0.2050

55 54 C17 CA B 1.7060 10.5840 1.2730 -0.2000

56 55 H13 HA E 1.6220 11.1020 2.2070 0.1390

57 55 C20 CA S 0.6520 10.6180 0.3550 -0.1490

58 57 H15 HA E -0.2110 11.2060 0.5870 0.1430

59 54 C16 CA M 2.9600 9.1960 -0.2290 -0.2000

60 59 H12 HA E 3.8530 8.6470 -0.4510 0.1390

61 59 C19 CA M 1.8780 9.2250 -1.1230 -0.1490

62 61 H14 HA E 1.9710 8.6850 -2.0340 0.1430

63 61 C24 CA M 0.7200 9.9410 -0.8510 0.0100

64 63 C27 CT M -0.4310 9.9150 -1.8680 0.1410

65 64 C30 CT 3 -1.6060 10.8150 -1.4140 -0.2060

66 65 H26 HC E -2.3000 10.8830 -2.2010 0.0570

67 65 H27 HC E -2.1150 10.3980 -0.5630 0.0570

68 65 H28 HC E -1.2700 11.7830 -1.1560 0.0570

69 64 C31 CT 3 0.0860 10.4480 -3.2150 -0.2060

70 69 H29 HC E 0.9330 9.8840 -3.5730 0.0570

71 69 H30 HC E -0.6870 10.4010 -3.9260 0.0570

72 69 H31 HC E 0.3950 11.4440 -3.1030 0.0570

73 64 C32 CA M -0.9700 8.4790 -1.9790 0.0100

74 73 C35 CA B -1.2020 7.8480 -3.2130 -0.1490

75 74 H33 HA E -0.9300 8.3570 -4.1110 0.1430

76 74 C37 CA S -1.7750 6.5710 -3.2860 -0.2000

77 76 H35 HA E -1.9380 6.1010 -4.2100 0.1390

78 73 C34 CA M -1.2890 7.7670 -0.8400 -0.1490

79 78 H32 HA E -1.0910 8.2050 0.1050 0.1430

80 78 C36 CA M -1.8540 6.5030 -0.9030 -0.2000

81 80 H34 HA E -2.0750 5.9730 -0.0250 0.1390

82 80 C39 CA M -2.1190 5.9260 -2.1360 0.2050

83 82 O6 OS M -2.7290 4.6870 -2.0800 -0.2880

84 83 C43 CT M -3.1140 4.0710 -3.3190 0.0610

85 84 H40 H1 E -3.5220 4.8130 -3.9810 0.0780

86 84 H41 H1 E -3.8820 3.3410 -3.1030 0.0780

87 84 C44 CA M -1.9410 3.3790 -3.9790 -0.0020

88 87 C46 CA S -2.0920 2.9580 -5.2810 -0.1630

89 88 H42 HA E -2.9840 3.2160 -5.8170 0.1660

90 87 C41 CA M -0.7530 3.0980 -3.2880 -0.0960

91 90 H38 HA E -0.6520 3.4310 -2.2690 0.1640

92 90 C40 CA M 0.2620 2.3830 -3.8940 -0.0020

93 92 C42 CA M 0.1380 1.9290 -5.2250 -0.1630

94 93 H39 HA E 0.9320 1.3630 -5.7120 0.1660

95 93 C45 CA M -1.0590 2.2460 -5.8660 -0.0210

96 95 N2 NO M -1.2200 1.7100 -7.2550 0.7770

97 96 O8 O E -0.3230 1.0730 -7.7450 -0.4560

98 96 O7 O M -2.3010 1.9340 -7.7940 -0.4560

CUDXUUg

1 0 C1 CA M 2.8350 6.4380 2.4520 -0.1390

2 1 H1 HA E 3.6390 6.9420 1.9230 0.1390

3 1 C2 CA M 1.5970 6.3240 1.8670 -0.1390

4 3 H2 HA E 1.4400 6.7720 0.8840 0.1390

5 3 C4 CA M 0.5970 5.6600 2.4840 -0.1390

6 5 H4 HA E -0.3440 5.5430 1.9910 0.1390

7 5 C6 CA M 0.8180 5.1260 3.7300 -0.1390

8 7 H6 HA E 0.0140 4.6210 4.2590 0.1390

9 7 C5 CA M 2.0560 5.2400 4.3150 -0.1390

10 9 H5 HA E 2.2130 4.7910 5.2980 0.1390

11 9 C3 CA M 3.0560 5.9030 3.6980 -0.1390

12 11 H3 HA E 3.9970 6.0200 4.1910 0.1390

CYCBOBh

1 0 O6 O M 6.3170 0.5100 2.7270 -0.5560

2 1 C8 C M 6.4380 1.6320 3.0950 0.7930

3 2 O4 OS M 6.6330 1.9910 4.3770 -0.3910

4 3 C5 CT M 6.4740 0.9780 5.3970 0.1830

5 4 C6 CT 3 7.2520 1.4550 6.6020 -0.0940

6 5 C4 CT 3 6.5890 2.6660 7.2380 0.0450

7 6 C2 CT B 5.1140 2.3820 7.4930 -0.1360

8 7 H2 HC E 4.6370 3.1980 7.9030 0.0580

9 7 H3 HC E 5.0210 1.6980 8.2370 0.0580

10 6 H4 HC E 6.7520 3.4580 6.7360 0.0080

11 6 H5 HC E 7.0950 2.8750 8.1700 0.0080

12 5 H7 HC E 8.3040 1.6880 6.3140 0.0620

13 5 H8 HC E 7.3020 0.6880 7.3810 0.0620

14 4 H6 H2 E 6.8620 0.1150 5.0690 0.1450

15 4 O1 OS M 5.1170 0.7890 5.6470 -0.3870

16 15 C1 CT M 4.4660 1.9400 6.1880 0.0810

17 16 H1 H1 E 4.5390 2.6670 5.5360 0.0630

18 16 C3 C M 3.0120 1.5820 6.3350 0.7930

19 18 O3 O E 2.5290 0.5210 6.1150 -0.5560

20 18 O2 OS M 2.3280 2.6710 6.7250 -0.3910

21 20 C7 CT M 0.8770 2.5410 6.7660 0.1830

22 21 O5 OS E 0.3850 2.6170 5.4480 -0.3870

23 21 H9 H2 E 0.9500 1.6350 7.1580 0.1450

24 21 C9 CT M 0.3540 3.6030 7.6740 -0.0940

25 24 H10 HC E 0.8120 3.5830 8.5260 0.0620

26 24 H11 HC E -0.6850 3.4580 7.8250 0.0620

27 24 C11 CT M 0.4890 4.9930 7.0630 0.0450

28 27 H13 HC E 1.4140 5.2500 7.0470 0.0080

29 27 H14 HC E -0.0460 5.6360 7.7030 0.0080

30 27 C14 CT M -0.0960 4.9930 5.6500 -0.1360

31 30 H18 HC E 0.0350 5.8440 5.1800 0.0580

32 30 H19 HC E -1.0650 4.8230 5.6130 0.0580

33 30 C12 CT M 0.5700 3.8920 4.8430 0.0810

34 33 H15 H1 E 1.5020 4.0730 4.7910 0.0630

35 33 C15 C M 0.0520 3.7680 3.4300 0.7930

36 35 O9 O E -0.4840 2.8210 2.9520 -0.5560

37 35 O8 OS M 0.3200 4.9040 2.7510 -0.3910

38 37 C19 CT M 0.1040 4.8510 1.3280 0.1830

39 38 O11 OS E 1.1400 4.1360 0.7240 -0.3870

40 38 H25 H2 E -0.6810 4.2710 1.1120 0.1450

41 38 C21 CT M -0.0170 6.2740 0.8170 -0.0940

42 41 H26 HC E -0.7570 6.7710 1.2890 0.0620

43 41 H27 HC E -0.3500 6.1880 -0.1000 0.0620

44 41 C23 CT M 1.3220 6.9930 0.8670 0.0450

45 44 H29 HC E 1.5720 7.1360 1.8010 0.0080

46 44 H30 HC E 1.2450 7.9170 0.4340 0.0080

47 44 C24 CT M 2.3920 6.1600 0.1950 -0.1360

48 47 H31 HC E 3.2730 6.5730 0.2450 0.0580

49 47 H32 HC E 2.1760 6.0210 -0.7340 0.0580

50 47 C22 CT M 2.4080 4.7760 0.8390 0.0810

51 50 H28 H1 E 2.5690 4.8960 1.7010 0.0630

52 50 C20 C M 3.4260 3.8560 0.2250 0.7930

53 52 O12 O E 3.2110 2.8800 -0.4070 -0.5560

54 52 O10 OS M 4.6590 4.3210 0.5240 -0.3910

55 54 C17 CT M 5.7930 3.5250 0.0730 0.1830

56 55 H22 H2 E 5.5140 3.0830 -0.7890 0.1450

57 55 O7 OS M 5.9800 2.4570 0.9510 -0.3870

58 57 C10 CT M 6.3840 2.8730 2.2510 0.0810

59 58 H12 H1 E 5.6860 3.4790 2.5680 0.0630

60 58 C13 CT M 7.6950 3.6280 2.2030 -0.1360

61 60 H16 HC E 7.9820 3.8750 3.0570 0.0580

62 60 H17 HC E 8.4240 3.0830 1.7780 0.0580

63 60 C16 CT M 7.5250 4.8430 1.3160 0.0450

64 63 H20 HC E 6.9370 5.4590 1.6780 0.0080

65 63 H21 HC E 8.3840 5.3230 1.1450 0.0080

66 63 C18 CT M 6.9750 4.4380 -0.0400 -0.0940

67 66 H23 HC E 6.6510 5.2920 -0.5560 0.0620

68 66 H24 HC E 7.7100 3.9900 -0.5780 0.0620

CYCBOBg

1 0 N1 N1 M 3.2460 2.8370 3.2860 -0.4910

2 1 C1 CZ M 2.7740 1.8500 2.9960 0.3850

3 2 C2 CT M 2.1280 0.6020 2.6740 -0.2420

4 3 H1 HC E 1.8590 0.0730 3.4460 0.1160

5 3 H2 HC E 2.6440 0.1250 1.9900 0.1160

6 3 H3 HC E 1.2830 0.7400 2.1340 0.1160

CYCBOF11h

1 0 O2 OS M 2.4180 2.2720 -1.1480 -0.3670

2 1 C3 C M 1.8680 2.9710 -0.1160 0.7730

3 2 O3 O E 1.3120 3.9880 -0.2770 -0.5610

4 2 C1 CT M 2.0760 2.2230 1.1600 0.0780

5 4 O1 OS E 1.3540 2.8970 2.1770 -0.3670

6 4 H1 H1 E 1.5280 1.2250 0.9460 0.0610

7 4 C2 CT M 3.5720 2.1010 1.4590 -0.1030

8 7 H2 HC E 3.7990 2.9230 1.4080 0.0490

9 7 H3 HC E 3.9600 1.4760 0.7520 0.0490

10 7 C4 CT M 3.7470 1.4790 2.8680 0.0450

11 10 H4 HC E 4.7540 1.4620 3.0820 0.0060

12 10 H5 HC E 3.5480 0.6940 2.8640 0.0060

13 10 C6 CT M 2.9310 2.2350 3.8850 -0.0880

14 13 H7 HC E 2.9550 1.7270 4.7570 0.0600

15 13 H8 HC E 3.3570 3.1150 3.9310 0.0600

16 13 C5 CT M 1.5010 2.3640 3.4680 0.1440

17 16 H6 H2 E 0.8940 2.9680 4.0530 0.1570

18 16 O4 OS M 0.9270 1.0280 3.5040 -0.3670

19 18 C8 C M -0.3740 0.9080 3.8730 0.7730

20 19 O6 O E -1.0640 1.8380 4.1550 -0.5610

21 19 C10 CT M -0.7270 -0.5210 3.9140 0.0780

22 21 O7 OS E -2.1510 -0.6020 4.1280 -0.3670

23 21 H12 H1 E -0.5630 -0.6790 3.0340 0.0610

24 21 C13 CT M 0.0010 -1.2610 5.0360 -0.1030

25 24 H16 HC E -0.2810 -0.7680 6.0430 0.0490

26 24 H17 HC E 0.9950 -1.1520 4.6590 0.0490

27 24 C16 CT M -0.4270 -2.7080 5.1670 0.0450

28 27 H20 HC E 0.1110 -3.1590 6.1160 0.0060

29 27 H21 HC E -0.1410 -3.2920 4.4900 0.0060

30 27 C18 CT M -1.9430 -2.7430 5.3030 -0.0880

31 30 H23 HC E -2.1610 -2.2440 6.1160 0.0600

32 30 H24 HC E -2.4620 -3.8240 5.1930 0.0600

33 30 C17 CT M -2.6330 -1.9250 4.2540 0.1440

34 33 H22 H2 E -3.5580 -1.8010 4.4900 0.1570

35 33 O10 OS M -2.4420 -2.6100 3.0170 -0.3670

36 35 C20 C M -3.4750 -2.6930 2.1890 0.7730

37 36 O12 O E -4.5130 -2.1720 2.3350 -0.5610

38 36 C22 CT M -3.0690 -3.5710 1.0000 0.0780

39 38 O13 OS E -4.1840 -3.5570 0.0680 -0.3670

40 38 H28 H1 E -2.2110 -3.1300 0.4370 0.0610

41 38 C25 CT M -2.6990 -4.9390 1.4200 -0.1030

42 41 H32 HC E -3.3060 -5.5370 2.0870 0.0490

43 41 H33 HC E -1.8890 -5.0050 2.2570 0.0490

44 41 C28 CT M -2.4120 -5.7520 0.1240 0.0450

45 44 H36 HC E -2.1000 -6.5700 0.2670 0.0060

46 44 H37 HC E -1.4370 -5.6550 -0.3400 0.0060

47 44 C30 CT M -3.5900 -5.7520 -0.7810 -0.0880

48 47 H39 HC E -4.3220 -6.4080 -0.4610 0.0600

49 47 H40 HC E -3.4570 -6.1270 -1.6020 0.0600

50 47 C29 CT M -4.0130 -4.3290 -1.0680 0.1440

51 50 H38 H2 E -4.7840 -4.1780 -1.6260 0.1570

52 50 O14 OS M -2.9800 -3.7600 -1.8860 -0.3670

53 52 C27 C M -3.3040 -2.7610 -2.6990 0.7730

54 53 O15 O E -4.3790 -2.2010 -2.7740 -0.5610

55 53 C24 CT M -2.1200 -2.4050 -3.5820 0.0780

56 55 C26 CT 3 -1.7700 -3.5710 -4.4990 -0.1030

57 56 C23 CT 3 -0.6450 -3.1300 -5.4460 0.0450

58 57 C21 CT B -1.0310 -1.8460 -6.1590 -0.0880

59 58 H26 HC E -1.7290 -1.9640 -6.6740 0.0600

60 58 H27 HC E -0.3020 -1.4620 -6.6250 0.0600

61 57 H29 HC E -0.4520 -4.0450 -6.1160 0.0060

62 57 H30 HC E 0.2110 -2.9090 -4.9990 0.0060

63 56 H34 HC E -2.5020 -3.7650 -4.9990 0.0490

64 56 H35 HC E -1.6180 -4.4440 -4.0530 0.0490

65 55 H31 H1 E -1.3470 -2.2000 -2.8880 0.0610

66 55 O11 OS M -2.4320 -1.2180 -4.2540 -0.3670

67 66 C19 CT M -1.4760 -0.7740 -5.1670 0.1440

68 67 H25 H2 E -1.8590 -0.0300 -5.6540 0.1570

69 67 O8 OS M -0.2810 -0.3960 -4.4360 -0.3670

70 69 C15 C M -0.1270 0.9090 -4.1450 0.7730

71 70 O9 O E -0.8890 1.7510 -4.4460 -0.5610

72 70 C12 CT M 1.1520 1.1040 -3.3730 0.0780

73 72 H15 H1 E 1.0450 0.6640 -2.3300 0.0610

74 72 O5 OS M 1.2590 2.4890 -3.1230 -0.3670

75 74 C7 CT M 2.4460 2.8460 -2.4530 0.1440

76 75 H9 H2 E 2.4620 3.6610 -2.3050 0.1570

77 75 C9 CT M 3.7130 2.3710 -3.1430 -0.0880

78 77 H10 HC E 3.9090 2.8490 -4.2950 0.0600

79 77 H11 HC E 4.3920 2.4800 -2.4270 0.0600

80 77 C11 CT M 3.6350 0.8950 -3.4120 0.0450

81 80 H13 HC E 4.4620 0.6640 -4.0530 0.0060

82 80 H14 HC E 3.7290 0.4720 -2.4270 0.0060

83 80 C14 CT M 2.3840 0.5650 -4.1860 -0.1030

84 83 H18 HC E 2.3820 1.0190 -5.1930 0.0490

85 83 H19 HC E 2.4320 -0.5460 -4.4410 0.0490

CYCBOF11g

1 0 N1 N1 M -1.1930 0.1710 -0.7090 -0.4910

2 1 C1 CZ M -2.2860 0.1990 -0.7280 0.3850

3 2 C2 CT M -3.5010 0.4830 -1.0170 -0.2420

4 3 H1 HC E -4.2710 -0.1180 -0.2180 0.1160

5 3 H2 HC E -3.6980 1.5500 -0.8980 0.1160

6 3 H3 HC E -3.6780 0.2660 -1.9660 0.1160

DENFORh

1 0 C28 CT M 11.1810 7.5250 13.2740 -0.0700

2 1 H29 H1 E 11.1110 7.8290 14.3190 0.0560

3 1 H30 H1 E 10.8850 8.3560 12.6330 0.0560

4 1 H31 H1 E 12.2080 7.2390 13.0470 0.0560

5 1 N1 NT M 10.2930 6.3840 13.0360 -0.3090

6 5 C21 CT 3 8.9260 6.7520 13.3870 -0.0210

7 6 C15 CT B 7.9560 5.6290 13.1030 -0.0970

8 7 H15 HC E 8.2260 4.7730 13.7210 0.0550

9 7 H16 HC E 6.9520 5.9610 13.3670 0.0550

10 6 H23 H1 E 8.8870 6.9930 14.4490 0.0580

11 6 H24 H1 E 8.6340 7.6270 12.8070 0.0580

12 5 C20 CT M 10.3710 5.9610 11.6450 -0.0210

13 12 H21 H1 E 10.1150 6.8060 11.0070 0.0580

14 12 H22 H1 E 11.3910 5.6430 11.4300 0.0580

15 12 C14 CT M 9.4290 4.8180 11.3400 -0.0970

16 15 H13 HC E 9.5210 4.5570 10.2860 0.0550

17 15 H14 HC E 9.7060 3.9580 11.9500 0.0550

18 15 C11 CT M 7.9570 5.1900 11.6360 0.0300

19 18 C10 CA M 7.4310 6.2500 10.6730 0.0020

20 19 C8 CA B 6.5290 7.2220 11.0910 -0.1510

21 20 C5 CA S 5.9500 8.1080 10.2110 -0.2310

22 21 H6 HA E 5.2420 8.8510 10.5770 0.1350

23 20 H10 HA E 6.2720 7.2840 12.1480 0.1550

24 19 C7 CA M 7.7370 6.2380 9.3290 -0.1510

25 24 H9 HA E 8.4540 5.5040 8.9610 0.1550

26 24 C4 CA M 7.1670 7.1250 8.4250 -0.2310

27 26 H5 HA E 7.4340 7.0820 7.3690 0.1350

28 26 C2 CA M 6.2600 8.0590 8.8770 0.2740

29 28 O1 OS M 5.6090 8.9760 8.0820 -0.3220

30 29 C1 CT M 5.7420 8.8510 6.6700 0.1180

31 30 H1 H1 E 5.4250 7.8570 6.3540 0.0290

32 30 H2 H1 E 6.7800 9.0130 6.3800 0.0290

33 30 C3 CT M 4.8670 9.8930 6.0350 0.0150

34 33 H3 HC E 5.1570 10.8710 6.4190 0.0100

35 33 H4 HC E 3.8310 9.6920 6.3080 0.0100

36 33 C6 CT M 4.9750 9.9160 4.5270 0.0150

37 36 H7 HC E 6.0310 9.8600 4.2640 0.0100

38 36 H8 HC E 4.5680 10.8620 4.1700 0.0100

39 36 C9 CT M 4.2600 8.8010 3.8200 0.1180

40 39 H11 H1 E 4.6180 7.8390 4.1860 0.0290

41 39 H12 H1 E 4.4330 8.8720 2.7460 0.0290

42 39 O2 OS M 2.8720 8.9380 4.0960 -0.3220

43 42 C12 CA M 2.0380 7.8990 3.7700 0.2740

44 43 C17 CA B 0.8340 7.8570 4.4350 -0.2310

45 44 C23 CA S -0.0570 6.8330 4.1900 -0.1510

46 45 H26 HA E -1.0040 6.8180 4.7290 0.1550

47 44 H18 HA E 0.5850 8.6340 5.1580 0.1350

48 43 C16 CA M 2.3260 6.9310 2.8420 -0.2310

49 48 H17 HA E 3.2660 6.9630 2.2920 0.1350

50 48 C22 CA M 1.4170 5.9120 2.6080 -0.1510

51 50 H25 HA E 1.6610 5.1510 1.8670 0.1550

52 50 C26 CA M 0.2120 5.8230 3.2800 0.0020

53 52 C29 CT M -0.7120 4.5910 3.0980 0.0300

54 53 C31 CT 3 -2.1830 4.9640 3.3950 -0.0970

55 54 C36 CT 3 -3.1260 3.8200 3.0890 -0.0210

56 55 N2 NT B -3.0480 3.3970 1.6980 -0.3090

57 56 C37 CT 3 -1.6810 3.0290 1.3470 -0.0210

58 57 C32 CT B -0.7110 4.1520 1.6310 -0.0970

59 58 H34 HC E 0.2930 3.8170 1.3700 0.0550

60 58 H35 HC E -0.9770 5.0080 1.0110 0.0550

61 57 H42 H1 E -1.3900 2.1550 1.9290 0.0580

62 57 H43 H1 E -1.6400 2.7850 0.2850 0.0580

63 56 C43 CT 3 -3.9350 2.2560 1.4600 -0.0700

64 63 H50 H1 E -3.8640 1.9530 0.4150 0.0560

65 63 H51 H1 E -4.9620 2.5410 1.6870 0.0560

66 63 H52 H1 E -3.6390 1.4250 2.1000 0.0560

67 55 H40 H1 E -4.1460 4.1400 3.3010 0.0580

68 55 H41 H1 E -2.8730 2.9750 3.7290 0.0580

69 54 H32 HC E -2.4600 5.8220 2.7830 0.0550

70 54 H33 HC E -2.2760 5.2270 4.4490 0.0550

71 53 C30 CA M -0.1850 3.5310 4.0610 0.0020

72 71 C35 CA B -0.4910 3.5430 5.4050 -0.1510

73 72 C40 CA S 0.0790 2.6570 6.3100 -0.2310

74 73 H47 HA E -0.1870 2.7000 7.3660 0.1350

75 72 H39 HA E -1.2080 4.2770 5.7720 0.1550

76 71 C34 CA M 0.7160 2.5590 3.6430 -0.1510

77 76 H38 HA E 0.9730 2.4960 2.5850 0.1550

78 76 C39 CA M 1.2960 1.6730 4.5240 -0.2310

79 78 H46 HA E 2.0050 0.9300 4.1590 0.1350

80 78 C42 CA M 0.9860 1.7220 5.8570 0.2740

81 80 O4 OS M 1.6370 0.8050 6.6530 -0.3220

82 81 C44 CT M 1.5030 0.9300 8.0650 0.1180

83 82 H53 H1 E 1.8190 1.9240 8.3820 0.0290

84 82 H54 H1 E 0.4650 0.7670 8.3550 0.0290

85 82 C41 CT M 2.3790 -0.1120 8.6990 0.0150

86 85 H48 HC E 2.0890 -1.0900 8.3150 0.0100

87 85 H49 HC E 3.4150 0.0890 8.4260 0.0100

88 85 C38 CT M 2.2700 -0.1340 10.2080 0.0150

89 88 H44 HC E 1.2140 -0.0760 10.4710 0.0100

90 88 H45 HC E 2.6760 -1.0800 10.5650 0.0100

91 88 C33 CT M 2.9860 0.9800 10.9140 0.1180

92 91 H36 H1 E 2.6280 1.9420 10.5480 0.0290

93 91 H37 H1 E 2.8130 0.9100 11.9880 0.0290

94 91 O3 OS M 4.3730 0.8430 10.6390 -0.3220

95 94 C27 CA M 5.2070 1.8820 10.9640 0.2740

96 95 C24 CA M 4.9200 2.8500 11.8920 -0.2310

97 96 H27 HA E 3.9810 2.8170 12.4440 0.1350

98 96 C18 CA M 5.8290 3.8690 12.1270 -0.1510

99 98 H19 HA E 5.5860 4.6290 12.8700 0.1550

100 98 C13 CA M 7.0330 3.9580 11.4550 0.0020

101 100 C19 CA M 7.3030 2.9480 10.5440 -0.1510

102 101 H20 HA E 8.2490 2.9650 10.0030 0.1550

103 101 C25 CA M 6.4120 1.9240 10.3000 -0.2310

104 103 H28 HA E 6.6630 1.1460 9.5790 0.1350

DENFORg

1 0 C1 CA M 3.5840 6.0030 8.1520 -0.1210

2 1 H1 HA E 3.5560 6.8960 8.7760 0.1210

3 1 C2 CA M 2.9040 5.9710 6.9770 -0.1210

4 3 H2 HA E 2.3210 6.8370 6.6640 0.1210

5 3 C4 CA M 2.9470 4.8630 6.1870 -0.1210

6 5 H4 HA E 2.4080 4.8470 5.2400 0.1210

7 5 C6 CA M 3.6610 3.7780 6.5820 -0.1210

8 7 H6 HA E 3.6880 2.8840 5.9580 0.1210

9 7 C5 CA M 4.3410 3.8100 7.7580 -0.1210

10 9 H5 HA E 4.9230 2.9430 8.0710 0.1210

11 9 C3 CA M 4.2990 4.9180 8.5470 -0.1210

12 11 H3 HA E 4.8380 4.9340 9.4940 0.1210

DERFUBh

1 0 C5 CA M 10.0260 3.1680 1.1710 -0.1390

2 1 H5 HA E 9.5260 2.4340 0.5400 0.1150

3 1 C2 CA M 11.3470 3.0270 1.4440 -0.1390

4 3 H1 HA E 11.8860 2.1920 0.9960 0.1150

5 3 C1 CA M 12.0250 3.8760 2.2480 0.0020

6 5 C3 CA M 11.3490 5.0060 2.7870 -0.1390

7 6 H2 HA E 11.8640 5.7200 3.4290 0.1150

8 6 C6 CA M 9.9770 5.1680 2.4580 -0.1390

9 8 H6 HA E 9.4370 6.0430 2.8200 0.1150

10 8 C7 CA M 9.3120 4.2220 1.6770 0.0020

11 10 C10 CT M 7.8440 4.3950 1.4190 0.0280

12 11 H12 H1 E 7.4520 3.4710 0.9940 0.0380

13 11 H13 H1 E 7.7110 5.2050 0.7020 0.0380

14 11 N2 NT M 7.0850 4.7140 2.6280 -0.1310

15 14 C15 CT 3 5.6760 5.0980 2.2780 -0.2320

16 15 H18 H1 E 5.1200 5.3120 3.1910 0.0820

17 15 H19 H1 E 5.1970 4.2770 1.7450 0.0820

18 15 H20 H1 E 5.6880 5.9840 1.6440 0.0820

19 14 C14 CT M 7.0760 3.5460 3.4640 0.0280

20 19 H16 H1 E 8.0840 3.1330 3.5110 0.0380

21 19 H17 H1 E 6.4010 2.8060 3.0340 0.0380

22 19 C18 CA M 6.6000 3.8920 4.9060 0.0020

23 22 C21 CA B 7.1370 5.0110 5.5540 -0.1390

24 23 C24 CA S 6.7310 5.2650 6.8520 -0.1390

25 24 H28 HA E 7.1040 6.1670 7.3370 0.1150

26 23 H24 HA E 7.8520 5.6620 5.0510 0.1150

27 22 C20 CA M 5.7300 3.0540 5.5720 -0.1390

28 27 H23 HA E 5.3150 2.1820 5.0660 0.1150

29 27 C23 CA M 5.3730 3.3360 6.9330 -0.1390

30 29 H27 HA E 4.6960 2.6710 7.4690 0.1150

31 29 C25 CA M 5.8910 4.4550 7.5640 0.0020

32 31 C28 CT M 5.5220 4.6710 9.0330 0.0280

33 32 H34 H1 E 4.9990 3.7860 9.3950 0.0380

34 32 H35 H1 E 4.8600 5.5340 9.1050 0.0380

35 32 N4 NT M 6.6920 4.9030 9.8800 -0.1310

36 35 C33 CT 3 6.2620 5.4170 11.1740 -0.2320

37 36 H40 H1 E 7.1340 5.5900 11.8040 0.0820

38 36 H41 H1 E 5.6050 4.6910 11.6530 0.0820

39 36 H42 H1 E 5.7250 6.3550 11.0320 0.0820

40 35 C32 CT M 7.4720 3.6170 10.0040 0.0280

41 40 H38 H1 E 7.4650 3.1050 9.0420 0.0380

42 40 H39 H1 E 6.9960 2.9840 10.7530 0.0380

43 40 C36 CA M 8.9330 3.8760 10.4220 0.0020

44 43 C35 CA B 9.6090 5.0060 9.8820 -0.1390

45 44 C31 CA S 10.9810 5.1680 10.2120 -0.1390

46 45 H37 HA E 11.5210 6.0430 9.8510 0.1150

47 44 H44 HA E 9.0930 5.7200 9.2390 0.1150

48 43 C34 CA M 9.6100 3.0270 11.2250 -0.1390

49 48 H43 HA E 9.0710 2.1930 11.6740 0.1150

50 48 C30 CA M 10.9310 3.1680 11.4990 -0.1390

51 50 H36 HA E 11.4310 2.4320 12.1290 0.1150

52 50 C29 CA M 11.6460 4.2220 10.9920 0.0020

53 52 C26 CT M 13.1130 4.3950 11.2500 0.0280

54 53 H29 H1 E 13.2480 5.2020 11.9700 0.0380

55 53 H30 H1 E 13.5030 3.4680 11.6710 0.0380

56 53 N3 NT M 13.8730 4.7140 10.0420 -0.1310

57 56 C27 CT 3 15.2810 5.0980 10.3910 -0.2320

58 57 H31 H1 E 15.2690 5.9720 11.0420 0.0820

59 57 H32 H1 E 15.8300 5.3320 9.4790 0.0820

60 57 H33 H1 E 15.7670 4.2690 10.9060 0.0820

61 56 C22 CT M 13.8820 3.5460 9.2060 0.0280

62 61 H25 H1 E 14.5550 2.8040 9.6360 0.0380

63 61 H26 H1 E 12.8740 3.1340 9.1590 0.0380

64 61 C19 CA M 14.3580 3.8920 7.7640 0.0020

65 64 C17 CA B 13.8210 5.0110 7.1150 -0.1390

66 65 C13 CA S 14.2270 5.2650 5.8180 -0.1390

67 66 H15 HA E 13.8540 6.1670 5.3330 0.1150

68 65 H22 HA E 13.1050 5.6610 7.6170 0.1150

69 64 C16 CA M 15.2270 3.0540 7.0970 -0.1390

70 69 H21 HA E 15.6400 2.1800 7.6010 0.1150

71 69 C12 CA M 15.5840 3.3360 5.7370 -0.1390

72 71 H14 HA E 16.2610 2.6710 5.2010 0.1150

73 71 C11 CA M 15.0670 4.4550 5.1060 0.0020

74 73 C9 CT M 15.4350 4.6710 3.6360 0.0280

75 74 H10 H1 E 16.0940 5.5360 3.5650 0.0380

76 74 H11 H1 E 15.9610 3.7880 3.2730 0.0380

77 74 N1 NT M 14.2650 4.9030 2.7900 -0.1310

78 77 C8 CT 3 14.6960 5.4170 1.4950 -0.2320

79 78 H7 H1 E 15.2530 6.3430 1.6390 0.0820

80 78 H8 H1 E 13.8230 5.6110 0.8720 0.0820

81 78 H9 H1 E 15.3350 4.6810 1.0070 0.0820

82 77 C4 CT M 13.4850 3.6170 2.6660 0.0280

83 82 H3 H1 E 13.9610 2.9860 1.9160 0.0380

84 82 H4 H1 E 13.4930 3.1030 3.6270 0.0380

DERFUBg

1 0 N1 N1 M 10.4790 1.2970 6.3350 -0.4910

2 1 C1 CZ M 10.4790 2.4870 6.3350 0.3850

3 2 C2 CT M 10.4790 3.9460 6.3350 -0.2420

4 3 H1 HC E 10.4790 4.3100 7.3620 0.1160

5 3 H2 HC E 9.5900 4.3100 5.8200 0.1160

6 3 H3 HC E 11.3700 4.3090 5.8220 0.1160

DESHEOh

1 0 O5 OH M 9.7430 17.8280 6.9930 -0.5250

2 1 H5 HO E 10.5450 17.0900 6.9710 0.3990

3 1 C7 CA M 8.6710 17.3130 6.4940 0.4290

4 3 C6 CA S 7.4820 18.0580 6.4040 -0.3690

5 4 H2 HA E 7.4780 19.0980 6.7300 0.1950

6 3 C4 CA M 8.5440 15.9840 6.0310 -0.3690

7 6 H1 HA E 9.3990 15.3100 6.0840 0.1950

8 6 C1 CA M 7.3530 15.5330 5.5150 0.2800

9 8 N1 NC M 6.2130 16.2400 5.4480 -0.5810

10 9 C2 CA M 6.3180 17.4980 5.9100 0.2800

11 10 C5 C M 5.0990 18.3710 5.8890 0.7660

12 11 O4 O E 5.1110 19.4960 6.1990 -0.6060

13 11 O3 OS M 4.0010 17.7450 5.5330 -0.3810

14 13 C9 CT M 2.7480 18.4830 5.5700 0.1360

15 14 H6 H1 E 2.9370 19.4990 5.9170 0.0580

16 14 H7 H1 E 2.0570 17.9890 6.2530 0.0580

17 14 C11 CT M 2.1580 18.5280 4.2270 0.0630

18 17 H10 H1 E 2.8720 18.9750 3.5350 0.0570

19 17 H11 H1 E 1.2510 19.1320 4.2520 0.0570

20 17 O7 OS M 1.8380 17.2230 3.7890 -0.3780

21 20 C13 CT M 1.2180 17.2090 2.5150 0.1020

22 21 H14 H1 E 1.8480 17.7330 1.7970 0.0500

23 21 H15 H1 E 0.2480 17.7020 2.5760 0.0500

24 21 C15 CT M 1.0340 15.8030 2.0810 0.0930

25 24 H18 H1 E 0.5210 15.7910 1.1200 0.0510

26 24 H19 H1 E 0.4300 15.2770 2.8200 0.0510

27 24 O8 OS M 2.2610 15.1550 1.9500 -0.3910

28 27 C14 CT M 2.1400 13.8410 1.4860 0.0930

29 28 H16 H1 E 1.6210 13.2390 2.2320 0.0510

30 28 H17 H1 E 1.5720 13.8360 0.5560 0.0510

31 28 C12 CT M 3.4790 13.2760 1.2460 0.1020

32 31 H12 H1 E 3.3830 12.3050 0.7610 0.0500

33 31 H13 H1 E 4.0480 13.9480 0.6030 0.0500

34 31 O6 OS M 4.1660 13.1190 2.5090 -0.3780

35 34 C10 CT M 5.4840 12.6500 2.3180 0.0630

36 35 H8 H1 E 5.4500 11.6610 1.8610 0.0570

37 35 H9 H1 E 6.0170 13.3350 1.6590 0.0570

38 35 C8 CT M 6.1960 12.5640 3.6090 0.1360

39 38 H3 H1 E 5.6240 11.9500 4.3050 0.0580

40 38 H4 H1 E 7.1810 12.1220 3.4570 0.0580

41 38 O2 OS M 6.3390 13.9070 4.1430 -0.3810

42 41 C3 C M 7.3300 14.1110 5.0150 0.7660

43 42 O1 O M 8.1160 13.2710 5.3330 -0.6060

DESHEOg

1 0 N1 N3 M 3.6950 15.1300 4.5030 -0.3210

2 1 H1 H E 3.4210 15.7350 4.0060 0.3140

3 1 H2 H E 4.5030 15.3550 4.8020 0.3140

4 1 H3 H E 3.7910 14.5480 3.8850 0.3140

5 1 C1 CT M 2.8370 14.6550 5.6050 0.0130

6 5 H4 HP E 1.9660 14.4490 5.2550 0.1200

7 5 H5 HP E 2.7620 15.3500 6.2620 0.1200

8 5 C2 CA M 3.3980 13.4380 6.2500 -0.0140

9 8 C3 CA M 2.7390 12.2340 6.1680 -0.1590

10 9 H6 HA E 1.9330 12.1720 5.6510 0.1570

11 9 C5 CA M 3.2150 11.1230 6.8120 -0.1450

12 11 H8 HA E 2.7410 10.2900 6.7450 0.1720

13 11 C7 CA M 4.3670 11.1940 7.5500 -0.0670

14 13 H10 HA E 4.6940 10.4140 8.0070 0.1610

15 13 C6 CA M 5.0490 12.3740 7.6360 -0.1450

16 15 H9 HA E 5.8650 12.4260 8.1370 0.1720

17 15 C4 CA M 4.5520 13.5010 6.9920 -0.1590

18 17 H7 HA E 5.0230 14.3340 7.0680 0.1570

DIZTIPh

1 0 C10 CA M 4.2990 7.8920 11.7940 -0.2370

2 1 C7 CA B 3.3220 7.0830 11.2640 -0.2020

3 2 C5 CA S 3.2020 6.9300 9.9010 -0.1810

4 3 H5 HA E 2.4460 6.2730 9.4970 0.1240

5 2 H7 HA E 2.6420 6.5620 11.9210 0.1690

6 1 H10 HA E 4.3890 8.0000 12.8650 0.1640

7 1 C6 CA M 5.1680 8.5710 10.9540 -0.0810

8 7 H6 HA E 5.9320 9.2140 11.3640 0.1290

9 7 C4 CA M 5.0480 8.4160 9.6090 0.1110

10 9 O3 OS E 5.8460 9.0810 8.6820 -0.2300

11 9 C2 CA M 4.0520 7.6190 9.0470 0.2230

12 11 O1 OS M 4.0170 7.5740 7.6900 -0.2790

13 12 C1 CT M 2.8340 7.0810 7.0670 0.2310

14 13 H1 H1 E 2.7010 6.0340 7.2990 0.0030

15 13 H2 H1 E 1.9750 7.6380 7.4100 0.0030

16 13 C3 CT M 3.0050 7.2610 5.5790 0.0580

17 16 H3 H1 E 3.4420 8.2260 5.3670 0.0400

18 16 H4 H1 E 2.0500 7.1820 5.0820 0.0400

19 16 O2 OS M 3.8690 6.2240 5.1430 -0.3150

20 19 C8 CT M 4.1070 6.2720 3.7460 -0.0580

21 20 H8 H1 E 3.1780 6.2050 3.2010 0.0810

22 20 H9 H1 E 4.6160 7.1870 3.4790 0.0810

23 20 C11 CA M 4.9730 5.0850 3.4560 0.4540

24 23 N1 NC E 6.2800 5.2650 3.6920 -0.6140

25 23 C14 CA M 4.4400 3.8850 3.0220 -0.2900

26 25 H12 HA E 3.3790 3.7840 2.8500 0.1340

27 25 C17 CA M 5.2850 2.8260 2.8120 -0.0570

28 27 H15 HA E 4.9050 1.8880 2.4300 0.1490

29 27 C21 CA M 6.6230 2.9750 3.0930 -0.2900

30 29 H17 HA E 7.3060 2.1480 2.9670 0.1340

31 29 C18 CA M 7.0790 4.2060 3.5410 0.4540

32 31 C22 CT M 8.5060 4.4180 3.9370 -0.0580

33 32 H18 H1 E 8.8290 5.4240 3.7080 0.0810

34 32 H19 H1 E 9.1560 3.7100 3.4450 0.0810

35 32 O5 OS M 8.4750 4.1750 5.4820 -0.3150

36 35 C27 CT M 9.6240 4.4970 5.8230 0.0580

37 36 H23 H1 E 10.3650 3.9360 5.2750 0.0400

38 36 H24 H1 E 9.7930 5.5540 5.6790 0.0400

39 36 C28 CT M 9.6410 4.1060 7.4000 0.2310

40 39 H25 H1 E 10.6560 4.0030 7.7520 0.0030

41 39 H26 H1 E 9.1060 3.1820 7.5670 0.0030

42 39 O6 OS M 8.9910 5.1850 8.0610 -0.2790

43 42 C23 CA M 8.9770 5.1270 9.4290 0.2230

44 43 C25 CA B 9.5930 4.1320 10.1880 -0.1810

45 44 C29 CA B 9.5160 4.1920 11.5710 -0.2020

46 45 C26 CA B 8.8230 5.1850 12.2160 -0.2370

47 46 C24 CA S 8.1940 6.1670 11.4560 -0.0810

48 47 H20 HA E 7.6420 6.9580 11.9420 0.1290

49 46 H22 HA E 8.7670 5.2050 13.2940 0.1640

50 45 H27 HA E 10.0180 3.4360 12.1580 0.1690

51 44 H21 HA E 10.1220 3.3230 9.7050 0.1240

52 43 C19 CA M 8.2770 6.1270 10.0980 0.1110

53 52 O4 OS M 7.5840 7.0840 9.3400 -0.2300

54 53 C12 CA M 8.0410 8.3760 9.3740 0.1210

55 54 C9 CA M 7.1560 9.3820 9.0510 0.1210

56 55 C13 CA M 7.5570 10.6860 8.9470 -0.1510

57 56 H11 HA E 6.8480 11.4550 8.6800 0.1480

58 56 C16 CA M 8.8890 11.0130 9.1890 -0.1800

59 58 H14 HA E 9.2190 12.0390 9.1000 0.1520

60 58 C20 CA M 9.7830 10.0290 9.5400 -0.1800

61 60 H16 HA E 10.8150 10.2830 9.7290 0.1520

62 60 C15 CA M 9.3630 8.7220 9.6500 -0.1510

63 62 H13 HA E 10.0640 7.9570 9.9520 0.1480

DIZTIPg

1 0 C1 CT M 7.0660 7.3640 6.0980 -0.1890

2 1 H1 H1 E 6.7960 6.6930 5.2950 0.1120

3 1 H2 H1 E 7.7450 6.8340 6.7490 0.1120

4 1 H3 H1 E 6.1620 7.6010 6.6370 0.1120

5 1 N1 NO M 7.7070 8.6360 5.7280 0.7910

6 5 O2 O E 7.0520 9.5160 5.3170 -0.4680

7 5 O1 O M 8.9040 8.6980 5.8590 -0.4680

DOXWAOh

1 0 O9 OS M -2.7560 9.2080 10.4180 -0.3020

2 1 C21 CT M -1.9120 9.7510 9.3220 0.0680

3 2 H29 H1 E -1.4250 8.9570 8.7920 0.0600

4 2 H30 H1 E -1.1770 10.4240 9.7070 0.0600

5 2 C19 CT M -2.8560 10.4580 8.4430 0.0670

6 5 H25 H1 E -2.3140 10.9050 7.6330 0.0400

7 5 H26 H1 E -3.3490 11.2260 9.0040 0.0400

8 5 O7 OS M -3.7950 9.5960 7.9360 -0.2760

9 8 C16 CT M -4.4970 9.8890 6.8450 0.0210

10 9 H18 H1 E -4.3670 10.9420 6.7290 0.0610

11 9 H19 H1 E -3.9720 9.3780 6.0580 0.0610

12 9 C14 CT M -5.6770 9.7080 6.5790 0.0060

13 12 H14 H1 E -5.7250 9.4580 5.5330 0.0970

14 12 H15 H1 E -6.1820 10.6310 6.7450 0.0970

15 12 O4 OS M -6.3750 8.7340 7.2530 -0.3440

16 15 C11 CT M -7.8030 8.7510 7.0560 0.0060

17 16 H11 H1 E -8.1440 9.7600 7.1620 0.1300

18 16 C9 CT M -8.1980 8.2770 5.6540 0.0450

19 18 H9 H1 E -7.6900 7.3500 5.4980 0.1140

20 18 O2 OS M -7.8360 9.2260 4.6260 -0.2670

21 20 C4 CT M -8.2400 8.7430 3.3410 0.1730

22 21 C1 CA S -7.7500 9.7770 2.3020 0.0600

23 22 C2 CA B -6.5760 10.4930 2.5200 -0.1180

24 23 C5 CA B -6.1880 11.4850 1.6160 -0.1680

25 24 C7 CA B -6.9730 11.7520 0.4960 -0.1200

26 25 C6 CA B -8.1460 11.0280 0.2780 -0.1680

27 26 C3 CA S -8.5350 10.0450 1.1800 -0.1180

28 27 H2 HA E -9.4340 9.4890 1.0130 0.1220

29 26 H5 HA E -8.7500 11.2370 -0.5690 0.1370

30 25 H6 HA E -6.6740 12.5110 -0.1970 0.1280

31 24 H4 HA E -5.2850 12.0370 1.7840 0.1370

32 23 H1 HA E -5.9710 10.2860 3.3780 0.1220

33 21 H3 H2 E -7.8240 7.7640 3.2150 0.0890

34 21 O1 OS M -9.5870 8.6220 3.1980 -0.3420

35 34 C8 CT M -10.1500 7.6910 4.1840 -0.0180

36 35 H7 H1 E -9.7810 6.7010 4.0020 0.0950

37 35 H8 H1 E -11.2170 7.6900 4.1170 0.0950

38 35 C10 CT M -9.7120 8.1650 5.5690 0.0970

39 38 H10 H1 E -10.1290 9.1290 5.7570 0.1470

40 38 O3 OS M -10.1750 7.1990 6.5480 -0.3660

41 40 C13 CT M -9.9480 7.6390 7.8690 0.0290

42 41 O6 OS S -10.6000 8.8030 8.1160 -0.2780

43 42 C17 CT 3 -12.0780 8.6650 8.1600 -0.0250

44 43 H20 H1 E -12.5210 9.6180 8.3620 0.0750

45 43 H21 H1 E -12.4320 8.2940 7.2250 0.0750

46 43 H22 H1 E -12.3460 7.9790 8.9400 0.0750

47 41 H13 H2 E -10.3260 6.8980 8.5390 0.1590

48 41 C12 CT M -8.4470 7.8290 8.1040 -0.0240

49 48 H12 H1 E -8.0550 6.8350 8.0310 0.1790

50 48 O5 OS M -8.1260 8.3630 9.3840 -0.3250

51 50 C15 CT M -8.7590 7.6650 10.4480 0.1360

52 51 H16 H1 E -8.5020 6.6260 10.4000 0.0260

53 51 H17 H1 E -9.8190 7.7740 10.3720 0.0260

54 51 C18 CT M -8.2770 8.2510 11.7410 0.0700

55 54 H23 H1 E -8.8430 7.8430 12.5530 0.0450

56 54 H24 H1 E -8.3880 9.3110 11.7220 0.0450

57 54 O8 OS M -6.8910 7.9060 11.8920 -0.2930

58 57 C20 CT M -6.4660 8.4580 13.1480 0.0150

59 58 H27 H1 E -7.0950 8.1100 13.9390 0.0560

60 58 H28 H1 E -6.5000 9.5280 13.1100 0.0560

61 58 C22 CT M -5.0860 8.0010 13.3570 0.1810

62 61 H31 H1 E -5.0820 6.9340 13.3550 0.0270

63 61 H32 H1 E -4.7560 8.3600 14.3110 0.0270

64 61 O10 OS M -4.1750 8.4500 12.3830 -0.3260

65 64 C24 CA M -2.8580 8.1910 12.4970 0.2220

66 65 C23 CA M -2.0550 8.6050 11.4340 0.1770

67 66 C25 CA M -0.6770 8.3890 11.4740 -0.1690

68 67 H33 HA E -0.0630 8.7060 10.6580 0.1330

69 67 C27 CA M -0.1020 7.7600 12.5790 -0.2180

70 69 H35 HA E 0.9550 7.5980 12.6100 0.1620

71 69 C28 CA M -0.9030 7.3460 13.6430 -0.2030

72 71 H36 HA E -0.4610 6.8660 14.4890 0.1600

73 71 C26 CA M -2.2810 7.5610 13.6010 -0.1770

74 73 H34 HA E -2.8960 7.2430 14.4170 0.1170

DOXWAOg

1 0 C8 CT M -6.1080 2.9310 10.6590 -0.0210

2 1 H9 H1 E -6.9980 2.6120 10.1590 0.1130

3 1 H10 H1 E -5.3040 2.2670 10.4120 0.1130

4 1 H11 H1 E -6.2620 2.9360 11.7100 0.1130

5 1 O2 OS M -5.2690 4.1130 10.6660 -0.3960

6 5 C3 C M -5.5390 5.1820 9.9150 0.7680

7 6 O1 O E -6.5460 5.1820 9.2180 -0.5640

8 6 C1 CT M -4.5370 6.2680 10.0790 -0.0150

9 8 N1 N3 3 -5.0880 7.5610 9.5760 -0.2320

10 9 H2 H E -4.3900 8.2870 9.6730 0.2910

11 9 H3 H E -5.9070 7.8120 10.1110 0.2910

12 9 H4 H E -5.3390 7.4680 8.6020 0.2910

13 8 H1 HP E -4.2890 6.4050 11.1110 0.1260

14 8 C2 CA M -3.2950 5.8630 9.2900 -0.0810

15 14 C4 CA M -3.2930 5.7850 7.8970 -0.1210

16 15 H5 HA E -4.1650 6.0660 7.3440 0.1480

17 15 C6 CA M -2.1520 5.3540 7.2230 -0.1320

18 17 H7 HA E -2.1480 5.2980 6.1530 0.1640

19 17 C9 CA M -1.0110 4.9920 7.9420 -0.0720

20 19 H12 HA E -0.1370 4.6590 7.4250 0.1590

21 19 C7 CA M -1.0150 5.0610 9.3350 -0.1320

22 21 H8 HA E -0.1410 4.7860 9.8870 0.1640

23 21 C5 CA M -2.1560 5.5010 10.0090 -0.1210

24 23 H6 HA E -2.1560 5.5530 11.0780 0.1480

DUGGUH10h

1 0 O1 O M -2.7540 9.1930 3.8700 -0.4650

2 1 C4 C M -2.4420 10.0930 4.6190 0.3690

3 2 O2 OH S -1.2300 10.6010 4.7380 -0.4450

4 3 H7 HO E -0.5750 10.0880 4.2180 0.4220

5 2 C1 CA M -3.4070 10.7440 5.5740 0.0860

6 5 C2 CA M -4.5360 11.4200 5.0670 0.0100

7 6 C5 CT M -4.8050 11.4990 3.5890 0.0320

8 7 H1 H1 E -4.9830 10.6140 3.2620 0.0830

9 7 H2 H1 E -5.5770 12.0520 3.4490 0.0830

10 7 O3 OS M -3.7260 12.0420 2.8430 -0.3250

11 10 C10 CT M -3.5000 13.3980 3.1640 0.0280

12 11 H9 H1 E -4.3030 13.8980 3.0110 0.0690

13 11 H10 H1 E -3.2490 13.4650 4.0870 0.0690

14 11 C12 CT M -2.4030 13.9650 2.3230 0.0760

15 14 H13 H1 E -2.6320 13.8770 1.3950 0.0550

16 14 H14 H1 E -2.2780 14.8930 2.5340 0.0550

17 14 O5 OS M -1.2150 13.2570 2.5830 -0.3540

18 17 C14 CT M -0.0870 13.8550 1.9830 0.0850

19 18 H17 H1 E -0.2660 14.0200 1.0530 0.0480

20 18 H18 H1 E 0.1030 14.6850 2.4230 0.0480

21 18 C16 CT M 1.0900 12.9430 2.1080 0.0730

22 21 H21 H1 E 1.8920 13.4310 1.9070 0.0430

23 21 H22 H1 E 1.1370 12.6050 3.0040 0.0430

24 21 O7 OS M 0.9680 11.8590 1.2070 -0.3750

25 24 C18 CT M 2.0770 10.9820 1.2610 0.1650

26 25 H25 H1 E 2.8490 11.4080 0.8820 0.0300

27 25 H26 H1 E 2.2580 10.7410 2.1720 0.0300

28 25 C20 CT M 1.7320 9.7550 0.4670 0.1450

29 28 H29 H1 E 0.9160 9.3680 0.7940 0.0170

30 28 H30 H1 E 1.6280 9.9790 -0.4600 0.0170

31 28 O9 OS M 2.8140 8.8310 0.6340 -0.3970

32 31 C22 CT M 2.6380 7.6180 -0.0910 0.1150

33 32 H33 H1 E 1.8210 7.1910 0.1830 0.0460

34 32 H34 H1 E 2.5970 7.8060 -1.0310 0.0460

35 32 C24 CT M 3.8040 6.7180 0.1980 0.1030

36 35 H37 H1 E 3.6850 5.8800 -0.2570 0.0330

37 35 H38 H1 E 4.6160 7.1320 -0.1010 0.0330

38 35 O11 OS M 3.8630 6.4960 1.5940 -0.3560

39 38 C25 CT M 4.8310 5.5480 1.9910 0.1030

40 39 H39 H1 E 4.5620 4.6720 1.7040 0.0330

41 39 H40 H1 E 5.6780 5.7670 1.5980 0.0330

42 39 C23 CT M 4.9450 5.5760 3.4840 0.1150

43 42 H35 H1 E 5.6930 5.0390 3.7550 0.0460

44 42 H36 H1 E 5.0750 6.4800 3.7770 0.0460

45 42 O10 OS M 3.7570 5.0580 4.0670 -0.3970

46 45 C21 CT M 3.8340 5.0810 5.4910 0.1450

47 46 H31 H1 E 4.6650 4.6920 5.7710 0.0170

48 46 H32 H1 E 3.7810 5.9880 5.7990 0.0170

49 46 C19 CT M 2.7030 4.2940 6.0630 0.1650

50 49 H27 H1 E 2.6690 3.4360 5.6330 0.0300

51 49 H28 H1 E 2.8500 4.1760 7.0030 0.0300

52 49 O8 OS M 1.4520 4.9530 5.8770 -0.3750

53 52 C17 CT M 0.4000 4.1570 6.4210 0.0730

54 53 H23 H1 E 0.4660 3.2660 6.0670 0.0430

55 53 H24 H1 E 0.4880 4.1300 7.3750 0.0430

56 53 C15 CT M -0.9300 4.7220 6.0700 0.0850

57 56 H19 H1 E -0.9470 4.9410 5.1330 0.0480

58 56 H20 H1 E -1.6080 4.0700 6.2560 0.0480

59 56 O6 OS M -1.1830 5.8980 6.8370 -0.3540

60 59 C13 CT M -2.4540 6.4510 6.5050 0.0760

61 60 H15 H1 E -2.4710 6.6700 5.5690 0.0550

62 60 H16 H1 E -3.1400 5.8100 6.6960 0.0550

63 60 C11 CT M -2.6870 7.6890 7.3090 0.0280

64 63 H11 H1 E -3.5760 8.0150 7.1460 0.0690

65 63 H12 H1 E -2.5860 7.4890 8.2420 0.0690

66 63 O4 OS M -1.7440 8.6770 6.9290 -0.3250

67 66 C7 CT M -1.9870 9.9480 7.5320 0.0320

68 67 H4 H1 E -2.1280 9.8190 8.4720 0.0830

69 67 H5 H1 E -1.2130 10.5020 7.3970 0.0830

70 67 C3 CA M -3.1970 10.6560 6.9500 0.0100

71 70 C8 CA M -4.1180 11.2270 7.8090 -0.1530

72 71 H6 HA E -3.9980 11.1400 8.7560 0.1360

73 71 C9 CA M -5.2110 11.9170 7.3210 -0.1930

74 73 H8 HA E -5.8270 12.3330 7.9290 0.1620

75 73 C6 CA M -5.4150 12.0100 5.9620 -0.1530

76 75 H3 HA E -6.1760 12.4890 5.6280 0.1360

DUGGUH10g

1 0 O1 O M 0.6980 9.3740 3.6400 -0.6380

2 1 C1 C M 1.0640 8.2850 4.0950 0.9940

3 2 N2 N B 0.5890 7.7800 5.2400 -1.0170

4 3 H3 H E 0.8230 6.9040 5.4970 0.4200

5 3 H4 H E -0.0810 8.1110 5.5470 0.4200

6 2 N1 N M 2.0240 7.5710 3.4860 -1.0170

7 6 H1 H E 2.2820 7.8610 2.7240 0.4200

8 6 H2 H E 2.2740 6.8240 3.8360 0.4200

DUKHUMh

1 0 N1 NC M 6.2750 8.5460 12.7440 -0.4420

2 1 C1 CA M 7.3510 7.8510 13.1510 0.2620

3 2 C3 CT M 8.4660 8.6450 13.7670 0.1050

4 3 H1 H1 E 8.6970 9.4490 13.2300 0.0460

5 3 H2 H1 E 9.1740 8.1150 13.8910 0.0460

6 3 O1 OS M 8.0320 9.1340 15.0330 -0.4020

7 6 C8 CT M 9.0930 9.7620 15.7470 0.1090

8 7 H8 H1 E 9.7770 9.0910 15.9950 0.0440

9 7 H9 H1 E 9.5870 10.4120 15.2290 0.0440

10 7 C10 CT M 8.5400 10.4480 16.9440 0.1110

11 10 H12 H1 E 8.0670 9.7770 17.4850 0.0440

12 10 H13 H1 E 9.1670 10.8760 17.4330 0.0440

13 10 O3 OS M 7.6570 11.4580 16.5430 -0.3810

14 13 C12 CT M 7.1800 12.3300 17.5800 0.0850

15 14 H16 H1 E 7.3580 11.8270 18.5440 0.0460

16 14 H17 H1 E 6.3630 12.7640 17.4620 0.0460

17 14 C14 CT M 7.8930 13.6430 17.6000 0.0760

18 17 H20 H1 E 7.5290 14.2040 18.4360 0.0510

19 17 H21 H1 E 8.9240 13.4640 17.6270 0.0510

20 17 O5 OS M 7.6200 14.3400 16.4080 -0.3420

21 20 C16 CT M 8.2130 15.6690 16.4050 0.0740

22 21 H24 H1 E 9.1670 15.5820 16.5700 0.0420

23 21 H25 H1 E 7.7550 16.1390 17.1560 0.0420

24 21 C18 CT M 8.0800 16.2880 15.0620 0.0760

25 24 H28 H1 E 6.9810 16.3960 14.5710 0.0470

26 24 H29 H1 E 8.4230 17.2950 15.1800 0.0470

27 24 O7 OS M 8.8680 15.5740 14.1840 -0.3390

28 27 C20 CT M 8.9930 16.1780 12.9100 0.1310

29 28 H32 H1 E 10.0830 15.8950 12.5650 0.0170

30 28 H33 H1 E 8.9720 17.2000 13.1180 0.0170

31 28 C22 CT M 8.0530 15.6140 11.9190 0.1430

32 31 H36 H1 E 8.3390 15.9150 10.9270 0.0410

33 31 H37 H1 E 8.0550 14.5600 12.0070 0.0410

34 31 O9 OS M 6.7170 16.0280 12.1850 -0.3890

35 34 C23 CT M 5.8210 15.5090 11.2130 0.1430

36 35 H38 H1 E 5.8300 14.4920 11.2830 0.0410

37 35 H39 H1 E 6.0410 15.7860 10.3730 0.0410

38 35 C21 CT M 4.4360 15.9770 11.5110 0.1310

39 38 H34 H1 E 3.7810 15.6450 10.8300 0.0170

40 38 H35 H1 E 4.3650 16.9660 11.6820 0.0170

41 38 O8 OS M 3.9810 15.3390 12.7010 -0.3390

42 41 C19 CT M 2.7860 15.9680 13.2050 0.0760

43 42 H30 H1 E 2.9850 16.8430 13.4350 0.0470

44 42 H31 H1 E 2.1400 16.0770 12.4280 0.0470

45 42 C17 CT M 2.1890 15.1490 14.2850 0.0740

46 45 H26 H1 E 2.7380 14.9060 14.9140 0.0420

47 45 H27 H1 E 1.5300 15.6920 14.7830 0.0420

48 45 O6 OS M 1.5920 13.9910 13.7260 -0.3420

49 48 C15 CT M 0.8590 13.2280 14.6790 0.0760

50 49 H22 H1 E 0.3670 13.8560 15.4700 0.0510

51 49 H23 H1 E 0.0860 12.8310 14.2560 0.0510

52 49 C13 CT M 1.7020 12.1100 15.2540 0.0850

53 52 H18 H1 E 2.5750 12.4180 15.7050 0.0460

54 52 H19 H1 E 1.2460 11.7250 16.0670 0.0460

55 52 O4 OS M 1.8530 11.0890 14.2620 -0.3810

56 55 C11 CT M 2.6150 10.0120 14.7670 0.1110

57 56 H14 H1 E 3.4130 10.3590 15.1370 0.0440

58 56 H15 H1 E 2.0720 9.5800 15.4730 0.0440

59 56 C9 CT M 2.8430 9.0110 13.6810 0.1090

60 59 H10 H1 E 1.9620 8.8210 13.3140 0.0440

61 59 H11 H1 E 3.3730 8.1880 14.1250 0.0440

62 59 O2 OS M 3.6430 9.6260 12.6560 -0.4020

63 62 C5 CT M 4.0980 8.6830 11.6890 0.1050

64 63 H4 H1 E 3.3110 8.0740 11.4520 0.0460

65 63 H5 H1 E 4.2980 9.1320 11.0170 0.0460

66 63 C2 CA M 5.2610 7.8590 12.1850 0.2620

67 66 C6 CA M 5.2890 6.4840 12.0360 -0.1970

68 67 H6 HA E 4.4680 6.0670 11.6460 0.1270

69 67 C7 CA M 6.4050 5.7910 12.4550 -0.1050

70 69 H7 HA E 6.4500 4.9080 12.3400 0.1540

71 69 C4 CA M 7.4540 6.4810 13.0090 -0.1970

72 71 H3 HA E 8.2580 6.0150 13.3320 0.1270

DUKHUMg

1 0 N1 NH M 6.0930 11.3090 14.1290 -0.9560

2 1 H1 H E 5.9690 10.6050 13.8210 0.4710

3 1 H2 H E 6.7310 11.3670 14.7000 0.4710

4 1 C1 C2 M 5.4880 12.4120 13.7330 1.0410

5 4 N3 NH B 4.5210 12.3620 12.8270 -0.9560

6 5 H5 H E 4.0800 13.0100 12.6740 0.4710

7 5 H6 H E 4.1840 11.7030 12.6940 0.4710

8 4 N2 NH M 5.8450 13.5870 14.2330 -0.9560

9 8 H3 H E 5.5310 14.3080 13.8930 0.4710

10 8 H4 H E 6.6270 13.6020 14.5870 0.4710

EBASEFh

1 0 C19 CA M 6.6140 0.7280 21.9370 -0.1130

2 1 C14 CA B 7.5710 0.9490 20.9890 -0.2550

3 2 C9 CA S 7.3300 0.8480 19.6140 -0.0080

4 3 C15 CT 3 8.5010 1.0520 18.6560 0.4100

5 4 C20 CT 3 8.1430 1.7130 17.3700 -0.1960

6 5 H9 HC E 7.4700 1.2400 16.8890 0.0350

7 5 H10 HC E 7.8030 2.6130 17.5280 0.0350

8 5 H11 HC E 8.9110 1.8100 16.7980 0.0350

9 4 C21 CT 3 9.5810 1.9190 19.2780 -0.1960

10 9 H12 HC E 10.2320 2.1620 18.6650 0.0350

11 9 H13 HC E 9.9990 1.4400 20.0190 0.0350

12 9 H14 HC E 9.1820 2.7120 19.6650 0.0350

13 4 C22 CT 3 9.1180 -0.2970 18.3660 -0.1960

14 13 H15 HC E 9.8550 -0.2160 17.8070 0.0350

15 13 H16 HC E 8.4610 -0.8760 17.9680 0.0350

16 13 H17 HC E 9.3860 -0.7080 19.2020 0.0350

17 2 H6 HA E 8.4450 1.1840 21.2770 0.1870

18 1 H8 HA E 6.8140 0.8230 22.8550 0.1390

19 1 C13 CA M 5.3550 0.3820 21.5670 -0.1400

20 19 H5 HA E 4.6770 0.2600 22.2170 0.1450

21 19 C8 CA M 5.0740 0.2800 20.2280 -0.1480

22 21 H2 HA E 4.1980 0.0170 19.9700 0.1450

23 21 C4 CA M 6.0330 0.5020 19.2770 -0.0370

24 23 N1 N* M 5.5730 0.3610 17.9040 0.0920

25 24 C3 C S 4.9700 1.4780 17.3160 0.3670

26 25 O2 O E 4.8830 2.5020 17.9300 -0.4660

27 24 C1 C M 5.5780 -0.9000 17.3440 0.3670

28 27 O1 O E 6.0470 -1.8390 17.9570 -0.4660

29 27 C2 CA M 5.0360 -1.0510 15.9840 -0.0250

30 29 C6 CA B 5.0450 -2.2560 15.3740 -0.0930

31 30 H1 HA E 5.3920 -3.0230 15.8170 0.1570

32 30 C11 CA S 4.5570 -2.4000 14.0690 -0.0930

33 32 H3 HA E 4.5640 -3.2430 13.6530 0.1570

34 29 C5 CA M 4.5230 0.0700 15.3170 -0.0200

35 34 C7 CA M 4.4950 1.3260 15.9290 -0.0250

36 35 C12 CA M 3.9940 2.4000 15.2600 -0.0930

37 36 H4 HA E 3.9870 3.2430 15.6760 0.1570

38 36 C18 CA M 3.5070 2.2560 13.9550 -0.0930

39 38 H7 HA E 3.1590 3.0230 13.5120 0.1570

40 38 C17 CA M 3.5160 1.0510 13.3450 -0.0250

41 40 C24 C S 2.9740 0.9000 11.9850 0.3670

42 41 O4 O E 2.5040 1.8390 11.3720 -0.4660

43 40 C10 CA M 4.0290 -0.0700 14.0120 -0.0200

44 43 C16 CA M 4.0570 -1.3260 13.4000 -0.0250

45 44 C23 C M 3.5820 -1.4780 12.0130 0.3670

46 45 O3 O E 3.6690 -2.5020 11.3990 -0.4660

47 45 N2 N* M 2.9780 -0.3610 11.4250 0.0920

48 47 C25 CA M 2.5180 -0.5020 10.0530 -0.0370

49 48 C26 CA M 3.4780 -0.2800 9.1010 -0.1480

50 49 H18 HA E 4.3540 -0.0170 9.3590 0.1450

51 49 C28 CA M 3.1960 -0.3820 7.7620 -0.1400

52 51 H19 HA E 3.8750 -0.2600 7.1120 0.1450

53 51 C31 CA M 1.9370 -0.7280 7.3920 -0.1130

54 53 H21 HA E 1.7370 -0.8230 6.4740 0.1390

55 53 C29 CA M 0.9810 -0.9490 8.3400 -0.2550

56 55 H20 HA E 0.1060 -1.1840 8.0520 0.1870

57 55 C27 CA M 1.2220 -0.8480 9.7150 -0.0080

58 57 C30 CT M 0.0510 -1.0520 10.6730 0.4100

59 58 C33 CT 3 -1.0290 -1.9190 10.0510 -0.1960

60 59 H25 HC E -1.6800 -2.1620 10.6640 0.0350

61 59 H26 HC E -1.4470 -1.4400 9.3100 0.0350

62 59 H27 HC E -0.6310 -2.7120 9.6640 0.0350

63 58 C34 CT 3 -0.5660 0.2970 10.9630 -0.1960

64 63 H28 HC E -1.3040 0.2160 11.5220 0.0350

65 63 H29 HC E 0.0910 0.8760 11.3610 0.0350

66 63 H30 HC E -0.8350 0.7080 10.1270 0.0350

67 58 C32 CT M 0.4080 -1.7130 11.9590 -0.1960

68 67 H22 HC E 0.7490 -2.6130 11.8010 0.0350

69 67 H23 HC E 1.0810 -1.2400 12.4400 0.0350

70 67 H24 HC E -0.3600 -1.8100 12.5310 0.0350

EBASEFg

1 0 N1 NA M 7.6650 -2.6740 12.8460 -0.2810

2 1 H1 H E 7.4320 -3.4110 12.2600 0.3260

3 1 C1 CW M 8.3010 -2.7760 14.0430 -0.1520

4 3 H2 H4 E 8.5800 -3.6240 14.4620 0.1900

5 3 C3 C* M 8.4690 -1.5520 14.5750 -0.3290

6 5 H3 HA E 8.9170 -1.3710 15.4170 0.1820

7 5 C4 CB M 7.8900 -0.6250 13.6720 0.1650

8 7 C2 CN M 7.4080 -1.3580 12.5880 0.0830

9 8 C5 CA M 6.7910 -0.7690 11.5010 -0.2480

10 9 H4 HA E 6.4800 -1.3060 10.7580 0.1610

11 9 C7 CA M 6.6420 0.5700 11.5220 -0.1270

12 11 H6 HA E 6.2030 1.0190 10.7650 0.1420

13 11 C8 CA M 7.0970 1.3350 12.5840 -0.2060

14 13 H7 HA E 6.9660 2.3010 12.5660 0.1510

15 13 C6 CA M 7.7230 0.7620 13.6530 -0.2170

16 15 H5 HA E 8.0410 1.3060 14.3900 0.1610

EGIRIVh

1 0 C34 CT M 0.3260 -11.5950 -2.2130 -0.1800

2 1 H55 H1 E 1.1790 -11.6550 -2.7110 0.1950

3 1 H56 H1 E 0.2780 -12.3730 -1.6020 0.1950

4 1 C31 CT M 0.3230 -10.3270 -1.3940 0.0760

5 4 H49 HP E -0.5210 -10.2730 -0.8810 0.0830

6 4 H50 HP E 1.0700 -10.3590 -0.7450 0.0830

7 4 N5 N3 M 0.4620 -9.1000 -2.2370 -0.2610

8 7 H43 H E 0.1850 -8.3700 -1.7500 0.3020

9 7 H44 H E -0.0910 -9.1750 -2.9670 0.3020

10 7 C26 CT M 1.8720 -8.8650 -2.7130 -0.0930

11 10 H35 HP E 2.4140 -8.5300 -1.9540 0.1950

12 10 H36 HP E 2.2600 -9.7270 -3.0030 0.1950

13 10 C20 CA M 1.9690 -7.8790 -3.8480 -0.0090

14 13 C25 CA B 2.6770 -8.2310 -4.9800 -0.0670

15 14 C19 CA B 2.8280 -7.3160 -6.0110 -0.2020

16 15 C13 CA S 2.2810 -6.0610 -5.9250 -0.0670

17 16 H25 HA E 2.3870 -5.4420 -6.6380 0.1770

18 15 H31 HA E 3.3170 -7.5620 -6.7880 0.2350

19 14 H34 HA E 3.0610 -9.0970 -5.0520 0.1770

20 13 C14 CA M 1.4380 -6.5990 -3.7480 -0.2000

21 20 H26 HA E 0.9830 -6.3390 -2.9550 0.1330

22 20 C10 CA M 1.5660 -5.7010 -4.7840 -0.0090

23 22 C7 CT M 1.0180 -4.3010 -4.6950 -0.0930

24 23 H19 HP E 1.5050 -3.8100 -3.9870 0.1950

25 23 H20 HP E 1.1850 -3.8370 -5.5540 0.1950

26 23 N2 N3 M -0.4410 -4.2570 -4.3990 -0.2610

27 26 H13 H E -0.5880 -4.6580 -3.5840 0.3020

28 26 H14 H E -0.8860 -4.7490 -5.0360 0.3020

29 26 C4 CT M -1.0240 -2.8710 -4.3670 0.0760

30 29 H7 HP E -0.4720 -2.3010 -3.7760 0.0830

31 29 H8 HP E -1.9360 -2.9130 -3.9830 0.0830

32 29 C1 CT M -1.0960 -2.2330 -5.7490 -0.1800

33 32 H1 H1 E -1.3110 -1.2710 -5.6550 0.1950

34 32 H2 H1 E -0.2120 -2.3070 -6.1890 0.1950

35 32 N1 NT M -2.1180 -2.8810 -6.5940 -0.5300

36 35 C2 CT M -3.4530 -2.3830 -6.2910 -0.1800

37 36 H3 H1 E -3.6300 -1.5770 -6.8400 0.1950

38 36 H4 H1 E -3.4890 -2.1140 -5.3380 0.1950

39 36 C5 CT M -4.5280 -3.4090 -6.5560 0.0760

40 39 H9 HP E -5.4170 -2.9780 -6.4900 0.0830

41 39 H10 HP E -4.4290 -3.7620 -7.4770 0.0830

42 39 N3 N3 M -4.4480 -4.5370 -5.5780 -0.2610

43 42 H15 H E -4.5460 -4.2050 -4.7250 0.3020

44 42 H16 H E -3.6170 -4.9270 -5.6350 0.3020

45 42 C8 CT M -5.5050 -5.5840 -5.8250 -0.0930

46 45 H21 HP E -6.3760 -5.1390 -5.9770 0.1950

47 45 H22 HP E -5.2760 -6.0940 -6.6420 0.1950

48 45 C11 CA M -5.6310 -6.5320 -4.6710 -0.0090

49 48 C16 CA S -5.2000 -7.8680 -4.7820 -0.2000

50 49 H28 HA E -4.7920 -8.1650 -5.5860 0.1330

51 48 C15 CA M -6.2490 -6.1290 -3.4880 -0.0670

52 51 H27 HA E -6.5610 -5.2350 -3.4040 0.1770

53 51 C21 CA M -6.4120 -7.0100 -2.4390 -0.2020

54 53 H32 HA E -6.8310 -6.7190 -1.6360 0.2350

55 53 C27 CA M -5.9680 -8.3130 -2.5510 -0.0670

56 55 H37 HA E -6.0730 -8.9140 -1.8220 0.1770

57 55 C22 CA M -5.3640 -8.7450 -3.7360 -0.0090

58 57 C28 CT M -4.9710 -10.1930 -3.8560 -0.0930

59 58 H38 HP E -5.5890 -10.7450 -3.3140 0.1950

60 58 H39 HP E -5.0570 -10.4760 -4.8000 0.1950

61 58 N6 N3 M -3.5630 -10.4350 -3.3960 -0.2610

62 61 H45 H E -2.9780 -10.0870 -4.0150 0.3020

63 61 H46 H E -3.4240 -9.9970 -2.5990 0.3020

64 61 C32 CT M -3.2780 -11.8880 -3.2140 0.0760

65 64 H51 HP E -3.2310 -12.3240 -4.1030 0.0830

66 64 H52 HP E -4.0230 -12.3040 -2.7110 0.0830

67 64 C35 CT M -1.9860 -12.1270 -2.4750 -0.1800

68 67 H57 H1 E -2.0340 -11.6700 -1.5980 0.1950

69 67 H58 H1 E -1.8950 -13.0980 -2.3030 0.1950

70 67 N8 NT M -0.8000 -11.6670 -3.1740 -0.5300

71 70 C36 CT M -0.4440 -12.5780 -4.2570 -0.1800

72 71 H59 H1 E -1.2760 -12.8800 -4.7010 0.1950

73 71 H60 H1 E -0.0070 -13.3770 -3.8680 0.1950

74 71 C33 CT M 0.4750 -11.9830 -5.3000 0.0760

75 74 H53 HP E 1.2480 -11.5600 -4.8480 0.0830

76 74 H54 HP E 0.8200 -12.7070 -5.8790 0.0830

77 74 N7 N3 M -0.2220 -10.9580 -6.1530 -0.2610

78 77 H47 H E -1.0090 -11.3100 -6.4710 0.3020

79 77 H48 H E -0.4270 -10.2240 -5.6370 0.3020

80 77 C30 CT M 0.6340 -10.5260 -7.3100 -0.0930

81 80 H41 HP E 0.9360 -11.3280 -7.8050 0.1950

82 80 H42 HP E 1.4370 -10.0620 -6.9640 0.1950

83 80 C24 CA M -0.0940 -9.6080 -8.2580 -0.0090

84 83 C29 CA B -0.9800 -10.1410 -9.1950 -0.0670

85 84 C23 CA B -1.6080 -9.2990 -10.0860 -0.2020

86 85 C17 CA S -1.3340 -7.9280 -10.0820 -0.0670

87 86 H29 HA E -1.7590 -7.3610 -10.7140 0.1770

88 85 H33 HA E -2.2330 -9.6530 -10.7080 0.2350

89 84 H40 HA E -1.1480 -11.0760 -9.2190 0.1770

90 83 C18 CA M 0.1620 -8.2490 -8.2620 -0.2000

91 90 H30 HA E 0.7730 -7.8920 -7.6290 0.1330

92 90 C12 CA M -0.4530 -7.3910 -9.1710 -0.0090

93 92 C9 CT M -0.1350 -5.9170 -9.1750 -0.0930

94 93 H23 HP E 0.6920 -5.7720 -8.6460 0.1950

95 93 H24 HP E 0.0550 -5.6370 -10.1060 0.1950

96 93 N4 N3 M -1.1890 -5.0500 -8.6320 -0.2610

97 96 H17 H E -1.4210 -5.3430 -7.7910 0.3020

98 96 H18 H E -1.9330 -5.1060 -9.1690 0.3020

99 96 C6 CT M -0.7380 -3.6210 -8.5540 0.0760

100 99 H11 HP E -0.4730 -3.3170 -9.4590 0.0830

101 99 H12 HP E 0.0580 -3.5650 -7.9710 0.0830

102 99 C3 CT M -1.8070 -2.6970 -8.0170 -0.1800

103 102 H5 H1 E -1.5160 -1.7610 -8.1590 0.1950

104 102 H6 H1 E -2.6360 -2.8330 -8.5420 0.1950

EGIRIVg

1 0 O1 O M -2.5660 -7.9660 -6.7240 -0.8650

2 1 C1 C M -2.0750 -7.1770 -5.9190 0.7300

3 2 O2 O E -1.9250 -5.9260 -6.1170 -0.8650

4 2 C2 C M -1.6640 -7.6940 -4.5410 0.7300

5 4 O4 O E -1.8360 -6.9440 -3.5580 -0.8650

6 4 O3 O M -1.2010 -8.8770 -4.5010 -0.8650

EGIROBh

1 0 C28 CT M 8.3980 20.2930 16.3420 -0.0890

2 1 H49 H1 E 7.5870 19.7320 16.4320 0.1620

3 1 H50 H1 E 8.5380 20.7640 17.2010 0.1620

4 1 C25 CT M 8.1860 21.3150 15.2350 0.0140

5 4 H43 HP E 7.4190 21.8980 15.4630 0.1070

6 4 H44 HP E 8.9910 21.8860 15.1510 0.1070

7 4 N5 N3 M 7.9210 20.6300 13.9310 -0.2450

8 7 H37 H E 8.0680 21.2260 13.2460 0.3060

9 7 H38 H E 8.5200 19.9390 13.8310 0.3060

10 7 C22 CT M 6.5360 20.0910 13.8130 -0.0940

11 10 H31 HP E 6.2950 19.6350 14.6570 0.2000

12 10 H32 HP E 5.9070 20.8450 13.6850 0.2000

13 10 C17 CA M 6.3860 19.1290 12.6780 0.1110

14 13 C16 C* B 5.9220 17.8910 12.5880 -0.1480

15 14 C13 C* S 5.9350 17.5400 11.2080 -0.1480

16 15 H25 HA E 5.6690 16.7120 10.8290 0.2290

17 14 H28 HA E 5.6370 17.3410 13.3090 0.2290

18 13 O1 OS M 6.6840 19.6450 11.4230 -0.2950

19 18 C10 CA M 6.3930 18.6100 10.5480 0.1110

20 19 C7 CT M 6.5530 18.9320 9.1160 -0.0940

21 20 H19 HP E 5.9840 19.7100 8.8930 0.2000

22 20 H20 HP E 6.2360 18.1620 8.5760 0.2000

23 20 N2 N3 M 7.9660 19.2360 8.7430 -0.2450

24 23 H13 H E 8.5020 18.5350 9.0010 0.3060

25 23 H14 H E 8.2410 19.9840 9.2050 0.3060

26 23 C4 CT M 8.1530 19.4790 7.2590 0.0140

27 26 H7 HP E 8.9900 19.9850 7.1140 0.1070

28 26 H8 HP E 7.4020 20.0300 6.9220 0.1070

29 26 C1 CT M 8.2130 18.1730 6.4620 -0.0890

30 29 H1 H1 E 7.3900 17.6500 6.6250 0.1620

31 29 H2 H1 E 8.2560 18.3820 5.4950 0.1620

32 29 N1 NT M 9.3770 17.3760 6.8380 -0.5380

33 32 C2 CT M 9.2220 15.9620 6.4440 -0.0890

34 33 H3 H1 E 8.8660 15.9230 5.5210 0.1620

35 33 H4 H1 E 10.1120 15.5300 6.4440 0.1620

36 33 C5 CT M 8.3040 15.1860 7.3540 0.0140

37 36 H9 HP E 8.0750 14.3210 6.9300 0.1070

38 36 H10 HP E 7.4640 15.6930 7.4820 0.1070

39 36 N3 N3 M 8.9350 14.9300 8.6860 -0.2450

40 39 H15 H E 9.2470 15.7210 9.0340 0.3060

41 39 H16 H E 9.6500 14.3610 8.5810 0.3060

42 39 C8 CT M 7.9460 14.3210 9.6320 -0.0940

43 42 H21 HP E 7.1400 14.8960 9.6610 0.2000

44 42 H22 HP E 7.6730 13.4390 9.2820 0.2000

45 42 C11 CA M 8.4520 14.1480 11.0160 0.1110

46 45 O2 OS E 8.0050 15.0510 11.9520 -0.2950

47 45 C14 C* M 9.2240 13.2190 11.5960 -0.1480

48 47 H26 HA E 9.6500 12.4870 11.1640 0.2290

49 47 C18 C* M 9.2860 13.5370 12.9850 -0.1480

50 49 H29 HA E 9.7590 13.0630 13.6600 0.2290

51 49 C19 CA M 8.5290 14.6570 13.1460 0.1110

52 51 C23 CT M 8.1010 15.3960 14.3580 -0.0940

53 52 H33 HP E 7.1870 15.7430 14.2070 0.2000

54 52 H34 HP E 8.0560 14.7560 15.1120 0.2000

55 52 N6 N3 M 8.9870 16.5480 14.7620 -0.2450

56 55 H39 H E 9.0140 17.1710 14.0870 0.3060

57 55 H40 H E 9.8430 16.2440 14.9080 0.3060

58 55 C26 CT M 8.4500 17.1810 16.0200 0.0140

59 58 H45 HP E 7.6320 17.6900 15.7950 0.1070

60 58 H46 HP E 8.1940 16.4650 16.6520 0.1070

61 58 C29 CT M 9.4270 18.1060 16.7030 -0.0890

62 61 H51 H1 E 9.1320 18.2440 17.6390 0.1620

63 61 H52 H1 E 10.3140 17.6680 16.7310 0.1620

64 61 N8 NT M 9.5750 19.4180 16.0610 -0.5380

65 64 C30 CT M 10.7870 20.0950 16.5310 -0.0890

66 65 H53 H1 E 10.7200 21.0610 16.3270 0.1620

67 65 H54 H1 E 10.8540 19.9960 17.5130 0.1620

68 65 C27 CT M 12.0290 19.5380 15.8920 0.0140

69 68 H47 HP E 12.1170 18.5820 16.1320 0.1070

70 68 H48 HP E 12.8200 20.0150 16.2500 0.1070

71 68 N7 N3 M 12.0080 19.6700 14.4170 -0.2450

72 71 H41 H E 11.3160 19.1670 14.0790 0.3060

73 71 H42 H E 11.8550 20.5480 14.1920 0.3060

74 71 C24 CT M 13.2960 19.2190 13.7980 -0.0940

75 74 H35 HP E 14.0510 19.6390 14.2790 0.2000

76 74 H36 HP E 13.3790 18.2370 13.9030 0.2000

77 74 C21 CA M 13.3850 19.5640 12.3480 0.1110

78 77 C20 C* B 13.7870 20.6760 11.6760 -0.1480

79 78 C15 C* S 13.7480 20.3490 10.2970 -0.1480

80 79 H27 HA E 13.9780 20.9190 9.5740 0.2290

81 78 H30 HA E 14.0430 21.5080 12.0540 0.2290

82 77 O3 OS M 13.0850 18.5610 11.4710 -0.2950

83 82 C12 CA M 13.3210 19.0730 10.2100 0.1110

84 83 C9 CT M 13.1390 18.1400 9.0780 -0.0940

85 84 H23 HP E 13.9130 18.2230 8.4660 0.2000

86 84 H24 HP E 13.1240 17.2140 9.4250 0.2000

87 84 N4 N3 M 11.8750 18.3970 8.3070 -0.2450

88 87 H17 H E 11.8230 19.2880 8.0930 0.3060

89 87 H18 H E 11.1500 18.1870 8.8320 0.3060

90 87 C6 CT M 11.8310 17.5780 7.0500 0.0140

91 90 H11 HP E 12.6460 17.7560 6.5180 0.1070

92 90 H12 HP E 11.8240 16.6180 7.2850 0.1070

93 90 C3 CT M 10.6050 17.9000 6.2160 -0.0890

94 93 H5 H1 E 10.5280 18.8800 6.1110 0.1620

95 93 H6 H1 E 10.7110 17.5030 5.3140 0.1620

EGIROBg

1 0 O1 O M 9.6830 18.5890 13.0360 -0.8650

2 1 C1 C M 9.9380 18.6350 11.7830 0.7300

3 2 O2 O E 10.0570 19.6570 11.1020 -0.8650

4 2 C2 C M 10.1720 17.2780 11.0930 0.7300

5 4 O4 O E 10.7210 16.3710 11.7290 -0.8650

6 4 O3 O M 9.8070 17.2430 9.8660 -0.8650

EMOZOVh

1 0 C2 CA M -1.5040 10.5600 6.5810 -0.3680

2 1 H1 HA E -2.4290 10.3560 6.5000 0.1590

3 1 C1 CA M -1.1150 11.8940 6.4430 -0.0500

4 3 C4 CT B -2.0990 13.0360 6.1900 0.0670

5 4 H2 HC E -1.6520 13.6640 5.5530 0.0670

6 4 C8 CT 3 -3.4120 12.6000 5.5310 0.0100

7 6 H5 HC E -3.2080 12.0530 4.7320 0.0030

8 6 H6 HC E -3.9200 12.0320 6.1650 0.0030

9 6 C13 CT 3 -4.2830 13.7820 5.1140 -0.0860

10 9 H9 HC E -5.0840 13.4520 4.6560 0.0130

11 9 H10 HC E -3.7780 14.3630 4.5080 0.0130

12 9 H11 HC E -4.5480 14.2910 5.9090 0.0130

13 3 C3 CA M 0.2580 12.1530 6.5680 0.2760

14 13 O1 OH S 0.8120 13.4050 6.4410 -0.6260

15 14 H4 HO E 0.2000 13.9790 6.4090 0.4280

16 13 C6 CA M 1.1570 11.1360 6.8120 -0.2140

17 16 H3 HA E 2.0850 11.3360 6.8760 0.1590

18 16 C9 CA M 0.7320 9.8290 6.9650 0.1850

19 18 O2 OH S 1.7230 8.9160 7.2190 -0.5910

20 19 H12 HO E 1.3780 8.1750 7.4110 0.4090

21 18 C5 CA M -0.6200 9.5080 6.8290 -0.0360

22 21 C10 CT M -1.0630 8.0540 6.9660 0.0990

23 22 H7 HC E -0.3430 7.4970 6.5540 0.0660

24 22 C15 CT 3 -2.3570 7.7280 6.2060 0.0030

25 24 H13 HC E -3.1250 8.1420 6.6740 0.0090

26 24 H14 HC E -2.3100 8.1150 5.2960 0.0090

27 24 C20 CT 3 -2.5800 6.2230 6.1030 -0.0870

28 27 H18 HC E -3.3520 6.0460 5.5230 0.0120

29 27 H19 HC E -2.7500 5.8560 6.9950 0.0120

30 27 H20 HC E -1.7820 5.8000 5.7240 0.0120

31 22 C14 CA M -1.1310 7.6590 8.4380 0.0280

32 31 C19 CA B -0.1280 6.8860 9.0360 0.0970

33 32 C24 CA B -0.1950 6.5780 10.3920 -0.2470

34 33 C27 CA S -1.2560 7.0340 11.1620 0.2220

35 34 O6 OH S -1.2520 6.6450 12.4900 -0.6140

36 35 H32 HO E -1.6340 7.2310 12.9540 0.4400

37 33 H22 HA E 0.4880 6.0520 10.7920 0.1540

38 32 O4 OH S 0.9190 6.4790 8.2530 -0.5790

39 38 H23 HO E 1.5600 6.2430 8.7430 0.4440

40 31 C18 CA M -2.1580 8.1220 9.2510 -0.2600

41 40 H17 HA E -2.8240 8.6710 8.8580 0.2010

42 40 C23 CA M -2.2690 7.8290 10.6130 0.0160

43 42 C28 CT M -3.4310 8.3750 11.4340 0.0660

44 43 H27 HC E -3.4180 7.8850 12.3050 0.0590

45 43 C33 CT 3 -4.7770 8.0530 10.7750 0.0130

46 45 H33 HC E -5.5060 8.4490 11.3140 0.0090

47 45 H34 HC E -4.8060 8.4640 9.8750 0.0090

48 45 C36 CT 3 -5.0080 6.5450 10.6480 -0.1000

49 48 H36 HC E -5.9100 6.3800 10.3050 0.0130

50 48 H37 HC E -4.9080 6.1240 11.5280 0.0130

51 48 H38 HC E -4.3490 6.1650 10.0280 0.0130

52 43 C32 CA M -3.2410 9.8590 11.7640 -0.0290

53 52 C35 CA B -2.3370 10.2210 12.7660 0.1520

54 53 C34 CA B -2.1660 11.5470 13.1500 -0.4030

55 54 C30 C S -2.8610 12.5640 12.5070 0.4490

56 55 O7 O E -2.7080 13.8530 12.9220 -0.7370

57 54 H35 HA E -1.5690 11.7590 13.8570 0.1370

58 53 O8 OH S -1.6150 9.2190 13.3630 -0.6140

59 58 H39 HO E -1.0970 9.5500 13.9370 0.4470

60 52 C29 CA M -3.9170 10.8990 11.1240 -0.3750

61 60 H28 HA E -4.5270 10.6810 10.4300 0.1400

62 60 C25 CA M -3.7400 12.2460 11.4560 -0.0940

63 62 C21 CT M -4.3970 13.3900 10.7030 0.0460

64 63 H21 HC E -4.5610 14.1130 11.3750 0.0650

65 63 C26 CT 3 -5.7590 13.0730 10.0700 0.0450

66 65 H24 HC E -5.6340 12.4370 9.3230 -0.0110

67 65 H25 HC E -6.3400 12.6430 10.7430 -0.0110

68 65 C31 CT 3 -6.4350 14.3380 9.5490 -0.0880

69 68 H29 HC E -5.9030 14.7150 8.8180 0.0080

70 68 H30 HC E -6.5040 14.9930 10.2740 0.0080

71 68 H31 HC E -7.3320 14.1170 9.2210 0.0080

72 63 C16 CA M -3.4070 13.9630 9.6930 -0.0210

73 72 C11 CA M -3.1790 13.3220 8.4770 -0.3050

74 73 H8 HA E -3.6240 12.4980 8.3140 0.2400

75 73 C7 CA M -2.3360 13.8170 7.4880 -0.0240

76 75 C12 CA M -1.6950 15.0310 7.7520 0.2220

77 76 O3 OH S -0.8790 15.5400 6.7620 -0.6140

78 77 H16 HO E -0.6010 16.2990 6.9920 0.4400

79 76 C17 CA M -1.8280 15.6550 8.9830 -0.3760

80 79 H15 HA E -1.3450 16.4520 9.1650 0.1720

81 79 C22 CA M -2.6660 15.1200 9.9520 0.3370

82 81 O5 OH M -2.7370 15.7490 11.1730 -0.6440

83 82 H26 HO E -2.7910 15.1730 11.7830 0.4670

EMOZOVg

1 0 C1 CR M 2.1890 11.1600 10.3340 0.0220

2 1 H1 H5 E 3.1170 11.3620 10.3200 0.2600

3 1 N1 NA M 1.6610 9.9890 10.6220 -0.1370

4 3 H2 H E 2.1080 9.2620 10.8340 0.3750

5 3 C2 CW M 0.3000 10.0900 10.5390 -0.1360

6 5 H4 H4 E -0.3300 9.3960 10.6980 0.2580

7 5 C3 CW M 0.0210 11.3570 10.1900 -0.1360

8 7 H5 H4 E -0.8430 11.7320 10.0540 0.2580

9 7 N2 NA M 1.2170 11.9990 10.0700 -0.1370

10 9 H3 H E 1.3210 12.8450 9.8500 0.3750

EMOZUBh

1 0 C2 CA M 3.4130 8.7380 -3.1240 -0.3430

2 1 H1 HA E 3.5380 9.6190 -2.7930 0.1940

3 1 C1 CA M 3.2540 7.7100 -2.1950 0.0100

4 3 C4 CT B 3.2390 7.9450 -0.6860 0.0740

5 4 H2 HC E 3.7630 7.2020 -0.2710 0.0570

6 4 C8 CT 3 3.9080 9.2590 -0.2540 0.0410

7 6 H5 HC E 3.4230 10.0200 -0.6620 0.0010

8 6 H6 HC E 4.8380 9.2760 -0.5920 0.0010

9 6 C13 CT 3 3.9250 9.4350 1.2640 -0.1050

10 9 H9 HC E 4.3970 8.6810 1.6760 0.0240

11 9 H10 HC E 4.3830 10.2720 1.4910 0.0240

12 9 H11 HC E 3.0040 9.4660 1.6000 0.0240

13 3 C3 CA M 3.0760 6.4180 -2.7120 0.1810

14 13 O1 OH S 2.8730 5.3110 -1.9350 -0.5430

15 14 H4 HO E 2.8090 5.5430 -1.1290 0.4070

16 13 C6 CA M 3.0740 6.1970 -4.0840 -0.2680

17 16 H3 HA E 2.9700 5.3130 -4.4170 0.1690

18 16 C9 CA M 3.2220 7.2490 -4.9720 0.1810

19 18 O2 OH S 3.1870 6.9910 -6.3130 -0.5430

20 19 H12 HO E 3.0820 6.0770 -6.4480 0.4070

21 18 C5 CA M 3.4000 8.5590 -4.5080 0.0100

22 21 C10 CT M 3.5370 9.7110 -5.5010 0.0740

23 22 H7 HC E 4.0870 9.3750 -6.2650 0.0570

24 22 C15 CT 3 4.2690 10.9300 -4.9200 0.0410

25 24 H13 HC E 3.7060 11.3440 -4.2200 0.0010

26 24 H14 HC E 5.1120 10.6310 -4.4950 0.0010

27 24 C20 CT 3 4.5880 11.9630 -5.9820 -0.1050

28 27 H18 HC E 5.1100 11.5460 -6.6980 0.0240

29 27 H19 HC E 5.1060 12.6930 -5.5820 0.0240

30 27 H20 HC E 3.7520 12.3200 -6.3520 0.0240

31 22 C14 CA M 2.1640 10.0860 -6.0670 0.0100

32 31 C19 CA B 1.8110 9.7950 -7.3870 0.1810

33 32 C24 CA B 0.5360 10.0660 -7.8680 -0.2680

34 33 C27 CA S -0.3980 10.6800 -7.0510 0.1810

35 34 O6 OH S -1.6760 10.9410 -7.4880 -0.5430

36 35 H32 HO E -1.7090 10.8580 -8.3220 0.4070

37 33 H22 HA E 0.3050 9.8290 -8.7580 0.1690

38 32 O4 OH S 2.7630 9.2480 -8.2120 -0.5430

39 38 H23 HO E 2.3910 8.9150 -8.8880 0.4070

40 31 C18 CA M 1.1980 10.7130 -5.2830 -0.3430

41 40 H17 HA E 1.4200 10.9260 -4.3850 0.1940

42 40 C23 CA M -0.0770 11.0480 -5.7430 0.0100

43 42 C28 CT M -1.0850 11.8020 -4.8790 0.0740

44 43 H27 HC E -1.8250 12.0850 -5.4890 0.0570

45 43 C33 CT 3 -0.4730 13.0930 -4.3130 0.0410

46 45 H33 HC E -0.0410 13.5930 -5.0500 0.0010

47 45 H34 HC E 0.2310 12.8540 -3.6590 0.0010

48 45 C36 CT 3 -1.4890 13.9970 -3.6330 -0.1050

49 48 H37 HC E -2.2160 14.2000 -4.2590 0.0240

50 48 H38 HC E -1.0530 14.8310 -3.3580 0.0240

51 48 H39 HC E -1.8540 13.5440 -2.8440 0.0240

52 43 C32 CA M -1.7110 10.8780 -3.8370 0.0100

53 52 C35 CA B -2.7360 9.9960 -4.1870 0.1810

54 53 C34 CA B -3.3140 9.1570 -3.2420 -0.2680

55 54 C30 CA S -2.8670 9.1770 -1.9310 0.1810

56 55 O7 OH S -3.5140 8.3310 -1.0460 -0.5430

57 56 H36 HO E -3.0540 8.2540 -0.3470 0.4070

58 54 H35 HA E -4.0170 8.5690 -3.4950 0.1690

59 53 O8 OH S -3.2470 9.9110 -5.4640 -0.5430

60 59 H40 HO E -2.7810 10.3780 -5.9830 0.4070

61 52 C29 CA M -1.2870 10.8580 -2.5120 -0.3430

62 61 H28 HA E -0.5800 11.4410 -2.2620 0.1940

63 61 C25 CA M -1.8380 10.0340 -1.5260 0.0100

64 63 C21 CT M -1.3410 10.0310 -0.0910 0.0740

65 64 H21 HC E -2.1390 9.8860 0.4950 0.0570

66 64 C26 CT 3 -0.6820 11.3390 0.3620 0.0410

67 66 H24 HC E 0.1540 11.4760 -0.1500 0.0010

68 66 H25 HC E -1.2890 12.0950 0.1600 0.0010

69 66 C31 CT 3 -0.3620 11.3380 1.8490 -0.1050

70 69 H29 HC E -1.1870 11.1890 2.3600 0.0240

71 69 H30 HC E 0.0260 12.2010 2.1010 0.0240

72 69 H31 HC E 0.2770 10.6220 2.0450 0.0240

73 64 C16 CA M -0.4040 8.8440 0.1280 0.0100

74 73 C11 CA M 0.8990 8.8650 -0.3530 -0.3430

75 74 H8 HA E 1.1810 9.6220 -0.8520 0.1940

76 74 C7 CA M 1.8160 7.8380 -0.1460 0.0100

77 76 C12 CA M 1.3620 6.7280 0.5690 0.1810

78 77 O3 OH S 2.2640 5.7010 0.7810 -0.5430

79 78 H16 HO E 1.8530 5.0170 1.0420 0.4070

80 77 C17 CA M 0.0610 6.6440 1.0340 -0.2680

81 80 H15 HA E -0.2290 5.8670 1.4970 0.1690

82 80 C22 CA M -0.8190 7.7000 0.8240 0.1810

83 82 O5 OH M -2.1230 7.6550 1.2490 -0.5430

84 83 H26 HO E -2.2250 7.0040 1.7690 0.4070

EMOZUBg

1 0 N1 NC M -0.1340 6.6940 -5.3560 -0.5320

2 1 C1 CA M -0.3610 5.6380 -4.6010 0.5040

3 2 H1 H5 E -0.5280 4.7750 -4.9630 0.0600

4 2 N3 ND M -0.3370 5.8720 -3.2860 -0.6190

5 4 C2 CR M -0.0730 7.1740 -3.2280 0.2660

6 5 H3 H5 E 0.0130 7.6770 -2.4270 0.1110

7 5 N2 NA M 0.0490 7.6630 -4.4480 -0.1040

8 7 H2 H E 0.2260 8.5040 -4.6390 0.3140

EZAVOQh

1 0 C9 CT M 6.0900 -1.0220 12.3880 -0.0020

2 1 H10 H1 E 6.2520 -0.1750 12.8090 0.0590

3 1 H68 H1 E 6.6840 -1.6820 12.7470 0.0590

4 1 Ha8 H1 E 6.2300 -0.9420 11.4410 0.0590

5 1 O5 OS M 4.7600 -1.4110 12.6270 -0.3110

6 5 C6 CT M 4.1990 -1.4100 11.4660 -0.0290

7 6 H7 H1 E 4.2240 -0.5160 11.0900 0.0880

8 6 H65 H1 E 4.6840 -2.0000 10.8670 0.0880

9 6 C5 CT M 2.8180 -1.8560 11.5830 0.0040

10 9 O4 OS E 2.8560 -3.2030 12.1090 -0.3000

11 9 H6 H1 E 2.4260 -1.8840 10.6850 0.1540

12 9 C4 CT M 1.9240 -0.9820 12.4770 -0.0050

13 12 O3 OS E 1.6640 0.2700 11.8420 -0.2550

14 12 H5 H1 E 2.3980 -0.8130 13.3180 0.1570

15 12 C3 CT M 0.6060 -1.6240 12.7930 -0.0240

16 15 O2 OS S -0.0150 -0.9060 13.8490 -0.3390

17 16 C8 CT 3 -1.3920 -0.6610 13.6770 -0.0060

18 17 H9 H1 E -1.5280 -0.1370 12.8850 0.0620

19 17 H67 H1 E -1.8580 -1.4960 13.5920 0.0620

20 17 Ha7 H1 E -1.7290 -0.1830 14.4380 0.0620

21 15 H4 H1 E 0.0320 -1.5730 11.9990 0.1640

22 15 C2 CT M 0.7500 -3.0740 13.1950 0.0320

23 22 O1 OS S -0.5410 -3.6430 13.3190 -0.2450

24 23 C7 CT 3 -0.5760 -4.8810 13.9080 -0.0600

25 24 H8 H1 E -0.2190 -4.8230 14.7970 0.0700

26 24 H66 H1 E -0.0490 -5.4960 13.3910 0.0700

27 24 Ha6 H1 E -1.4830 -5.1910 13.9490 0.0700

28 22 H3 H1 E 1.1890 -3.1100 14.0710 0.1470

29 22 C1 CT M 1.6180 -3.8320 12.1860 -0.0070

30 29 H2 H2 E 1.7410 -4.7560 12.4940 0.1760

31 29 O8 OS M 0.9850 -3.8390 10.9080 -0.2550

32 31 C13 CT M 0.7850 -5.0620 10.2230 -0.0050

33 32 C14 CT 3 2.1030 -5.6210 9.6840 0.0040

34 33 O9 OS E 1.8160 -6.7440 8.8270 -0.3000

35 33 C15 CT 3 3.0940 -6.1150 10.7830 -0.0290

36 35 O10 OS S 2.5600 -6.8780 11.7810 -0.3110

37 36 C18 CT 3 2.2160 -8.2430 11.4660 -0.0020

38 37 H19 H1 E 3.0010 -8.7120 11.1750 0.0590

39 37 H72 H1 E 1.5600 -8.2530 10.7640 0.0590

40 37 H01 H1 E 1.8560 -8.6720 12.2460 0.0590

41 35 H16 H1 E 3.7940 -6.6290 10.3510 0.0880

42 35 H69 H1 E 3.5130 -5.3380 11.1860 0.0880

43 33 H15 H1 E 2.5450 -4.9270 9.1540 0.1540

44 32 H14 H1 E 0.3640 -5.7160 10.8210 0.1570

45 32 C12 CT M -0.1240 -4.7480 9.0970 -0.0240

46 45 O7 OS S -1.4790 -4.6280 9.6300 -0.3390

47 46 C17 CT 3 -1.9050 -3.4810 10.0350 -0.0060

48 47 H18 H1 E -1.5730 -2.7930 9.4530 0.0620

49 47 H71 H1 E -1.5940 -3.3220 10.9300 0.0620

50 47 H00 H1 E -2.8640 -3.4740 10.0240 0.0620

51 45 H13 H1 E 0.1450 -3.9080 8.6730 0.1640

52 45 C11 CT M -0.2150 -5.8580 8.0550 0.0320

53 52 O6 OS S -0.9210 -5.4650 6.9100 -0.2450

54 53 C16 CT 3 -2.2450 -5.8140 6.7280 -0.0600

55 54 H17 H1 E -2.3010 -6.5300 6.0920 0.0700

56 54 H70 H1 E -2.6190 -6.0980 7.5630 0.0700

57 54 Ha9 H1 E -2.7320 -5.0550 6.3990 0.0700

58 52 H12 H1 E -0.7180 -6.5950 8.4630 0.1470

59 52 C10 CT M 1.0340 -6.3620 7.7020 -0.0070

60 59 H11 H2 E 0.8760 -7.1720 7.1740 0.1760

61 59 O13 OS M 1.7770 -5.4690 6.8800 -0.2550

62 61 C22 CT M 2.7750 -6.0260 6.0130 -0.0050

63 62 C23 CT 3 3.9020 -4.9640 5.9230 0.0040

64 63 O14 OS E 4.8780 -5.4330 4.9470 -0.3000

65 63 C24 CT 3 4.6340 -4.5890 7.1140 -0.0290

66 65 O15 OS S 5.0350 -5.7220 7.8510 -0.3110

67 66 C27 CT 3 5.8100 -5.0960 8.9040 -0.0020

68 67 H28 H1 E 6.1470 -5.7770 9.4940 0.0590

69 67 H76 H1 E 5.2520 -4.4930 9.3960 0.0590

70 67 H93 H1 E 6.5440 -4.6160 8.5180 0.0590

71 65 H25 H1 E 5.4170 -4.0770 6.8610 0.0880

72 65 H73 H1 E 4.0750 -4.0250 7.6690 0.0880

73 63 H24 H1 E 3.5010 -4.1470 5.5610 0.1540

74 62 H23 H1 E 3.1370 -6.8370 6.4290 0.1570

75 62 C21 CT M 2.2070 -6.3870 4.6450 -0.0240

76 75 O12 OS S 1.2850 -7.4780 4.8090 -0.3390

77 76 C26 CT 3 0.1020 -7.3160 4.0850 -0.0060

78 77 H27 H1 E -0.2480 -6.4350 4.2370 0.0620

79 77 H75 H1 E -0.5410 -7.9680 4.3730 0.0620

80 77 H92 H1 E 0.2810 -7.4330 3.1490 0.0620

81 75 H22 H1 E 1.7110 -5.6130 4.3020 0.1640

82 75 C20 CT M 3.3060 -6.7190 3.6690 0.0320

83 82 O11 OS S 2.6880 -7.0010 2.4480 -0.2450

84 83 C25 CT 3 3.6410 -7.5120 1.5310 -0.0600

85 84 H26 H1 E 3.7920 -6.8690 0.8350 0.0700

86 84 H74 H1 E 3.3100 -8.3280 1.1480 0.0700

87 84 Hb2 H1 E 4.5879 -7.7188 2.0230 0.0700

88 82 H21 H1 E 3.7340 -7.5430 3.9790 0.1470

89 82 C19 CT M 4.3440 -5.6460 3.6800 -0.0070

90 89 H20 H2 E 5.0720 -5.9220 3.0840 0.1760

91 89 O18 OS M 3.7650 -4.4690 3.1470 -0.2550

92 91 C31 CT M 4.3930 -3.7520 2.0640 -0.0050

93 92 C32 CT 3 5.3100 -2.6880 2.6030 0.0040

94 93 O19 OS E 5.7460 -1.8020 1.5750 -0.3000

95 93 C33 CT 3 6.5760 -3.2080 3.2500 -0.0290

96 95 O20 OS S 7.1730 -4.1540 2.2520 -0.3110

97 96 C36 CT 3 8.2710 -4.5170 2.5400 -0.0020

98 97 H37 H1 E 8.5950 -5.1350 1.8740 0.0590

99 97 H80 H1 E 8.2550 -4.9590 3.3940 0.0590

100 97 H96 H1 E 8.8640 -3.7630 2.5840 0.0590

101 95 H34 H1 E 6.3710 -3.6680 4.0790 0.0880

102 95 H77 H1 E 7.1880 -2.4800 3.4400 0.0880

103 93 H33 H1 E 4.8170 -2.1660 3.2690 0.1540

104 92 H32 H1 E 4.9120 -4.3800 1.5200 0.1570

105 92 C30 CT M 3.2960 -3.1900 1.2460 -0.0240

106 105 O17 OS S 2.5070 -4.2550 0.6740 -0.3390

107 106 C35 CT 3 1.1310 -4.2980 1.1690 -0.0060

108 107 H36 H1 E 1.0860 -4.8700 1.9390 0.0620

109 107 H79 H1 E 0.8500 -3.4130 1.4110 0.0620

110 107 H95 H1 E 0.5540 -4.6410 0.4810 0.0620

111 105 H31 H1 E 2.7160 -2.6640 1.8330 0.1640

112 105 C29 CT M 3.8810 -2.2570 0.2090 0.0320

113 112 O16 OS S 2.8860 -1.7200 -0.5900 -0.2450

114 113 C34 CT 3 3.2090 -1.3270 -1.7760 -0.0600

115 114 H35 H1 E 3.8340 -0.6000 -1.7130 0.0700

116 114 H78 H1 E 2.4220 -1.0310 -2.2410 0.0700

117 114 H94 H1 E 3.6100 -2.0530 -2.2570 0.0700

118 112 H30 H1 E 4.4800 -2.7800 -0.3640 0.1470

119 112 C28 CT M 4.6920 -1.1870 0.8700 -0.0070

120 119 H29 H2 E 5.0720 -0.6050 0.1820 0.1760

121 119 O23 OS M 3.8770 -0.4070 1.7340 -0.2550

122 121 C40 CT M 3.9100 1.0000 1.6620 -0.0050

123 122 C41 CT 3 4.6590 1.5440 2.8850 0.0040

124 123 O24 OS E 4.5500 2.9920 2.9320 -0.3000

125 123 C42 CT 3 6.1050 1.1560 3.0190 -0.0290

126 125 O25 OS S 6.6890 1.4520 1.8280 -0.3110

127 126 C45 CT 3 8.0600 0.9900 1.7190 -0.0020

128 127 H46 H1 E 8.4900 1.0530 2.5730 0.0590

129 127 H84 H1 E 8.5320 1.5330 1.0820 0.0590

130 127 H99 H1 E 8.0670 0.0740 1.4250 0.0590

131 125 H43 H1 E 6.1850 0.2090 3.2120 0.0880

132 125 H81 H1 E 6.5240 1.6570 3.7370 0.0880

133 123 H42 H1 E 4.1980 1.1990 3.6800 0.1540

134 122 H41 H1 E 4.3730 1.2760 0.8430 0.1570

135 122 C39 CT M 2.4750 1.5010 1.6320 -0.0240

136 135 O22 OS S 1.8780 1.1460 0.4110 -0.3390

137 136 C44 CT 3 0.7660 0.3180 0.5550 -0.0060

138 137 H45 H1 E 0.2180 0.3790 -0.2310 0.0620

139 137 H83 H1 E 0.2580 0.5930 1.3220 0.0620

140 137 H98 H1 E 1.0570 -0.5900 0.6720 0.0620

141 135 H40 H1 E 1.9720 1.1010 2.3740 0.1640

142 135 C38 CT M 2.4500 3.0430 1.7600 0.0320

143 142 O21 OS S 1.1230 3.4310 1.7920 -0.2450

144 143 C43 CT 3 0.8690 4.7630 1.4980 -0.0600

145 144 H44 H1 E -0.0770 4.9270 1.5470 0.0700

146 144 H82 H1 E 1.1830 4.9640 0.6150 0.0700

147 144 H97 H1 E 1.3220 5.3230 2.1350 0.0700

148 142 H39 H1 E 2.8720 3.4310 0.9650 0.1470

149 142 C37 CT M 3.2080 3.4930 2.9780 -0.0070

150 149 H38 H2 E 3.2240 4.4720 3.0110 0.1760

151 149 O28 OS M 2.6040 2.9880 4.1580 -0.2550

152 151 C49 CT M 2.3640 3.9420 5.2160 -0.0050

153 152 C50 CT 3 3.3810 3.8210 6.3390 0.0040

154 153 O29 OS E 3.0220 4.6930 7.3960 -0.3000

155 153 C51 CT 3 4.8120 4.1850 5.9560 -0.0290

156 155 O30 OS S 4.8550 5.4810 5.3930 -0.3110

157 156 C54 CT 3 6.1110 5.9560 5.2490 -0.0020

158 157 H55 H1 E 6.5840 5.4220 4.6070 0.0590

159 157 H88 H1 E 6.0760 6.8660 4.9410 0.0590

160 157 Ha2 H1 E 6.5670 5.9210 6.0920 0.0590

161 155 H52 H1 E 5.1490 3.5410 5.3140 0.0880

162 155 H85 H1 E 5.3780 4.1530 6.7420 0.0880

163 153 H51 H1 E 3.3710 2.9020 6.6740 0.1540

164 152 H50 H1 E 2.3770 4.8510 4.8520 0.1570

165 152 C48 CT M 0.9790 3.6270 5.7600 -0.0240

166 165 O27 OS S -0.0170 3.9880 4.7730 -0.3390

167 166 C53 CT 3 -0.7780 2.8350 4.3380 -0.0060

168 167 H54 H1 E -1.2280 3.0410 3.5140 0.0620

169 167 H87 H1 E -1.4250 2.6060 5.0070 0.0620

170 167 Ha1 H1 E -0.1850 2.0930 4.1990 0.0620

171 165 H49 H1 E 0.9050 2.6790 5.9990 0.1640

172 165 C47 CT M 0.6790 4.5020 6.9480 0.0320

173 172 O26 OS S -0.5690 4.1390 7.5280 -0.2450

174 173 C52 CT 3 -1.4950 5.1690 7.6260 -0.0600

175 174 H53 H1 E -2.3830 4.8090 7.5690 0.0700

176 174 H86 H1 E -1.3550 5.7940 6.9100 0.0700

177 174 Ha0 H1 E -1.3860 5.6180 8.4690 0.0700

178 172 H48 H1 E 0.6430 5.5250 6.5740 0.1470

179 172 C46 CT M 1.7590 4.4320 8.0200 -0.0070

180 179 H47 H2 E 1.5870 5.1260 8.6890 0.1760

181 179 O33 OS M 1.7600 3.1750 8.6560 -0.2550

182 181 C58 CT M 2.1150 3.1620 10.0490 -0.0050

183 182 H59 H1 E 2.4770 4.0410 10.2850 0.1570

184 182 C57 CT M 0.8650 2.9220 10.8700 -0.0240

185 184 O32 OS S 0.1150 4.1380 10.8150 -0.3390

186 185 C62 CT 3 -1.2710 3.9970 10.9490 -0.0060

187 186 H63 H1 E -1.4760 3.6740 11.8300 0.0620

188 186 H91 H1 E -1.5940 3.3710 10.2960 0.0620

189 186 Ha4 H1 E -1.6930 4.8480 10.8130 0.0620

190 184 H58 H1 E 0.3430 2.1940 10.4700 0.1640

191 184 C56 CT M 1.2210 2.5660 12.2980 0.0320

192 191 O31 OS S 0.0250 2.2930 13.0230 -0.2450

193 192 C61 CT 3 0.1220 2.4350 14.3810 -0.0600

194 193 H62 H1 E 0.5260 3.2810 14.5880 0.0700

195 193 H90 H1 E 0.6620 1.7260 14.7400 0.0700

196 193 Ha3 H1 E -0.7550 2.3950 14.7730 0.0700

197 191 H57 H1 E 1.6380 3.3550 12.7060 0.1470

198 191 C55 CT M 2.2240 1.4380 12.3630 -0.0070

199 198 H56 H2 E 2.4550 1.2810 13.3010 0.1760

200 198 O34 OS M 3.3950 1.8470 11.6730 -0.3000

201 200 C59 CT M 3.2060 2.1140 10.2690 0.0040

202 201 H60 H1 E 2.9790 1.2870 9.7960 0.1540

203 201 C60 CT M 4.5200 2.6370 9.7900 -0.0290

204 203 H61 H1 E 4.4650 2.8210 8.8410 0.0880

205 203 H89 H1 E 5.2030 1.9610 9.9260 0.0880

206 203 O35 OS M 4.8800 3.8020 10.4690 -0.3110

207 206 C63 CT M 5.9970 4.4390 10.0270 -0.0020

208 207 H1 H1 E 5.8100 4.8670 9.1890 0.0590

209 207 H64 H1 E 6.7040 3.8010 9.9100 0.0590

210 207 Ha5 H1 E 6.2650 5.0990 10.6720 0.0590

EZAVOQg

1 0 C4 CA M 6.5490 -0.7950 8.3710 -0.1690

2 1 H5 HA E 6.1900 -1.5510 8.7730 0.1570

3 1 C2 CA M 7.8140 -0.3460 8.7350 -0.2050

4 3 H3 HA E 8.3000 -0.8020 9.3820 0.1520

5 3 C1 CA M 8.3500 0.7840 8.1310 0.3220

6 5 N1 N2 B 9.6990 1.1510 8.0340 -0.8680

7 6 H1 H E 10.2770 0.8100 8.5690 0.3750

8 6 H2 H E 9.9490 1.7180 7.4350 0.3750

9 5 C3 CA M 7.6230 1.4680 7.1630 -0.2050

10 9 H4 HA E 7.9820 2.2240 6.7580 0.1520

11 9 C5 CA M 6.3590 1.0190 6.7990 -0.1690

12 11 H6 HA E 5.8720 1.4750 6.1520 0.1570

13 11 C6 CA M 5.8220 -0.1130 7.4030 -0.0870

14 13 C7 C M 4.4740 -0.5720 7.4540 0.7330

15 14 O1 O E 3.8560 -1.2760 8.2210 -0.5590

16 14 O2 OS M 3.7830 -0.0240 6.4450 -0.4110

17 16 C8 CT M 2.4080 -0.4840 6.4050 0.0220

18 17 H7 H1 E 2.2940 -1.1860 7.0650 0.0760

19 17 H8 H1 E 1.8260 0.2510 6.6520 0.0760

20 17 C9 CT M 1.9690 -1.0250 5.0230 -0.0030

21 20 H9 HC E 2.5180 -1.7830 4.7620 0.0380

22 20 H10 HC E 2.0490 -0.3360 4.3460 0.0380

23 20 C10 CT M 0.5380 -1.4410 5.1860 0.0110

24 23 H11 HC E 0.3570 -1.8160 6.0650 0.0070

25 23 H12 HC E -0.0830 -0.7190 4.9990 0.0070

26 23 C11 CT M 0.5530 -2.5170 4.0710 -0.0700

27 26 H13 HC E -0.3210 -2.9060 3.9900 0.0160

28 26 H14 HC E 0.8000 -2.1110 3.2390 0.0160

29 26 H15 HC E 1.1870 -3.2000 4.2970 0.0160

EZUMERh

1 0 O1 OH M -0.5760 7.1500 6.2080 -0.5960

2 1 H1 HO E 0.1710 7.7420 6.4560 0.4310

3 1 C1 CA M -0.8040 6.0390 6.9950 0.2430

4 3 C2 CA M -2.0790 5.5070 7.0400 -0.0320

5 4 C5 CT B -3.1970 6.0520 6.1580 -0.0260

6 5 H3 HC E -4.0550 5.8880 6.5990 0.0550

7 5 H4 HC E -3.0830 7.0310 6.0780 0.0550

8 4 C4 CA M -2.3250 4.4350 7.8960 -0.2670

9 8 H2 HA E -3.2030 4.0720 7.9390 0.1790

10 8 C8 CA M -1.3350 3.8620 8.6840 -0.0440

11 10 C11 CT 3 -1.5940 2.7070 9.6460 0.4390

12 11 C16 CT 3 -0.5830 1.5900 9.4160 -0.2460

13 12 H10 HC E 0.3280 1.9410 9.5030 0.0470

14 12 H11 HC E -0.7340 0.8790 10.0940 0.0470

15 12 H12 HC E -0.7150 1.2200 8.5290 0.0470

16 11 C17 CT 3 -2.9640 2.1520 9.5120 -0.2460

17 16 H13 HC E -3.6090 2.8740 9.6600 0.0470

18 16 H14 HC E -3.1200 1.4580 10.1960 0.0470

19 16 H15 HC E -3.1010 1.7970 8.6310 0.0470

20 11 C18 CT 3 -1.4030 3.2160 11.0950 -0.2460

21 20 H16 HC E -0.4990 3.5770 11.1980 0.0470

22 20 H17 HC E -1.5320 2.4790 11.7330 0.0470

23 20 H18 HC E -2.0580 3.9230 11.2860 0.0470

24 10 C6 CA M -0.0400 4.4060 8.5720 -0.2670

25 24 H5 HA E 0.6620 4.0310 9.0880 0.1790

26 24 C3 CA M 0.2470 5.4780 7.7390 -0.0320

27 26 C7 CT M 1.6550 6.0200 7.6480 -0.0260

28 27 H6 HC E 1.6220 7.0030 7.5460 0.0550

29 27 H7 HC E 2.1240 5.8290 8.4990 0.0550

30 27 C10 CA M 2.4590 5.4210 6.4940 -0.0320

31 30 C15 CA B 3.1660 4.2520 6.6650 -0.2670

32 31 C22 CA B 3.8980 3.6440 5.6450 -0.0440

33 32 C27 CA S 3.8720 4.2790 4.4050 -0.2670

34 33 H26 HA E 4.3570 3.8830 3.6890 0.1790

35 32 C28 CT 3 4.6980 2.3730 5.9320 0.4390

36 35 C34 CT 3 5.7980 2.6850 6.9520 -0.2460

37 36 H36 HC E 5.3820 3.0090 7.7780 0.0470

38 36 H37 HC E 6.3170 1.8750 7.1380 0.0470

39 36 H38 HC E 6.3850 3.3780 6.5860 0.0470

40 35 C35 CT 3 3.7790 1.2890 6.4920 -0.2460

41 40 H39 HC E 3.0770 1.0940 5.8350 0.0470

42 40 H40 HC E 3.3740 1.5740 7.3180 0.0470

43 40 H41 HC E 4.3070 0.4610 6.6510 0.0470

44 35 C36 CT 3 5.3770 1.8140 4.6630 -0.2460

45 44 H42 HC E 4.6930 1.6150 3.9890 0.0470

46 44 H43 HC E 5.8780 1.0050 4.8700 0.0470

47 44 H44 HC E 6.0050 2.4940 4.2990 0.0470

48 31 H9 HA E 3.1460 3.8300 7.5160 0.1790

49 30 C14 CA M 2.4740 6.0140 5.2270 0.2430

50 49 O3 OH S 1.7990 7.2050 5.0430 -0.5960

51 50 H20 HO E 1.1980 7.3480 4.2620 0.4310

52 49 C21 CA M 3.1810 5.4380 4.1660 -0.0320

53 52 C26 CT M 3.1710 6.0520 2.7620 -0.0260

54 53 H24 HC E 4.0280 5.8880 2.3210 0.0550

55 53 H25 HC E 3.0560 7.0310 2.8420 0.0550

56 53 C33 CA M 2.0520 5.5070 1.8800 -0.0320

57 56 C39 CA B 2.2980 4.4350 1.0240 -0.2670

58 57 C40 CA B 1.3090 3.8620 0.2350 -0.0440

59 58 C38 CA S 0.0130 4.4060 0.3480 -0.2670

60 59 H45 HA E -0.6890 4.0310 -0.1680 0.1790

61 58 C41 CT 3 1.5680 2.7070 -0.7260 0.4390

62 61 C42 CT 3 0.5570 1.5900 -0.4960 -0.2460

63 62 H48 HC E -0.3550 1.9410 -0.5830 0.0470

64 62 H49 HC E 0.7070 0.8790 -1.1740 0.0470

65 62 H50 HC E 0.6880 1.2200 0.3910 0.0470

66 61 C43 CT 3 2.9370 2.1520 -0.5920 -0.2460

67 66 H51 HC E 3.5820 2.8740 -0.7400 0.0470

68 66 H52 HC E 3.0740 1.7970 0.2890 0.0470

69 66 H53 HC E 3.0930 1.4580 -1.2760 0.0470

70 61 C44 CT 3 1.3770 3.2160 -2.1750 -0.2460

71 70 H54 HC E 0.4720 3.5770 -2.2780 0.0470

72 70 H55 HC E 1.5060 2.4790 -2.8130 0.0470

73 70 H56 HC E 2.0310 3.9230 -2.3660 0.0470

74 57 H46 HA E 3.1760 4.0720 0.9810 0.1790

75 56 C37 CA M 0.7770 6.0390 1.9250 0.2430

76 75 O4 OH S 0.5490 7.1500 2.7120 -0.5960

77 76 H47 HO E -0.1970 7.7420 2.4640 0.4310

78 75 C29 CA M -0.2740 5.4780 1.1810 -0.0320

79 78 C24 CT M -1.6820 6.0200 1.2720 -0.0260

80 79 H22 HC E -1.6480 7.0030 1.3740 0.0550

81 79 H23 HC E -2.1500 5.8290 0.4210 0.0550

82 79 C19 CA M -2.4860 5.4210 2.4260 -0.0320

83 82 C23 CA S -3.1930 4.2520 2.2550 -0.2670

84 83 H21 HA E -3.1720 3.8300 1.4040 0.1790

85 82 C12 CA M -2.5010 6.0140 3.6930 0.2430

86 85 O2 OH S -1.8260 7.2050 3.8770 -0.5960

87 86 H19 HO E -1.2250 7.3480 4.6580 0.4310

88 85 C9 CA M -3.2080 5.4380 4.7540 -0.0320

89 88 C13 CA M -3.8980 4.2790 4.5150 -0.2670

90 89 H8 HA E -4.3840 3.8830 5.2310 0.1790

91 89 C20 CA M -3.9250 3.6440 3.2750 -0.0440

92 91 C25 CT M -4.7250 2.3730 2.9880 0.4390

93 92 C31 CT 3 -5.4040 1.8140 4.2570 -0.2460

94 93 H30 HC E -4.7200 1.6150 4.9310 0.0470

95 93 H31 HC E -6.0320 2.4940 4.6210 0.0470

96 93 H32 HC E -5.9040 1.0050 4.0500 0.0470

97 92 C32 CT 3 -5.8250 2.6850 1.9680 -0.2460

98 97 H33 HC E -5.4090 3.0090 1.1420 0.0470

99 97 H34 HC E -6.3440 1.8750 1.7820 0.0470

100 97 H35 HC E -6.4120 3.3780 2.3330 0.0470

101 92 C30 CT M -3.8060 1.2890 2.4280 -0.2460

102 101 H27 HC E -3.4010 1.5740 1.6020 0.0470

103 101 H28 HC E -4.3340 0.4610 2.2690 0.0470

104 101 H29 HC E -3.1040 1.0940 3.0850 0.0470

EZUMERg

1 0 C2 CA M -0.8360 2.3310 5.3390 -0.1600

2 1 H1 HA E -1.4130 2.8050 5.9280 0.1650

3 1 C1 CA M -0.8060 0.9800 5.3150 0.1430

4 3 F1 F E -1.6180 0.2910 6.1600 -0.1480

5 3 C3 CA M -0.0130 0.2340 4.4600 -0.1600

6 5 H2 HA E -0.0130 -0.7160 4.4600 0.1650

7 5 C5 CA M 0.7790 0.9800 3.6050 0.1430

8 7 F3 F E 1.5910 0.2910 2.7600 -0.1480

9 7 C6 CA M 0.8100 2.3310 3.5800 -0.1600

10 9 H3 HA E 1.3860 2.8050 2.9920 0.1650

11 9 C4 CA M -0.0130 2.9820 4.4600 0.1430

12 11 F2 F M -0.0130 4.3240 4.4600 -0.1480

FADCAPh

1 0 C1 CA M 4.6300 3.5190 -0.6640 0.2020

2 1 C2 CA M 4.6190 3.6700 -2.0300 -0.1720

3 2 H25 HA E 5.3940 3.9940 -2.4750 0.1210

4 2 C3 CA M 3.4740 3.3500 -2.7700 -0.2190

5 4 H26 HA E 3.4710 3.4540 -3.7150 0.1670

6 4 C4 CA M 2.3530 2.8830 -2.1250 -0.2190

7 6 H27 HA E 1.5790 2.6570 -2.6280 0.1670

8 6 C5 CA M 2.3450 2.7400 -0.7370 -0.1720

9 8 H28 HA E 1.5680 2.4170 -0.2960 0.1210

10 8 C6 CA M 3.4690 3.0680 -0.0050 0.2020

11 10 O1 OS M 3.5420 2.9910 1.3730 -0.3110

12 11 C7 CT M 2.2910 2.7980 2.0720 0.1570

13 12 H2 H1 E 1.9220 1.9040 1.8640 0.0370

14 12 H14 H1 E 1.6350 3.4820 1.7840 0.0370

15 12 C8 CT M 2.5420 2.9180 3.5410 0.0770

16 15 H3 H1 E 1.7020 2.7630 4.0410 0.0290

17 15 H15 H1 E 3.2040 2.2390 3.8270 0.0290

18 15 O2 OS M 3.0360 4.2220 3.8190 -0.3370

19 18 C9 CT M 3.0440 4.5030 5.2190 0.1170

20 19 H4 H1 E 3.4360 3.7430 5.7180 0.0320

21 19 H16 H1 E 2.1200 4.6470 5.5430 0.0320

22 19 C10 CT M 3.8680 5.7430 5.4350 0.1170

23 22 H5 H1 E 3.4940 6.4860 4.8980 0.0320

24 22 H17 H1 E 3.8300 6.0030 6.3890 0.0320

25 22 O3 OS M 5.2420 5.5150 5.0520 -0.3370

26 25 C11 CT M 6.1670 5.3160 6.1310 0.0770

27 26 H6 H1 E 6.1570 6.1230 6.7030 0.0290

28 26 H18 H1 E 7.0770 5.2310 5.7510 0.0290

29 26 C12 CT M 5.9010 4.1180 6.9950 0.1570

30 29 H7 H1 E 6.6110 4.0300 7.6790 0.0370

31 29 H19 H1 E 5.0340 4.2220 7.4600 0.0370

32 29 O4 OS M 5.8760 2.9440 6.1580 -0.3110

33 32 C13 CA M 5.5780 1.7550 6.7820 0.2020

34 33 C14 CA M 5.5520 1.5750 8.1610 -0.1720

35 34 H29 HA E 5.7380 2.3080 8.7380 0.1210

36 34 C15 CA M 5.2550 0.3300 8.6970 -0.2190

37 36 H30 HA E 5.2470 0.2130 9.6400 0.1670

38 36 C16 CA M 4.9740 -0.7350 7.8760 -0.2190

39 38 H31 HA E 4.7570 -1.5800 8.2510 0.1670

40 38 C17 CA M 5.0070 -0.5720 6.4930 -0.1720

41 40 H32 HA E 4.8190 -1.3110 5.9250 0.1210

42 40 C18 CA M 5.3150 0.6580 5.9420 0.2020

43 42 O5 OS M 5.3990 0.9000 4.5870 -0.3110

44 43 C19 CT M 5.4770 -0.2740 3.7460 0.1570

45 44 H8 H1 E 4.6250 -0.7760 3.7910 0.0370

46 44 H20 H1 E 6.2040 -0.8670 4.0610 0.0370

47 44 C20 CT M 5.7420 0.1530 2.3390 0.0770

48 47 H9 H1 E 5.7780 -0.6420 1.7490 0.0290

49 47 H21 H1 E 5.0130 0.7420 2.0230 0.0290

50 47 O6 OS M 6.9790 0.8460 2.2920 -0.3370

51 50 C21 CT M 7.4470 1.0290 0.9570 0.1170

52 51 H10 H1 E 6.7230 1.3900 0.3870 0.0320

53 51 H22 H1 E 7.7430 0.1650 0.5770 0.0320

54 51 C22 CT M 8.5990 1.9960 1.0040 0.1170

55 54 H11 H1 E 8.9800 2.0980 0.0960 0.0320

56 54 H23 H1 E 9.3070 1.6350 1.5950 0.0320

57 54 O7 OS M 8.1790 3.2860 1.4940 -0.3370

58 57 C23 CT M 8.0190 4.3080 0.5070 0.0770

59 58 H12 H1 E 8.8840 4.4290 0.0410 0.0290

60 58 H24 H1 E 7.8100 5.1590 0.9690 0.0290

61 58 C24 CT M 6.9500 4.0510 -0.5290 0.1570

62 61 H1 H1 E 6.8690 4.8320 -1.1330 0.0370

63 61 H13 H1 E 7.1890 3.2590 -1.0730 0.0370

64 61 O8 OS M 5.7000 3.8230 0.1560 -0.3110

FADCAPg

1 0 N1 N3 M 5.8640 3.4740 3.1670 -0.7080

2 1 H1 H E 5.1920 3.4290 2.5510 0.4270

3 1 H2 H E 5.7120 4.2930 3.6790 0.4270

4 1 H3 H E 5.7790 2.7660 3.6920 0.4270

5 1 H4 H E 6.7150 3.4590 2.7050 0.4270

FAHDOHh

1 0 C1 CA M 3.5930 2.1820 1.0150 0.2020

2 1 C2 CA M 2.4220 2.5690 0.4010 -0.1720

3 2 H25 HA E 2.3750 3.5110 -0.1450 0.1210

4 2 C3 CA M 1.3110 1.7590 0.4810 -0.2190

5 4 H26 HA E 0.4130 2.0340 -0.0720 0.1670

6 4 C4 CA M 1.3040 0.6110 1.2400 -0.2190

7 6 H27 HA E 0.3980 0.0170 1.3610 0.1670

8 6 C5 CA M 2.4870 0.2440 1.8400 -0.1720

9 8 H28 HA E 2.5320 -0.6960 2.3900 0.1210

10 8 C6 CA M 3.6140 1.0280 1.7670 0.2020

11 10 O1 OS M 4.8230 0.7410 2.3440 -0.3110

12 11 C7 CT M 5.0780 -0.5160 2.8250 0.1570

13 12 H2 H1 E 4.8990 -1.2320 2.0230 0.0370

14 12 H14 H1 E 4.4160 -0.6630 3.6780 0.0370

15 12 C8 CT M 6.4240 -0.7050 3.3090 0.0770

16 15 H3 H1 E 6.5440 -1.7500 3.5930 0.0290

17 15 H15 H1 E 7.0960 -0.4130 2.5020 0.0290

18 15 O2 OS M 6.7370 0.0760 4.3900 -0.3370

19 18 C9 CT M 7.9390 -0.1520 5.0040 0.1170

20 19 H4 H1 E 8.7170 0.0950 4.2820 0.0320

21 19 H16 H1 E 7.8720 -1.1930 5.3190 0.0320

22 19 C10 CT M 8.2580 0.5520 6.2240 0.1170

23 22 H5 H1 E 8.4300 -0.2020 6.9920 0.0320

24 22 H17 H1 E 9.1110 1.1730 5.9510 0.0320

25 22 O3 OS M 7.3530 1.4440 6.7320 -0.3370

26 25 C11 CT M 7.7460 1.9590 7.9390 0.0770

27 26 H6 H1 E 8.5810 2.6420 7.7790 0.0290

28 26 H18 H1 E 8.0420 1.1320 8.5850 0.0290

29 26 C12 CT M 6.6680 2.6750 8.5820 0.1570

30 29 H7 H1 E 5.7980 2.0200 8.6350 0.0370

31 29 H19 H1 E 7.0180 2.9680 9.5720 0.0370

32 29 O4 OS M 6.3130 3.8200 7.9190 -0.3110

33 32 C13 CA M 5.1300 4.4160 8.2600 0.2020

34 33 C14 CA M 4.3720 4.0230 9.3480 -0.1720

35 34 H29 HA E 4.7290 3.2330 10.0090 0.1210

36 34 C15 CA M 3.1640 4.6370 9.5900 -0.2190

37 36 H30 HA E 2.5440 4.2920 10.4180 0.1670

38 36 C16 CA M 2.7260 5.6790 8.8040 -0.2190

39 38 H31 HA E 1.7720 6.1660 9.0070 0.1670

40 38 C17 CA M 3.5170 6.0940 7.7560 -0.1720

41 40 H32 HA E 3.1790 6.9130 7.1210 0.1210

42 40 C18 CA M 4.7280 5.4910 7.4980 0.2020

43 42 O5 OS M 5.5820 5.8740 6.4990 -0.3110

44 43 C19 CT M 5.2950 7.0170 5.8000 0.1570

45 44 H8 H1 E 5.1610 7.8370 6.5050 0.0370

46 44 H20 H1 E 4.3860 6.8400 5.2250 0.0370

47 44 C20 CT M 6.3480 7.3640 4.8750 0.0770

48 47 H9 H1 E 7.2790 7.4370 5.4370 0.0290

49 47 H21 H1 E 6.0420 8.3020 4.4110 0.0290

50 47 O6 OS M 6.5420 6.4810 3.8470 -0.3370

51 50 C21 CT M 6.9940 7.0610 2.6930 0.1170

52 51 H10 H1 E 7.9140 7.6050 2.9070 0.0320

53 51 H22 H1 E 6.2190 7.7340 2.3260 0.0320

54 51 C22 CT M 7.2580 6.0790 1.6680 0.1170

55 54 H11 H1 E 7.6260 6.5840 0.7750 0.0320

56 54 H23 H1 E 8.0070 5.3730 2.0270 0.0320

57 54 O7 OS M 6.1070 5.4030 1.3600 -0.3370

58 57 C23 CT M 6.1400 4.6410 0.2220 0.0770

59 58 H12 H1 E 6.2410 5.3180 -0.6260 0.0290

60 58 H24 H1 E 6.9820 3.9520 0.2870 0.0290

61 58 C24 CT M 4.9300 3.8780 0.0300 0.1570

62 61 H1 H1 E 4.9680 3.4060 -0.9520 0.0370

63 61 H13 H1 E 4.0920 4.5690 0.1150 0.0370

64 61 O8 OS M 4.7560 2.9040 0.9780 -0.3110

FAHDOHg

1 0 N2 N1 M 7.0250 2.9550 3.2430 0.0370

2 1 N1 N1 M 6.0490 3.1120 3.7010 0.4540

3 2 C6 CA M 4.8180 3.3090 4.2410 -0.0260

4 3 C5 CA B 4.4220 2.4260 5.2230 -0.1560

5 4 C4 CA S 3.1920 2.6200 5.8100 -0.1870

6 5 H6 HA E 2.8520 1.9670 6.6140 0.1710

7 4 H7 HA E 5.0640 1.5980 5.5250 0.2260

8 3 C1 CA M 4.0290 4.3450 3.7970 -0.1560

9 8 H4 HA E 4.3670 4.9960 2.9910 0.2260

10 8 C2 CA M 2.8030 4.5410 4.3940 -0.1870

11 10 H5 HA E 2.1690 5.3790 4.1040 0.1710

12 10 C3 CA M 2.3980 3.6530 5.3650 0.4630

13 12 O1 OS M 1.2070 3.7860 6.0250 -0.3120

14 13 C7 CT M 0.3570 4.8150 5.7170 -0.0470

15 14 H1 H1 E 0.1060 4.7750 4.6570 0.1080

16 14 H2 H1 E 0.8410 5.7660 5.9410 0.1080

17 14 H3 H1 E -0.5530 4.7230 6.3090 0.1080

FANJAGh

1 0 C1 CA M -5.0230 6.5700 9.7810 0.1090

2 1 O1 OS M -4.2270 5.6030 9.2290 -0.1870

3 2 C2 CT M -4.0580 5.6030 7.8090 0.0300

4 3 H1 H1 E -3.6090 6.4350 7.5150 0.0640

5 3 H2 H1 E -4.9360 5.5410 7.3570 0.0640

6 3 C5 CT M -3.2130 4.4090 7.4770 0.0300

7 6 H4 H1 E -2.5030 4.2910 8.1570 0.0640

8 6 H5 H1 E -2.7860 4.5250 6.5920 0.0640

9 6 O2 OS M -4.0780 3.2740 7.4640 -0.1870

10 9 C11 CA M -3.5140 2.0230 7.4140 0.1090

11 10 C15 CA B -2.1370 1.8040 7.4270 -0.1490

12 11 H12 HA E -1.5340 2.5380 7.4620 0.1180

13 11 C20 CA B -1.6580 0.5080 7.3870 -0.1820

14 13 C24 CA B -2.5280 -0.5600 7.3130 -0.1870

15 14 C19 CA S -3.9000 -0.3230 7.3190 -0.1350

16 15 H17 HA E -4.4970 -1.0630 7.2870 0.1440

17 14 H22 HA E -2.1950 -1.4480 7.2600 0.1460

18 13 H18 HA E -0.7210 0.3510 7.4090 0.1550

19 10 C14 CA M -4.4180 0.9570 7.3700 0.0160

20 19 C18 CT M -5.9120 1.2300 7.3620 -0.0450

21 20 H15 HC E -6.0780 2.0930 7.8200 0.0450

22 20 H16 HC E -6.2100 1.3300 6.4230 0.0450

23 20 C23 CA M -6.7480 0.1650 8.0220 -0.0150

24 23 C27 CA B -7.3740 -0.8300 7.2810 -0.2450

25 24 H23 HA E -7.2810 -0.8340 6.3360 0.1700

26 24 C31 CA B -8.1290 -1.8130 7.8890 -0.1650

27 26 C34 CA S -8.2860 -1.7920 9.2610 -0.2450

28 27 H29 HA E -8.8130 -2.4640 9.6780 0.1700

29 26 H26 HA E -8.5360 -2.4960 7.3690 0.1410

30 23 C26 CA M -6.9110 0.1630 9.4200 0.2620

31 30 O4 OH S -6.3110 1.1780 10.1060 -0.5100

32 31 H25 HO E -5.8970 0.9470 10.8380 0.3490

33 30 C30 CA M -7.6910 -0.8120 10.0460 -0.0150

34 33 C35 CT M -7.8870 -0.8320 11.5460 -0.0450

35 34 H30 HC E -7.7790 0.0900 11.8910 0.0450

36 34 H31 HC E -8.8140 -1.1170 11.7390 0.0450

37 34 C38 CA M -6.9370 -1.7400 12.2940 0.0160

38 37 C41 CA B -6.1420 -2.6930 11.6710 -0.1350

39 38 H37 HA E -6.1800 -2.7760 10.7250 0.1440

40 38 C43 CA B -5.3010 -3.5200 12.3810 -0.1870

41 40 C44 CA B -5.2220 -3.4050 13.7440 -0.1820

42 41 C42 CA S -5.9970 -2.4700 14.4150 -0.1490

43 42 H38 HA E -5.9390 -2.3920 15.3610 0.1180

44 41 H40 HA E -4.6340 -3.9700 14.2320 0.1550

45 40 H39 HA E -4.7760 -4.1690 11.9260 0.1460

46 37 C40 CA M -6.8550 -1.6570 13.7030 0.1090

47 46 O6 OS M -7.6510 -0.6890 14.2550 -0.1870

48 47 C39 CT M -7.8200 -0.6900 15.6740 0.0300

49 48 H35 H1 E -6.9420 -0.6270 16.1260 0.0640

50 48 H36 H1 E -8.2690 -1.5220 15.9690 0.0640

51 48 C36 CT M -8.6650 0.5040 16.0060 0.0300

52 51 H32 H1 E -9.3750 0.6220 15.3270 0.0640

53 51 H33 H1 E -9.0920 0.3880 16.8920 0.0640

54 51 O5 OS M -7.8000 1.6390 16.0190 -0.1870

55 54 C28 CA M -8.3640 2.8900 16.0700 0.1090

56 55 C32 CA B -9.7410 3.1090 16.0570 -0.1490

57 56 H27 HA E -10.3440 2.3750 16.0220 0.1180

58 56 C37 CA B -10.2200 4.4060 16.0970 -0.1820

59 58 C33 CA B -9.3500 5.4730 16.1710 -0.1870

60 59 C29 CA S -7.9780 5.2370 16.1650 -0.1350

61 60 H24 HA E -7.3810 5.9760 16.1970 0.1440

62 59 H28 HA E -9.6830 6.3610 16.2240 0.1460

63 58 H34 HA E -11.1570 4.5620 16.0740 0.1550

64 55 C25 CA M -7.4600 3.9560 16.1130 0.0160

65 64 C22 CT M -5.9660 3.6830 16.1210 -0.0450

66 65 H20 HC E -5.6680 3.5830 17.0610 0.0450

67 65 H21 HC E -5.8000 2.8210 15.6630 0.0450

68 65 C16 CA M -5.1300 4.7490 15.4620 -0.0150

69 68 C21 CA B -4.5040 5.7430 16.2020 -0.2450

70 69 C17 CA B -3.7490 6.7260 15.5940 -0.1650

71 70 C13 CA S -3.5920 6.7050 14.2230 -0.2450

72 71 H11 HA E -3.0650 7.3770 13.8060 0.1700

73 70 H14 HA E -3.3420 7.4090 16.1140 0.1410

74 69 H19 HA E -4.5970 5.7470 17.1480 0.1700

75 68 C12 CA M -4.9670 4.7500 14.0630 0.2620

76 75 O3 OH S -5.5670 3.7350 13.3770 -0.5100

77 76 H13 HO E -5.9810 3.9660 12.6460 0.3490

78 75 C9 CA M -4.1870 5.7250 13.4370 -0.0150

79 78 C6 CT M -3.9910 5.7450 11.9380 -0.0450

80 79 H6 HC E -4.0990 4.8230 11.5930 0.0450

81 79 H7 HC E -3.0640 6.0300 11.7440 0.0450

82 79 C3 CA M -4.9410 6.6530 11.1900 0.0160

83 82 C7 CA M -5.7360 7.6060 11.8120 -0.1350

84 83 H8 HA E -5.6980 7.6890 12.7590 0.1440

85 83 C10 CA M -6.5770 8.4330 11.1030 -0.1870

86 85 H10 HA E -7.1020 9.0820 11.5570 0.1460

87 85 C8 CA M -6.6560 8.3180 9.7400 -0.1820

88 87 H9 HA E -7.2440 8.8830 9.2510 0.1550

89 87 C4 CA M -5.8810 7.3830 9.0680 -0.1490

90 89 H3 HA E -5.9390 7.3050 8.1230 0.1180

FANJAGg

1 0 N1 NT M -6.9520 3.9940 11.1320 -1.0490

2 1 H1 H E -6.7170 4.6870 10.4850 0.3760

3 1 H2 H E -6.8040 3.2500 10.7080 0.3760

4 1 C1 CT M -8.3790 4.0890 11.5090 0.4550

5 4 H3 H1 E -8.5090 5.0470 11.7660 0.0250

6 4 C3 CT 3 -8.7810 3.3750 12.6170 -0.3150

7 6 H6 HC E -8.1900 3.5820 13.3700 0.0760

8 6 H7 HC E -8.7350 2.4160 12.4190 0.0760

9 6 H8 HC E -9.7020 3.6180 12.8470 0.0760

10 4 C2 CT M -9.3420 3.8680 10.3270 -0.1040

11 10 H4 HC E -9.2200 2.9450 9.9920 0.0310

12 10 H5 HC E -10.2710 3.9440 10.6600 0.0310

13 10 C4 CA M -9.1730 4.8050 9.1950 0.0150

14 13 C5 CA M -8.3830 4.5180 8.1110 -0.1230

15 14 H9 HA E -7.9330 3.6820 8.0720 0.1120

16 14 C7 CA M -8.2270 5.4180 7.0770 -0.1720

17 16 H11 HA E -7.6730 5.1880 6.3410 0.1450

18 16 C9 CA M -8.8420 6.6120 7.0870 -0.1270

19 18 H13 HA E -8.7100 7.2040 6.3570 0.1360

20 18 C8 CA M -9.6120 6.9810 8.0680 -0.1720

21 20 H12 HA E -10.0310 7.8330 8.0470 0.1450

22 20 C6 CA M -9.8210 6.1290 9.1470 -0.1230

23 22 H10 HA E -10.3870 6.4030 9.8580 0.1120

FIKVIEh

1 0 O1 OS M 1.0960 2.1800 9.5790 -0.3230

2 1 C1 CT M 0.2490 1.5520 8.6520 0.0820

3 2 H1 H1 E -0.1990 0.8130 9.0690 0.0580

4 2 H2 H1 E -0.4020 2.1800 8.3310 0.0580

5 2 C3 CT M 1.0890 1.0610 7.5080 0.0880

6 5 H3 H1 E 1.6520 1.7710 7.1930 0.0330

7 5 H4 H1 E 0.5190 0.7640 6.7950 0.0330

8 5 O2 OS M 1.8860 -0.0140 7.9570 -0.3020

9 8 C8 CT M 2.7180 -0.5100 6.9190 0.0850

10 9 H9 H1 E 2.1710 -0.8900 6.2270 0.0430

11 9 H10 H1 E 3.2390 0.2100 6.5540 0.0430

12 9 C10 CT M 3.6170 -1.5470 7.4630 0.0070

13 12 H11 H1 E 4.1330 -1.1750 8.1840 0.0650

14 12 H12 H1 E 4.2070 -1.8530 6.7720 0.0650

15 12 O4 OS M 2.8490 -2.6390 7.9450 -0.3070

16 15 C12 CT M 3.6410 -3.6560 8.5170 0.0070

17 16 H15 H1 E 4.2160 -4.0290 7.8430 0.0650

18 16 H16 H1 E 4.1750 -3.2840 9.2220 0.0650

19 16 C14 CT M 2.7900 -4.7160 9.0630 0.0850

20 19 H19 H1 E 2.1990 -5.0370 8.3780 0.0430

21 19 H20 H1 E 3.3370 -5.4390 9.3770 0.0430

22 19 O6 OS M 2.0230 -4.1970 10.1430 -0.3020

23 22 C16 CT M 1.4640 -5.2330 10.9220 0.0880

24 23 H23 H1 E 2.1740 -5.7630 11.2910 0.0330

25 23 H24 H1 E 0.9100 -5.7820 10.3640 0.0330

26 23 C18 CT M 0.6400 -4.6880 12.0370 0.0820

27 26 H27 H1 E 0.0110 -4.0510 11.6900 0.0580

28 26 H28 H1 E 0.1670 -5.4020 12.4720 0.0580

29 26 O8 OS M 1.4930 -4.0520 12.9630 -0.3230

30 29 C20 CA M 0.9620 -3.5950 14.1430 0.2410

31 30 C23 CA B 1.8490 -3.0260 15.0200 -0.2080

32 31 C25 CA S 1.4380 -2.5610 16.2370 -0.2080

33 32 H36 HA E 2.0750 -2.1720 16.8400 0.1470

34 31 H34 HA E 2.7750 -2.9540 14.7750 0.1470

35 30 C22 CA M -0.3650 -3.6940 14.4910 -0.2080

36 35 H33 HA E -0.9970 -4.0960 13.8920 0.1470

37 35 C24 CA M -0.7790 -3.1990 15.7330 -0.2080

38 37 H35 HA E -1.7060 -3.2500 15.9800 0.1470

39 37 C27 CA M 0.1170 -2.6450 16.5970 0.2410

40 39 O10 OS M -0.3310 -2.1150 17.7680 -0.3230

41 40 C28 CT M 0.2510 -2.2190 18.8710 0.0820

42 41 H39 H1 E 0.5740 -3.1100 19.0710 0.0580

43 41 H40 H1 E 1.0520 -1.6230 18.9360 0.0580

44 41 C26 CT M -0.8410 -1.7280 19.9720 0.0880

45 44 H37 H1 E -0.5380 -1.8790 20.8830 0.0330

46 44 H38 H1 E -1.6610 -2.2400 19.8510 0.0330

47 44 O9 OS M -1.0670 -0.3760 19.7470 -0.3020

48 47 C21 CT M -1.6290 0.2990 20.8340 0.0850

49 48 H31 H1 E -2.1150 -0.3240 21.3770 0.0430

50 48 H32 H1 E -0.9300 0.7010 21.3530 0.0430

51 48 C19 CT M -2.5360 1.3370 20.3560 0.0070

52 51 H29 H1 E -3.2140 0.9380 19.8060 0.0650

53 51 H30 H1 E -2.9480 1.7670 21.1080 0.0650

54 51 O7 OS M -1.8110 2.3070 19.5900 -0.3070

55 54 C17 CT M -2.6760 3.2960 19.0730 0.0070

56 55 H25 H1 E -3.2040 3.6550 19.7900 0.0650

57 55 H26 H1 E -3.2550 2.8920 18.4210 0.0650

58 55 C15 CT M -1.9440 4.3770 18.4440 0.0850

59 58 H21 H1 E -2.5600 5.0640 18.1800 0.0430

60 58 H22 H1 E -1.3140 4.7350 19.0730 0.0430

61 58 O5 OS M -1.2350 3.9190 17.2890 -0.3020

62 61 C13 CT M -0.7780 5.0140 16.5230 0.0880

63 62 H17 H1 E -0.2460 5.5850 17.0810 0.0330

64 62 H18 H1 E -1.5360 5.5030 16.1940 0.0330

65 62 C11 CT M 0.0500 4.5590 15.3630 0.0820

66 65 H13 H1 E 0.7380 3.9620 15.6690 0.0580

67 65 H14 H1 E 0.4510 5.3160 14.9320 0.0580

68 65 O3 OS M -0.8080 3.8840 14.4540 -0.3230

69 68 C9 CA M -0.2980 3.5080 13.2490 0.2410

70 69 C6 CA M 1.0260 3.6060 12.8890 -0.2080

71 70 H7 HA E 1.6620 3.9820 13.5020 0.1470

72 70 C4 CA M 1.4420 3.1680 11.6550 -0.2080

73 72 H5 HA E 2.3660 3.2540 11.4080 0.1470

74 72 C2 CA M 0.5590 2.6120 10.7740 0.2410

75 74 C5 CA M -0.7690 2.5190 11.1140 -0.2080

76 75 H6 HA E -1.4010 2.1430 10.4970 0.1470

77 75 C7 CA M -1.1870 2.9670 12.3500 -0.2080

78 77 H8 HA E -2.1160 2.9020 12.5850 0.1470

FIKVIEg

1 0 N1 N* M -2.1970 0.5010 14.3420 0.1040

2 1 C1 CA M -3.1720 0.1830 13.4860 -0.0250

3 2 H1 H4 E -4.0810 0.4180 13.6840 0.2150

4 2 C4 CA M -2.8700 -0.4800 12.3270 -0.0980

5 4 H4 HA E -3.5630 -0.6890 11.6980 0.2100

6 4 C8 CA M -1.5860 -0.8410 12.0680 -0.0020

7 6 H8 HA E -1.3730 -1.3230 11.2670 0.2150

8 6 C5 CA M -0.6060 -0.5130 12.9580 -0.1120

9 8 H5 HA E 0.3040 -0.7600 12.7790 0.1880

10 8 C2 CA M -0.9130 0.1620 14.1000 0.0770

11 10 C6 CA M 0.0970 0.5490 15.1010 0.0770

12 11 C9 CA M 1.4100 0.7070 14.7680 -0.1120

13 12 H9 HA E 1.6980 0.6140 13.8570 0.1880

14 12 C10 CA M 2.3210 1.0030 15.7670 -0.0020

15 14 H10 HA E 3.2530 1.0910 15.5590 0.2150

16 14 C12 CA M 1.8890 1.1690 17.0420 -0.0980

17 16 H12 HA E 2.5220 1.3600 17.7370 0.2100

18 16 C11 CA M 0.5650 1.0650 17.3410 -0.0250

19 18 H11 H4 E 0.2550 1.2070 18.2390 0.2150

20 18 N2 N* M -0.3040 0.7610 16.3560 0.1040

21 20 C7 CT M -1.7330 0.6420 16.6890 -0.0530

22 21 H6 H1 E -1.9700 -0.2830 16.7810 0.1400

23 21 H7 H1 E -1.9160 1.1030 17.5120 0.1400

24 21 C3 CT M -2.5170 1.2550 15.5820 -0.0530

25 24 H2 H1 E -2.2720 2.1770 15.4790 0.1400

26 24 H3 H1 E -3.4560 1.1920 15.7700 0.1400

FIRXOTh

1 0 O1 OS M 2.0610 9.5100 0.9320 -0.3150

2 1 C1 CT M 3.2220 9.7970 1.7100 0.0920

3 2 H1 H1 E 3.7470 10.4740 1.2770 0.0590

4 2 H2 H1 E 3.7490 9.0010 1.8120 0.0590

5 2 C3 CT M 2.7770 10.2770 3.0330 0.0140

6 5 H3 H1 E 3.5330 10.6150 3.5180 0.0570

7 5 H4 H1 E 2.1330 10.9770 2.9150 0.0570

8 5 O2 OS M 2.1900 9.2220 3.7630 -0.2570

9 8 C8 CT M 1.6750 9.6200 5.0090 0.0290

10 9 H9 H1 E 2.4000 9.9040 5.5700 0.0570

11 9 H10 H1 E 1.0670 10.3510 4.8790 0.0570

12 9 C10 CT M 0.9680 8.5130 5.6590 0.1120

13 12 H11 H1 E 0.5280 8.8410 6.4470 0.0380

14 12 H12 H1 E 0.3150 8.1550 5.0530 0.0380

15 12 O4 OS M 1.8700 7.4930 6.0260 -0.3830

16 15 C12 CT M 1.2310 6.4470 6.7330 0.1120

17 16 H15 H1 E 0.8020 6.8080 7.5120 0.0380

18 16 H16 H1 E 0.5730 6.0380 6.1670 0.0380

19 16 C14 CT M 2.2120 5.4260 7.1510 0.0290

20 19 H19 H1 E 2.8980 5.8430 7.6770 0.0570

21 19 H20 H1 E 1.7700 4.7540 7.6760 0.0570

22 19 O6 OS M 2.7880 4.8340 6.0440 -0.2570

23 22 C16 CT M 3.4360 3.5880 6.3150 0.0140

24 23 H23 H1 E 4.1840 3.7270 6.9010 0.0570

25 23 H24 H1 E 2.8200 2.9750 6.7230 0.0570

26 23 C18 CT M 3.9060 3.0490 5.0020 0.0920

27 26 H27 H1 E 4.4600 3.6960 4.5600 0.0590

28 26 H28 H1 E 4.4060 2.2410 5.1390 0.0590

29 26 O8 OS M 2.7510 2.7780 4.2200 -0.3150

30 29 C20 CA M 2.9130 2.3460 2.9390 0.1730

31 30 C23 CA B 4.1390 2.1360 2.3300 -0.1750

32 31 H34 HA E 4.9560 2.3070 2.8040 0.1370

33 31 C25 CA S 4.1680 1.6790 1.0350 -0.1750

34 33 H36 HA E 5.0140 1.5340 0.6060 0.1370

35 30 C22 CA M 1.7400 2.1020 2.2310 -0.1750

36 35 H33 HA E 0.8890 2.2470 2.6520 0.1370

37 35 C24 CA M 1.7890 1.6620 0.9450 -0.1750

38 37 H35 HA E 0.9740 1.5180 0.4600 0.1370

39 37 C27 CA M 3.0000 1.4200 0.3320 0.1730

40 39 O10 OS M 3.1710 0.9280 -0.9320 -0.3150

41 40 C28 CT M 2.0100 0.6420 -1.7100 0.0920

42 41 H39 H1 E 1.4840 -0.0360 -1.2770 0.0590

43 41 H40 H1 E 1.4830 1.4380 -1.8120 0.0590

44 41 C26 CT M 2.4550 0.1610 -3.0330 0.0140

45 44 H37 H1 E 1.6990 -0.1770 -3.5180 0.0570

46 44 H38 H1 E 3.0990 -0.5380 -2.9150 0.0570

47 44 O9 OS M 3.0420 1.2160 -3.7630 -0.2570

48 47 C21 CT M 3.5570 0.8190 -5.0090 0.0290

49 48 H31 H1 E 4.1650 0.0870 -4.8790 0.0570

50 48 H32 H1 E 2.8310 0.5350 -5.5700 0.0570

51 48 C19 CT M 4.2640 1.9250 -5.6590 0.1120

52 51 H29 H1 E 4.9170 2.2830 -5.0530 0.0380

53 51 H30 H1 E 4.7040 1.5970 -6.4470 0.0380

54 51 O7 OS M 3.3620 2.9450 -6.0260 -0.3830

55 54 C17 CT M 4.0010 3.9910 -6.7330 0.1120

56 55 H25 H1 E 4.6590 4.4000 -6.1670 0.0380

57 55 H26 H1 E 4.4300 3.6300 -7.5120 0.0380

58 55 C15 CT M 3.0200 5.0120 -7.1510 0.0290

59 58 H21 H1 E 2.3340 4.5950 -7.6770 0.0570

60 58 H22 H1 E 3.4620 5.6840 -7.6760 0.0570

61 58 O5 OS M 2.4440 5.6040 -6.0440 -0.2570

62 61 C13 CT M 1.7960 6.8510 -6.3150 0.0140

63 62 H17 H1 E 2.4120 7.4630 -6.7230 0.0570

64 62 H18 H1 E 1.0480 6.7110 -6.9010 0.0570

65 62 C11 CT M 1.3260 7.3890 -5.0020 0.0920

66 65 H13 H1 E 0.7720 6.7420 -4.5600 0.0590

67 65 H14 H1 E 0.8260 8.1970 -5.1390 0.0590

68 65 O3 OS M 2.4810 7.6600 -4.2200 -0.3150

69 68 C9 CA M 2.3190 8.0920 -2.9390 0.1730

70 69 C6 CA M 1.0930 8.3020 -2.3300 -0.1750

71 70 H7 HA E 0.2760 8.1310 -2.8040 0.1370

72 70 C4 CA M 1.0640 8.7600 -1.0350 -0.1750

73 72 H5 HA E 0.2180 8.9040 -0.6060 0.1370

74 72 C2 CA M 2.2320 9.0180 -0.3320 0.1730

75 74 C5 CA M 3.4420 8.7770 -0.9450 -0.1750

76 75 H6 HA E 4.2580 8.9200 -0.4600 0.1370

77 75 C7 CA M 3.4920 8.3360 -2.2310 -0.1750

78 77 H8 HA E 4.3430 8.1920 -2.6520 0.1370

FIRXOTg

1 0 C4 CA M 4.2680 5.6380 0.7810 -0.1170

2 1 H6 HA E 4.8520 5.4760 0.0370 0.1800

3 1 C1 CA M 4.7960 6.0580 1.9570 -0.0130

4 3 H1 H4 E 5.7460 6.1810 2.0280 0.2160

5 3 N1 N* M 4.0290 6.3030 3.0120 0.1080

6 5 C3 CT 3 4.6190 6.8420 4.2580 -0.1640

7 6 H3 H1 E 4.8610 7.7590 4.1090 0.1440

8 6 H4 H1 E 5.4130 6.3190 4.3910 0.1440

9 6 H5 H1 E 4.0610 6.7810 5.0360 0.1440

10 5 C2 CA M 2.7270 6.0970 2.9250 -0.0130

11 10 H2 H4 E 2.1750 6.2590 3.6930 0.2160

12 10 C5 CA M 2.1480 5.6580 1.7630 -0.1170

13 12 H7 HA E 1.2020 5.5000 1.7360 0.1800

14 12 C6 CA M 2.9090 5.4380 0.6300 0.0900

15 14 C7 CA M 2.3230 5.0010 -0.6300 0.0900

16 15 C8 CA M 0.9640 4.8000 -0.7810 -0.1170

17 16 H8 HA E 0.3800 4.9630 -0.0370 0.1800

18 16 C10 CA M 0.4360 4.3800 -1.9570 -0.0130

19 18 H10 H4 E -0.5140 4.2570 -2.0280 0.2160

20 18 N2 N* M 1.2030 4.1360 -3.0120 0.1080

21 20 C12 CT 3 0.6130 3.5960 -4.2580 -0.1640

22 21 H12 H1 E -0.1810 4.1200 -4.3910 0.1440

23 21 H13 H1 E 1.1710 3.6580 -5.0360 0.1440

24 21 H14 H1 E 0.3710 2.6790 -4.1090 0.1440

25 20 C11 CA M 2.5050 4.3410 -2.9250 -0.0130

26 25 H11 H4 E 3.0570 4.1800 -3.6930 0.2160

27 25 C9 CA M 3.0840 4.7800 -1.7630 -0.1170

28 27 H9 HA E 4.0300 4.9380 -1.7360 0.1800

FODTIBh

1 0 O3 OS M -1.8160 5.0270 6.1350 -0.3140

2 1 C10 CT M -2.6550 6.1690 6.4150 0.0850

3 2 H10 H1 E -2.2280 6.9740 6.1090 0.0460

4 2 H11 H1 E -2.8170 6.2330 7.3590 0.0460

5 2 C8 CT M -3.9390 5.9610 5.6890 -0.0350

6 5 H8 HC E -4.5440 6.6710 5.9200 0.0450

7 5 H9 HC E -4.3150 5.1210 5.9620 0.0450

8 5 C7 CT M -3.7620 5.9470 4.1780 -0.0630

9 8 H5 HC E -4.6130 6.1370 3.7740 0.0360

10 8 H6 HC E -3.1350 6.6360 3.9450 0.0360

11 8 C4 C M -3.2580 4.6460 3.6180 0.5470

12 11 O1 O E -3.8560 3.5980 3.8310 -0.5270

13 11 N1 N M -2.1340 4.7460 2.8330 -0.4830

14 13 H2 H E -1.6490 5.7490 2.9470 0.2940

15 13 C1 CA M -1.4950 3.7030 2.1540 0.4090

16 15 N2 NC E -0.2580 4.0120 1.7240 -0.5470

17 15 C2 CM M -2.0850 2.4780 1.9000 -0.1410

18 17 H1 HA E -2.9750 2.2850 2.2080 0.1440

19 17 C3 CA M -1.3600 1.5420 1.1920 -0.2250

20 19 H3 HA E -1.7490 0.6870 0.9980 0.1870

21 19 C6 CM M -0.0750 1.8300 0.7650 -0.1410

22 21 H4 HA E 0.4460 1.1780 0.2900 0.1440

23 21 C5 CA M 0.4330 3.0830 1.0420 0.4090

24 23 N3 N M 1.7130 3.5060 0.6430 -0.4830

25 24 H7 H E 1.9420 4.6160 0.7210 0.2940

26 24 C9 C M 2.7470 2.7190 0.2260 0.5470

27 26 O2 O E 2.6760 1.5060 0.1170 -0.5270

28 26 C11 CT M 4.0100 3.4610 -0.1430 -0.0630

29 28 H12 HC E 3.8710 4.3970 0.0180 0.0360

30 28 H13 HC E 4.1890 3.3170 -1.0730 0.0360

31 28 C12 CT M 5.2140 3.0070 0.6580 -0.0350

32 31 H14 HC E 5.1880 2.0500 0.7340 0.0450

33 31 H15 HC E 6.0110 3.2680 0.1870 0.0450

34 31 C14 CT M 5.2580 3.5990 2.0400 0.0850

35 34 H16 H1 E 6.0750 3.3480 2.4780 0.0460

36 34 H17 H1 E 5.2040 4.5550 1.9880 0.0460

37 34 O4 OS M 4.1360 3.0890 2.7730 -0.3140

38 37 C19 CA M 3.5780 3.8850 3.7320 0.2110

39 38 C21 CA M 2.2790 3.6550 4.0380 -0.2500

40 39 H21 HA E 1.8150 2.9350 3.6110 0.1680

41 39 C18 CA M 1.5950 4.4630 4.9840 0.0510

42 41 C16 CA M 0.2030 4.3280 5.2360 -0.2500

43 42 H19 HA E -0.2750 3.5940 4.8420 0.1680

44 42 C13 CA M -0.4670 5.2050 6.0130 0.2110

45 44 C15 CA M 0.2220 6.2700 6.6640 -0.1210

46 45 H18 HA E -0.2480 6.8800 7.2370 0.1180

47 45 C17 CA M 1.5590 6.4000 6.4540 -0.3030

48 47 H20 HA E 2.0270 7.1120 6.8950 0.1770

49 47 C20 CA M 2.2930 5.5330 5.6120 0.1880

50 49 C23 CA M 3.6660 5.6970 5.3240 -0.3030

51 50 H23 HA E 4.1670 6.3830 5.7720 0.1770

52 50 C22 CA M 4.2950 4.8900 4.4120 -0.1210

53 52 H22 HA E 5.2320 5.0100 4.2330 0.1180

FODTIBg

1 0 O2 O M 2.8970 6.0950 1.3370 -0.5560

2 1 C5 C M 2.2780 6.9200 1.9930 0.5550

3 2 N1 NA M 1.0080 6.7090 2.4240 -0.4740

4 3 H1 H E 0.5530 5.7460 2.1440 0.3360

5 3 C1 C M 0.2180 7.5940 3.1410 0.5980

6 5 O1 O E -0.9450 7.2950 3.3770 -0.5560

7 5 C2 CM M 0.8620 8.7990 3.5510 -0.0030

8 7 C4 CT 3 0.0830 9.7660 4.4190 -0.2660

9 8 H3 HC E -0.7900 9.4080 4.5960 0.0910

10 8 H4 HC E 0.5490 9.8970 5.2490 0.0910

11 8 H5 HC E -0.0010 10.6060 3.9630 0.0910

12 7 C3 CM M 2.0930 9.0190 3.1100 -0.2960

13 12 H2 H4 E 2.5140 9.8480 3.3460 0.2510

14 12 N2 N* M 2.8140 8.1280 2.3250 -0.0150

15 14 C6 CT M 4.1660 8.5000 1.8220 -0.0160

16 15 H6 H1 E 4.0820 9.2940 1.2880 0.0510

17 15 H7 H1 E 4.4950 7.7820 1.2750 0.0510

18 15 C7 CT M 5.1350 8.7560 2.8820 -0.0040

19 18 H8 HC E 4.8680 9.5360 3.3720 0.0270

20 18 H9 HC E 5.1640 8.0010 3.4730 0.0270

21 18 C8 CT M 6.5520 8.9880 2.2780 0.0020

22 21 H10 HC E 6.4970 9.6550 1.5910 0.0180

23 21 H11 HC E 6.8750 8.1660 1.9030 0.0180

24 21 C9 CT M 7.4340 9.4180 3.2710 -0.0990

25 24 H12 HC E 8.3000 9.5710 2.8870 0.0270

26 24 H13 HC E 7.5010 8.7450 3.9530 0.0270

27 24 H14 HC E 7.1060 10.2330 3.6600 0.0270

FUCVAAh

1 0 O11 O M 4.0660 8.1650 4.6250 -0.6550

2 1 C15 C M 4.2000 7.1050 5.1900 0.7980

3 2 O12 OH S 5.1540 6.2980 5.0820 -0.6490

4 3 H22 HO E 5.9450 6.5960 4.6610 0.4440

5 2 C13 CT M 3.0810 6.6720 6.1700 -0.0230

6 5 H20 H1 E 3.0600 7.3830 6.8150 0.0760

7 5 O9 OS M 3.3320 5.4450 6.8290 -0.2890

8 7 C11 CT M 4.1450 5.5290 7.9670 0.4770

9 8 H16 H1 E 3.8210 6.1640 8.5600 -0.0730

10 8 H17 H1 E 5.0360 5.7560 7.6860 -0.0730

11 8 C9 CT M 4.1930 4.1290 8.5880 -0.2190

12 11 H12 H1 E 3.2930 3.8980 8.8070 0.1060

13 11 H13 H1 E 4.6680 4.2410 9.4380 0.1060

14 11 O7 OS M 4.7650 3.2480 7.8420 -0.3120

15 14 C7 CT M 4.7700 1.9730 8.4370 -0.2190

16 15 H8 H1 E 5.0210 2.0480 9.3670 0.1060

17 15 H9 H1 E 5.4930 1.4580 8.0130 0.1060

18 15 C5 CT M 3.5490 1.1480 8.3620 0.4770

19 18 H3 H1 E 2.8310 1.6110 8.7970 -0.0730

20 18 H4 H1 E 3.6590 0.3000 8.7580 -0.0730

21 18 O1 OS M 3.2110 0.9770 6.9950 -0.2890

22 21 C1 CT M 2.2100 0.0000 6.7070 -0.0230

23 22 C3 C B 0.8460 0.3740 7.2980 0.7980

24 23 O3 OH S 0.0150 -0.5920 7.2130 -0.6490

25 24 H5 HO E -0.3990 -0.6190 7.6390 0.4440

26 23 O4 O E 0.6270 1.4670 7.7270 -0.6550

27 22 H1 H1 E 2.4490 -0.8240 7.1210 0.0760

28 22 C2 CT M 2.1660 -0.1910 5.2290 0.0550

29 28 C4 C B 3.5110 -0.7040 4.7110 0.8700

30 29 O5 O E 3.8650 -1.8320 5.0000 -0.8140

31 29 O6 O E 4.1830 0.1300 4.0120 -0.8140

32 28 H2 H1 E 1.5270 -0.8370 4.9640 0.0140

33 28 O2 OS M 1.7660 1.0550 4.6730 -0.2810

34 33 C6 CT M 1.2770 0.9820 3.3320 0.1210

35 34 H6 H1 E 0.5200 0.3910 3.2880 0.0280

36 34 H7 H1 E 1.9460 0.6610 2.7430 0.0280

37 34 C8 CT M 0.8630 2.3170 2.9050 0.0410

38 37 H10 H1 E 0.2070 2.6820 3.5120 0.0290

39 37 H11 H1 E 0.4340 2.2850 2.0300 0.0290

40 37 O8 OS M 1.9450 3.2240 2.8160 -0.3490

41 40 C10 CT M 1.6220 4.5270 2.3870 0.0410

42 41 H14 H1 E 0.9440 4.4680 1.6990 0.0290

43 41 H15 H1 E 2.3840 4.9180 2.0560 0.0290

44 41 C12 CT M 1.0820 5.4780 3.3680 0.1210

45 44 H18 H1 E 0.1940 5.1930 3.7000 0.0280

46 44 H19 H1 E 0.9570 6.3530 3.0080 0.0280

47 44 O10 OS M 1.9950 5.5120 4.4580 -0.2810

48 47 C14 CT M 1.7690 6.5110 5.4310 0.0550

49 48 H21 H1 E 1.4500 7.3050 5.0280 0.0140

50 48 C16 C M 0.6100 6.1940 6.3770 0.8700

51 50 O14 O E 0.5160 6.9710 7.3610 -0.8140

52 50 O13 O M -0.1550 5.3100 6.1010 -0.8140

FUCVAAg

1 0 N1 N3 M 3.8030 3.1690 5.0320 -0.0920

2 1 H3 H E 3.6830 2.4450 5.6990 0.3250

3 1 H4 H E 3.2660 2.9880 4.3180 0.3250

4 1 H5 H E 3.5390 3.9740 5.4710 0.3250

5 1 N2 NT M 5.1160 3.2110 4.4940 -0.7190

6 5 H1 H E 5.1440 2.6870 3.6310 0.4180

7 5 H2 H E 5.3770 4.1700 4.3150 0.4180

GAMBIFh

1 0 O9 O M 4.4650 1.0580 14.6700 -0.5270

2 1 C22 C M 3.7640 1.3720 13.6300 0.4710

3 2 C20 CA M 4.2740 1.1870 12.3030 -0.0450

4 3 C23 CA S 3.4610 1.4070 11.2110 -0.1790

5 4 H28 HA E 3.8470 1.2300 10.1900 0.1320

6 3 C18 CT M 5.6910 0.7660 12.0960 0.0690

7 6 H24 H1 E 5.9550 1.0920 11.2630 0.0620

8 6 H25 H1 E 6.2200 1.2650 12.8720 0.0620

9 6 O7 OS M 5.7850 -0.6750 12.1000 -0.3930

10 9 C16 CT M 6.9300 -1.1670 11.5220 0.0330

11 10 H20 H1 E 7.6860 -0.4600 11.2810 0.0730

12 10 H21 H1 E 7.1740 -1.4490 12.7290 0.0730

13 10 C14 CT M 7.1680 -2.4720 11.3860 0.0810

14 13 H16 H1 E 8.0260 -2.8170 11.0840 0.0690

15 13 H17 H1 E 7.3310 -2.5640 12.1570 0.0690

16 13 O5 OS M 6.3200 -3.4530 11.9050 -0.4440

17 16 C12 CT M 6.3230 -4.6500 11.2540 0.0690

18 17 H12 H1 E 7.5450 -4.7610 11.1740 0.0530

19 17 H13 H1 E 6.8270 -4.8410 10.6730 0.0530

20 17 C10 CT M 5.6450 -5.7160 11.8420 0.1280

21 20 H8 H1 E 5.5360 -6.5430 11.2630 0.0920

22 20 H9 H1 E 6.2720 -6.0370 12.4790 0.0920

23 20 O1 OS M 4.4140 -5.3800 12.4610 -0.4710

24 23 C7 CT M 3.7770 -6.5000 13.0720 -0.0270

25 24 H4 H1 E 3.5570 -7.1640 12.3710 0.1180

26 24 H5 H1 E 4.3460 -7.0260 13.7120 0.1180

27 24 C4 CA M 2.5310 -6.0590 13.7550 -0.0270

28 27 C6 CA S 2.4950 -5.7400 15.1170 -0.0760

29 28 C9 C B 3.7600 -5.8200 15.9430 0.8000

30 29 O3 O E 4.6260 -4.9560 15.7610 -0.7770

31 29 O4 O E 3.8080 -6.7140 16.7730 -0.7770

32 27 C2 CA M 1.3450 -6.0070 13.0200 -0.0810

33 32 H2 HA E 1.4630 -6.2780 11.9420 0.1240

34 32 C1 CA M 0.1560 -5.6490 13.6120 -0.3230

35 34 H1 HA E -0.6660 -5.6810 13.0690 0.1450

36 34 C3 CA M 0.1420 -5.3410 14.9600 -0.0810

37 36 H3 HA E -0.7270 -5.0830 15.4280 0.1240

38 36 C5 CA M 1.2990 -5.3640 15.7070 -0.0270

39 38 C8 CT M 1.2320 -4.9700 17.1720 -0.0270

40 39 H6 H1 E 2.1290 -5.2090 17.7530 0.1180

41 39 H7 H1 E 0.4100 -5.3590 17.5920 0.1180

42 39 O2 OS M 1.1430 -3.5870 17.2570 -0.4710

43 42 C11 CT M 1.1010 -3.0890 18.5840 0.1280

44 43 H10 H1 E 2.0490 -3.0360 18.8790 0.0920

45 43 H11 H1 E 0.5960 -3.6340 19.0930 0.0920

46 43 C13 CT M 0.4100 -1.7800 18.6020 0.0690

47 46 H14 H1 E 0.1930 -1.4490 19.4690 0.0530

48 46 H15 H1 E -0.5250 -1.8740 18.2710 0.0530

49 46 O6 OS M 1.1530 -0.8060 17.8790 -0.4440

50 49 C15 CT M 0.4080 0.4040 17.8290 0.0810

51 50 H18 H1 E 0.1500 0.5860 18.7720 0.0690

52 50 H19 H1 E -0.3500 0.3450 17.3590 0.0690

53 50 C17 CT M 1.1860 1.4850 17.1880 0.0330

54 53 H22 H1 E 2.0960 1.5410 17.7170 0.0730

55 53 H23 H1 E 0.7590 2.2880 17.1800 0.0730

56 53 O8 OS M 1.4310 1.1410 15.8270 -0.3930

57 56 C19 CT M 1.9350 2.2930 15.1330 0.0690

58 57 H26 H1 E 2.7360 2.7710 15.6430 0.0620

59 57 H27 H1 E 1.1620 2.9090 15.0530 0.0620

60 57 C21 CA M 2.4380 1.8960 13.7600 -0.0450

61 60 C24 CA M 1.6360 2.0940 12.6590 -0.1790

62 61 H29 HA E 0.6320 2.4610 12.8000 0.1320

63 61 C25 CA M 2.1380 1.8200 11.3760 0.1710

64 63 N1 N1 M 1.3680 1.9210 10.2190 -0.1940

65 64 N2 N1 M 0.2060 2.4120 10.3980 -0.2240

66 65 C26 CA M -0.6380 2.3150 9.2820 0.1890

67 66 C27 CA M -0.6510 1.1830 8.4470 -0.0520

68 67 H30 HA E 0.3110 0.3100 8.5280 0.0500

69 67 C29 CA M -1.6620 0.9990 7.5190 -0.2650

70 69 H31 HA E -1.7950 0.0920 6.9190 0.1900

71 69 C31 CA M -2.6440 1.9650 7.4100 0.0010

72 71 N4 NO B -3.7380 1.7640 6.4540 0.7300

73 72 O12 O E -4.5120 2.6880 6.2460 -0.5070

74 72 O13 O E -3.8140 0.6670 5.9140 -0.5070

75 71 C30 CA M -2.6430 3.1210 8.1630 -0.1640

76 75 H32 HA E -3.2880 3.8180 7.9200 0.1820

77 75 C28 CA M -1.6390 3.2680 9.0930 -0.1220

78 77 N3 NO M -1.6090 4.4920 9.8880 0.7670

79 78 O11 O E -2.6910 4.9960 10.1630 -0.4940

80 78 O10 O M -0.5590 4.9770 10.1870 -0.4940

GAMBIFg

1 0 N1 N3 M 3.2800 -1.0530 15.8540 -0.1600

2 1 H1 H E 2.7530 -0.8970 16.5370 0.3180

3 1 H2 H E 3.5270 -0.2530 15.4820 0.3180

4 1 C1 CT M 2.5040 -1.8470 14.8420 -0.0380

5 4 H3 HP E 1.7700 -1.3340 14.6060 0.1500

6 4 H4 HP E 2.1850 -2.6910 15.3210 0.1500

7 4 C3 CT M 3.3820 -2.2310 13.6590 -0.0380

8 7 H7 HP E 3.6290 -1.4830 13.1220 0.1500

9 7 H8 HP E 2.8980 -2.8060 13.0330 0.1500

10 7 N2 N3 M 4.6420 -2.8690 14.1200 -0.1600

11 10 H11 H E 5.1670 -2.9320 13.5160 0.3180

12 10 H12 H E 4.5310 -3.6340 14.5170 0.3180

13 10 C4 CT M 5.3820 -2.0160 15.0820 -0.0380

14 13 H9 HP E 5.5770 -1.1040 14.6060 0.1500

15 13 H10 HP E 6.2510 -2.5640 15.3750 0.1500

16 13 C2 CT M 4.5080 -1.7470 16.2900 -0.0380

17 16 H5 HP E 4.8850 -1.1270 16.9840 0.1500

18 16 H6 HP E 4.1960 -2.5760 16.7160 0.1500

GIGKEMh

1 0 C11 CT M 4.6800 -0.2120 6.6890 0.0340

2 1 H13 H1 E 3.8650 -0.1260 6.1900 0.0280

3 1 H14 H1 E 5.0530 -1.0850 6.5480 0.0280

4 1 C8 CT M 4.3610 -0.0800 8.1370 0.2230

5 4 H8 H1 E 5.1150 -0.3560 8.6600 0.0000

6 4 H9 H1 E 3.6050 -0.6350 8.3460 0.0000

7 4 O2 OS M 4.0400 1.2950 8.4480 -0.3860

8 7 C3 CT M 3.6550 1.4930 9.8160 0.1320

9 8 H3 H1 E 2.7930 1.0980 9.9710 0.0370

10 8 H4 H1 E 4.3030 1.0830 10.3940 0.0370

11 8 C1 CT M 3.5980 2.9470 10.0900 0.1260

12 11 H1 H1 E 4.4350 3.3600 9.8670 0.0350

13 11 H2 H1 E 3.4000 3.1000 11.0170 0.0350

14 11 O1 OS M 2.5380 3.4870 9.2600 -0.3430

15 14 C2 CA M 2.2970 4.8440 9.3750 0.2160

16 15 C5 CA B 3.0070 5.7220 10.0900 -0.1870

17 16 C7 CA B 2.6700 7.0770 10.0880 -0.2020

18 17 C10 CA B 1.5490 7.4850 9.4230 -0.2020

19 18 C6 CA S 0.7590 6.6230 8.6990 -0.1870

20 19 H6 HA E -0.0170 6.9350 8.2290 0.1420

21 18 H12 HA E 1.3100 8.4140 9.4630 0.1590

22 17 H7 HA E 3.2250 7.7120 10.5470 0.1590

23 16 H5 HA E 3.7560 5.4150 10.6080 0.1420

24 15 C4 CA M 1.1360 5.2610 8.6840 0.2160

25 24 O3 OS M 0.4690 4.2900 7.9910 -0.3430

26 25 C9 CT M -0.7990 4.4700 7.5520 0.1260

27 26 H10 H1 E -1.3410 4.7540 8.2930 0.0350

28 26 H11 H1 E -0.8010 5.1490 6.8740 0.0350

29 26 C12 CT M -1.3510 3.2920 7.0090 0.1320

30 29 H15 H1 E -1.4260 2.6420 7.7130 0.0370

31 29 H16 H1 E -2.2250 3.5000 6.6710 0.0370

32 29 O4 OS M -0.5780 2.7170 5.9480 -0.3860

33 32 C15 CT M -0.8320 1.3770 5.6850 0.2230

34 33 H21 H1 E -1.5550 1.1060 6.2540 0.0000

35 33 H22 H1 E -0.0420 0.8810 5.9140 0.0000

36 33 C18 CT M -1.1490 1.0480 4.4670 0.0340

37 36 H25 H1 E -1.4570 0.1400 4.5170 0.0280

38 36 H26 H1 E -1.8780 1.6200 4.2150 0.0280

39 36 N2 NT M -0.1800 1.0960 3.3280 -0.2570

40 39 C22 CT 3 0.5060 -0.1940 3.1820 -0.1520

41 40 C26 C B 1.9040 -0.1040 2.8670 0.9160

42 41 O10 O E 2.6820 0.6560 3.3080 -0.6100

43 41 O11 OS S 2.2220 -1.0930 1.9600 -0.5580

44 43 C29 CT 3 3.6410 -1.3020 1.5930 0.3340

45 44 C33 CT 3 3.9640 -0.7250 0.4940 -0.0870

46 45 H49 HC E 4.8940 -0.9260 0.3660 0.0240

47 45 H50 HC E 3.8230 0.2190 0.3860 0.0240

48 45 H51 HC E 3.4430 -1.2010 -0.1570 0.0240

49 44 H44 H1 E 4.1930 -0.9560 2.2970 -0.0060

50 44 H45 H1 E 3.7980 -2.2440 1.4940 -0.0060

51 40 H33 H1 E 0.0720 -0.6890 2.4850 0.0650

52 40 H34 H1 E 0.4190 -0.6690 4.0120 0.0650

53 39 C21 CT M -0.8000 1.4310 2.0720 0.0340

54 53 H31 H1 E -1.3180 0.6740 1.7880 0.0280

55 53 H32 H1 E -1.3820 2.1820 2.2130 0.0280

56 53 C25 CT M 0.1730 1.7840 0.9470 0.2230

57 56 H40 H1 E -0.3100 1.9290 0.1300 0.0000

58 56 H41 H1 E 0.8050 1.0720 0.8230 0.0000

59 56 O9 OS M 0.8470 2.9530 1.3200 -0.3860

60 59 C28 CT M 1.5270 3.5660 0.2410 0.1320

61 60 H42 H1 E 2.3580 3.1090 0.0860 0.0370

62 60 H43 H1 E 0.9820 3.5140 -0.5500 0.0370

63 60 C32 CT M 1.8020 4.9900 0.5470 0.1260

64 63 H47 H1 E 1.0000 5.3990 0.8830 0.0350

65 63 H48 H1 E 2.0840 5.4440 -0.2490 0.0350

66 63 O12 OS M 2.8160 5.0840 1.5250 -0.3430

67 66 C30 CA M 3.1720 6.3010 2.0170 0.2160

68 67 C34 CA B 2.4050 7.4690 1.7190 -0.1870

69 68 C36 CA B 2.8340 8.6870 2.2890 -0.2020

70 69 C35 CA B 3.9130 8.7350 3.0920 -0.2020

71 70 C31 CA S 4.7030 7.6070 3.3830 -0.1870

72 71 H46 HA E 5.4960 7.6590 3.9220 0.1420

73 70 H53 HA E 4.1510 9.5720 3.4980 0.1590

74 69 H54 HA E 2.3560 9.4960 2.0940 0.1590

75 68 H52 HA E 1.6300 7.4250 1.1540 0.1420

76 67 C27 CA M 4.2680 6.4060 2.8290 0.2160

77 76 O8 OS M 4.9020 5.2090 3.0150 -0.3430

78 77 C23 CT M 6.2390 5.2200 3.5710 0.1260

79 78 H35 H1 E 6.2000 5.4830 4.4930 0.0350

80 78 H36 H1 E 6.7820 5.8430 3.0810 0.0350

81 78 C19 CT M 6.8060 3.9050 3.4740 0.1320

82 81 H27 H1 E 6.8110 3.6480 2.5490 0.0370

83 81 H28 H1 E 7.7070 3.9390 3.8020 0.0370

84 81 O5 OS M 6.0870 2.9150 4.2150 -0.3860

85 84 C16 CT M 6.6950 1.6250 4.0300 0.2230

86 85 H23 H1 E 6.7560 1.4290 3.0920 0.0000

87 85 H24 H1 E 7.5760 1.6260 4.4140 0.0000

88 85 C13 CT M 5.8350 0.5890 4.7250 0.0340

89 88 H17 H1 E 6.2460 -0.2690 4.5950 0.0280

90 88 H18 H1 E 4.9700 0.5950 4.3100 0.0280

91 88 N1 NT M 5.6430 0.7850 6.1700 -0.2570

92 91 C14 CT M 6.8280 0.9120 6.9760 -0.1520

93 92 H19 H1 E 7.3470 1.6330 6.6120 0.0650

94 92 H20 H1 E 6.5480 1.1370 7.8650 0.0650

95 92 C17 C M 7.7660 -0.3210 7.0840 0.9160

96 95 O6 O E 7.5640 -1.3730 6.5370 -0.6100

97 95 O7 OS M 8.7510 -0.0290 7.8720 -0.5580

98 97 C20 CT M 9.6620 -1.1170 8.1960 0.3340

99 98 H29 H1 E 10.1680 -1.3500 7.4150 -0.0060

100 98 H30 H1 E 9.1620 -1.8800 8.4940 -0.0060

101 98 C24 CT M 10.5230 -0.7170 9.2140 -0.0870

102 101 H37 HC E 11.1860 -1.3150 9.5670 0.0240

103 101 H38 HC E 9.8560 -0.5500 9.8850 0.0240

104 101 H39 HC E 10.9390 0.1100 8.9640 0.0240

GIGKEMg

1 0 N1 N* M 3.6800 4.2000 6.2740 0.1040

2 1 C1 CA M 4.8000 3.9350 6.9380 -0.0250

3 2 H1 H4 E 5.0810 3.0200 7.0180 0.2150

4 2 C4 CA M 5.5510 4.8930 7.5060 -0.0980

5 4 H4 HA E 6.3190 4.6610 8.0350 0.2100

6 4 C8 CA M 5.2000 6.2230 7.3200 -0.0020

7 6 H8 HA E 5.7320 6.9370 7.6800 0.2150

8 6 C5 CA M 4.0470 6.4900 6.5920 -0.1120

9 8 H5 HA E 3.7650 7.3990 6.4680 0.1880

10 8 C2 CA M 3.2980 5.4720 6.0400 0.0770

11 10 C6 CA M 2.0790 5.7240 5.2700 0.0770

12 11 C9 CA M 1.4080 6.9420 5.3140 -0.1120

13 12 H9 HA E 1.7390 7.6530 5.8680 0.1880

14 12 C10 CA M 0.2580 7.1290 4.5750 -0.0020

15 14 H10 HA E -0.1910 7.9770 4.5900 0.2150

16 14 C12 CA M -0.2270 6.1170 3.8130 -0.0980

17 16 H12 HA E -1.0210 6.2470 3.2900 0.2100

18 16 C11 CA M 0.4080 4.9120 3.8000 -0.0250

19 18 H11 H4 E 0.0320 4.1830 3.3010 0.2150

20 18 N2 N* M 1.5630 4.7300 4.4800 0.1040

21 20 C7 CT M 2.2770 3.4580 4.4030 -0.0530

22 21 H6 H1 E 3.0000 3.5400 3.7780 0.1400

23 21 H7 H1 E 1.6710 2.7750 4.1050 0.1400

24 21 C3 CT M 2.8250 3.0810 5.7420 -0.0530

25 24 H2 H1 E 3.3530 2.2840 5.6610 0.1400

26 24 H3 H1 E 2.0980 2.9240 6.3490 0.1400

GIKKEQh

1 0 O1 O M 3.6460 -1.8010 0.8150 -0.5850

2 1 C1 C M 3.4600 -1.6690 -0.4540 0.4600

3 2 C2 CA M 3.8300 -0.4540 -1.0630 -0.1170

4 3 C4 CA M 3.7390 -0.3500 -2.3880 -0.2150

5 4 H1 HA E 4.0470 0.4550 -2.7850 0.1730

6 4 C8 CA M 3.2100 -1.3600 -3.2150 -0.0400

7 6 N1 NO B 3.1450 -1.2870 -4.6470 0.7130

8 7 O4 O E 2.6810 -2.2390 -5.2900 -0.5440

9 7 O5 O E 3.4790 -0.2080 -5.1760 -0.5440

10 6 C6 CA M 2.7180 -2.4960 -2.5910 -0.2150

11 10 H4 HA E 2.3240 -3.1680 -3.1310 0.1730

12 10 C3 CA M 2.7880 -2.6600 -1.2270 -0.1170

13 12 C7 CT M 2.2060 -3.7810 -0.5020 -0.0320

14 13 H5 H1 E 2.8580 -4.1650 0.0710 0.0630

15 13 H6 H1 E 1.8960 -4.4420 -1.1130 0.0630

16 13 O3 OS M 1.0930 -3.3190 0.2910 -0.2520

17 16 C10 CT M 0.5820 -4.2810 1.2360 0.0240

18 17 H9 H1 E 1.2870 -4.5520 1.8130 0.0440

19 17 H10 H1 E 0.2510 -5.0350 0.7640 0.0440

20 17 C12 CT M -0.4960 -3.7050 2.0390 0.0640

21 20 H13 H1 E -0.9040 -4.3860 2.5590 0.0280

22 20 H14 H1 E -1.1450 -3.3130 1.4640 0.0280

23 20 O7 OS M 0.0380 -2.7230 2.8820 -0.2700

24 23 C14 CT M -0.9270 -2.2170 3.7880 0.0880

25 24 H17 H1 E -1.2410 -2.9270 4.3330 0.0250

26 24 H18 H1 E -1.6530 -1.8530 3.2940 0.0250

27 24 C16 CT M -0.3350 -1.1500 4.6530 0.0840

28 27 H21 H1 E 0.4230 -1.4980 5.1070 0.0260

29 27 H22 H1 E -0.9810 -0.8670 5.2900 0.0260

30 27 O8 OS M 0.0540 -0.0490 3.8520 -0.2920

31 30 C15 CT M 0.4880 1.0730 4.5560 0.0840

32 31 H19 H1 E 1.2310 0.8270 5.0930 0.0260

33 31 H20 H1 E -0.2200 1.3800 5.1090 0.0260

34 31 C13 CT M 0.8960 2.1730 3.6410 0.0880

35 34 H15 H1 E 1.1390 2.9370 4.1500 0.0250

36 34 H16 H1 E 0.1720 2.3940 3.0640 0.0250

37 34 O6 OS M 1.9730 1.7710 2.8840 -0.2700

38 37 C11 CT M 2.3500 2.8060 2.0310 0.0640

39 38 H11 H1 E 2.5720 3.5730 2.5450 0.0280

40 38 H12 H1 E 1.6290 3.0140 1.4460 0.0280

41 38 C9 CT M 3.5330 2.3670 1.2350 0.0240

42 41 H7 H1 E 4.2220 2.0870 1.8260 0.0440

43 41 H8 H1 E 3.8460 3.0980 0.7120 0.0440

44 41 O2 OS M 3.1800 1.2800 0.3680 -0.2520

45 44 C5 CT M 4.3080 0.7280 -0.2900 -0.0320

46 45 H2 H1 E 4.9580 0.4610 0.3510 0.0630

47 45 H3 H1 E 4.6860 1.3690 -0.8760 0.0630

GIKKEQg

1 0 N1 N3 M 1.1660 -0.5630 1.3460 -0.3570

2 1 H1 H E 1.0630 -0.5600 2.2910 0.3580

3 1 H2 H E 1.3040 -1.4500 1.0430 0.3580

4 1 H3 H E 1.9130 -0.0260 1.1090 0.3580

5 1 C1 CT M -0.0480 -0.0130 0.7220 -0.0120

6 5 H4 HP E -0.1820 0.8750 1.0330 0.1480

7 5 H5 HP E -0.7920 -0.5490 0.9680 0.1480

8 5 C2 CT M 0.0480 0.0130 -0.7220 -0.0120

9 8 H6 HP E 0.1820 -0.8750 -1.0330 0.1480

10 8 H7 HP E 0.7920 0.5490 -0.9680 0.1480

11 8 N2 N3 M -1.1660 0.5630 -1.3460 -0.3570

12 11 H8 H E -1.0630 0.5600 -2.2910 0.3580

13 11 H9 H E -1.3040 1.4500 -1.0430 0.3580

14 11 H10 H E -1.9130 0.0260 -1.1090 0.3580

GIXNOQh

1 0 C16 CA M 4.9780 -3.0790 4.4740 -0.1720

2 1 C13 CA B 5.2300 -1.7170 4.3130 -0.1190

3 2 C8 CA S 6.2210 -1.3360 3.3830 -0.1350

4 3 H2 HA E 6.3990 -0.4140 3.2420 0.1390

5 2 H5 HA E 4.7520 -1.0680 4.8180 0.1430

6 1 H6 HA E 4.3270 -3.3790 5.0930 0.1510

7 1 C11 CA M 5.6890 -3.9920 3.7130 -0.1320

8 7 H4 HA E 5.4980 -4.9140 3.8390 0.1370

9 7 C7 CA M 6.6650 -3.6620 2.7680 0.0100

10 9 C12 CT 3 7.3900 -4.7460 1.9410 0.2610

11 10 C17 CT 3 8.7940 -4.9990 2.4820 -0.1910

12 11 H7 HC E 9.3050 -4.2000 2.4250 0.0440

13 11 H8 HC E 9.2130 -5.6810 1.9740 0.0440

14 11 H9 HC E 8.7380 -5.2710 3.3920 0.0440

15 10 C18 CT 3 7.4480 -4.3820 0.4250 -0.1910

16 15 H10 HC E 6.5810 -4.1380 0.1240 0.0440

17 15 H11 HC E 7.7540 -5.1320 -0.0700 0.0440

18 15 H12 HC E 8.0450 -3.6540 0.2970 0.0440

19 10 C19 CT 3 6.6530 -6.0910 2.0280 -0.1910

20 19 H13 HC E 6.6480 -6.3940 2.9260 0.0440

21 19 H14 HC E 7.0950 -6.7290 1.4800 0.0440

22 19 H15 HC E 5.7590 -5.9810 1.7250 0.0440

23 9 C4 CA M 6.9280 -2.2730 2.6800 -0.0710

24 23 N1 N* M 7.9610 -1.7320 1.8040 0.0250

25 24 C3 C S 7.6540 -1.0830 0.6000 0.4780

26 25 O2 O E 6.5740 -1.0470 0.0620 -0.4820

27 24 C1 C M 9.2590 -1.4500 2.2480 0.4780

28 27 O1 O E 9.7190 -1.7590 3.3350 -0.4820

29 27 C2 CB M 9.9080 -0.6840 1.1420 -0.0690

30 29 C6 CA S 11.2130 -0.2090 1.0470 -0.0880

31 30 H1 HA E 11.8960 -0.3280 1.6980 0.1770

32 29 C5 CB M 8.9510 -0.4720 0.1460 -0.0690

33 32 C9 CA M 9.2270 0.2090 -1.0470 -0.0880

34 33 H3 HA E 8.5440 0.3280 -1.6980 0.1770

35 33 C14 CB M 10.5320 0.6840 -1.1420 -0.0690

36 35 C20 C S 11.1810 1.4500 -2.2480 0.4780

37 36 O4 O E 10.7210 1.7590 -3.3350 -0.4820

38 35 C10 CB M 11.4890 0.4720 -0.1460 -0.0690

39 38 C15 C M 12.7860 1.0830 -0.6000 0.4780

40 39 O3 O E 13.8660 1.0470 -0.0620 -0.4820

41 39 N2 N* M 12.4790 1.7320 -1.8040 0.0250

42 41 C21 CA M 13.5120 2.2730 -2.6800 -0.0710

43 42 C22 CA M 13.7750 3.6620 -2.7680 0.0100

44 43 C25 CT 3 13.0500 4.7460 -1.9410 0.2610

45 44 C28 CT 3 11.6460 4.9990 -2.4820 -0.1910

46 45 H20 HC E 11.1350 4.2000 -2.4250 0.0440

47 45 H21 HC E 11.2270 5.6810 -1.9740 0.0440

48 45 H22 HC E 11.7020 5.2710 -3.3920 0.0440

49 44 C29 CT 3 12.9920 4.3820 -0.4250 -0.1910

50 49 H23 HC E 13.8590 4.1380 -0.1240 0.0440

51 49 H24 HC E 12.6860 5.1320 0.0700 0.0440

52 49 H25 HC E 12.3950 3.6540 -0.2970 0.0440

53 44 C30 CT 3 13.7870 6.0910 -2.0280 -0.1910

54 53 H26 HC E 13.7920 6.3940 -2.9260 0.0440

55 53 H27 HC E 13.3450 6.7290 -1.4800 0.0440

56 53 H28 HC E 14.6810 5.9810 -1.7250 0.0440

57 43 C24 CA M 14.7510 3.9920 -3.7130 -0.1320

58 57 H17 HA E 14.9420 4.9140 -3.8390 0.1370

59 57 C27 CA M 15.4620 3.0790 -4.4740 -0.1720

60 59 H19 HA E 16.1130 3.3790 -5.0930 0.1510

61 59 C26 CA M 15.2100 1.7170 -4.3130 -0.1190

62 61 H18 HA E 15.6880 1.0680 -4.8180 0.1430

63 61 C23 CA M 14.2190 1.3360 -3.3830 -0.1350

64 63 H16 HA E 14.0410 0.4140 -3.2420 0.1390

GIXNOQg

1 0 O1 OH M 12.9870 -1.5720 -2.2770 -0.5310

2 1 H1 HO E 12.3670 -1.5320 -2.9960 0.3790

3 1 C1 CA M 12.7260 -2.3610 -1.1940 0.2710

4 3 C2 CA M 11.5640 -3.1280 -1.0840 -0.1730

5 4 H2 HA E 10.9260 -3.1260 -1.7880 0.1390

6 4 C4 CA M 11.3530 -3.8790 0.0290 -0.1800

7 6 H4 HA E 10.5760 -4.4260 0.0780 0.1500

8 6 C6 CA M 12.2410 -3.8710 1.1130 -0.1150

9 8 H6 HA E 12.0630 -4.3750 1.8990 0.1220

10 8 C5 CA M 13.4010 -3.0950 0.9960 -0.1800

11 10 H5 HA E 14.0300 -3.0840 1.7110 0.1500

12 10 C3 CA M 13.6440 -2.3440 -0.1390 -0.1730

13 12 H3 HA E 14.4320 -1.8180 -0.2000 0.1390

GIYKOOh

1 0 C16 CA M -2.2040 -1.2980 -7.0830 -0.1720

2 1 C13 CA B -0.9150 -1.0960 -6.6760 -0.1190

3 2 C8 CA S -0.6690 -0.8580 -5.3460 -0.1350

4 3 H2 HA E 0.2230 -0.7080 -5.0580 0.1390

5 2 H5 HA E -0.2000 -1.1230 -7.3060 0.1430

6 1 H6 HA E -2.3950 -1.4690 -7.9990 0.1510

7 1 C11 CA M -3.2200 -1.2540 -6.1680 -0.1320

8 7 H4 HA E -4.1080 -1.3850 -6.4790 0.1370

9 7 C7 CA M -3.0320 -1.0240 -4.7990 0.0100

10 9 C12 CT 3 -4.2500 -0.9730 -3.8720 0.2610

11 10 C17 CT 3 -4.0620 -1.7990 -2.6180 -0.1910

12 11 H7 HC E -3.9340 -2.7130 -2.8460 0.0440

13 11 H8 HC E -4.8060 -1.7090 -2.0460 0.0440

14 11 H9 HC E -3.2730 -1.4940 -2.1520 0.0440

15 10 C18 CT 3 -5.4830 -1.5040 -4.5450 -0.1910

16 15 H10 HC E -5.6590 -1.0360 -5.3380 0.0440

17 15 H11 HC E -6.2280 -1.4340 -3.9520 0.0440

18 15 H12 HC E -5.3540 -2.4380 -4.7480 0.0440

19 10 C19 CT 3 -4.4970 0.4480 -3.4750 -0.1910

20 19 H13 HC E -3.7230 0.7920 -2.9990 0.0440

21 19 H14 HC E -5.2490 0.5260 -2.9170 0.0440

22 19 H15 HC E -4.6130 0.9980 -4.2520 0.0440

23 9 C4 CA M -1.7010 -0.8300 -4.4200 -0.0710

24 23 N1 N* M -1.2910 -0.5920 -3.0510 0.0250

25 24 C3 C S -0.8380 -1.5970 -2.1760 0.4780

26 25 O2 O E -0.8560 -2.7730 -2.4070 -0.4820

27 24 C1 C M -1.0370 0.6780 -2.5310 0.4780

28 27 O1 O E -1.2420 1.7160 -3.0970 -0.4820

29 27 C2 CB M -0.4860 0.4700 -1.1700 -0.0690

30 29 C6 CA S -0.1310 1.4130 -0.2250 -0.0880

31 30 H1 HA E -0.2330 2.3390 -0.4150 0.1770

32 29 C5 CB M -0.3650 -0.8890 -0.9610 -0.0690

33 32 C9 CA M 0.1310 -1.4130 0.2250 -0.0880

34 33 H3 HA E 0.2330 -2.3390 0.4150 0.1770

35 33 C14 CB M 0.4860 -0.4700 1.1700 -0.0690

36 35 C20 C S 1.0370 -0.6780 2.5310 0.4780

37 36 O4 O E 1.2420 -1.7160 3.0970 -0.4820

38 35 C10 CB M 0.3650 0.8890 0.9610 -0.0690

39 38 C15 C M 0.8380 1.5970 2.1760 0.4780

40 39 O3 O E 0.8560 2.7730 2.4070 -0.4820

41 39 N2 N* M 1.2910 0.5920 3.0510 0.0250

42 41 C21 CA M 1.7010 0.8300 4.4200 -0.0710

43 42 C22 CA M 3.0320 1.0240 4.7990 0.0100

44 43 C25 CT 3 4.2500 0.9730 3.8720 0.2610

45 44 C28 CT 3 4.0620 1.7990 2.6180 -0.1910

46 45 H20 HC E 3.9340 2.7130 2.8460 0.0440

47 45 H21 HC E 4.8060 1.7090 2.0460 0.0440

48 45 H22 HC E 3.2730 1.4940 2.1520 0.0440

49 44 C29 CT 3 5.4830 1.5040 4.5450 -0.1910

50 49 H23 HC E 5.6590 1.0360 5.3380 0.0440

51 49 H24 HC E 6.2280 1.4340 3.9520 0.0440

52 49 H25 HC E 5.3540 2.4380 4.7480 0.0440

53 44 C30 CT 3 4.4970 -0.4480 3.4750 -0.1910

54 53 H26 HC E 3.7230 -0.7920 2.9990 0.0440

55 53 H27 HC E 5.2490 -0.5260 2.9170 0.0440

56 53 H28 HC E 4.6130 -0.9980 4.2520 0.0440

57 43 C24 CA M 3.2200 1.2540 6.1680 -0.1320

58 57 H17 HA E 4.1080 1.3850 6.4790 0.1370

59 57 C27 CA M 2.2040 1.2980 7.0830 -0.1720

60 59 H19 HA E 2.3950 1.4690 7.9990 0.1510

61 59 C26 CA M 0.9150 1.0960 6.6760 -0.1190

62 61 H18 HA E 0.2000 1.1230 7.3060 0.1430

63 61 C23 CA M 0.6690 0.8580 5.3460 -0.1350

64 63 H16 HA E -0.2230 0.7080 5.0580 0.1390

GIYKOOg

1 0 N1 NA M -2.9080 2.9670 1.6830 -0.2810

2 1 H1 H E -2.5510 3.6720 2.2060 0.3260

3 1 C1 CW M -3.5290 3.1150 0.4800 -0.1520

4 3 H2 H4 E -3.6730 3.9440 0.0310 0.1900

5 3 C3 C* M -3.9070 1.9150 0.0230 -0.3290

6 5 H3 HA E -4.3630 1.7470 -0.7920 0.1820

7 5 C4 CB M -3.5040 0.9410 0.9720 0.1650

8 7 C2 CN M -2.8840 1.6430 2.0120 0.0830

9 8 C5 CA M -2.3620 1.0290 3.1290 -0.2480

10 9 H4 HA E -1.9750 1.5390 3.8340 0.1610

11 9 C7 CA M -2.4170 -0.3220 3.1950 -0.1270

12 11 H6 HA E -2.0460 -0.7650 3.9550 0.1420

13 11 C8 CA M -2.9960 -1.0600 2.1960 -0.2060

14 13 H7 HA E -3.0080 -2.0060 2.2720 0.1510

15 13 C6 CA M -3.5610 -0.4650 1.0900 -0.2170

16 15 H5 HA E -3.9740 -0.9920 0.4220 0.1610

GOBYOLh

1 0 N1 NT M 5.0880 7.6740 -2.7420 -0.6480

2 1 H1 H E 4.8680 7.4520 -1.9980 0.3380

3 1 C1 CT M 4.9310 6.5230 -3.6330 0.0290

4 3 H2 H1 E 5.1710 6.7810 -4.5250 0.0720

5 3 H3 H1 E 4.0130 6.2390 -3.6170 0.0720

6 3 C3 CT M 5.7960 5.3860 -3.2030 -0.0280

7 6 H6 H1 E 5.6650 4.6380 -3.7910 0.0740

8 6 H7 H1 E 6.7180 5.6540 -3.2290 0.0740

9 6 O1 OS M 5.4430 5.0250 -1.8870 -0.2780

10 9 C5 CT M 6.1570 3.8920 -1.4290 0.0260

11 10 H10 H1 E 6.0230 3.1650 -2.0400 0.0560

12 10 H11 H1 E 7.0910 4.1050 -1.3810 0.0560

13 10 C7 CT M 5.6740 3.4970 -0.0790 0.0260

14 13 H14 H1 E 4.7140 3.4850 -0.0790 0.0560

15 13 H15 H1 E 6.0050 2.6220 0.1320 0.0560

16 13 O3 OS M 6.1260 4.4130 0.8980 -0.2780

17 16 C9 CT M 5.7150 4.0120 2.1970 -0.0280

18 17 H18 H1 E 4.7930 3.7490 2.1750 0.0740

19 17 H19 H1 E 6.2530 3.2740 2.4900 0.0740

20 17 C11 CT M 5.8810 5.1590 3.1420 0.0290

21 20 H22 H1 E 5.6380 4.8770 4.0270 0.0720

22 20 H23 H1 E 6.7980 5.4410 3.1380 0.0720

23 20 N2 NT M 5.0270 6.2870 2.7420 -0.6480

24 23 H26 H E 5.2470 6.5090 1.9980 0.3380

25 23 C12 CT M 5.1840 7.4380 3.6330 0.0290

26 25 H24 H1 E 4.9440 7.1800 4.5250 0.0720

27 25 H25 H1 E 6.1020 7.7220 3.6170 0.0720

28 25 C10 CT M 4.3190 8.5750 3.2030 -0.0280

29 28 H20 H1 E 4.4500 9.3230 3.7910 0.0740

30 28 H21 H1 E 3.3970 8.3070 3.2290 0.0740

31 28 O4 OS M 4.6720 8.9360 1.8870 -0.2780

32 31 C8 CT M 3.9580 10.0690 1.4290 0.0260

33 32 H16 H1 E 3.0240 9.8560 1.3810 0.0560

34 32 H17 H1 E 4.0920 10.7960 2.0400 0.0560

35 32 C6 CT M 4.4410 10.4640 0.0790 0.0260

36 35 H12 H1 E 5.4010 10.4760 0.0790 0.0560

37 35 H13 H1 E 4.1100 11.3390 -0.1320 0.0560

38 35 O2 OS M 3.9890 9.5480 -0.8980 -0.2780

39 38 C4 CT M 4.4000 9.9490 -2.1970 -0.0280

40 39 H8 H1 E 3.8620 10.6870 -2.4900 0.0740

41 39 H9 H1 E 5.3220 10.2120 -2.1750 0.0740

42 39 C2 CT M 4.2340 8.8020 -3.1420 0.0290

43 42 H4 H1 E 4.4770 9.0840 -4.0270 0.0720

44 42 H5 H1 E 3.3170 8.5200 -3.1380 0.0720

GOBYOLg

1 0 C4 CA M 9.5240 9.1050 0.5350 -0.1720

2 1 H4 HA E 10.1820 9.7130 0.8810 0.1840

3 1 C2 CA M 8.9220 9.3580 -0.6660 -0.2100

4 3 H1 HA E 9.1400 10.1580 -1.1500 0.1660

5 3 C1 CA M 8.0010 8.4720 -1.1880 0.3100

6 5 O1 OH S 7.4830 8.7480 -2.3980 -0.5090

7 6 H3 HO E 6.7130 8.2590 -2.5280 0.3880

8 5 C3 CA M 7.6570 7.3450 -0.4680 -0.2100

9 8 H2 HA E 7.0040 6.7330 -0.8170 0.1660

10 8 C5 CA M 8.2510 7.0960 0.7470 -0.1720

11 10 H5 HA E 8.0180 6.3100 1.2470 0.1840

12 10 C6 CA M 9.1830 7.9820 1.2390 0.0550

13 12 N1 NO M 9.7960 7.7150 2.5260 0.7180

14 13 O3 O E 10.6720 8.4720 2.9110 -0.4500

15 13 O2 O M 9.4240 6.7450 3.1640 -0.4500

GOKQUSh

1 0 O1 OH M 11.3250 10.4360 0.6790 -0.5970

2 1 H1 HO E 10.5220 10.9320 0.8070 0.4300

3 1 C1 CA M 11.9760 10.7930 -0.4920 0.2560

4 3 C2 CA M 11.7650 12.0550 -1.0640 -0.0430

5 4 C5 CA S 12.4880 12.3900 -2.2060 -0.2620

6 5 H4 HA E 12.3310 13.3570 -2.6640 0.1800

7 4 C4 CT M 10.7650 13.0460 -0.4880 -0.0240

8 7 H2 HC E 10.7140 12.9080 0.5890 0.0550

9 7 H3 HC E 11.0990 14.0500 -0.7040 0.0550

10 7 C8 CA M 9.3670 12.8660 -1.0560 -0.0430

11 10 C12 CA S 8.4540 11.9760 -0.4920 0.2560

12 11 O2 OH S 8.8110 11.3250 0.6790 -0.5970

13 12 H10 HO E 8.3150 10.5220 0.8070 0.4300

14 10 C11 CA M 8.9830 13.5650 -2.1920 -0.2620

15 14 H8 HA E 9.6880 14.2560 -2.6380 0.1800

16 14 C16 CA M 7.7130 13.3940 -2.8140 -0.0300

17 16 C24 CT 3 7.3680 14.2610 -4.0330 0.3680

18 17 C29 CT 3 8.4210 14.0440 -5.1290 -0.2160

19 18 H27 HC E 8.1480 14.6530 -5.9800 0.0430

20 18 H28 HC E 9.3800 14.3710 -4.7510 0.0430

21 18 H29 HC E 8.4560 13.0110 -5.4040 0.0430

22 17 C30 CT 3 5.9850 13.8660 -4.5670 -0.2160

23 22 H30 HC E 5.7870 14.4870 -5.4550 0.0430

24 22 H31 HC E 6.0180 12.8310 -4.8920 0.0430

25 22 H32 HC E 5.2350 14.0120 -3.8420 0.0430

26 17 C31 CT 3 7.3550 15.7400 -3.6220 -0.2160

27 26 H33 HC E 7.0960 16.3340 -4.4820 0.0430

28 26 H34 HC E 6.6340 15.8850 -2.8300 0.0430

29 26 H35 HC E 8.3400 16.0140 -3.2660 0.0430

30 16 C23 CA M 6.8570 12.4880 -2.2060 -0.2620

31 30 H21 HA E 5.8900 12.3310 -2.6640 0.1800

32 30 C17 CA M 7.1920 11.7650 -1.0640 -0.0430

33 32 C25 CT M 6.2010 10.7650 -0.4880 -0.0240

34 33 H22 HC E 6.3390 10.7140 0.5890 0.0550

35 33 H23 HC E 5.1970 11.0990 -0.7040 0.0550

36 33 C32 CA M 6.3810 9.3670 -1.0560 -0.0430

37 36 C38 CA S 7.2710 8.4540 -0.4920 0.2560

38 37 O4 OH S 7.9220 8.8110 0.6790 -0.5970

39 38 H47 HO E 8.7250 8.3150 0.8070 0.4300

40 36 C37 CA M 5.6820 8.9830 -2.1920 -0.2620

41 40 H45 HA E 4.9910 9.6880 -2.6380 0.1800

42 40 C40 CA M 5.8530 7.7130 -2.8140 -0.0300

43 42 C41 CT 3 4.9860 7.3680 -4.0330 0.3680

44 43 C42 CT 3 5.2030 8.4210 -5.1290 -0.2160

45 44 H48 HC E 4.5940 8.1480 -5.9800 0.0430

46 44 H49 HC E 4.8760 9.3800 -4.7510 0.0430

47 44 H50 HC E 6.2360 8.4560 -5.4040 0.0430

48 43 C43 CT 3 5.3810 5.9850 -4.5670 -0.2160

49 48 H51 HC E 4.7600 5.7870 -5.4550 0.0430

50 48 H52 HC E 6.4160 6.0180 -4.8920 0.0430

51 48 H53 HC E 5.2350 5.2350 -3.8420 0.0430

52 43 C44 CT 3 3.5080 7.3550 -3.6220 -0.2160

53 52 H54 HC E 2.9130 7.0960 -4.4820 0.0430

54 52 H55 HC E 3.3620 6.6340 -2.8300 0.0430

55 52 H56 HC E 3.2340 8.3400 -3.2660 0.0430

56 42 C39 CA M 6.7590 6.8570 -2.2060 -0.2620

57 56 H46 HA E 6.9160 5.8900 -2.6640 0.1800

58 56 C36 CA M 7.4820 7.1920 -1.0640 -0.0430

59 58 C28 CT M 8.4820 6.2010 -0.4880 -0.0240

60 59 H25 HC E 8.5330 6.3390 0.5890 0.0550

61 59 H26 HC E 8.1480 5.1970 -0.7040 0.0550

62 59 C22 CA M 9.8800 6.3810 -1.0560 -0.0430

63 62 C26 CA B 10.2640 5.6820 -2.1920 -0.2620

64 63 C21 CA B 11.5340 5.8530 -2.8140 -0.0300

65 64 C14 CA S 12.3900 6.7590 -2.2060 -0.2620

66 65 H9 HA E 13.3570 6.9160 -2.6640 0.1800

67 64 C27 CT 3 11.8790 4.9860 -4.0330 0.3680

68 67 C33 CT 3 10.8260 5.2030 -5.1290 -0.2160

69 68 H36 HC E 11.0990 4.5940 -5.9800 0.0430

70 68 H37 HC E 9.8670 4.8760 -4.7510 0.0430

71 68 H38 HC E 10.7910 6.2360 -5.4040 0.0430

72 67 C34 CT 3 13.2620 5.3810 -4.5670 -0.2160

73 72 H39 HC E 13.4600 4.7600 -5.4550 0.0430

74 72 H40 HC E 13.2290 6.4160 -4.8920 0.0430

75 72 H41 HC E 14.0120 5.2350 -3.8420 0.0430

76 67 C35 CT 3 11.8920 3.5080 -3.6220 -0.2160

77 76 H42 HC E 12.1510 2.9130 -4.4820 0.0430

78 76 H43 HC E 12.6130 3.3620 -2.8300 0.0430

79 76 H44 HC E 10.9070 3.2340 -3.2660 0.0430

80 63 H24 HA E 9.5590 4.9910 -2.6380 0.1800

81 62 C15 CA M 10.7930 7.2710 -0.4920 0.2560

82 81 O3 OH S 10.4360 7.9220 0.6790 -0.5970

83 82 H20 HO E 10.9320 8.7250 0.8070 0.4300

84 81 C10 CA M 12.0550 7.4820 -1.0640 -0.0430

85 84 C7 CT M 13.0460 8.4820 -0.4880 -0.0240

86 85 H6 HC E 12.9080 8.5330 0.5890 0.0550

87 85 H7 HC E 14.0500 8.1480 -0.7040 0.0550

88 85 C3 CA M 12.8660 9.8800 -1.0560 -0.0430

89 88 C6 CA M 13.5650 10.2640 -2.1920 -0.2620

90 89 H5 HA E 14.2560 9.5590 -2.6380 0.1800

91 89 C9 CA M 13.3940 11.5340 -2.8140 -0.0300

92 91 C13 CT M 14.2610 11.8790 -4.0330 0.3680

93 92 C19 CT 3 13.8660 13.2620 -4.5670 -0.2160

94 93 H14 HC E 14.4870 13.4600 -5.4550 0.0430

95 93 H15 HC E 12.8310 13.2290 -4.8920 0.0430

96 93 H16 HC E 14.0120 14.0120 -3.8420 0.0430

97 92 C20 CT 3 15.7400 11.8920 -3.6220 -0.2160

98 97 H17 HC E 16.3340 12.1510 -4.4820 0.0430

99 97 H18 HC E 15.8850 12.6130 -2.8300 0.0430

100 97 H19 HC E 16.0140 10.9070 -3.2660 0.0430

101 92 C18 CT M 14.0440 10.8260 -5.1290 -0.2160

102 101 H11 HC E 14.6530 11.0990 -5.9800 0.0430

103 101 H12 HC E 14.3710 9.8670 -4.7510 0.0430

104 101 H13 HC E 13.0110 10.7910 -5.4040 0.0430

GOKQUSg

1 0 N1 NC M 8.5140 9.7170 -5.8460 -0.5490

2 1 C1 CA M 9.7520 9.3310 -6.1800 0.2630

3 2 H1 H4 E 9.9060 9.0200 -7.1970 0.0790

4 2 C2 CA M 10.8040 9.2120 -5.2720 -0.2700

5 4 H2 HA E 11.7540 8.8410 -5.5960 0.1410

6 4 C3 CA M 10.5590 9.5130 -3.9310 -0.0200

7 6 H3 HA E 11.3560 9.4310 -3.2010 0.1430

8 6 C4 CA M 9.2760 9.9190 -3.5640 -0.2700

9 8 H4 HA E 9.0720 10.1880 -2.5230 0.1410

10 8 C5 CA M 8.2980 10.0030 -4.5540 0.2630

11 10 H5 H4 E 7.3140 10.3290 -4.2260 0.0790

GUGGUKh

1 0 O6 OH M 2.0000 -1.2840 -9.3380 -0.5110

2 1 H11 HO E 3.3720 -0.9840 -9.7390 0.4260

3 1 C13 CA M 1.2880 -0.2030 -8.9510 0.1550

4 3 C10 CA M 0.3960 -0.2700 -7.8620 0.0360

5 4 C14 CA S -0.3260 0.8610 -7.5000 -0.2780

6 5 H9 HA E -0.9250 0.8150 -6.7890 0.1830

7 4 C8 CT M 0.2430 -1.5350 -7.0430 0.0080

8 7 H5 HC E 0.2600 -2.3020 -7.6360 -0.0030

9 7 H6 HC E -0.6180 -1.5230 -6.5970 -0.0030

10 7 C5 CA M 1.3420 -1.6850 -6.0010 0.0840

11 10 C6 CA S 2.5460 -2.3510 -6.3070 0.1180

12 11 O4 OH S 2.7430 -2.8720 -7.5440 -0.5710

13 12 H7 HO E 2.2770 -2.3750 -8.3750 0.3680

14 10 C3 CA M 1.1710 -1.1300 -4.7370 -0.2810

15 14 H2 HA E 0.3870 -0.6720 -4.5320 0.1980

16 14 C1 CA M 2.1770 -1.2610 -3.7770 -0.0390

17 16 S1 S6 3 2.0770 -0.4290 -2.2310 1.1610

18 17 O1 O E 2.5860 -1.3260 -1.2000 -0.7120

19 17 O2 O E 0.6700 -0.0620 -2.0300 -0.7120

20 17 O3 O E 2.9030 0.7740 -2.3650 -0.7120

21 16 C2 CA M 3.3460 -1.9530 -4.0760 -0.2810

22 21 H1 HA E 4.0000 -2.0470 -3.4230 0.1980

23 21 C4 CA M 3.5530 -2.5030 -5.3320 0.0840

24 23 C7 CT M 4.8800 -3.1640 -5.6520 0.0080

25 24 H3 HC E 4.7740 -3.7730 -6.4010 -0.0030

26 24 H4 HC E 5.1880 -3.6740 -4.8870 -0.0030

27 24 C9 CA M 5.8950 -2.0830 -6.0010 0.0360

28 27 C12 CA S 6.1350 -1.6900 -7.3220 0.1550

29 28 O5 OH S 5.6240 -2.4890 -8.3100 -0.5110

30 29 H10 HO E 5.2330 -1.6760 -9.1110 0.4260

31 27 C11 CA M 6.5130 -1.3730 -4.9650 -0.2780

32 31 H8 HA E 6.3930 -1.6370 -4.0810 0.1830

33 31 C15 CA M 7.3030 -0.2740 -5.2610 -0.0280

34 33 S2 S6 3 8.1230 0.6030 -3.9770 1.1620

35 34 O7 O E 9.5520 0.4270 -4.2050 -0.7200

36 34 O8 O E 7.7150 -0.0140 -2.7070 -0.7200

37 34 O9 O E 7.6770 1.9850 -4.0810 -0.7200

38 33 C19 CA M 7.4760 0.1540 -6.5770 -0.2740

39 38 H12 HA E 7.9920 0.9080 -6.7530 0.1900

40 38 C16 CA M 6.8860 -0.5360 -7.6290 0.0110

41 40 C20 CT M 7.0730 -0.0280 -9.0500 0.0010

42 41 H13 HC E 7.9590 0.3580 -9.1300 -0.0060

43 41 H14 HC E 7.0230 -0.7800 -9.6610 -0.0060

44 41 C23 CA M 6.0390 1.0160 -9.4640 0.0100

45 44 C26 C S 4.7570 0.6210 -9.8770 0.2450

46 45 O13 O E 4.4640 -0.7180 -9.9050 -0.7410

47 44 C25 CA M 6.3520 2.3750 -9.4710 -0.2450

48 47 H18 HA E 7.2040 2.6550 -9.2300 0.1880

49 47 C28 CA M 5.3890 3.3200 -9.8390 -0.1210

50 49 S4 S6 3 5.7610 5.0470 -9.9510 1.1650

51 50 O14 O E 6.8630 5.3150 -9.0330 -0.7210

52 50 O15 O E 6.1100 5.3300 -11.3370 -0.7210

53 50 O16 O E 4.5300 5.7360 -9.5470 -0.7210

54 49 C27 CA M 4.1280 2.9030 -10.2410 -0.2450

55 54 H19 HA E 3.4920 3.5380 -10.4800 0.1880

56 54 C24 CA M 3.7990 1.5580 -10.2930 0.0100

57 56 C21 CT M 2.4340 1.1250 -10.7970 0.0010

58 57 H15 HC E 2.5110 0.2670 -11.2410 -0.0060

59 57 H16 HC E 2.1090 1.7690 -11.4440 -0.0060

60 57 C17 CA M 1.4460 1.0150 -9.6560 0.0110

61 60 C22 CA M 0.7190 2.1270 -9.2640 -0.2740

62 61 H17 HA E 0.8220 2.9270 -9.7250 0.1900

63 61 C18 CA M -0.1670 2.0630 -8.1860 -0.0280

64 63 S3 S6 M -1.0570 3.5000 -7.7390 1.1620

65 64 O11 O E -1.9030 3.8560 -8.8670 -0.7200

66 64 O12 O E -0.0500 4.5290 -7.4670 -0.7200

67 64 O10 O M -1.8220 3.1500 -6.5430 -0.7200

GUGGUKg

1 0 N1 N3 M 5.1590 2.6640 -5.6040 -0.3350

2 1 H1 H E 5.0250 3.1320 -4.7830 0.3540

3 1 H2 H E 6.1960 2.3450 -5.5760 0.3540

4 1 H3 H E 4.8300 3.3790 -6.2450 0.3540

5 1 C1 CT M 4.3160 1.4520 -5.8300 -0.0190

6 5 H4 HP E 4.4490 0.8310 -5.0990 0.1470

7 5 H5 HP E 4.6030 1.0160 -6.6480 0.1470

8 5 C2 CT M 2.8500 1.7780 -5.9380 -0.0190

9 8 H6 HP E 2.7170 2.4240 -6.6500 0.1470

10 8 H7 HP E 2.3610 0.9740 -6.1710 0.1470

11 8 N2 N3 M 2.3080 2.3300 -4.6750 -0.3350

12 11 H8 H E 2.4760 3.0700 -4.4440 0.3540

13 11 H9 H E 1.2920 2.3170 -4.6580 0.3540

14 11 H10 H E 2.5490 1.7140 -3.9250 0.3540

GUQHUVh

1 0 C32 CT M 0.5130 13.0330 22.5680 -0.2588

2 1 H21 HC E 0.5360 13.9410 22.2560 0.0944

3 1 H22 HC E 0.4310 13.0120 23.5240 0.0944

4 1 C29 CT M 1.7030 12.1890 22.0460 0.0072

5 4 H19 HC E 2.5810 12.4600 22.3230 0.0830

6 4 C27 CA M 1.3980 12.2500 20.5420 0.0069

7 6 C25 CA M 2.1960 12.2610 19.4440 -0.1860

8 7 H17 HA E 3.1510 12.2560 19.5400 0.1330

9 7 C23 CA M 1.6060 12.2770 18.1460 0.0252

10 9 C21 CA M 2.4070 12.2560 16.9660 -0.1860

11 10 H15 HA E 3.3650 12.2540 17.0340 0.1330

12 10 C19 CA M 1.8110 12.2410 15.7450 0.0069

13 12 C16 CT M 2.3600 12.1780 14.3160 0.0072

14 13 H11 HC E 3.2760 12.4370 14.1910 0.0830

15 13 C18 CT B 1.2850 13.0330 13.6000 -0.2588

16 15 H13 HC E 1.2630 13.9410 13.9110 0.0944

17 15 H14 HC E 1.3680 13.0120 12.6440 0.0944

18 13 C14 CA M 1.9530 10.8020 13.8000 0.0211

19 18 C12 CA M 2.6930 9.6530 13.5450 -0.2329

20 19 H9 HA E 3.6470 9.6370 13.6470 0.1468

21 19 C9 CA M 1.9910 8.5350 13.1380 0.0156

22 21 C4 CT M 2.4410 7.0940 12.8550 0.0040

23 22 H2 HC E 3.3630 6.9670 12.6190 0.0804

24 22 C8 CT B 1.3890 6.7090 11.7920 -0.2585

25 24 H6 HC E 1.4010 5.7740 11.5770 0.0921

26 24 H7 HC E 1.4580 7.2360 10.9920 0.0921

27 22 C1 CA M 1.9130 6.2820 14.0290 0.0382

28 27 C2 CA M 2.5290 5.6950 15.1020 -0.1575

29 28 H1 HA E 3.4860 5.7080 15.1840 0.1292

30 28 C5 CA M 1.7440 5.0820 16.0660 -0.1778

31 30 H3 HA E 2.1620 4.6560 16.8180 0.1480

32 30 C10 CA M 0.3780 5.0770 15.9570 -0.1778

33 32 H8 HA E -0.1480 4.6480 16.6360 0.1480

34 32 C6 CA M -0.2630 5.6890 14.8710 -0.1575

35 34 H4 HA E -1.2200 5.6910 14.7970 0.1292

36 34 C3 CA M 0.5270 6.2870 13.9100 0.0382

37 36 C7 CT M 0.1810 7.1080 12.6700 0.0040

38 37 H5 HC E -0.6950 6.9860 12.2940 0.0804

39 37 C11 CA M 0.5990 8.5380 13.0180 0.0156

40 39 C13 CA M -0.1410 9.6740 13.3080 -0.2329

41 40 H10 HA E -1.1000 9.6740 13.2560 0.1468

42 40 C15 CA M 0.5630 10.8100 13.6760 0.0211

43 42 C17 CT M 0.0960 12.1890 14.1210 0.0072

44 43 H12 HC E -0.7830 12.4600 13.8450 0.0830

45 43 C20 CA M 0.4000 12.2500 15.6260 0.0069

46 45 C22 CA M -0.3980 12.2610 16.7240 -0.1860

47 46 H16 HA E -1.3530 12.2560 16.6270 0.1330

48 46 C24 CA M 0.1930 12.2770 18.0220 0.0252

49 48 C26 CA M -0.6080 12.2560 19.2020 -0.1860

50 49 H18 HA E -1.5670 12.2540 19.1340 0.1330

51 49 C28 CA M -0.0120 12.2410 20.4230 0.0069

52 51 C30 CT M -0.5620 12.1780 21.8520 0.0072

53 52 H20 HC E -1.4770 12.4370 21.9770 0.0830

54 52 C33 CA M -0.1550 10.8020 22.3680 0.0211

55 54 C35 CA S -0.8950 9.6530 22.6230 -0.2329

56 55 H24 HA E -1.8490 9.6370 22.5210 0.1468

57 54 C31 CA M 1.2360 10.8100 22.4920 0.0211

58 57 C34 CA M 1.9400 9.6740 22.8600 -0.2329

59 58 H23 HA E 2.8980 9.6740 22.9110 0.1468

60 58 C36 CA M 1.1990 8.5380 23.1500 0.0156

61 60 C37 CA M -0.1920 8.5350 23.0300 0.0156

62 61 C39 CT M -0.6420 7.0940 23.3130 0.0040

63 62 H26 HC E -1.5640 6.9670 23.5490 0.0804

64 62 C41 CT M 0.4100 6.7090 24.3760 -0.2585

65 64 H27 HC E 0.3410 7.2360 25.1760 0.0921

66 64 H28 HC E 0.3970 5.7740 24.5910 0.0921

67 64 C38 CT M 1.6180 7.1080 23.4970 0.0040

68 67 H25 HC E 2.4930 6.9860 23.8730 0.0804

69 67 C40 CA M 1.2710 6.2870 22.2570 0.0382

70 69 C42 CA M -0.1140 6.2820 22.1390 0.0382

71 70 C44 CA M -0.7310 5.6950 21.0660 -0.1575

72 71 H30 HA E -1.6870 5.7080 20.9840 0.1292

73 71 C46 CA M 0.0540 5.0820 20.1020 -0.1778

74 73 H32 HA E -0.3640 4.6560 19.3500 0.1480

75 73 C45 CA M 1.4200 5.0770 20.2110 -0.1778

76 75 H31 HA E 1.9460 4.6480 19.5320 0.1480

77 75 C43 CA M 2.0610 5.6890 21.2970 -0.1575

78 77 H29 HA E 3.0180 5.6910 21.3710 0.1292

GUQHUVg

1 0 N1 N1 M 4.1890 8.6860 20.2620 -0.4380

2 1 C1 CZ M 3.2390 8.6970 19.6230 0.3200

3 2 C2 CA M 2.0400 8.7020 18.8340 0.0670

4 3 C3 CA M 2.1200 8.7050 17.4470 -0.1460

5 4 H1 HA E 2.9750 8.7050 17.0120 0.1710

6 4 C5 CA M 0.9770 8.7070 16.7120 -0.1460

7 6 H3 HA E 1.0250 8.7130 15.7530 0.1710

8 6 C7 CA M -0.2420 8.7020 17.3340 0.0670

9 8 C8 CZ S -1.4400 8.6970 16.5450 0.3200

10 9 N2 N1 E -2.3910 8.6860 15.9060 -0.4380

11 8 C6 CA M -0.3220 8.7050 18.7200 -0.1460

12 11 H4 HA E -1.1770 8.7050 19.1560 0.1710

13 11 C4 CA M 0.8210 8.7070 19.4560 -0.1460

14 13 H2 HA E 0.7730 8.7130 20.4150 0.1710

GUQJEHh

1 0 C32 CT M -4.5190 1.1090 8.9030 -0.2588

2 1 H21 HC E -4.7170 0.8360 8.0040 0.0944

3 1 H22 HC E -5.3320 1.2390 9.3980 0.0944

4 1 C29 CT M -3.5650 2.3390 8.9690 0.0072

5 4 H19 HC E -3.8930 3.1610 8.5980 0.0830

6 4 C27 CA M -2.3320 1.7240 8.3000 0.0069

7 6 C25 CA M -1.3570 2.2300 7.4870 -0.1860

8 7 H17 HA E -1.3950 3.1450 7.2000 0.1330

9 7 C23 CA M -0.2790 1.3890 7.0630 0.0252

10 9 C21 CA M 0.7570 1.8620 6.1980 -0.1860

11 10 H15 HA E 0.7310 2.7640 5.8680 0.1330

12 10 C19 CA M 1.7890 1.0280 5.8370 0.0069

13 12 C16 CT M 3.0690 1.2300 5.0090 0.0072

14 13 H11 HC E 3.0760 1.9630 4.3900 0.0830

15 13 C18 CT B 3.2060 -0.2010 4.4110 -0.2588

16 15 H13 HC E 2.4730 -0.4370 3.8360 0.0944

17 15 H14 HC E 4.0410 -0.3380 3.9570 0.0944

18 13 C14 CA M 4.2000 1.1780 6.0430 0.0211

19 18 C12 CA M 4.9920 2.1790 6.5910 -0.2329

20 19 H9 HA E 4.9730 3.0760 6.2490 0.1468

21 19 C9 CA M 5.8180 1.8280 7.6580 0.0156

22 21 C4 CT M 6.6780 2.6720 8.5950 0.0040

23 22 H2 HC E 7.0010 3.5520 8.2460 0.0804

24 22 C8 CT B 7.7650 1.6110 8.9580 -0.2585

25 24 H6 HC E 8.2820 1.3300 8.1990 0.0921

26 24 H7 HC E 8.3450 1.8990 9.6670 0.0921

27 22 C1 CA M 5.9040 2.6930 9.9150 0.0382

28 27 C2 CA M 5.1780 3.6840 10.5500 -0.1575

29 28 H1 HA E 5.1270 4.5690 10.1800 0.1292

30 28 C5 CA M 4.5170 3.3710 11.7440 -0.1778

31 30 H3 HA E 4.0140 4.0470 12.2020 0.1480

32 30 C10 CA M 4.5850 2.0900 12.2700 -0.1778

33 32 H8 HA E 4.1370 1.8950 13.0960 0.1480

34 32 C6 CA M 5.2950 1.0780 11.6150 -0.1575

35 34 H4 HA E 5.3270 0.1900 11.9770 0.1292

36 34 C3 CA M 5.9480 1.3860 10.4310 0.0382

37 36 C7 CT M 6.7320 0.5470 9.4150 0.0040

38 37 H5 HC E 7.0810 -0.2930 9.7220 0.0804

39 37 C11 CA M 5.8390 0.5130 8.1590 0.0156

40 39 C13 CA M 5.0550 -0.4950 7.6150 -0.2329

41 40 H10 HA E 5.0730 -1.3910 7.9590 0.1468

42 40 C15 CA M 4.2360 -0.1370 6.5360 0.0211

43 42 C17 CT M 3.1430 -0.9160 5.7930 0.0072

44 43 H12 HC E 3.2120 -1.8720 5.7930 0.0830

45 43 C20 CA M 1.8430 -0.3100 6.3220 0.0069

46 45 C22 CA M 0.8660 -0.8000 7.1500 -0.1860

47 46 H16 HA E 0.9190 -1.7020 7.4740 0.1330

48 46 C24 CA M -0.2230 0.0290 7.5290 0.0252

49 48 C26 CA M -1.2680 -0.4610 8.3740 -0.1860

50 49 H18 HA E -1.2460 -1.3670 8.6900 0.1330

51 49 C28 CA M -2.2990 0.3620 8.7290 0.0069

52 51 C30 CT M -3.5170 0.1560 9.6200 0.0072

53 52 H20 HC E -3.8030 -0.7500 9.7590 0.0830

54 52 C33 CA M -3.2260 0.9890 10.8730 0.0211

55 54 C35 CA S -2.8960 0.6220 12.1750 -0.2329

56 55 H24 HA E -2.8340 -0.2990 12.4380 0.1468

57 54 C31 CA M -3.2680 2.3330 10.4720 0.0211

58 57 C34 CA M -3.0170 3.3630 11.3670 -0.2329

59 58 H23 HA E -3.0320 4.2820 11.0900 0.1468

60 58 C36 CA M -2.7380 3.0030 12.6890 0.0156

61 60 C37 CA M -2.6600 1.6590 13.0810 0.0156

62 61 C39 CT M -2.2840 1.6570 14.5660 0.0040

63 62 H26 HC E -2.4480 0.8460 15.0520 0.0804

64 62 C41 CT M -3.0790 2.9190 15.0140 -0.2585

65 64 H27 HC E -4.0280 2.8230 14.9010 0.0921

66 64 H28 HC E -2.8760 3.1920 15.9110 0.0921

67 64 C38 CT M -2.4280 3.8380 13.9340 0.0040

68 67 H25 HC E -2.7110 4.7550 13.9200 0.0804

69 67 C40 CA M -0.9580 3.5780 14.2450 0.0382

70 69 C42 CA M -0.8650 2.2310 14.6380 0.0382

71 70 C44 CA M 0.3630 1.6760 14.9670 -0.1575

72 71 H30 HA E 0.4300 0.7580 15.2390 0.1292

73 71 C46 CA M 1.5000 2.4860 14.8910 -0.1778

74 73 H32 HA E 2.3570 2.1200 15.1240 0.1480

75 73 C45 CA M 1.4090 3.8050 14.4850 -0.1778

76 75 H31 HA E 2.2050 4.3410 14.4300 0.1480

77 75 C43 CA M 0.1650 4.3700 14.1530 -0.1575

78 77 H29 HA E 0.1030 5.2840 13.8680 0.1292

GUQJEHg

1 0 N1 N1 M -0.3570 5.9600 10.0580 -0.4300

2 1 C1 CZ M 0.4140 5.2040 9.6690 0.4140

3 2 C2 CM M 1.3850 4.2440 9.1960 -0.2130

4 3 C4 CZ S 2.2660 4.6820 8.1490 0.4140

5 4 N2 N1 E 2.9480 5.0550 7.3010 -0.4300

6 3 C3 CA M 1.4410 2.9750 9.7290 0.1950

7 6 C6 CA B 2.3990 2.0050 9.2540 -0.1250

8 7 C8 CA S 2.4560 0.7740 9.7910 -0.1250

9 8 H4 HA E 3.0970 0.1420 9.4580 0.1510

10 7 H2 HA E 3.0030 2.2420 8.5460 0.1510

11 6 C5 CA M 0.5250 2.5760 10.7720 -0.1250

12 11 H1 HA E -0.1380 3.1970 11.0820 0.1510

13 11 C7 CA M 0.5870 1.3510 11.3130 -0.1250

14 13 H3 HA E -0.0290 1.1110 12.0090 0.1510

15 13 C9 CA M 1.5630 0.3890 10.8650 0.1950

16 15 C10 CM M 1.6560 -0.8510 11.4620 -0.2130

17 16 C12 CZ S 2.6460 -1.8140 11.0500 0.4140

18 17 N4 N1 E 3.4440 -2.5670 10.7190 -0.4300

19 16 C11 CZ M 0.7920 -1.2360 12.5460 0.4140

20 19 N3 N1 M 0.1000 -1.5130 13.4220 -0.4300

GUQJILh

1 0 C1 CA M -8.3310 13.2190 1.9830 0.0290

2 1 C2 CA M -7.2350 13.8380 1.4990 -0.1550

3 2 H1 HA E -6.4170 13.3570 1.3550 0.1290

4 2 C5 CA M -7.3180 15.1770 1.2160 -0.1760

5 4 H3 HA E -6.5520 15.6360 0.8610 0.1470

6 4 C10 CA M -8.4880 15.8720 1.4280 -0.1760

7 6 H8 HA E -8.5260 16.8120 1.2440 0.1470

8 6 C6 CA M -9.6030 15.2220 1.9080 -0.1550

9 8 H4 HA E -10.4250 15.6970 2.0470 0.1290

10 8 C3 CA M -9.5150 13.9330 2.1710 0.0300

11 10 C7 CT M -10.4950 12.9360 2.6800 0.0190

12 11 H5 HC E -11.4240 13.1630 2.5950 0.0740

13 11 C8 CT M -10.0260 11.7140 1.9380 -0.2530

14 13 H6 HC E -10.4480 10.9080 2.2440 0.0890

15 13 H7 HC E -10.1240 11.7980 0.9860 0.0860

16 13 C4 CT M -8.5790 11.8310 2.3900 0.0190

17 16 H2 HC E -7.9680 11.1660 2.0660 0.0740

18 16 C9 CA M -8.8360 11.8590 3.8680 0.0180

19 18 C12 CA S -8.1580 11.3340 4.9240 -0.2380

20 19 H9 HA E -7.3090 10.8980 4.8110 0.1470

21 18 C11 CA M -10.0150 12.5280 4.0650 0.0180

22 21 C13 CA M -10.5770 12.6880 5.2720 -0.2390

23 22 H10 HA E -11.3850 13.1930 5.3940 0.1470

24 22 C15 CA M -9.9380 12.0930 6.3210 0.0150

25 24 C14 CA M -8.7440 11.4540 6.1660 0.0110

26 25 C16 CT M -8.4150 10.8380 7.4720 0.0330

27 26 H11 HC E -7.7660 10.1300 7.4580 0.0770

28 26 C18 CT M -9.8320 10.4490 7.9020 -0.2910

29 28 H13 HC E -9.8760 10.1400 8.8100 0.0940

30 28 H14 HC E -10.2420 9.8120 7.3120 0.0980

31 28 C17 CT M -10.3770 11.8610 7.7210 0.0360

32 31 H12 HC E -11.3090 11.9950 7.9120 0.0770

33 31 C20 CA M -9.3950 12.5680 8.6060 0.0060

34 33 C22 CA S -9.5660 13.5660 9.5250 -0.2220

35 34 H16 HA E -10.3950 14.0460 9.6010 0.1410

36 33 C19 CA M -8.1960 11.9130 8.4760 0.0260

37 36 C21 CA M -7.1330 12.2310 9.2500 -0.2300

38 37 H15 HA E -6.2820 11.8020 9.1370 0.1410

39 37 C23 CA M -7.3220 13.1820 10.1840 0.0060

40 39 C24 CA M -8.5180 13.8350 10.3230 0.0060

41 40 C26 CT M -8.3530 14.8310 11.4620 0.0360

42 41 H18 HC E -9.1530 15.1820 11.8620 0.0760

43 41 C27 CT M -7.4260 13.9940 12.3560 -0.2880

44 43 H19 HC E -7.0650 14.4950 13.0920 0.0940

45 43 H20 HC E -7.8400 13.1870 12.6730 0.0970

46 43 C25 CT M -6.4120 13.7510 11.2590 0.0360

47 46 H17 HC E -5.6440 13.2260 11.4950 0.0770

48 46 C28 CA M -6.1600 15.1560 10.8730 0.0140

49 48 C30 CA S -5.0530 15.7690 10.3750 -0.2270

50 49 H21 HA E -4.2160 15.3040 10.3060 0.1420

51 48 C29 CA M -7.3490 15.8170 10.9770 0.0140

52 51 C31 CA M -7.4880 17.1050 10.5610 -0.2280

53 52 H22 HA E -8.3240 17.5730 10.6260 0.1430

54 52 C33 CA M -6.3800 17.6900 10.0640 0.0130

55 54 C32 CA M -5.1780 17.0600 9.9720 0.0130

56 55 C34 CT M -4.2350 17.9470 9.2740 0.0120

57 56 H23 HC E -3.3000 17.7560 9.3770 0.0780

58 56 C36 CT M -4.7230 19.2870 9.8010 -0.2700

59 58 H25 HC E -4.3290 20.0360 9.3470 0.0920

60 58 H26 HC E -4.6120 19.3780 10.7510 0.0960

61 58 C35 CT M -6.1950 19.0390 9.3840 0.0120

62 61 H24 HC E -6.8340 19.7270 9.5870 0.0780

63 61 C38 CA M -5.9840 18.7060 7.9590 0.0410

64 63 C37 CA M -4.7670 18.0510 7.8620 0.0420

65 64 C39 CA M -4.3080 17.5560 6.7020 -0.1720

66 65 H27 HA E -3.4770 17.0790 6.6460 0.1340

67 65 C41 CA M -5.1010 17.7740 5.5920 -0.1740

68 67 H29 HA E -4.7920 17.4740 4.7330 0.1470

69 67 C42 CA M -6.2710 18.3810 5.6790 -0.1740

70 69 H30 HA E -6.8040 18.4880 4.8890 0.1470

71 69 C40 CA M -6.7320 18.8590 6.8690 -0.1710

72 71 H28 HA E -7.5830 19.3010 6.9190 0.1340

GUQJILg

1 0 N1 N1 M -11.6710 16.1350 4.7170 -0.4300

2 1 C1 CZ M -10.9170 16.1280 5.5330 0.4130

3 2 C2 CM M -9.9360 16.1020 6.5990 -0.2120

4 3 C4 CZ S -10.3640 16.6170 7.8320 0.4130

5 4 N2 N1 E -10.6960 17.0230 8.8200 -0.4300

6 3 C3 CA M -8.6930 15.6240 6.4060 0.1940

7 6 C6 CA B -8.2750 15.1560 5.1660 -0.1250

8 7 H2 HA E -8.8820 15.1800 4.4230 0.1510

9 7 C8 CA S -7.0920 14.6870 4.9970 -0.1250

10 9 H4 HA E -6.8240 14.3920 4.1240 0.1510

11 6 C5 CA M -7.7890 15.5820 7.4690 -0.1250

12 11 H1 HA E -8.0470 15.9410 8.3210 0.1510

13 11 C7 CA M -6.5990 15.0690 7.3230 -0.1250

14 13 H3 HA E -6.0120 15.0060 8.0810 0.1510

15 13 C9 CA M -6.1590 14.5960 6.0600 0.1940

16 15 C10 CM M -4.9320 14.1110 5.8950 -0.2120

17 16 C12 CZ S -3.9840 14.0900 6.9240 0.4130

18 17 N4 N1 E -3.2270 14.0460 7.7520 -0.4300

19 16 C11 CZ M -4.4590 13.6620 4.6320 0.4130

20 19 N3 N1 M -4.0850 13.3290 3.6320 -0.4300

HASWUTh

1 0 C12 CT M 0.0560 -2.5730 0.5520 0.0380

2 1 C13 CT 3 -1.4170 -2.2140 0.3060 0.0380

3 2 N4 N2 S -1.6360 -1.0920 1.2160 -0.4230

4 3 H8 H E -2.2810 -0.6010 1.1480 0.3650

5 2 H12 H1 E -1.5540 -1.8560 -0.6060 0.1040

6 2 H13 H1 E -1.9110 -2.9700 0.4560 0.1040

7 1 H10 H1 E 0.0990 -3.4030 1.1480 0.1040

8 1 H11 H1 E 0.5810 -2.8020 -0.2870 0.1040

9 1 N3 N2 M 0.5440 -1.3870 1.2340 -0.4230

10 9 H7 H E 1.4200 -1.2110 1.4570 0.3650

11 9 C6 CM M -0.4680 -0.6240 1.6290 0.3950

12 11 N1 N2 M -0.3080 0.4750 2.3600 -0.3640

13 12 H4 H E 0.4070 0.6810 2.5190 0.3160

14 12 C1 CT M -1.4040 1.3710 2.7230 0.0320

15 14 H1 H1 E -1.8960 1.5640 1.9010 0.1120

16 14 C2 CT M -2.3760 0.8240 3.7790 -0.0860

17 16 H2 HC E -3.2180 0.5300 3.3830 0.0640

18 16 H3 HC E -1.9300 0.0880 4.2950 0.0640

19 16 C4 CT M -2.6550 1.9980 4.7420 -0.0380

20 19 H5 HC E -3.5180 2.4830 4.4010 0.0670

21 19 H6 HC E -2.6460 1.6880 5.6440 0.0670

22 19 C5 CA M -1.5010 2.9380 4.5190 -0.0390

23 22 C7 CA S -1.0380 3.9860 5.3160 -0.0510

24 23 C10 CA S -1.6810 4.3660 6.6170 0.0200

25 24 C15 CA B -1.0210 4.1800 7.8130 -0.1060

26 25 C18 CA B -1.5950 4.5360 9.0080 -0.1730

27 26 C21 CA B -2.8470 5.0920 9.0270 -0.0770

28 27 C19 CA B -3.5290 5.2710 7.8700 -0.1730

29 28 C16 CA S -2.9460 4.9210 6.6450 -0.1060

30 29 H17 HA E -3.4240 5.0460 5.8040 0.1380

31 28 H22 HA E -4.3430 5.6390 7.8600 0.1650

32 27 H23 HA E -3.2620 5.3470 9.9170 0.1560

33 26 H21 HA E -1.0900 4.3480 9.8850 0.1650

34 25 H16 HA E -0.2660 3.7830 7.8250 0.1380

35 22 C3 CA M -0.8350 2.6100 3.3480 0.3010

36 35 N2 NC M 0.2520 3.2140 2.8850 -0.6290

37 36 C8 CA M 0.7160 4.1710 3.6860 0.3010

38 37 C9 CA M 0.1390 4.6090 4.8760 -0.0390

39 38 C14 CT M 0.9840 5.6960 5.4660 -0.0380

40 39 H14 HC E 1.0600 5.6740 6.4030 0.0670

41 39 H15 HC E 0.6170 6.6370 5.1020 0.0670

42 39 C17 CT M 2.3590 5.4400 4.8210 -0.0860

43 42 H18 HC E 2.8390 4.7020 5.2930 0.0640

44 42 H19 HC E 2.9300 6.2220 4.8440 0.0640

45 42 C11 CT M 2.0200 4.8880 3.4340 0.0320

46 45 H9 H1 E 1.8540 5.6120 2.7390 0.1120

47 45 N5 N2 M 2.9990 3.9590 2.8510 -0.3640

48 47 H20 H E 2.7240 3.1020 2.8190 0.3160

49 47 C20 CM M 4.1970 4.3470 2.4430 0.3950

50 49 N6 N2 M 5.1040 3.4930 1.9770 -0.4230

51 50 H24 H E 4.9650 2.5450 2.1360 0.3650

52 50 C22 CT M 6.3570 4.1880 1.7250 0.0380

53 52 H26 H1 E 7.0670 3.9420 2.3600 0.1040

54 52 H27 H1 E 6.6910 3.9860 0.7330 0.1040

55 52 C23 CT M 5.9560 5.6590 1.7860 0.0380

56 55 H28 H1 E 5.8140 6.0810 0.8290 0.1040

57 55 H29 H1 E 6.4220 6.2130 2.2640 0.1040

58 55 N7 N2 M 4.6430 5.5780 2.4550 -0.4230

59 58 H25 H E 4.1010 6.2570 2.4040 0.3650

HASWUTg

1 0 O1 O M 2.3900 1.1910 3.0160 -0.8720

2 1 P1 P M 3.5300 0.2290 2.8290 1.2900

3 2 O2 O E 3.1330 -1.0410 2.0930 -0.8720

4 2 O3 O E 4.7750 0.8730 2.2740 -0.8720

5 2 O4 OS M 3.9540 -0.3720 4.2910 -0.6510

6 5 C1 CA M 4.5080 0.3640 5.3090 0.5690

7 6 C2 CA M 4.3160 1.7300 5.4850 -0.2110

8 7 H1 HA E 3.8420 2.2100 4.8250 0.1020

9 7 C4 CA M 4.8820 2.3690 6.5530 -0.2050

10 9 H3 HA E 4.6940 3.2970 6.6450 0.1130

11 9 C6 CA M 5.6230 1.6780 7.4900 -0.2950

12 11 H5 HA E 5.9770 2.1920 8.1950 0.1030

13 11 C5 CA M 5.8000 0.3280 7.3120 -0.2050

14 13 H4 HA E 6.3040 -0.1680 7.8130 0.1130

15 13 C3 CA M 5.2660 -0.3380 6.2400 -0.2110

16 15 H2 HA E 5.3810 -1.2550 6.0590 0.1020

HIWNIKh

1 0 C9 CT M -0.4690 2.5420 7.8390 -0.1780

2 1 H5 HC E -0.0060 2.2730 8.6350 0.0420

3 1 H6 HC E -1.1630 1.9080 7.6440 0.0420

4 1 H7 HC E -0.8560 3.4090 7.9720 0.0420

5 1 C5 CT M 0.5180 2.5950 6.6690 0.1840

6 5 H4 HC E 1.1790 3.2840 6.8900 0.0340

7 5 C2 CA M 1.2770 1.2800 6.5400 0.0020

8 7 C4 CA S 0.6120 0.0560 6.5600 -0.3620

9 8 H3 HA E -0.3160 0.0660 6.6110 0.2040

10 7 C1 CA M 2.6650 1.2310 6.4240 0.1640

11 10 O1 OH S 3.4380 2.3670 6.3700 -0.5520

12 11 H1 HO E 3.0890 2.9120 5.8660 0.4120

13 10 C3 CA M 3.3360 0.0180 6.3710 -0.2270

14 13 H2 HA E 4.2640 0.0070 6.3150 0.1660

15 13 C6 CA M 2.6360 -1.1750 6.4000 0.1640

16 15 O2 OH S 3.3800 -2.3310 6.3120 -0.5520

17 16 H8 HO E 2.9490 -2.9040 5.9160 0.4120

18 15 C7 CA M 1.2440 -1.1790 6.5080 0.0020

19 18 C10 CT M 0.4380 -2.4720 6.5430 0.1840

20 19 C13 CT 3 -0.5320 -2.5160 7.7270 -0.1780

21 20 H11 HC E -0.0500 -2.3570 8.5410 0.0420

22 20 H12 HC E -0.9500 -3.3790 7.7670 0.0420

23 20 H13 HC E -1.2050 -1.8390 7.6140 0.0420

24 19 H9 HC E 1.0790 -3.2020 6.6730 0.0340

25 19 C14 CA M -0.2390 -2.7150 5.2070 0.0020

26 25 C18 CA B 0.4770 -3.3020 4.1550 0.1640

27 26 O4 OH S 1.7920 -3.6340 4.3980 -0.5520

28 27 H18 HO E 2.1070 -4.0040 3.7370 0.4120

29 26 C22 CA B -0.1100 -3.5410 2.9300 -0.2270

30 29 C25 CA S -1.4250 -3.1620 2.7160 0.1640

31 30 O6 OH S -1.9630 -3.4800 1.4820 -0.5520

32 31 H26 HO E -2.5940 -2.9800 1.3230 0.4120

33 29 H19 HA E 0.3740 -3.9520 2.2530 0.1660

34 25 C17 CA M -1.5520 -2.3530 4.9480 -0.3620

35 34 H16 HA E -2.0410 -1.9600 5.6350 0.2040

36 34 C21 CA M -2.1820 -2.5460 3.7150 0.0020

37 36 C26 CT M -3.6220 -2.1440 3.4500 0.1840

38 37 C29 CT 3 -4.4930 -2.1460 4.7210 -0.1780

39 38 H27 HC E -5.3890 -1.8870 4.4970 0.0420

40 38 H28 HC E -4.1300 -1.5280 5.3590 0.0420

41 38 H29 HC E -4.5020 -3.0290 5.1010 0.0420

42 37 H24 HC E -3.9970 -2.8230 2.8540 0.0340

43 37 C30 CA M -3.7320 -0.8080 2.7140 0.0020

44 43 C32 CA B -4.0220 -0.7370 1.3560 0.1640

45 44 C31 CA B -4.3070 0.4760 0.7420 -0.2270

46 45 C28 CA S -4.2280 1.6510 1.4660 0.1640

47 46 O7 OH S -4.6700 2.7790 0.7880 -0.5520

48 47 H30 HO E -4.0240 3.2220 0.5460 0.4120

49 45 H31 HA E -4.5500 0.4970 -0.1550 0.1660

50 44 O8 OH S -4.0270 -1.8960 0.5930 -0.5520

51 50 H32 HO E -3.5750 -1.7810 -0.0820 0.4120

52 43 C27 CA M -3.5890 0.4100 3.3740 -0.3620

53 52 H25 HA E -3.3370 0.3900 4.2680 0.2040

54 52 C23 CA M -3.7970 1.6600 2.7840 0.0020

55 54 C19 CT M -3.5690 2.9370 3.5910 0.1840

56 55 C24 CT 3 -4.4490 2.9690 4.8460 -0.1780

57 56 H20 HC E -5.3650 2.8300 4.5960 0.0420

58 56 H21 HC E -4.3610 3.8220 5.2770 0.0420

59 56 H22 HC E -4.1720 2.2760 5.4500 0.0420

60 55 H17 HC E -3.8570 3.6830 3.0240 0.0340

61 55 C15 CA M -2.0880 3.1490 3.8730 0.0020

62 61 C11 CA M -1.4830 2.8160 5.0890 -0.3620

63 62 H10 HA E -2.0130 2.4390 5.7550 0.2040

64 62 C8 CA M -0.1360 3.0160 5.3600 0.0020

65 64 C12 CA M 0.6230 3.6110 4.3460 0.1640

66 65 O3 OH S 1.9600 3.8520 4.6020 -0.5520

67 66 H14 HO E 2.3500 4.0040 3.8980 0.4120

68 65 C16 CA M 0.0620 3.9680 3.1290 -0.2270

69 68 H15 HA E 0.5830 4.3740 2.4760 0.1660

70 68 C20 CA M -1.2770 3.7150 2.8920 0.1640

71 70 O5 OH M -1.8620 4.0060 1.6780 -0.5520

72 71 H23 HO E -1.3320 4.4220 1.2110 0.4120

HIWNIKg

1 0 C5 CA M -0.1100 0.9050 1.6430 -0.2900

2 1 C6 CA B 0.1650 0.1910 2.7720 -0.0290

3 2 C3 CA S 1.4810 0.0260 3.1590 -0.2830

4 3 H2 HA E 1.6860 -0.4660 3.9220 0.1300

5 2 H4 HA E -0.5250 -0.1800 3.2730 0.1460

6 1 H3 HA E -0.9880 1.0200 1.3580 0.1460

7 1 C2 CA M 0.9420 1.4560 0.9300 0.2830

8 7 H1 H4 E 0.7530 1.9420 0.1610 0.0700

9 7 N1 NC M 2.2210 1.3180 1.3010 -0.5480

10 9 C1 CA M 2.4840 0.5930 2.4130 0.3750

11 10 C4 CA M 3.9180 0.4370 2.7700 0.3750

12 11 N2 NC M 4.7030 1.5130 2.5930 -0.5480

13 12 C8 CA M 6.0110 1.3740 2.8760 0.2830

14 13 H6 H4 E 6.5620 2.1170 2.7810 0.0700

15 13 C10 CA M 6.5810 0.1950 3.3000 -0.2900

16 15 H8 HA E 7.4960 0.1360 3.4550 0.1460

17 15 C9 CA M 5.7540 -0.9010 3.4870 -0.0290

18 17 H7 HA E 6.1030 -1.7100 3.7870 0.1460

19 17 C7 CA M 4.4200 -0.7750 3.2260 -0.2830

20 19 H5 HA E 3.8500 -1.4990 3.3530 0.1300

HUNWUIh

1 0 O1 OS M 3.0250 12.9870 6.1580 -0.3050

2 1 C1 CT M 1.8020 12.4260 5.6490 0.0680

3 2 H1 H1 E 1.0520 12.8380 6.1060 0.0420

4 2 H2 H1 E 1.7840 11.4760 5.8480 0.0420

5 2 C3 CT M 1.6400 12.6090 4.2230 0.0680

6 5 H5 H1 E 0.7870 12.2470 3.9370 0.0420

7 5 H6 H1 E 1.6570 13.5550 4.0070 0.0420

8 5 O2 OS M 2.6960 11.9430 3.5590 -0.3050

9 8 C5 CT M 2.6410 12.1220 2.1460 0.0680

10 9 H9 H1 E 2.2900 13.0030 1.9410 0.0420

11 9 H10 H1 E 2.0470 11.4620 1.7540 0.0420

12 9 C7 CT M 3.9940 11.9780 1.5750 0.0680

13 12 H13 H1 E 4.4270 11.1890 1.9360 0.0420

14 12 H14 H1 E 3.9430 11.8870 0.6100 0.0420

15 12 O4 OS M 4.7320 13.1490 1.9220 -0.3050

16 15 C9 CT M 6.1130 13.0540 1.6400 0.0680

17 16 H17 H1 E 6.2570 13.0030 0.6820 0.0420

18 16 H18 H1 E 6.4830 12.2560 2.0480 0.0420

19 16 C10 CT M 6.7710 14.2670 2.1950 0.0680

20 19 H19 H1 E 7.7030 14.2930 1.9280 0.0420

21 19 H20 H1 E 6.3340 15.0650 1.8610 0.0420

22 19 O5 OS M 6.6720 14.2130 3.6100 -0.3050

23 22 C8 CT M 6.8900 15.4300 4.2950 0.0680

24 23 H15 H1 E 7.8370 15.6420 4.3060 0.0420

25 23 H16 H1 E 6.4200 16.1530 3.8470 0.0420

26 23 C6 CT M 6.3840 15.2740 5.6860 0.0680

27 26 H11 H1 E 6.7850 14.4920 6.0980 0.0420

28 26 H12 H1 E 6.6210 16.0530 6.2130 0.0420

29 26 O3 OS M 4.9800 15.1290 5.6440 -0.3050

30 29 C4 CT M 4.3740 14.7870 6.8740 0.0680

31 30 H7 H1 E 4.3600 15.5600 7.4600 0.0420

32 30 H8 H1 E 4.8870 14.0880 7.3090 0.0420

33 30 C2 CT M 2.9740 14.3100 6.6290 0.0680

34 33 H3 H1 E 2.4620 14.3480 7.4520 0.0420

35 33 H4 H1 E 2.5400 14.8780 5.9740 0.0420

HUNWUIg

1 0 N3 NH M 5.5030 11.6680 4.6650 -0.9980

2 1 H1 H E 4.7110 11.8250 4.3750 0.4160

3 1 H2 H E 6.0630 12.3260 4.7040 0.4160

4 1 C1 CR M 5.5470 10.8610 5.7620 0.5560

5 4 N1 NB M 6.3970 10.9530 6.7300 -0.3790

6 5 O1 OS M 6.1440 9.8490 7.5560 -0.0200

7 6 N2 NB M 5.0870 9.0930 7.0520 -0.3790

8 7 C2 CR M 4.7310 9.7080 5.9630 0.5560

9 8 N4 NH M 3.7490 9.2730 5.1310 -0.9980

10 9 H3 H E 3.4270 8.5110 5.3200 0.4160

11 9 H4 H E 3.5850 9.6420 4.3780 0.4160

HUNXAPh

1 0 O1 OS M 7.0500 17.9320 5.8350 -0.2740

2 1 C1 CT M 5.7800 18.5970 5.7560 0.0750

3 2 H19 H1 E 5.3170 18.3390 4.9440 0.0530

4 2 H20 H1 E 5.9040 19.5590 5.7480 0.0530

5 2 C3 CT M 4.9770 18.1890 6.9610 0.1120

6 5 H1 H1 E 5.4460 18.4490 7.7690 0.0330

7 5 H2 H1 E 4.1180 18.6410 6.9500 0.0330

8 5 O2 OS M 4.7780 16.7880 6.9590 -0.3530

9 8 C8 CT M 4.1510 16.3340 8.1570 0.0980

10 9 H6 H1 E 3.2110 16.5690 8.1480 0.0410

11 9 H7 H1 E 4.5640 16.7560 8.9260 0.0410

12 9 C11 CT M 4.3050 14.8380 8.2470 0.0790

13 12 H11 H1 E 3.7760 14.4950 8.9840 0.0330

14 12 H12 H1 E 3.9920 14.4240 7.4280 0.0330

15 12 O4 OS M 5.6880 14.5300 8.4530 -0.2900

16 15 C13 CT M 6.0880 13.2470 7.9460 0.0790

17 16 H15 H1 E 5.6450 13.0730 7.1020 0.0330

18 16 H16 H1 E 5.8320 12.5520 8.5730 0.0330

19 16 C14 CT M 7.5760 13.2360 7.7530 0.0980

20 19 H17 H1 E 8.0160 13.5330 8.5660 0.0410

21 19 H18 H1 E 7.8760 12.3340 7.5580 0.0410

22 19 O5 OS M 7.9210 14.1000 6.6770 -0.3530

23 22 C12 CT M 9.3370 14.2480 6.5670 0.1120

24 23 H13 H1 E 9.7020 14.5420 7.4160 0.0330

25 23 H14 H1 E 9.7390 13.3950 6.3430 0.0330

26 23 C9 CT M 9.6600 15.2590 5.4970 0.0750

27 26 H8 H1 E 10.6140 15.2680 5.3200 0.0530

28 26 H9 H1 E 9.1970 15.0380 4.6740 0.0530

29 26 O3 OS M 9.2320 16.5460 5.9780 -0.2740

30 29 C4 CA M 9.2100 17.5590 5.0460 0.1760

31 30 C2 CA M 8.0310 18.3130 4.9580 0.1760

32 31 C5 CA M 7.9440 19.3720 4.0610 -0.1570

33 32 H3 HA E 7.1610 19.8660 3.9910 0.1250

34 32 C7 CA M 9.0480 19.6820 3.2670 -0.2210

35 34 H5 HA E 8.9910 20.3810 2.6540 0.1660

36 34 C10 CA M 10.2270 18.9640 3.3790 -0.2210

37 36 H10 HA E 10.9660 19.1980 2.8640 0.1660

38 36 C6 CA M 10.3010 17.8940 4.2620 -0.1570

39 38 H4 HA E 11.0870 17.4010 4.3270 0.1250

HUNXAPg

1 0 N3 NH M 7.8670 16.4420 8.5960 -0.9980

2 1 H1 H E 8.2350 16.8220 7.9120 0.4160

3 1 H2 H E 7.1450 15.9900 8.4710 0.4160

4 1 C1 CR M 8.0960 17.1030 9.7450 0.5560

5 4 N1 NB M 9.0280 17.9880 9.9250 -0.3790

6 5 O1 OS M 8.9390 18.3650 11.2810 -0.0210

7 6 N2 NB M 7.9250 17.6280 11.9220 -0.3790

8 7 C2 CR M 7.4150 16.8630 11.0080 0.5560

9 8 N4 NH M 6.3620 16.0220 11.1860 -0.9980

10 9 H3 H E 6.3270 15.3620 10.6200 0.4160

11 9 H4 H E 6.0710 15.6760 11.9180 0.4160

HUNXIXh

1 0 O1 OS M 2.9180 1.8810 4.6300 -0.2410

2 1 C1 CT M 2.9250 0.7320 5.4880 -0.0020

3 2 H1 H1 E 3.8350 0.5800 5.8480 0.0630

4 2 H2 H1 E 2.3070 0.8710 6.2490 0.0630

5 2 C2 CT M 2.4870 -0.4470 4.6770 0.2090

6 5 H3 H1 E 2.9800 -0.4660 3.8180 0.0150

7 5 H4 H1 E 2.6820 -1.2840 5.1680 0.0150

8 5 O2 OS M 1.0910 -0.3420 4.4330 -0.4210

9 8 C4 CT M 0.5990 -1.3910 3.6100 0.0940

10 9 H5 H1 E 0.5640 -2.2360 4.1240 0.0630

11 9 H6 H1 E 1.2010 -1.5230 2.8350 0.0630

12 9 C7 CT M -0.7660 -1.0290 3.1360 0.0340

13 12 H8 H1 E -1.1810 -1.8010 2.6740 0.0690

14 12 H9 H1 E -1.3370 -0.7810 3.9060 0.0690

15 12 O4 OS M -0.6530 0.0770 2.2350 -0.3570

16 15 C12 CT M -1.8870 0.3980 1.6070 0.0310

17 16 H15 H1 E -2.5050 0.7970 2.2700 0.0680

18 16 H16 H1 E -2.3060 -0.4260 1.2510 0.0680

19 16 C14 CT M -1.6470 1.3640 0.4940 0.0310

20 19 H19 H1 E -0.9040 1.0400 -0.0750 0.0680

21 19 H20 H1 E -2.4590 1.4330 -0.0680 0.0680

22 19 O6 OS M -1.3190 2.6520 1.0270 -0.3570

23 22 C16 CT M -1.0380 3.6240 0.0100 0.0340

24 23 H23 H1 E -1.4940 3.3610 -0.8290 0.0690

25 23 H24 H1 E -1.3970 4.5030 0.2900 0.0690

26 23 C15 CT M 0.4310 3.7470 -0.2420 0.0940

27 26 H21 H1 E 0.8220 2.8560 -0.4290 0.0630

28 26 H22 H1 E 0.5970 4.3320 -1.0230 0.0630

29 26 O5 OS M 1.0180 4.3050 0.9220 -0.4210

30 29 C13 CT M 2.4190 4.4920 0.7910 0.2090

31 30 H17 H1 E 2.6090 5.1500 0.0750 0.0150

32 30 H18 H1 E 2.8560 3.6370 0.5530 0.0150

33 30 C11 CT M 2.9460 4.9910 2.0970 -0.0020

34 33 H13 H1 E 3.8700 5.3290 1.9840 0.0630

35 33 H14 H1 E 2.3800 5.7300 2.4320 0.0630

36 33 O3 OS M 2.9300 3.8950 3.0270 -0.2410

37 36 C5 CA M 3.2400 4.1810 4.3350 0.1600

38 37 C3 CA M 3.2130 3.0900 5.2070 0.1600

39 38 C6 CA M 3.4650 3.2800 6.5580 -0.1870

40 39 H7 HA E 3.4440 2.5430 7.1560 0.1500

41 39 C9 CA M 3.7500 4.5650 7.0330 -0.1830

42 41 H11 HA E 3.9110 4.7020 7.9590 0.1550

43 41 C10 CA M 3.7960 5.6250 6.1700 -0.1830

44 43 H12 HA E 4.0070 6.4910 6.4990 0.1550

45 43 C8 CA M 3.5390 5.4490 4.8110 -0.1870

46 45 H10 HA E 3.5670 6.1910 4.2170 0.1500

HUNXIXg

1 0 C1 CA M -0.2490 2.4190 4.5120 0.4290

2 1 H1 H5 E -0.2940 1.4910 4.3110 0.1510

3 1 N1 NA M -0.3140 3.2850 3.5080 -0.2360

4 3 H2 H E -0.4300 2.9880 2.6880 0.3460

5 3 C2 CM M -0.2060 4.6010 3.7250 0.0980

6 5 H3 H4 E -0.2290 5.2180 3.0020 0.2250

7 5 C4 CM M -0.0630 5.0370 5.0170 -0.2660

8 7 H5 HA E 0.0210 5.9610 5.2170 0.2020

9 7 C3 CA M -0.0420 4.0900 6.0100 0.4270

10 9 H4 H4 E 0.0350 4.3820 6.9100 0.1370

11 9 N2 NC M -0.1250 2.7790 5.7700 -0.5140

HUNXODh

1 0 O1 OS M 0.0330 1.1340 -2.3870 -0.3030

2 1 C1 CT M -0.5730 -0.0310 -2.9800 0.1360

3 2 H1 H1 E -1.0620 0.2240 -3.8020 0.0410

4 2 H2 H1 E -1.2150 -0.4370 -2.3470 0.0410

5 2 C2 CT M 0.5030 -1.0110 -3.3120 0.0260

6 5 H3 H1 E 0.1180 -1.7890 -3.7890 0.0560

7 5 H4 H1 E 1.1790 -0.5870 -3.8990 0.0560

8 5 O2 OS M 1.1100 -1.4370 -2.1000 -0.3020

9 8 C4 CT M 1.7460 -2.6960 -2.1710 0.0260

10 9 H5 H1 E 2.6200 -2.6100 -2.6290 0.0560

11 9 H6 H1 E 1.1870 -3.3300 -2.6870 0.0560

12 9 C7 CT M 1.9460 -3.2050 -0.7800 0.1360

13 12 H8 H1 E 1.0730 -3.2840 -0.3190 0.0410

14 12 H9 H1 E 2.3700 -4.1000 -0.8020 0.0410

15 12 O4 OS M 2.7890 -2.2750 -0.0800 -0.3030

16 15 C12 CA M 2.9490 -2.4810 1.2690 0.2010

17 16 C14 CA M 2.4820 -3.5730 1.9680 -0.1690

18 17 H17 HA E 1.9950 -4.2510 1.5160 0.1220

19 17 C16 CA M 2.7210 -3.6850 3.3400 -0.2240

20 19 H18 HA E 2.3900 -4.4370 3.8180 0.1680

21 19 C19 CA M 3.4290 -2.7160 3.9970 -0.2240

22 21 H22 HA E 3.6090 -2.8100 4.9250 0.1680

23 21 C17 CA M 3.8890 -1.5980 3.3150 -0.1690

24 23 H19 HA E 4.3710 -0.9220 3.7770 0.1220

25 23 C15 CA M 3.6390 -1.4700 1.9530 0.2010

26 25 O6 OS M 4.0310 -0.3920 1.1890 -0.3030

27 26 C20 CT M 4.5260 0.7340 1.9260 0.1360

28 27 H23 H1 E 5.4050 0.5220 2.3260 0.0410

29 27 H24 H1 E 3.8970 0.9710 2.6530 0.0410

30 27 C18 CT M 4.6560 1.8660 0.9670 0.0260

31 30 H20 H1 E 5.0840 2.6390 1.4130 0.0560

32 30 H21 H1 E 5.2180 1.5960 0.2000 0.0560

33 30 O5 OS M 3.3490 2.2280 0.5110 -0.3020

34 33 C13 CT M 3.2660 3.5490 -0.0380 0.0260

35 34 H15 H1 E 3.6610 3.5670 -0.9460 0.0560

36 34 H16 H1 E 3.7610 4.1890 0.5320 0.0560

37 34 C11 CT M 1.8240 3.9220 -0.0970 0.1360

38 37 H13 H1 E 1.4260 3.8950 0.8090 0.0410

39 37 H14 H1 E 1.7220 4.8380 -0.4590 0.0410

40 37 O3 OS M 1.1710 2.9710 -0.9560 -0.3030

41 40 C5 CA M -0.1940 3.0230 -0.9920 0.2010

42 41 C3 CA M -0.8200 2.0120 -1.7640 0.2010

43 42 C6 CA M -2.1930 1.9710 -1.8490 -0.1690

44 43 H7 HA E -2.6190 1.2880 -2.3550 0.1220

45 43 C9 CA M -2.9560 2.9310 -1.1930 -0.2240

46 45 H11 HA E -3.9030 2.9070 -1.2590 0.1680

47 45 C10 CA M -2.3450 3.9210 -0.4450 -0.2240

48 47 H12 HA E -2.8730 4.5710 0.0010 0.1680

49 47 C8 CA M -0.9660 3.9630 -0.3450 -0.1690

50 49 H10 HA E -0.5500 4.6430 0.1730 0.1220

HUNXODg

1 0 C1 CA M 3.0320 1.6000 -3.0850 0.4410

2 1 H1 H5 E 2.4810 2.3680 -2.9900 0.1490

3 1 N1 NC M 3.4380 1.2780 -4.2700 -0.5230

4 3 C2 CA M 4.2230 0.2110 -4.3670 0.4380

5 4 H3 H4 E 4.5470 -0.0400 -5.2250 0.1330

6 4 C4 CM M 4.5930 -0.5460 -3.2760 -0.2530

7 6 H5 HA E 5.1620 -1.3000 -3.3720 0.1960

8 6 C3 CM M 4.1180 -0.1780 -2.0580 0.0890

9 8 H4 H4 E 4.3260 -0.6800 -1.2790 0.2280

10 8 N2 NA M 3.3490 0.9080 -1.9890 -0.2500

11 10 H2 H E 3.0440 1.1740 -1.2070 0.3510

HUNXUJh

1 0 O1 OS M -0.5840 -6.1270 -7.6870 -0.3030

2 1 C1 CT M -1.1000 -7.2670 -8.3890 0.1360

3 2 H1 H1 E -1.9960 -7.0630 -8.7550 0.0410

4 2 H2 H1 E -0.5000 -7.5020 -9.1430 0.0410

5 2 C2 CT M -1.1850 -8.4150 -7.4240 0.0260

6 5 H3 H1 E -1.6710 -9.1700 -7.8390 0.0560

7 5 H4 H1 E -1.6750 -8.1370 -6.6100 0.0560

8 5 O2 OS M 0.1420 -8.8190 -7.0810 -0.3020

9 8 C4 CT M 0.1920 -10.0900 -6.4360 0.0260

10 9 H5 H1 E -0.2020 -10.0310 -5.5300 0.0560

11 9 H6 H1 E -0.3200 -10.7590 -6.9560 0.0560

12 9 C7 CT M 1.6350 -10.4940 -6.3500 0.1360

13 12 H8 H1 E 2.0470 -10.4870 -7.2490 0.0410

14 12 H9 H1 E 1.7130 -11.4060 -5.9730 0.0410

15 12 O4 OS M 2.2940 -9.5380 -5.4910 -0.3030

16 15 C12 CA M 3.6650 -9.5900 -5.4450 0.2010

17 16 C14 CA M 4.4370 -10.5570 -6.0660 -0.1690

18 17 H17 HA E 4.0200 -11.2450 -6.5720 0.1220

19 17 C16 CA M 5.8200 -10.5210 -5.9490 -0.2240

20 19 H18 HA E 6.3460 -11.1930 -6.3630 0.1680

21 19 C19 CA M 6.4310 -9.5140 -5.2320 -0.2240

22 21 H22 HA E 7.3790 -9.4890 -5.1670 0.1680

23 21 C17 CA M 5.6690 -8.5360 -4.6070 -0.1690

24 23 H19 HA E 6.0940 -7.8450 -4.1120 0.1220

25 23 C15 CA M 4.2930 -8.5670 -4.7060 0.2010

26 25 O6 OS M 3.4300 -7.6650 -4.1130 -0.3030

27 26 C20 CT M 4.0410 -6.5130 -3.5150 0.1360

28 27 H23 H1 E 4.4950 -6.7700 -2.6740 0.0410

29 27 H24 H1 E 4.7160 -6.1330 -4.1290 0.0410

30 27 C18 CT M 2.9740 -5.4880 -3.2290 0.0260

31 30 H20 H1 E 3.3590 -4.7290 -2.7230 0.0560

32 30 H21 H1 E 2.2540 -5.8920 -2.6830 0.0560

33 30 O5 OS M 2.4480 -5.0310 -4.4670 -0.3020

34 33 C13 CT M 1.7300 -3.8020 -4.3640 0.0260

35 34 H15 H1 E 0.8660 -3.9510 -3.9010 0.0560

36 34 H16 H1 E 2.2530 -3.1460 -3.8400 0.0560

37 34 C11 CT M 1.4880 -3.2810 -5.7470 0.1360

38 37 H13 H1 E 2.3480 -3.1730 -6.2240 0.0410

39 37 H14 H1 E 1.0410 -2.4000 -5.7060 0.0410

40 37 O3 OS M 0.6570 -4.2210 -6.4430 -0.3030

41 40 C5 CA M 0.5110 -4.0530 -7.8020 0.2010

42 41 C3 CA M -0.1750 -5.0770 -8.4740 0.2010

43 42 C6 CA M -0.4090 -4.9730 -9.8290 -0.1690

44 43 H7 HA E -0.8970 -5.6500 -10.2810 0.1220

45 43 C9 CA M 0.0720 -3.8710 -10.5340 -0.2240

46 45 H11 HA E -0.0930 -3.8020 -11.4670 0.1680

47 45 C10 CA M 0.7770 -2.8940 -9.9000 -0.2240

48 47 H12 HA E 1.1210 -2.1600 -10.3950 0.1680

49 47 C8 CA M 0.9990 -2.9670 -8.5130 -0.1690

50 49 H10 HA E 1.4780 -2.2790 -8.0680 0.1220

HUNXUJg

1 0 N1 NC M -1.0710 -6.6580 -2.3260 -0.3710

2 1 C1 CA M -1.1420 -6.0260 -3.5110 0.2540

3 2 H1 H4 E -1.7410 -5.2950 -3.6030 0.1430

4 2 C3 CA M -0.3900 -6.3960 -4.5870 -0.0810

5 4 H3 H4 E -0.4420 -5.9160 -5.4060 0.2110

6 4 N2 NA M 0.4100 -7.4300 -4.4660 -0.0470

7 6 H5 H E 0.9020 -7.6720 -5.1540 0.3650

8 6 C4 CA M 0.4990 -8.1220 -3.3330 -0.0810

9 8 H4 H4 E 1.0670 -8.8830 -3.2720 0.2110

10 8 C2 CA M -0.2320 -7.7190 -2.2720 0.2540

11 10 H2 H4 E -0.1560 -8.1990 -1.4550 0.1430

HUNYAQh

1 0 O1 OS M 4.6070 8.1220 1.2940 -0.3030

2 1 C1 CT M 3.9310 7.0670 0.5830 0.1360

3 2 H1 H1 E 3.4850 7.4310 -0.2230 0.0410

4 2 H2 H1 E 3.2440 6.6510 1.1600 0.0410

5 2 C2 CT M 4.9530 6.0650 0.1960 0.0260

6 5 H3 H1 E 4.5340 5.3410 -0.3330 0.0560

7 5 H4 H1 E 5.6540 6.4920 -0.3560 0.0560

8 5 O2 OS M 5.5300 5.5270 1.3970 -0.3020

9 8 C4 CT M 6.2560 4.3000 1.2190 0.0260

10 9 H5 H1 E 7.1080 4.4780 0.7490 0.0560

11 9 H6 H1 E 5.7250 3.6680 0.6740 0.0560

12 9 C7 CT M 6.5350 3.6970 2.5880 0.1360

13 12 H8 H1 E 5.6870 3.5390 3.0710 0.0410

14 12 H9 H1 E 7.0060 2.8310 2.4900 0.0410

15 12 O4 OS M 7.3520 4.6250 3.3110 -0.3030

16 15 C12 CA M 7.4940 4.3790 4.6630 0.2010

17 16 C14 CA M 7.0090 3.2650 5.3110 -0.1690

18 17 H17 HA E 6.5530 2.5940 4.8160 0.1220

19 17 C16 CA M 7.1810 3.1140 6.6890 -0.2240

20 19 H18 HA E 6.8440 2.3470 7.1380 0.1680

21 19 C19 CA M 7.8480 4.1020 7.3890 -0.2240

22 21 H22 HA E 7.9630 4.0110 8.3270 0.1680

23 21 C17 CA M 8.3520 5.2180 6.7540 -0.1690

24 23 H19 HA E 8.8220 5.8780 7.2500 0.1220

25 23 C15 CA M 8.1660 5.3690 5.3760 0.2010

26 25 O6 OS M 8.5970 6.4460 4.6540 -0.3030

27 26 C20 CT M 9.1750 7.5400 5.4200 0.1360

28 27 H23 H1 E 10.0490 7.2680 5.7990 0.0410

29 27 H24 H1 E 8.5750 7.7910 6.1660 0.0410

30 27 C18 CT M 9.3520 8.6920 4.4910 0.0260

31 30 H20 H1 E 9.8640 9.4130 4.9370 0.0560

32 30 H21 H1 E 9.8540 8.4070 3.6870 0.0560

33 30 O5 OS M 8.0540 9.1690 4.1160 -0.3020

34 33 C13 CT M 8.0490 10.4150 3.4080 0.0260

35 34 H15 H1 E 8.3950 10.2890 2.4890 0.0560

36 34 H16 H1 E 8.6250 11.0740 3.8720 0.0560

37 34 C11 CT M 6.6510 10.8990 3.3680 0.1360

38 37 H13 H1 E 6.2760 10.9420 4.2830 0.0410

39 37 H14 H1 E 6.6140 11.8040 2.9690 0.0410

40 37 O3 OS M 5.9020 9.9650 2.5620 -0.3030

41 40 C5 CA M 4.5280 10.0890 2.5870 0.2010

42 41 C3 CA M 3.8290 9.0960 1.8970 0.2010

43 42 C6 CA M 2.4370 9.1150 1.8650 -0.1690

44 43 H7 HA E 1.9590 8.4290 1.4160 0.1220

45 43 C9 CA M 1.7560 10.1390 2.4920 -0.2240

46 45 H11 HA E 0.8080 10.1660 2.4580 0.1680

47 45 C10 CA M 2.4520 11.1330 3.1710 -0.2240

48 47 H12 HA E 1.9780 11.8320 3.6060 0.1680

49 47 C8 CA M 3.8380 11.1040 3.2150 -0.1690

50 49 H10 HA E 4.3110 11.7850 3.6790 0.1220

HUNYAQg

1 0 C1 CA M 3.7210 5.8720 5.6960 0.2660

2 1 H1 H4 E 3.0560 5.3590 6.1400 0.1500

3 1 C2 CA M 4.3690 6.8640 6.4170 -0.0750

4 3 H2 HA E 4.1600 7.0220 7.3310 0.1920

5 3 C3 CA M 5.3000 7.5960 5.7790 -0.0490

6 5 H3 HA E 5.7680 8.2930 6.2260 0.1930

7 5 C4 CA M 5.5540 7.3030 4.4540 0.0130

8 7 H5 H4 E 6.2060 7.7980 3.9710 0.2050

9 7 N2 NA M 4.8950 6.3420 3.8730 0.1210

10 9 H4 H E 5.0820 6.1730 3.0300 0.3350

11 9 N1 NC M 3.9710 5.6050 4.4490 -0.3520

IKARUHh

1 0 C9 CT M 5.4480 3.9050 0.2420 -0.1780

2 1 H5 HC E 5.2270 4.7390 -0.2270 0.0420

3 1 H6 HC E 4.7530 3.2440 0.0480 0.0420

4 1 H7 HC E 6.3500 3.5930 -0.1310 0.0420

5 1 C5 CT M 5.5480 4.1710 1.7500 0.1840

6 5 H4 HC E 6.1970 4.8840 1.9250 0.0340

7 5 C2 CA M 4.2230 4.6880 2.2980 0.0020

8 7 C4 CA S 3.0700 3.8960 2.2730 -0.3620

9 8 H3 HA E 3.1680 2.9810 1.9310 0.2040

10 7 C1 CA M 4.1010 5.9580 2.8730 0.1640

11 10 O1 OH S 5.2530 6.7070 3.0940 -0.5520

12 11 H1 HO E 5.1300 7.1910 3.8400 0.4120

13 10 C3 CA M 2.8640 6.4490 3.2710 -0.2270

14 13 H2 HA E 2.7840 7.3270 3.6400 0.1660

15 13 C6 CA M 1.7320 5.6460 3.1780 0.1640

16 15 O2 OH S 0.5060 6.1010 3.6000 -0.5520

17 16 H8 HO E 0.5270 7.0100 3.5740 0.4120

18 15 C7 CA M 1.8230 4.3250 2.7230 0.0020

19 18 C10 CT M 0.6100 3.4040 2.7320 0.1840

20 19 C13 CT 3 0.1070 3.1230 1.3140 -0.1780

21 20 H11 HC E -0.1970 3.9570 0.8700 0.0420

22 20 H12 HC E -0.6300 2.5130 1.3330 0.0420

23 20 H13 HC E 0.7940 2.7150 0.7500 0.0420

24 19 H9 HC E -0.1030 3.8440 3.1800 0.0340

25 19 C14 CA M 0.8940 2.1460 3.5440 0.0020

26 25 C18 CA B 0.8980 2.2430 4.9400 0.1640

27 26 O4 OH S 0.6970 3.4790 5.4980 -0.5520

28 27 H18 HO E 0.8130 3.4130 6.3510 0.4120

29 26 C22 CA B 1.0890 1.1160 5.7330 -0.2270

30 29 C25 CA S 1.3000 -0.1230 5.1390 0.1640

31 30 O6 OH S 1.4430 -1.2480 5.9110 -0.5520

32 31 H26 HO E 1.3000 -1.0390 6.7900 0.4120

33 29 H19 HA E 1.0490 1.1830 6.7090 0.1660

34 25 C17 CA M 1.1300 0.8910 2.9850 -0.3620

35 34 H16 HA E 1.1230 0.7860 2.0350 0.2040

36 34 C21 CA M 1.3460 -0.2630 3.7470 0.0020

37 36 C26 CT M 1.5450 -1.6450 3.1540 0.1840

38 37 C29 CT 3 0.9550 -1.8130 1.7490 -0.1780

39 38 H27 HC E 0.9480 -2.7500 1.4730 0.0420

40 38 H28 HC E 1.4840 -1.3090 1.0760 0.0420

41 38 H29 HC E 0.0150 -1.5390 1.7690 0.0420

42 37 H24 HC E 1.0610 -2.2570 3.7300 0.0340

43 37 C30 CA M 3.0030 -2.1030 3.1410 0.0020

44 43 C32 CA B 3.3070 -3.4430 3.4150 0.1640

45 44 C31 CA B 4.6110 -3.9190 3.3430 -0.2270

46 45 C28 CA S 5.6430 -3.0580 2.9800 0.1640

47 46 O7 OH S 6.9360 -3.5180 2.8790 -0.5520

48 47 H30 HO E 6.9280 -4.3740 2.6900 0.4120

49 45 H31 HA E 4.8240 -4.9120 3.5380 0.1660

50 44 O8 OH S 2.2850 -4.3030 3.7610 -0.5520

51 50 H32 HO E 2.5950 -4.9300 4.3210 0.4120

52 43 C27 CA M 4.0740 -1.2660 2.8040 -0.3620

53 52 H25 HA E 3.8530 -0.3360 2.6240 0.2040

54 52 C23 CA M 5.3990 -1.7070 2.7200 0.0020

55 54 C19 CT M 6.5660 -0.8310 2.2770 0.1840

56 55 C24 CT 3 6.7150 -0.9050 0.7550 -0.1780

57 56 H20 HC E 6.8610 -1.8460 0.4690 0.0420

58 56 H21 HC E 7.5000 -0.3530 0.4360 0.0420

59 56 H22 HC E 5.9210 -0.5730 0.2630 0.0420

60 55 H17 HC E 7.3890 -1.2620 2.6690 0.0340

61 55 C15 CA M 6.5150 0.5850 2.8130 0.0020

62 61 C11 CA M 6.0510 1.6700 2.0690 -0.3620

63 62 H10 HA E 5.7390 1.5040 1.1510 0.2040

64 62 C8 CA M 6.0430 2.9780 2.5500 0.0020

65 64 C12 CA M 6.5290 3.1820 3.8450 0.1640

66 65 O3 OH S 6.5610 4.4290 4.4360 -0.5520

67 66 H14 HO E 6.2530 5.0440 3.9180 0.4120

68 65 C16 CA M 7.0020 2.1360 4.6170 -0.2270

69 68 H15 HA E 7.3150 2.2690 5.4660 0.1660

70 68 C20 CA M 6.9990 0.8460 4.0990 0.1640

71 70 O5 OH M 7.4540 -0.2130 4.8400 -0.5520

72 71 H23 HO E 7.6820 0.1140 5.5560 0.4120

IKARUHg

1 0 C3 CA M 4.4190 2.7670 8.5120 -0.0610

2 1 H4 HA E 4.5970 2.3490 9.3450 0.1700

3 1 C1 CA M 4.3680 4.1410 8.4890 -0.0270

4 3 H2 H4 E 4.5110 4.6820 9.2880 0.2380

5 3 N1 NA M 4.1380 4.7810 7.3300 -0.0790

6 5 H1 H E 4.1050 5.7060 7.3100 0.3640

7 5 C2 CA M 3.9400 4.1270 6.1820 -0.0270

8 7 H3 H4 E 3.7650 4.6820 5.4090 0.2380

9 7 C4 CA M 3.9810 2.7490 6.1520 -0.0610

10 9 H5 HA E 3.8060 2.2890 5.2720 0.1700

11 9 C5 CA M 4.2240 2.0460 7.3340 0.0760

12 11 C6 CA M 4.2560 0.5600 7.3300 0.0760

13 12 C7 CA M 3.9530 -0.1680 8.4770 -0.0610

14 13 H6 HA E 3.7060 0.2620 9.3060 0.1700

15 13 C9 CA M 3.9720 -1.5500 8.4420 -0.0270

16 15 H8 H4 E 3.7820 -2.0960 9.1930 0.2380

17 15 N2 NA M 4.3110 -2.1820 7.3080 -0.0790

18 17 H10 H E 4.3530 -3.0670 7.2890 0.3640

19 17 C10 CA M 4.6010 -1.5180 6.1830 -0.0270

20 19 H9 H4 E 4.8270 -2.0190 5.4090 0.2380

21 19 C8 CA M 4.5770 -0.1440 6.1610 -0.0610

22 21 H7 HA E 4.8060 0.3410 5.3140 0.1700

IKUTOXh

1 0 C12 CT M 0.8100 0.9650 9.3350 -0.1780

2 1 H13 HC E 0.0490 0.5760 8.8980 0.0440

3 1 H14 HC E 1.2910 0.2820 9.8070 0.0440

4 1 H15 HC E 0.5150 1.6370 9.9540 0.0440

5 1 C11 CT M 1.7810 1.5670 8.3100 0.2000

6 5 C13 CT 3 2.9370 2.2410 9.0920 -0.1780

7 6 H16 HC E 3.4800 2.7580 8.4930 0.0440

8 6 H17 HC E 2.5170 2.8260 9.7280 0.0440

9 6 H18 HC E 3.4870 1.6070 9.5570 0.0440

10 5 C9 CA M 2.3490 0.4900 7.3780 -0.0040

11 10 C7 CA B 3.4970 0.6940 6.6500 -0.1590

12 11 C4 CA S 3.9950 -0.2250 5.7440 -0.2250

13 12 H4 HA E 4.8070 -0.0530 5.2630 0.1360

14 11 H8 HA E 3.9680 1.5200 6.7810 0.1490

15 10 C6 CA M 1.6940 -0.7100 7.1520 -0.1590

16 15 H7 HA E 0.9040 -0.9130 7.6590 0.1490

17 15 C3 CA M 2.1960 -1.6390 6.2290 -0.2250

18 17 H3 HA E 1.7110 -2.4520 6.0680 0.1360

19 17 C1 CA M 3.3270 -1.3890 5.5160 0.2660

20 19 O1 OS M 3.9460 -2.2080 4.5150 -0.3210

21 20 C2 CT M 4.8700 -1.8330 3.5320 0.1210

22 21 H1 H1 E 5.7430 -1.7130 3.9120 0.0240

23 21 H2 H1 E 4.5790 -0.9920 3.1700 0.0240

24 21 C5 CT M 4.9020 -2.8980 2.4490 0.0200

25 24 H5 HC E 5.2550 -3.7190 2.8260 0.0120

26 24 H6 HC E 5.5050 -2.6130 1.7460 0.0120

27 24 C8 CT M 3.5510 -3.1760 1.8520 0.0200

28 27 H9 HC E 3.0330 -3.7220 2.4640 0.0120

29 27 H10 HC E 3.0770 -2.3410 1.7200 0.0120

30 27 C10 CT M 3.6890 -3.9000 0.5200 0.1210

31 30 H11 H1 E 4.2130 -4.7080 0.6270 0.0240

32 30 H12 H1 E 4.1270 -3.3280 -0.1300 0.0240

33 30 O2 OS M 2.3770 -4.2140 0.0970 -0.3210

34 33 C15 CA M 2.2080 -4.5750 -1.2350 0.2660

35 34 C19 CA B 3.2580 -4.7430 -2.1300 -0.2250

36 35 H22 HA E 4.1390 -4.6540 -1.8450 0.1360

37 35 C23 CA S 2.9680 -5.0550 -3.4840 -0.1590

38 37 H26 HA E 3.6670 -5.1390 -4.0920 0.1490

39 34 C18 CA M 0.9150 -4.7370 -1.6590 -0.2250

40 39 H21 HA E 0.2080 -4.6020 -1.0710 0.1360

41 39 C22 CA M 0.6670 -5.1050 -2.9860 -0.1590

42 41 H25 HA E -0.2080 -5.2680 -3.2560 0.1490

43 41 C25 CA M 1.6940 -5.2330 -3.9070 -0.0040

44 43 C27 CT M 1.3430 -5.6600 -5.3340 0.2000

45 44 C32 CT 3 2.6240 -5.9330 -6.1300 -0.1780

46 45 H33 HC E 3.0870 -6.6310 -5.6600 0.0440

47 45 H34 HC E 3.1970 -5.1670 -6.2090 0.0440

48 45 H35 HC E 2.3820 -6.2440 -7.0070 0.0440

49 44 C33 CT 3 0.5310 -6.9570 -5.2960 -0.1780

50 49 H36 HC E 1.0440 -7.6630 -4.8930 0.0440

51 49 H37 HC E 0.3190 -7.1930 -6.2020 0.0440

52 49 H38 HC E -0.2800 -6.8300 -4.8000 0.0440

53 44 C31 CA M 0.5150 -4.5770 -6.0570 0.0130

54 53 C37 CA B 0.1500 -4.7640 -7.3960 -0.1750

55 54 H42 HA E 0.4340 -5.5610 -7.8480 0.1380

56 54 C41 CA S -0.5870 -3.8100 -8.0780 -0.1750

57 56 H46 HA E -0.8070 -3.9770 -8.9970 0.1380

58 53 C36 CA M 0.0550 -3.4270 -5.4760 -0.1750

59 58 H41 HA E 0.3000 -3.2590 -4.5630 0.1380

60 58 C40 CA M -0.6980 -2.4850 -6.1600 -0.1750

61 60 H45 HA E -1.0150 -1.7110 -5.6890 0.1380

62 60 C43 CA M -1.0330 -2.6380 -7.4900 0.0130

63 62 C44 CT M -1.7810 -1.5670 -8.3100 0.2000

64 63 C47 CT 3 -0.8100 -0.9650 -9.3350 -0.1780

65 64 H49 HC E -0.0490 -0.5760 -8.8980 0.0440

66 64 H50 HC E -1.2910 -0.2820 -9.8070 0.0440

67 64 H51 HC E -0.5150 -1.6370 -9.9540 0.0440

68 63 C48 CT 3 -2.9370 -2.2410 -9.0920 -0.1780

69 68 H52 HC E -3.4800 -2.7580 -8.4930 0.0440

70 68 H53 HC E -2.5170 -2.8260 -9.7280 0.0440

71 68 H54 HC E -3.4870 -1.6070 -9.5570 0.0440

72 63 C46 CA M -2.3490 -0.4900 -7.3780 -0.0040

73 72 C51 CA B -3.4970 -0.6940 -6.6500 -0.1590

74 73 H58 HA E -3.9680 -1.5200 -6.7810 0.1490

75 73 C54 CA S -3.9950 0.2250 -5.7440 -0.2250

76 75 H62 HA E -4.8070 0.0530 -5.2630 0.1360

77 72 C50 CA M -1.6940 0.7100 -7.1520 -0.1590

78 77 H57 HA E -0.9040 0.9130 -7.6590 0.1490

79 77 C53 CA M -2.1960 1.6390 -6.2290 -0.2250

80 79 H61 HA E -1.7110 2.4520 -6.0680 0.1360

81 79 C56 CA M -3.3270 1.3890 -5.5160 0.2660

82 81 O4 OS M -3.9460 2.2080 -4.5150 -0.3210

83 82 C55 CT M -4.8700 1.8330 -3.5320 0.1210

84 83 H63 H1 E -5.7430 1.7130 -3.9120 0.0240

85 83 H64 H1 E -4.5790 0.9920 -3.1700 0.0240

86 83 C52 CT M -4.9020 2.8980 -2.4490 0.0200

87 86 H59 HC E -5.2550 3.7190 -2.8260 0.0120

88 86 H60 HC E -5.5050 2.6130 -1.7460 0.0120

89 86 C49 CT M -3.5510 3.1760 -1.8520 0.0200

90 89 H55 HC E -3.0330 3.7220 -2.4640 0.0120

91 89 H56 HC E -3.0770 2.3410 -1.7200 0.0120

92 89 C45 CT M -3.6890 3.9000 -0.5200 0.1210

93 92 H47 H1 E -4.2130 4.7080 -0.6270 0.0240

94 92 H48 H1 E -4.1270 3.3280 0.1300 0.0240

95 92 O3 OS M -2.3770 4.2140 -0.0970 -0.3210

96 95 C42 CA M -2.2080 4.5750 1.2350 0.2660

97 96 C39 CA B -3.2580 4.7430 2.1300 -0.2250

98 97 C35 CA S -2.9680 5.0550 3.4840 -0.1590

99 98 H40 HA E -3.6670 5.1390 4.0920 0.1490

100 97 H44 HA E -4.1390 4.6540 1.8450 0.1360

101 96 C38 CA M -0.9150 4.7370 1.6590 -0.2250

102 101 H43 HA E -0.2080 4.6020 1.0710 0.1360

103 101 C34 CA M -0.6670 5.1050 2.9860 -0.1590

104 103 H39 HA E 0.2080 5.2680 3.2560 0.1490

105 103 C30 CA M -1.6940 5.2330 3.9070 -0.0040

106 105 C26 CT M -1.3430 5.6600 5.3340 0.2000

107 106 C28 CT 3 -2.6240 5.9330 6.1300 -0.1780

108 107 H27 HC E -3.0870 6.6310 5.6600 0.0440

109 107 H28 HC E -3.1970 5.1670 6.2090 0.0440

110 107 H29 HC E -2.3820 6.2440 7.0070 0.0440

111 106 C29 CT 3 -0.5310 6.9570 5.2960 -0.1780

112 111 H30 HC E -1.0440 7.6630 4.8930 0.0440

113 111 H31 HC E -0.3190 7.1930 6.2020 0.0440

114 111 H32 HC E 0.2800 6.8300 4.8000 0.0440

115 106 C24 CA M -0.5150 4.5770 6.0570 0.0130

116 115 C20 CA M -0.0550 3.4270 5.4760 -0.1750

117 116 H23 HA E -0.3000 3.2590 4.5630 0.1380

118 116 C16 CA M 0.6980 2.4850 6.1600 -0.1750

119 118 H19 HA E 1.0150 1.7110 5.6890 0.1380

120 118 C14 CA M 1.0330 2.6380 7.4900 0.0130

121 120 C17 CA M 0.5870 3.8100 8.0780 -0.1750

122 121 H20 HA E 0.8070 3.9770 8.9970 0.1380

123 121 C21 CA M -0.1500 4.7640 7.3960 -0.1750

124 123 H24 HA E -0.4340 5.5610 7.8480 0.1380

IKUTOXg

1 0 C3 CA M -1.3050 0.4050 0.0070 -0.2110

2 1 H1 HA E -2.2480 0.4430 0.1800 0.1480

3 1 C1 CA M -0.2260 0.9950 0.9150 -0.2110

4 3 H9 HA E -0.2710 2.0050 1.3230 0.1480

5 3 C2 CA M 0.7090 0.2040 1.1410 0.1160

6 5 C4 CT 3 1.7890 0.8670 2.1150 -0.1320

7 6 H2 HC E 1.3280 1.2600 2.8590 0.0470

8 6 H3 HC E 2.3850 0.1840 2.4310 0.0470

9 6 H4 HC E 2.2920 1.5430 1.6560 0.0470

10 5 C5 CA M 1.3050 -0.4050 -0.0070 -0.2110

11 10 H5 HA E 2.2480 -0.4430 -0.1800 0.1480

12 10 C7 CA M 0.2260 -0.9950 -0.9150 -0.2110

13 12 H10 HA E 0.2710 -2.0050 -1.3230 0.1480

14 12 C6 CA M -0.7090 -0.2040 -1.1410 0.1160

15 14 C8 CT M -1.7890 -0.8670 -2.1150 -0.1320

16 15 H6 HC E -1.3280 -1.2600 -2.8590 0.0470

17 15 H7 HC E -2.3850 -0.1840 -2.4310 0.0470

18 15 H8 HC E -2.2920 -1.5430 -1.6560 0.0470

INUJACh

1 0 C5 CA M 2.8330 7.1870 6.0720 -0.2540

2 1 H5 HA E 3.6600 7.4460 6.4630 0.1650

3 1 C2 CA M 2.5890 7.4470 4.7400 -0.1630

4 3 H1 HA E 3.2480 7.8950 4.2210 0.1580

5 3 C1 CA M 1.3930 7.0630 4.1470 0.0070

6 5 C3 CA M 0.4520 6.4380 4.9480 -0.1630

7 6 H2 HA E -0.3780 6.1790 4.5660 0.1580

8 6 C6 CA M 0.6830 6.1820 6.2750 -0.2540

9 8 H6 HA E 0.0190 5.7520 6.8000 0.1650

10 8 C7 CA M 1.8800 6.5520 6.8370 0.3570

11 10 O1 OS M 2.1320 6.2450 8.1790 -0.4050

12 11 C9 CA M 1.7900 7.1890 9.1360 0.3570

13 12 C12 CA B 1.9140 6.8030 10.4580 -0.2540

14 13 C15 CA S 1.6400 7.6960 11.4580 -0.1630

15 14 H14 HA E 1.7260 7.4180 12.3630 0.1580

16 13 H12 HA E 2.1890 5.9180 10.6710 0.1650

17 12 C11 CA M 1.3750 8.4680 8.8300 -0.2540

18 17 H11 HA E 1.2690 8.7350 7.9260 0.1650

19 17 C14 CA M 1.1150 9.3580 9.8590 -0.1630

20 19 H13 HA E 0.8430 10.2430 9.6430 0.1580

21 19 C18 CA M 1.2390 9.0000 11.1850 0.0070

22 21 C21 CT M 0.9730 9.9570 12.3230 0.1210

23 22 H19 H1 E 0.4310 9.4820 13.0020 0.0540

24 22 H20 H1 E 1.8400 10.1800 12.7450 0.0540

25 22 N3 N M 0.3280 11.1430 12.0190 -0.6820

26 25 H21 H E -0.5520 11.1560 12.0070 0.3510

27 25 C23 C M 1.0050 12.2990 11.7350 0.8310

28 27 O4 O E 2.2210 12.3600 11.6890 -0.5770

29 27 N4 N M 0.1970 13.3840 11.5360 -0.6820

30 29 H22 H E -0.6580 13.3010 11.7280 0.3510

31 29 C27 CT M 0.6700 14.6820 11.0180 0.1210

32 31 H25 H1 E 1.6320 14.7760 11.2310 0.0540

33 31 H26 H1 E 0.1860 15.4070 11.4870 0.0540

34 31 C30 CA M 0.4820 14.8590 9.5230 0.0070

35 34 C29 CA B 0.5230 16.1250 8.9570 -0.1630

36 35 C26 CA S 0.3310 16.3220 7.6020 -0.2540

37 36 H24 HA E 0.3820 17.1970 7.2340 0.1650

38 35 H28 HA E 0.6860 16.8750 9.5140 0.1580

39 34 C28 CA M 0.2330 13.7850 8.6800 -0.1630

40 39 H27 HA E 0.2070 12.9050 9.0390 0.1580

41 39 C25 CA M 0.0220 13.9730 7.3280 -0.2540

42 41 H23 HA E -0.1530 13.2290 6.7630 0.1650

43 41 C24 CA M 0.0680 15.2470 6.7990 0.3570

44 43 O3 OS M -0.2260 15.5130 5.4660 -0.4050

45 44 C22 CA M 0.1570 14.5900 4.4960 0.3570

46 45 C20 CA B 1.4090 14.0000 4.5060 -0.2540

47 46 C17 CA S 1.7720 13.1650 3.4630 -0.1630

48 47 H16 HA E 2.6290 12.7560 3.4690 0.1580

49 46 H18 HA E 2.0130 14.1660 5.2210 0.1650

50 45 C19 CA M -0.7170 14.3590 3.4560 -0.2540

51 50 H17 HA E -1.5780 14.7610 3.4540 0.1650

52 50 C16 CA M -0.3320 13.5420 2.4180 -0.1630

53 52 H15 HA E -0.9280 13.4040 1.6920 0.1580

54 52 C13 CA M 0.9020 12.9160 2.4070 0.0070

55 54 C10 CT M 1.2980 11.9940 1.2760 0.1210

56 55 H9 H1 E 0.4960 11.8320 0.7200 0.0540

57 55 H10 H1 E 1.9570 12.4710 0.7100 0.0540

58 55 N2 N M 1.8390 10.7640 1.6160 -0.6820

59 58 H8 H E 2.7130 10.6610 1.6260 0.3510

60 58 C8 C M 1.0340 9.7010 1.9370 0.8310

61 60 O2 O E -0.1790 9.7720 1.9640 -0.5770

62 60 N1 N M 1.7240 8.5300 2.1410 -0.6820

63 62 H7 H E 2.5810 8.5270 1.9380 0.3510

64 62 C4 CT M 1.1540 7.2730 2.6680 0.1210

65 64 H3 H1 E 1.5470 6.5120 2.1710 0.0540

66 64 H4 H1 E 0.1790 7.2690 2.4990 0.0540

INUJACg

1 0 C1 CT M 0.2990 10.5260 5.3280 -0.2900

2 1 H1 HC E 0.2180 9.5710 5.1210 0.0980

3 1 H2 HC E -0.4180 10.7910 5.9400 0.0980

4 1 H3 HC E 0.2320 11.0470 4.5000 0.0980

5 1 C2 C M 1.6180 10.7830 5.9680 0.8040

6 5 O2 OH S 1.7080 11.1600 7.1220 -0.6620

7 6 H4 HO E 2.4030 11.1820 7.6350 0.4540

8 5 O1 O M 2.6800 10.5570 5.2160 -0.6010

ITAMIZh

1 0 C9 CT M 5.2250 10.8710 -0.1510 -0.1780

2 1 H5 HC E 6.1320 10.8640 -0.5220 0.0420

3 1 H6 HC E 4.7460 10.0680 -0.4450 0.0420

4 1 H7 HC E 4.7490 11.6680 -0.4650 0.0420

5 1 C5 CT M 5.3000 10.8910 1.3880 0.1840

6 5 H4 HC E 4.3590 10.9570 1.7200 0.0340

7 5 C2 CA M 5.8730 9.5940 1.9560 0.0020

8 7 C4 CA S 6.8170 8.8170 1.2780 -0.3620

9 8 H3 HA E 7.1430 9.1360 0.4450 0.2040

10 7 C1 CA M 5.4360 9.1210 3.1900 0.1640

11 10 O1 OH S 4.5890 9.9240 3.9300 -0.5520

12 11 H1 HO E 4.5230 9.6260 4.7020 0.4120

13 10 C3 CA M 5.8380 7.8860 3.6680 -0.2270

14 13 H2 HA E 5.4830 7.5540 4.4850 0.1660

15 13 C6 CA M 6.7610 7.1260 2.9550 0.1640

16 15 O2 OH S 7.0810 5.9060 3.4960 -0.5520

17 16 H8 HO E 7.8520 5.6230 3.3210 0.4120

18 15 C7 CA M 7.3070 7.6000 1.7550 0.0020

19 18 C10 CT M 8.4150 6.8360 1.0350 0.1840

20 19 C13 CT 3 8.3730 6.9920 -0.4820 -0.1780

21 20 H11 HC E 9.1050 6.4760 -0.8840 0.0420

22 20 H12 HC E 8.4750 7.9380 -0.7170 0.0420

23 20 H13 HC E 7.5150 6.6620 -0.8230 0.0420

24 19 H9 HC E 8.2720 5.8660 1.2310 0.0340

25 19 C14 CA M 9.7910 7.2160 1.6310 0.0020

26 25 C18 CA B 10.3430 6.4760 2.6800 0.1640

27 26 O4 OH S 9.6690 5.3560 3.1150 -0.5520

28 27 H18 HO E 10.0600 5.0470 3.7790 0.4120

29 26 C22 CA B 11.5420 6.8560 3.2710 -0.2270

30 29 C25 CA S 12.1990 7.9860 2.8270 0.1640

31 30 O6 OH S 13.3640 8.4110 3.4220 -0.5520

32 31 H26 HO E 13.5150 7.9390 4.0990 0.4120

33 29 H19 HA E 11.9090 6.3400 3.9790 0.1660

34 25 C17 CA M 10.5010 8.3320 1.1960 -0.3620

35 34 H16 HA E 10.1490 8.8380 0.4750 0.2040

36 34 C21 CA M 11.7060 8.7440 1.7690 0.0020

37 36 C26 CT M 12.4580 9.9930 1.2880 0.1840

38 37 C29 CT 3 12.5150 10.0750 -0.2360 -0.1780

39 38 H27 HC E 13.0020 10.8830 -0.5020 0.0420

40 38 H28 HC E 11.6040 10.1100 -0.5960 0.0420

41 38 H29 HC E 12.9760 9.2850 -0.5890 0.0420

42 37 H24 HC E 13.4020 9.8920 1.6010 0.0340

43 37 C30 CA M 11.9230 11.2720 1.9330 0.0020

44 43 C32 CA B 12.4160 11.7560 3.1370 0.1640

45 44 C31 CA B 11.9740 12.9670 3.6600 -0.2270

46 45 C28 CA S 11.0030 13.7020 2.9890 0.1640

47 46 O7 OH S 10.5630 14.9130 3.4780 -0.5520

48 47 H30 HO E 10.6500 14.9130 4.2860 0.4120

49 45 H31 HA E 12.3360 13.2900 4.4750 0.1660

50 44 O8 OH S 13.3650 11.1010 3.8900 -0.5520

51 50 H32 HO E 13.3370 10.2680 3.7790 0.4120

52 43 C27 CA M 10.9260 12.0310 1.3030 -0.3620

53 52 H25 HA E 10.5620 11.7020 0.4890 0.2040

54 52 C23 CA M 10.4370 13.2380 1.7990 0.0020

55 54 C19 CT M 9.3180 14.0100 1.1160 0.1840

56 55 C24 CT 3 9.3130 13.8390 -0.4040 -0.1780

57 56 H20 HC E 8.5800 14.3650 -0.7910 0.0420

58 56 H21 HC E 9.1870 12.8930 -0.6280 0.0420

59 56 H22 HC E 10.1670 14.1520 -0.7710 0.0420

60 55 H17 HC E 9.4760 14.9800 1.3000 0.0340

61 55 C15 CA M 7.9650 13.6490 1.7480 0.0020

62 61 C11 CA M 7.2660 12.5070 1.3750 -0.3620

63 62 H10 HA E 7.6600 11.9440 0.7200 0.2040

64 62 C8 CA M 6.0300 12.1290 1.8960 0.0020

65 64 C12 CA M 5.4840 12.9630 2.8890 0.1640

66 65 O3 OH S 4.2650 12.7170 3.4890 -0.5520

67 66 H14 HO E 4.1710 11.8820 3.5290 0.4120

68 65 C16 CA M 6.1540 14.0980 3.3000 -0.2270

69 68 H15 HA E 5.7740 14.6480 3.9760 0.1660

70 68 C20 CA M 7.3720 14.4470 2.7430 0.1640

71 70 O5 OH M 7.9510 15.6030 3.2030 -0.5520

72 71 H23 HO E 8.7590 15.4620 3.3340 0.4120

ITAMIZg

1 0 C5 CT M 8.2450 11.7460 4.6990 -0.0200

2 1 H10 HP E 8.8240 12.1760 4.0210 0.1360

3 1 H11 HP E 7.3780 11.5310 4.2720 0.1360

4 1 C1 CT M 8.0210 12.7010 5.8560 -0.0200

5 4 H1 HP E 7.0740 12.9880 5.8700 0.1360

6 4 H2 HP E 8.5860 13.5040 5.7380 0.1360

7 4 N1 N3 M 8.3660 12.0250 7.1550 -0.0270

8 7 C4 CT 3 8.0690 12.9370 8.3100 -0.2020

9 8 H7 HP E 8.6190 13.7460 8.2380 0.1570

10 8 H8 HP E 7.1210 13.1860 8.2970 0.1570

11 8 H9 HP E 8.2750 12.4780 9.1500 0.1570

12 7 C2 CT M 9.8110 11.6520 7.1550 -0.0200

13 12 H3 HP E 10.3650 12.4740 7.1690 0.1360

14 12 H4 HP E 10.0180 11.1300 7.9710 0.1360

15 12 C6 CT M 10.1530 10.8340 5.9310 -0.0200

16 15 H12 HP E 10.6200 10.0050 6.2040 0.1360

17 15 H13 HP E 10.7580 11.3510 5.3420 0.1360

18 15 N2 N3 M 8.8910 10.4890 5.1890 -0.0270

19 18 C8 CT 3 9.2080 9.5940 4.0310 -0.2020

20 19 H16 HP E 8.3820 9.3740 3.5510 0.1570

21 19 H17 HP E 9.6240 8.7700 4.3580 0.1570

22 19 H18 HP E 9.8260 10.0520 3.4230 0.1570

23 18 C7 CT M 7.9520 9.8150 6.1450 -0.0200

24 23 H14 HP E 8.3970 9.0290 6.5500 0.1360

25 23 H15 HP E 7.1510 9.4990 5.6580 0.1360

26 23 C3 CT M 7.5410 10.7780 7.2260 -0.0200

27 26 H5 HP E 7.6600 10.3520 8.1110 0.1360

28 26 H6 HP E 6.5840 11.0070 7.1190 0.1360

IXEKAXh

1 0 O2 O M 3.1530 4.8340 -0.0710 -0.5570

2 1 C2 C M 2.7460 5.9590 0.0680 0.8590

3 2 C5 CT 3 1.5310 6.5530 -0.5840 -0.4130

4 3 H1 HC E 1.7450 6.8290 -1.4900 0.1200

5 3 H2 HC E 1.2380 7.3230 -0.0710 0.1260

6 3 H3 HC E 0.8220 5.8910 -0.6090 0.1080

7 2 O1 OS M 3.3700 6.8850 0.8420 -0.4370

8 7 C1 CA M 4.6200 6.5750 1.4060 0.1460

9 8 C3 CA M 5.7060 6.3320 0.6320 -0.0330

10 9 C6 CT M 5.9130 6.1880 -0.8690 0.0110

11 10 H4 HC E 5.2150 6.5170 -1.4410 0.1100

12 10 C11 CT B 7.3080 6.8940 -0.9790 -0.2670

13 12 H6 HC E 7.2830 7.8180 -0.7170 0.1050

14 12 H7 HC E 7.7060 6.8040 -1.8480 0.1020

15 10 C10 CA M 6.3640 4.7330 -1.0780 -0.0440

16 15 C16 CA M 5.7290 3.6410 -1.6660 -0.1780

17 16 H10 HA E 4.8550 3.7140 -2.0590 0.1350

18 16 C20 CA M 6.4330 2.4410 -1.6660 0.0430

19 18 C26 CT M 6.0610 1.0340 -2.1580 -0.0140

20 19 H16 HC E 5.3650 0.9740 -2.8150 0.0850

21 19 C32 CT B 7.4770 0.5390 -2.5710 -0.2660

22 21 H21 HC E 7.8560 1.0500 -3.2910 0.0940

23 21 H22 HC E 7.4940 -0.3980 -2.7810 0.0900

24 19 C31 CA M 5.8800 0.2020 -0.8920 0.0400

25 24 C36 CA M 4.7690 -0.3440 -0.2810 -0.1450

26 25 H25 HA E 3.9020 -0.2740 -0.6870 0.1270

27 25 C40 CA M 4.9420 -0.9890 0.9420 -0.1840

28 27 H27 HA E 4.1830 -1.3840 1.3770 0.1470

29 27 C45 CA M 6.1810 -1.0850 1.5290 -0.1730

30 29 H32 HA E 6.2680 -1.5320 2.3730 0.1470

31 29 C41 CA M 7.3160 -0.5340 0.9230 -0.1730

32 31 H28 HA E 8.1800 -0.5930 1.3350 0.1310

33 31 C37 CA M 7.1490 0.0990 -0.2930 0.0480

34 33 C33 CT M 8.1180 0.8630 -1.1940 0.0110

35 34 H23 HC E 9.0530 0.6690 -1.0900 0.0760

36 34 C27 CA M 7.6970 2.3250 -1.0700 0.0130

37 36 C21 CA M 8.3090 3.4080 -0.4500 -0.2200

38 37 H12 HA E 9.1650 3.3250 -0.0250 0.1410

39 37 C17 CA M 7.6310 4.6150 -0.4870 -0.0080

40 39 C12 CT M 7.9750 5.9850 0.0930 0.0280

41 40 H8 HC E 8.9010 6.1540 0.2850 0.1030

42 40 C7 CA M 6.9890 6.2050 1.2310 0.0230

43 42 C13 CA M 7.1430 6.3110 2.5730 0.1310

44 43 O3 OS S 8.3910 6.0720 3.1850 -0.3970

45 44 C22 C B 9.3140 7.0720 3.1900 0.7860

46 45 O4 O E 9.0970 8.1400 2.7320 -0.5710

47 45 C28 CT 3 10.5730 6.6000 3.8200 -0.3250

48 47 H17 HC E 11.2390 7.3040 3.7870 0.0960

49 47 H18 HC E 10.9000 5.8230 3.3400 0.0990

50 47 H19 HC E 10.4030 6.3600 4.7440 0.1020

51 43 C8 CA M 6.0270 6.5700 3.4180 -0.0300

52 51 C14 CA S 6.1690 6.7100 4.8320 -0.1040

53 52 H9 HA E 7.0330 6.6480 5.2450 0.1230

54 51 C4 CA M 4.7300 6.6870 2.8260 -0.0300

55 54 C9 CA M 3.5880 6.8770 3.6570 -0.1070

56 55 H5 HA E 2.7110 6.9360 3.2740 0.1190

57 55 C15 CA M 3.7610 6.9810 5.0120 -0.0150

58 57 C18 CA M 5.0610 6.9320 5.5880 -0.0240

59 58 C23 CT M 4.8660 7.0030 7.1060 0.0090

60 59 H13 HC E 5.6250 7.2580 7.6350 0.0950

61 59 C25 CT M 3.6120 7.9200 7.1590 -0.2900

62 61 H14 HC E 3.7850 8.8020 6.8180 0.1070

63 61 H15 HC E 3.2280 7.9710 8.0380 0.1070

64 61 C19 CT M 2.7780 7.0520 6.1820 0.0330

65 64 H11 HC E 1.8850 7.3450 5.9880 0.0790

66 64 C24 CA M 2.9250 5.6870 6.8630 0.0210

67 66 C30 CA S 2.1090 4.5620 6.8710 -0.2400

68 67 H20 HA E 1.2280 4.5770 6.4890 0.1480

69 66 C29 CA M 4.2160 5.6610 7.4270 -0.0020

70 69 C34 CA M 4.7400 4.5090 7.9940 -0.2280

71 70 H24 HA E 5.6220 4.4900 8.3740 0.1450

72 70 C38 CA M 3.9260 3.3910 8.0080 0.0250

73 72 C42 CT B 4.2020 1.9460 8.4340 0.0190

74 73 C44 CT B 2.7630 1.5350 8.8740 -0.2520

75 74 H30 HC E 2.4470 2.0350 9.6300 0.0880

76 74 H31 HC E 2.6780 0.5940 9.0360 0.0870

77 73 H29 HC E 4.9130 1.8070 9.0660 0.0750

78 72 C35 CA M 2.6340 3.4080 7.4610 0.0180

79 78 C39 CT M 2.1030 1.9780 7.5420 0.0250

80 79 H26 HC E 1.1530 1.8640 7.4660 0.0720

81 79 C43 CA M 2.9730 1.1790 6.5790 0.0390

82 81 C46 CA M 4.2730 1.1670 7.1190 0.0270

83 82 C48 CA M 5.3200 0.5890 6.4370 -0.1630

84 83 H34 HA E 6.2050 0.5940 6.8080 0.1300

85 83 C50 CA M 5.0530 -0.0070 5.1970 -0.1640

86 85 H36 HA E 5.7720 -0.4130 4.7070 0.1470

87 85 C49 CA M 3.7840 -0.0170 4.6760 -0.1900

88 87 H35 HA E 3.6220 -0.4450 3.8310 0.1530

89 87 C47 CA M 2.7220 0.5890 5.3530 -0.1600

90 89 H33 HA E 1.8370 0.5950 4.9800 0.1310

IXEKAXg

1 0 N1 N1 M 9.6750 2.4490 2.6960 -0.4300

2 1 C1 CZ M 8.7840 2.7500 3.3480 0.4130

3 2 C2 CM M 7.6450 3.1000 4.1540 -0.2120

4 3 C4 CZ S 7.9000 3.5330 5.4930 0.4130

5 4 N2 N1 E 8.0680 3.8680 6.5830 -0.4300

6 3 C3 CA M 6.3660 3.0640 3.6500 0.1940

7 6 C6 CA B 6.1210 2.6330 2.2890 -0.1250

8 7 C8 CA S 4.8660 2.5650 1.8090 -0.1250

9 8 H4 HA E 4.7150 2.2780 0.9060 0.1510

10 7 H2 HA E 6.8630 2.3980 1.7250 0.1510

11 6 C5 CA M 5.2450 3.4580 4.4600 -0.1250

12 11 H1 HA E 5.3920 3.7570 5.3600 0.1510

13 11 C7 CA M 3.9960 3.4020 3.9730 -0.1250

14 13 H3 HA E 3.2610 3.6960 4.5160 0.1510

15 13 C9 CA M 3.7390 2.9160 2.6370 0.1940

16 15 C10 CM M 2.4520 2.7700 2.1780 -0.2120

17 16 C12 CZ S 2.1540 2.2050 0.8910 0.4130

18 17 N4 N1 E 1.9110 1.7150 -0.1130 -0.4300

19 16 C11 CZ M 1.3190 3.1730 2.9580 0.4130

20 19 N3 N1 M 0.4320 3.5200 3.5850 -0.4300

IXEKEBh

1 0 O2 O M 4.8860 0.4240 11.9660 -0.5570

2 1 C2 C M 3.9230 -0.1150 11.5200 0.8590

3 2 C5 CT 3 3.6090 -1.5680 11.5940 -0.4130

4 3 H1 HC E 3.3120 -1.8770 10.7220 0.1200

5 3 H2 HC E 2.9040 -1.7130 12.2460 0.1260

6 3 H3 HC E 4.4020 -2.0600 11.8580 0.1080

7 2 O1 OS M 2.9230 0.5400 10.8580 -0.4370

8 7 C1 CA M 2.9130 1.9510 10.8270 0.1460

9 8 C3 CA M 3.8910 2.6950 10.2490 -0.0330

10 9 C6 CT M 5.2540 2.4040 9.6040 0.0110

11 10 H4 HC E 5.4300 1.4950 9.3520 0.1100

12 10 C11 CT B 5.2060 3.4600 8.4740 -0.2670

13 12 H6 HC E 4.4970 3.3100 7.8440 0.1050

14 12 H7 HC E 6.0450 3.5480 8.0150 0.1020

15 10 C10 CA M 6.2900 3.0720 10.5140 -0.0440

16 15 C16 CA M 7.2340 2.5370 11.3790 -0.1780

17 16 H10 HA E 7.3350 1.5890 11.4850 0.1350

18 16 C20 CA M 8.0140 3.4410 12.0890 0.0430

19 18 C26 CT M 9.0610 3.2420 13.1880 -0.0140

20 19 H16 HC E 9.4980 2.3880 13.2230 0.0850

21 19 C32 CT B 9.9500 4.4780 12.9260 -0.2660

22 21 H21 HC E 10.6060 4.6280 13.6110 0.0940

23 21 H22 HC E 10.3740 4.4520 12.0630 0.0900

24 19 C31 CA M 8.3560 3.7130 14.4660 0.0400

25 24 C36 CA M 7.8410 3.0370 15.5500 -0.1450

26 25 H25 HA E 7.9390 2.0850 15.6130 0.1270

27 25 C40 CA M 7.2030 3.7600 16.5430 -0.1840

28 27 H27 HA E 6.8400 3.3050 17.3070 0.1470

29 27 C45 CA M 7.0730 5.1290 16.4460 -0.1730

30 29 H32 HA E 6.6500 5.6110 17.1610 0.1470

31 29 C41 CA M 7.5550 5.8150 15.3290 -0.1730

32 31 H28 HA E 7.4400 6.7630 15.2390 0.1310

33 31 C37 CA M 8.1990 5.0960 14.3510 0.0480

34 33 C33 CT M 8.7760 5.4790 12.9850 0.0110

35 34 H23 HC E 8.9780 6.4070 12.8470 0.0760

36 34 C27 CA M 7.8370 4.8210 11.9590 0.0130

37 36 C21 CA M 6.8630 5.3550 11.1310 -0.2200

38 37 H12 HA E 6.7090 6.3000 11.0740 0.1410

39 37 C17 CA M 6.1070 4.4460 10.3970 -0.0080

40 39 C12 CT M 4.9340 4.6410 9.4290 0.0280

41 40 H8 HC E 4.8430 5.5110 9.0350 0.1030

42 40 C7 CA M 3.7150 4.0980 10.1470 0.0230

43 42 C13 CA M 2.6090 4.7080 10.6380 0.1310

44 43 O3 OS S 2.5030 6.1090 10.5510 -0.3970

45 44 C22 C B 1.9090 6.6140 9.4250 0.7860

46 45 O4 O E 1.3790 5.9190 8.6190 -0.5710

47 45 C28 CT 3 2.0570 8.0870 9.3750 -0.3250

48 47 H17 HC E 1.3170 8.4710 8.8770 0.0960

49 47 H18 HC E 2.0580 8.4420 10.2780 0.0990

50 47 H19 HC E 2.8950 8.3130 8.9380 0.1020

51 43 C8 CA M 1.5930 3.9650 11.2950 -0.0300

52 51 C14 CA S 0.4690 4.5910 11.9010 -0.1040

53 52 H9 HA E 0.3690 5.5440 11.8600 0.1230

54 51 C4 CA M 1.7380 2.5510 11.3700 -0.0300

55 54 C9 CA M 0.7190 1.7870 12.0000 -0.1070

56 55 H5 HA E 0.7820 0.8300 12.0290 0.1190

57 55 C15 CA M -0.3490 2.4170 12.5590 -0.0150

58 57 C18 CA M -0.4650 3.8310 12.5360 -0.0240

59 58 C23 CT M -1.7190 4.1680 13.3530 0.0090

60 59 H13 HC E -2.0920 5.0440 13.2310 0.0950

61 59 C25 CT M -2.6000 2.9610 12.9520 -0.2900

62 61 H14 HC E -2.8080 2.9440 12.0130 0.1070

63 61 H15 HC E -3.4050 2.8960 13.4710 0.1070

64 61 C19 CT M -1.5440 1.9050 13.3560 0.0330

65 64 H11 HC E -1.7790 0.9820 13.2380 0.0790

66 64 C24 CA M -1.2590 2.3690 14.7880 0.0210

67 66 C30 CA S -0.8210 1.6610 15.9020 -0.2400

68 67 H20 HA E -0.7150 0.7070 15.8860 0.1480

69 66 C29 CA M -1.3590 3.7570 14.7890 -0.0020

70 69 C34 CA M -1.0280 4.5040 15.9190 -0.2280

71 70 H24 HA E -1.0800 5.4610 15.9220 0.1450

72 70 C38 CA M -0.6210 3.7980 17.0480 0.0250

73 72 C42 CT B -0.0770 4.2540 18.4150 0.0190

74 73 C44 CT B -0.5980 3.0740 19.2860 -0.2520

75 74 H30 HC E -1.5570 3.0250 19.3290 0.0880

76 74 H31 HC E -0.2270 3.0810 20.1720 0.0870

77 73 H29 HC E -0.3030 5.1440 18.6930 0.0750

78 72 C35 CA M -0.5340 2.4070 17.0380 0.0180

79 78 C39 CT M 0.0260 1.9870 18.3900 0.0250

80 79 H26 HC E -0.1130 1.0740 18.6490 0.0720

81 79 C43 CA M 1.4720 2.4840 18.3680 0.0390

82 81 C46 CA M 1.4110 3.8830 18.3920 0.0270

83 82 C48 CA M 2.5730 4.6270 18.3510 -0.1630

84 83 H34 HA E 2.5350 5.5840 18.4050 0.1300

85 83 C50 CA M 3.7880 3.9610 18.2340 -0.1640

86 85 H36 HA E 4.6030 4.4680 18.1940 0.1470

87 85 C49 CA M 3.8410 2.5830 18.1780 -0.1900

88 87 H35 HA E 4.6930 2.1470 18.0830 0.1530

89 87 C47 CA M 2.6780 1.8250 18.2540 -0.1600

90 89 H33 HA E 2.7050 0.8670 18.2310 0.1310

IXEKEBg

1 0 C5 CA M 2.6480 4.9320 14.5700 -0.1460

2 1 H3 HA E 2.0570 5.6610 14.7750 0.1710

3 1 C2 CA M 2.2820 3.6310 14.8410 -0.1460

4 3 H1 HA E 1.4280 3.4410 15.2370 0.1710

5 3 C1 CA M 3.1410 2.5990 14.5440 0.0670

6 5 C4 CZ S 2.7220 1.2300 14.7130 0.3200

7 6 N1 N1 E 2.3910 0.1380 14.7830 -0.4380

8 5 C3 CA M 4.4060 2.8570 14.0100 -0.1460

9 8 H2 HA E 5.0170 2.1360 13.8420 0.1710

10 8 C6 CA M 4.7600 4.1520 13.7400 -0.1460

11 10 H4 HA E 5.6230 4.3470 13.3670 0.1710

12 10 C7 CA M 3.8860 5.1900 13.9970 0.0670

13 12 C8 CZ M 4.2620 6.5290 13.6280 0.3200

14 13 N2 N1 M 4.5750 7.5840 13.2950 -0.4380

JAXPONh

1 0 C37 CT M 5.1910 7.9220 11.9590 -0.2050

2 1 H27 HC E 4.6590 7.2090 12.3700 0.0440

3 1 H28 HC E 5.1180 7.8620 10.9830 0.0440

4 1 H29 HC E 4.8580 8.7940 12.2590 0.0440

5 1 C30 CT M 6.6570 7.7680 12.3710 0.3360

6 5 C38 CT 3 6.7240 7.6830 13.9020 -0.2050

7 6 H30 HC E 6.1070 6.9920 14.2190 0.0440

8 6 H31 HC E 6.4730 8.5470 14.2880 0.0440

9 6 H32 HC E 7.6370 7.4570 14.1770 0.0440

10 5 C39 CT 3 7.1880 6.4550 11.7670 -0.2050

11 10 H33 HC E 6.6450 5.7050 12.0870 0.0440

12 10 H34 HC E 7.1360 6.5010 10.7890 0.0440

13 10 H35 HC E 8.1200 6.3220 12.0390 0.0440

14 5 C23 CA M 7.5300 8.9080 11.8320 0.0270

15 14 C18 CA B 7.1530 9.6640 10.7230 -0.1750

16 15 C11 CA S 7.9980 10.5980 10.1500 -0.1740

17 16 H8 HA E 7.7140 11.0990 9.3940 0.1660

18 15 H13 HA E 6.2890 9.5330 10.3490 0.1560

19 14 C17 CA M 8.7850 9.1800 12.3850 -0.1750

20 19 H12 HA E 9.0530 8.7150 13.1680 0.1560

21 19 C10 CA M 9.6500 10.1090 11.8260 -0.1740

22 21 H7 HA E 10.5000 10.2710 12.2180 0.1660

23 21 C6 CA M 9.2590 10.7990 10.6850 0.0270

24 23 S1 S6 M 10.3900 11.8670 9.8530 0.8470

25 24 O1 O E 9.6320 12.7700 9.0400 -0.4990

26 24 O2 O E 11.3230 12.3730 10.8150 -0.4990

27 24 N1 NT M 11.2580 10.9450 8.8140 -0.1530

28 27 C3 CT B 10.5660 10.4320 7.6110 -0.0740

29 28 H3 H1 E 9.7280 10.9410 7.4710 0.0780

30 28 H4 H1 E 10.3260 9.4820 7.7500 0.0780

31 27 C1 CT M 12.3280 10.0920 9.3630 -0.0740

32 31 H1 H1 E 12.0740 9.1410 9.2590 0.0780

33 31 H2 H1 E 12.4310 10.2780 10.3300 0.0780

34 31 C2 CA M 13.6520 10.3370 8.6610 0.0260

35 34 C5 CA S 14.4400 9.2750 8.2530 -0.0930

36 35 H6 HA E 14.1480 8.3890 8.4350 0.1360

37 34 C4 CA M 14.0590 11.6420 8.3870 -0.0900

38 37 H5 HA E 13.5290 12.3800 8.6660 0.1180

39 37 C8 CA M 15.2530 11.8530 7.7020 -0.1790

40 39 C15 C B 15.6890 13.2220 7.2930 0.7560

41 40 O3 O E 16.7110 13.4340 6.6850 -0.5560

42 40 O4 OS S 14.8110 14.1690 7.6400 -0.3740

43 42 C27 CT 3 15.0570 15.5030 7.0880 0.1270

44 43 H17 H1 E 14.6920 16.1910 7.6990 0.0490

45 43 H18 H1 E 16.0310 15.6540 6.9990 0.0490

46 43 C33 CT 3 14.4010 15.6240 5.7400 -0.0620

47 46 H21 HC E 14.5580 16.5210 5.3800 0.0290

48 46 H22 HC E 14.7820 14.9570 5.1320 0.0290

49 46 H23 HC E 13.4380 15.4720 5.8320 0.0290

50 39 C14 CA M 16.0420 10.7800 7.3090 -0.0670

51 50 H11 HA E 16.8580 10.9420 6.8500 0.1110

52 50 C9 CA M 15.6530 9.4660 7.5800 -0.0280

53 52 C16 CA M 16.4650 8.3050 7.1290 -0.0280

54 53 C22 CA S 17.3430 8.3940 6.0380 -0.0930

55 54 H15 HA E 17.4620 9.2340 5.6090 0.1360

56 53 C21 CA M 16.3520 7.0670 7.7640 -0.0670

57 56 H14 HA E 15.7780 6.9790 8.5160 0.1110

58 56 C28 CA M 17.0660 5.9610 7.3180 -0.1790

59 58 C35 C B 16.8960 4.6930 8.0990 0.7560

60 59 O7 O E 16.0510 4.5430 8.9460 -0.5560

61 59 O8 OS S 17.7980 3.7590 7.7610 -0.3740

62 61 C47 CT 3 17.7320 2.5170 8.5340 0.1270

63 62 H44 H1 E 16.7850 2.2790 8.6990 0.0490

64 62 H45 H1 E 18.1470 1.7820 8.0170 0.0490

65 62 C48 CT 3 18.4450 2.6750 9.8450 -0.0620

66 65 H46 HC E 18.0160 3.3840 10.3680 0.0290

67 65 H47 HC E 19.3820 2.9120 9.6810 0.0290

68 65 H48 HC E 18.4040 1.8310 10.3430 0.0290

69 58 C34 CA M 17.9060 6.0630 6.2110 -0.0900

70 69 H24 HA E 18.3800 5.3010 5.8980 0.1180

71 69 C29 CA M 18.0420 7.2930 5.5670 0.0260

72 71 C36 CT M 18.9170 7.4150 4.3350 -0.0740

73 72 H25 H1 E 19.7540 6.9060 4.4750 0.0780

74 72 H26 H1 E 19.1560 8.3650 4.1950 0.0780

75 72 N2 NT M 18.2250 6.9020 3.1320 -0.1530

76 75 S2 S6 3 19.0930 5.9800 2.0930 0.8470

77 76 O11 O E 19.8510 5.0770 2.9060 -0.4990

78 76 O12 O E 18.1600 5.4730 1.1310 -0.4990

79 76 C49 CA S 20.2230 7.0480 1.2610 0.0270

80 79 C51 CA B 19.8330 7.7380 0.1200 -0.1740

81 80 H51 HA E 18.9830 7.5760 -0.2720 0.1660

82 80 C54 CA B 20.6980 8.6670 -0.4390 -0.1750

83 82 H56 HA E 20.4290 9.1320 -1.2220 0.1560

84 82 C56 CA B 21.9520 8.9390 0.1140 0.0270

85 84 C55 CA B 22.3300 8.1830 1.2230 -0.1750

86 85 C52 CA S 21.4850 7.2490 1.7960 -0.1740

87 86 H52 HA E 21.7690 6.7480 2.5520 0.1660

88 85 H57 HA E 23.1930 8.3140 1.5970 0.1560

89 84 C57 CT 3 22.8250 10.0790 -0.4260 0.3360

90 89 C58 CT 3 24.2920 9.9250 -0.0130 -0.2050

91 90 H58 HC E 24.8240 10.6380 -0.4240 0.0440

92 90 H59 HC E 24.3650 9.9850 0.9630 0.0440

93 90 H60 HC E 24.6250 9.0530 -0.3130 0.0440

94 89 C59 CT 3 22.7580 10.1640 -1.9570 -0.2050

95 94 H61 HC E 23.0100 9.3000 -2.3430 0.0440

96 94 H62 HC E 23.3760 10.8550 -2.2730 0.0440

97 94 H63 HC E 21.8450 10.3900 -2.2310 0.0440

98 89 C60 CT 3 22.2950 11.3920 0.1790 -0.2050

99 98 H64 HC E 22.8380 12.1420 -0.1410 0.0440

100 98 H65 HC E 22.3470 11.3460 1.1560 0.0440

101 98 H66 HC E 21.3630 11.5240 -0.0930 0.0440

102 75 C46 CT M 17.1550 7.7550 2.5830 -0.0740

103 102 H42 H1 E 17.4090 8.7060 2.6870 0.0780

104 102 H43 H1 E 17.0520 7.5690 1.6160 0.0780

105 102 C42 CA M 15.8310 7.5100 3.2850 0.0260

106 105 C44 CA B 15.4230 6.2050 3.5590 -0.0900

107 106 C41 CA B 14.2300 5.9940 4.2440 -0.1790

108 107 C31 CA S 13.4400 7.0670 4.6370 -0.0670

109 108 H19 HA E 12.6240 6.9050 5.0960 0.1110

110 107 C45 C B 13.7930 4.6250 4.6530 0.7560

111 110 O9 O E 12.7710 4.4130 5.2610 -0.5560

112 110 O10 OS S 14.6720 3.6780 4.3050 -0.3740

113 112 C50 CT 3 14.4260 2.3440 4.8580 0.1270

114 113 H49 H1 E 14.7910 1.6560 4.2470 0.0490

115 113 H50 H1 E 13.4510 2.1930 4.9470 0.0490

116 113 C53 CT 3 15.0810 2.2230 6.2050 -0.0620

117 116 H53 HC E 14.9250 1.3260 6.5650 0.0290

118 116 H54 HC E 14.7010 2.8900 6.8140 0.0290

119 116 H55 HC E 16.0440 2.3750 6.1140 0.0290

120 106 H41 HA E 15.9540 5.4670 3.2800 0.1180

121 105 C32 CA M 15.0430 8.5720 3.6930 -0.0930

122 121 H20 HA E 15.3350 9.4580 3.5110 0.1360

123 121 C26 CA M 13.8300 8.3810 4.3660 -0.0280

124 123 C20 CA M 13.0180 9.5420 4.8170 -0.0280

125 124 C24 CA S 13.1300 10.7790 4.1820 -0.0670

126 125 H16 HA E 13.7050 10.8680 3.4300 0.1110

127 124 C13 CA M 12.1400 9.4530 5.9080 -0.0930

128 127 H10 HA E 12.0210 8.6130 6.3370 0.1360

129 127 C7 CA M 11.4400 10.5540 6.3790 0.0260

130 129 C12 CA M 11.5770 11.7840 5.7340 -0.0900

131 130 H9 HA E 11.1030 12.5460 6.0480 0.1180

132 130 C19 CA M 12.4170 11.8860 4.6280 -0.1790

133 132 C25 C M 12.5870 13.1540 3.8470 0.7560

134 133 O5 O E 13.4310 13.3040 3.0000 -0.5560

135 133 O6 OS M 11.6840 14.0880 4.1840 -0.3740

136 135 C40 CT M 11.7500 15.3300 3.4110 0.1270

137 136 H36 H1 E 12.6970 15.5680 3.2470 0.0490

138 136 H37 H1 E 11.3350 16.0650 3.9290 0.0490

139 136 C43 CT M 11.0370 15.1720 2.1010 -0.0620

140 139 H38 HC E 10.1010 14.9350 2.2650 0.0290

141 139 H39 HC E 10.1010 14.9350 2.2650 0.0290

142 139 H40 HC E 10.1010 14.9350 2.2650 0.0290

JAXPONg

1 0 C1 CA M 11.0960 6.1050 7.8480 -0.1210

2 1 H1 HA E 10.7780 5.9590 6.9640 0.1210

3 1 C2 CA M 12.3760 5.7940 8.1660 -0.1210

4 3 H2 HA E 12.9480 5.4280 7.5020 0.1210

5 3 C4 CA M 12.8580 5.9980 9.4300 -0.1210

6 5 H4 HA E 13.7560 5.7690 9.6370 0.1210

7 5 C6 CA M 12.0490 6.5310 10.3960 -0.1210

8 7 H6 HA E 12.3900 6.6850 11.2700 0.1210

9 7 C5 CA M 10.7370 6.8460 10.0990 -0.1210

10 9 H5 HA E 10.1670 7.2030 10.7690 0.1210

11 9 C3 CA M 10.2550 6.6340 8.8030 -0.1210

12 11 H3 HA E 9.3580 6.8520 8.5840 0.1210

JEBTALh

1 0 C1 CT M -3.5150 2.7090 12.1750 0.0670

2 1 H1 H1 E -3.9750 3.5370 12.3260 0.0520

3 1 H2 H1 E -2.5740 2.8350 12.3200 0.0520

4 1 O1 OS M -4.0150 1.7070 13.0590 -0.3350

5 4 C3 CA M -3.5730 1.6810 14.3530 0.2470

6 5 C7 CA B -3.8920 0.5420 15.0650 -0.1920

7 6 C11 CA S -3.4800 0.3940 16.3670 -0.1940

8 7 H9 HA E -3.7170 -0.3950 16.8590 0.1670

9 6 H6 HA E -4.4080 -0.1510 14.6480 0.1520

10 5 C6 CA M -2.8410 2.6860 14.9600 -0.1920

11 10 H5 HA E -2.6270 3.4900 14.4790 0.1520

12 10 C10 CA M -2.4230 2.5130 16.2730 -0.1940

13 12 H8 HA E -1.9120 3.2050 16.7000 0.1670

14 12 C14 CA M -2.7210 1.3800 16.9630 0.1610

15 14 O3 OS M -2.3000 1.2510 18.3010 -0.2560

16 15 C16 CA M -1.2870 0.2870 18.4540 0.1610

17 16 C20 CA B 0.0180 0.6110 18.1470 -0.1940

18 17 C24 CA S 1.0150 -0.3440 18.2500 -0.1920

19 18 H20 HA E 1.9250 -0.1160 18.0480 0.1520

20 17 H16 HA E 0.2370 1.5020 17.8650 0.1670

21 16 C19 CA M -1.5920 -0.9670 18.9340 -0.1940

22 21 H15 HA E -2.4900 -1.1700 19.2050 0.1670

23 21 C23 CA M -0.6130 -1.9310 19.0230 -0.1920

24 23 H19 HA E -0.8300 -2.8090 19.3480 0.1520

25 23 C26 CA M 0.6870 -1.6290 18.6440 0.2470

26 25 O5 OS M 1.5640 -2.6940 18.6580 -0.3350

27 26 C28 CT M 2.9130 -2.4550 18.1850 0.0670

28 27 H21 H1 E 2.8890 -1.8720 17.4220 0.0520

29 27 H22 H1 E 3.4320 -2.0500 18.8830 0.0520

30 27 C31 CA M 3.5220 -3.7600 17.7980 0.0260

31 30 C35 CA S 3.3680 -4.2310 16.5120 -0.1380

32 31 H28 HA E 2.8510 -3.7140 15.8890 0.1210

33 30 C34 CA M 4.2840 -4.5180 18.6870 -0.1170

34 33 H27 HA E 4.4000 -4.2190 19.5920 0.1460

35 33 C37 CA M 4.8690 -5.7020 18.2710 -0.2140

36 35 H29 HA E 5.3920 -6.2230 18.8830 0.1560

37 35 C39 CA M 4.7010 -6.1250 16.9770 -0.1170

38 37 H30 HA E 5.1290 -6.9340 16.6890 0.1460

39 37 C38 CA M 3.9310 -5.4210 16.0850 0.0260

40 39 C40 CT M 3.6900 -5.8910 14.6780 0.0670

41 40 H31 H1 E 4.1490 -6.7190 14.5280 0.0520

42 40 H32 H1 E 2.7490 -6.0170 14.5330 0.0520

43 40 O6 OS M 4.1890 -4.8890 13.7950 -0.3350

44 43 C36 CA M 3.7480 -4.8630 12.5000 0.2470

45 44 C33 CA B 4.0660 -3.7240 11.7890 -0.1920

46 45 C30 CA S 3.6550 -3.5760 10.4860 -0.1940

47 46 H24 HA E 3.8920 -2.7870 9.9950 0.1670

48 45 H26 HA E 4.5830 -3.0310 12.2050 0.1520

49 44 C32 CA M 3.0150 -5.8680 11.8930 -0.1920

50 49 H25 HA E 2.8020 -6.6720 12.3740 0.1520

51 49 C29 CA M 2.5980 -5.6950 10.5800 -0.1940

52 51 H23 HA E 2.0870 -6.3870 10.1530 0.1670

53 51 C27 CA M 2.8950 -4.5620 9.8900 0.1610

54 53 O4 OS M 2.4740 -4.4330 8.5530 -0.2560

55 54 C25 CA M 1.4610 -3.4690 8.4000 0.1610

56 55 C22 CA B 0.1570 -3.7930 8.7060 -0.1940

57 56 C18 CA S -0.8400 -2.8380 8.6040 -0.1920

58 57 H14 HA E -1.7500 -3.0660 8.8050 0.1520

59 56 H18 HA E -0.0620 -4.6840 8.9880 0.1670

60 55 C21 CA M 1.7670 -2.2150 7.9190 -0.1940

61 60 H17 HA E 2.6640 -2.0120 7.6480 0.1670

62 60 C17 CA M 0.7880 -1.2510 7.8300 -0.1920

63 62 H13 HA E 1.0040 -0.3730 7.5050 0.1520

64 62 C15 CA M -0.5130 -1.5530 8.2090 0.2470

65 64 O2 OS M -1.3890 -0.4880 8.1960 -0.3350

66 65 C13 CT M -2.7380 -0.7270 8.6680 0.0670

67 66 H11 H1 E -2.7140 -1.3100 9.4310 0.0520

68 66 H12 H1 E -3.2580 -1.1320 7.9700 0.0520

69 66 C9 CA M -3.3470 0.5780 9.0550 0.0260

70 69 C5 CA M -3.1930 1.0490 10.3410 -0.1380

71 70 H4 HA E -2.6760 0.5320 10.9640 0.1210

72 70 C2 CA M -3.7570 2.2390 10.7680 0.0260

73 72 C4 CA M -4.5260 2.9430 9.8770 -0.1170

74 73 H3 HA E -4.9550 3.7520 10.1640 0.1460

75 73 C8 CA M -4.6940 2.5200 8.5820 -0.2140

76 75 H7 HA E -5.2170 3.0410 7.9700 0.1560

77 75 C12 CA M -4.1090 1.3360 8.1660 -0.1170

78 77 H10 HA E -4.2250 1.0370 7.2610 0.1460

JEBTALg

1 0 C1 CA M 0.4260 -2.4240 12.4220 -0.1210

2 1 H1 HA E 0.6740 -3.0140 11.7050 0.1210

3 1 C2 CA M 0.7240 -2.7400 13.6980 -0.1210

4 3 H2 HA E 1.1760 -3.5650 13.8910 0.1210

5 3 C4 CA M 0.3980 -1.9170 14.6910 -0.1210

6 5 H4 HA E 0.6260 -2.1470 15.5940 0.1210

7 5 C6 CA M -0.2520 -0.7580 14.4310 -0.1210

8 7 H6 HA E -0.4990 -0.1680 15.1480 0.1210

9 7 C5 CA M -0.5500 -0.4420 13.1550 -0.1210

10 9 H5 HA E -1.0010 0.3830 12.9620 0.1210

11 9 C3 CA M -0.2230 -1.2650 12.1620 -0.1210

12 11 H3 HA E -0.4510 -1.0350 11.2600 0.1210

JEJWOKh

1 0 C9 CT M -2.7850 7.1600 3.7100 0.0030

2 1 H13 H1 E -1.8000 6.8890 4.0900 0.0550

3 1 H64 H1 E -3.5190 6.4310 4.0530 0.0550

4 1 H82 H1 E -2.7630 7.1700 2.6200 0.0550

5 1 O5 OS M -3.1540 8.5070 4.2100 -0.3000

6 5 C6 CT M -2.6270 8.8140 5.4640 -0.0170

7 6 H6 H1 E -3.2280 9.5390 5.9180 0.0860

8 6 H7 H1 E -2.7150 8.0710 6.0240 0.0860

9 6 C5 CT M -1.1440 9.3420 5.3750 0.0200

10 9 O4 OS E -1.2760 10.6230 4.7420 -0.3150

11 9 H5 H1 E -0.4070 8.7580 4.7930 0.1480

12 9 C4 CT M -0.5380 9.4840 6.7770 -0.0070

13 12 O3 OS E -0.1610 8.1940 7.2540 -0.2360

14 12 H4 H1 E -1.1440 9.9170 7.4410 0.1440

15 12 C3 CT M 0.7140 10.3550 6.7520 -0.0350

16 15 O2 OS S 0.9190 10.7740 8.1380 -0.3450

17 16 C8 CT 3 2.2710 11.0530 8.5330 -0.0150

18 17 H12 H1 E 2.8850 11.2000 7.6450 0.0650

19 17 H63 H1 E 2.6620 10.2150 9.1100 0.0650

20 17 H81 H1 E 2.2930 11.9560 9.1430 0.0650

21 15 H3 H1 E 1.5830 9.8230 6.4740 0.1660

22 15 C2 CT M 0.6010 11.5770 5.8530 0.0310

23 22 O1 OS S 1.8770 12.1560 5.7410 -0.2340

24 23 C7 CT 3 1.8640 13.5650 5.4550 -0.1270

25 24 H11 H1 E 1.6670 14.1200 6.3720 0.0890

26 24 H62 H1 E 1.0830 13.7810 4.7260 0.0890

27 24 H80 H1 E 2.8320 13.8630 5.0520 0.0890

28 22 H2 H1 E 0.1220 12.2610 6.2760 0.1470

29 22 C1 CT M -0.0100 11.2590 4.4900 0.0060

30 29 H1 H2 E -0.1320 12.1900 3.8390 0.1690

31 29 O8 OS M 0.8190 10.4140 3.7520 -0.2360

32 31 C13 CT M 0.9080 10.6420 2.3220 -0.0070

33 32 C14 CT 3 -0.0810 9.7330 1.5490 0.0200

34 33 O9 OS E 0.2560 9.6760 0.1710 -0.3150

35 33 C15 CT 3 -1.5290 10.2890 1.5950 -0.0170

36 35 O10 OS S -2.3730 9.3990 0.9330 -0.3000

37 36 C18 CT 3 -3.7110 9.8460 0.9520 0.0030

38 37 H16 H1 E -4.3390 9.0920 1.4260 0.0550

39 37 H67 H1 E -3.7760 10.7770 1.5140 0.0550

40 37 H85 H1 E -4.0530 10.0130 -0.0690 0.0550

41 35 H33 H1 E -1.8280 10.2960 2.5680 0.0860

42 35 H34 H1 E -1.6450 11.1950 1.1120 0.0860

43 33 H32 H1 E -0.0710 8.8050 2.0120 0.1480

44 32 H31 H1 E 0.6940 11.6450 2.1320 0.1440

45 32 C12 CT M 2.3590 10.3550 1.9730 -0.0350

46 45 O7 OS S 3.1520 11.4010 2.4640 -0.3450

47 46 C17 CT 3 4.1690 10.9540 3.4240 -0.0150

48 47 H15 H1 E 4.4860 9.9420 3.1730 0.0650

49 47 H66 H1 E 3.7500 10.9650 4.4300 0.0650

50 47 H84 H1 E 5.0280 11.6240 3.3810 0.0650

51 45 H30 H1 E 2.7110 9.5620 2.4490 0.1660

52 45 C11 CT M 2.5230 10.2840 0.4510 0.0310

53 52 O6 OS S 3.8570 9.9980 0.1030 -0.2340

54 53 C16 CT 3 4.4320 10.8810 -0.7750 -0.1270

55 54 H14 H1 E 4.4950 11.8660 -0.3120 0.0890

56 54 H65 H1 E 5.4340 10.5360 -1.0320 0.0890

57 54 H83 H1 E 3.8260 10.9420 -1.6790 0.0890

58 52 H9 H1 E 2.3980 11.1720 0.0130 0.1470

59 52 C10 CT M 1.5680 9.2500 -0.1170 0.0060

60 59 H8 H2 E 1.6690 9.1360 -1.1120 0.1690

61 59 O13 OS M 1.8230 7.9810 0.4830 -0.2360

62 61 C22 CT M 1.9260 6.8210 -0.3380 -0.0070

63 62 C23 CT 3 0.6400 6.0210 -0.3470 0.0200

64 63 O14 OS E 0.8340 4.8020 -1.1080 -0.3150

65 63 C24 CT 3 -0.4970 6.7600 -1.1540 -0.0170

66 65 O15 OS S -1.7470 6.0120 -0.9070 -0.3000

67 66 C27 CT 3 -2.8770 6.6060 -1.0750 0.0030

68 67 H19 H1 E -2.7620 7.6710 -0.8750 0.0550

69 67 H70 H1 E -3.6130 6.1860 -0.3900 0.0550

70 67 H88 H1 E -3.2140 6.4630 -2.1020 0.0550

71 65 H40 H1 E -0.6050 7.7160 -0.7680 0.0860

72 65 H41 H1 E -0.2130 6.8170 -2.3430 0.0860

73 63 H39 H1 E 0.4270 5.8230 0.7020 0.1480

74 62 H38 H1 E 2.1730 7.1010 -1.2840 0.1440

75 62 C21 CT M 3.0580 5.9620 0.2360 -0.0350

76 75 O12 OS S 4.3230 6.6510 0.1270 -0.3450

77 76 C26 CT 3 4.8920 6.9940 1.4390 -0.0150

78 77 H18 H1 E 5.1620 6.0800 1.9690 0.0650

79 77 H69 H1 E 4.1560 7.5480 2.0210 0.0650

80 77 H87 H1 E 5.7810 7.6080 1.2990 0.0650

81 75 H37 H1 E 2.8590 5.8230 1.3370 0.1660

82 75 C20 CT M 3.1890 4.6490 -0.4830 0.0310

83 82 O11 OS S 4.1210 3.8010 0.1500 -0.2340

84 83 C25 CT 3 5.3080 3.5550 -0.5200 -0.1270

85 84 H17 H1 E 6.1020 3.3740 0.2040 0.0890

86 84 H68 H1 E 5.5660 4.4190 -1.1330 0.0890

87 84 H86 H1 E 5.1910 2.6780 -1.1570 0.0890

88 82 H36 H1 E 3.6710 4.8050 -1.4030 0.1470

89 82 C19 CT M 1.7720 3.9010 -0.5440 0.0060

90 89 H35 H2 E 1.6580 2.9820 -1.1920 0.1690

91 89 O18 OS M 1.3920 3.5050 0.7350 -0.2360

92 91 C31 CT M 0.7490 2.2110 0.8530 -0.0070

93 92 C32 CT 3 -0.6510 2.4280 1.4590 0.0200

94 93 O19 OS E -1.1760 1.1760 1.9040 -0.3150

95 93 C33 CT 3 -1.6970 2.9870 0.4810 -0.0170

96 95 O20 OS S -1.7350 2.1400 -0.6470 -0.3000

97 96 C36 CT 3 -2.8090 2.6180 -1.5560 0.0030

98 97 H22 H1 E -2.4280 3.4310 -2.1740 0.0550

99 97 H73 H1 E -3.1370 1.7980 -2.1950 0.0550

100 97 H91 H1 E -3.6520 2.9770 -0.9650 0.0550

101 95 H47 H1 E -1.5140 3.9290 0.2120 0.0860

102 95 H48 H1 E -2.8740 3.0770 0.9140 0.0860

103 93 H46 H1 E -0.5490 3.1010 2.3300 0.1480

104 92 H45 H1 E 0.6970 1.7750 -0.1190 0.1440

105 92 C30 CT M 1.6550 1.3400 1.7580 -0.0350

106 105 O17 OS S 2.7900 0.9890 0.9840 -0.3450

107 106 C35 CT 3 4.0700 1.3370 1.7570 -0.0150

108 107 H21 H1 E 4.8330 1.6830 1.0590 0.0650

109 107 H72 H1 E 4.4310 0.4520 2.2800 0.0650

110 107 H90 H1 E 3.8560 2.1240 2.4800 0.0650

111 105 H44 H1 E 2.0950 1.8460 2.5290 0.1660

112 105 C29 CT M 0.9300 0.1230 2.2610 0.0310

113 112 O16 OS S 1.7340 -0.5750 3.1630 -0.2340

114 113 C34 CT 3 1.4340 -1.9380 3.3140 -0.1270

115 114 H20 H1 E 0.4740 -2.1520 2.8440 0.0890

116 114 H71 H1 E 1.3810 -2.1840 4.3750 0.0890

117 114 H89 H1 E 2.2120 -2.5370 2.8400 0.0890

118 112 H43 H1 E 0.7920 -0.5680 1.5760 0.1470

119 112 C28 CT M -0.3860 0.5250 2.9030 0.0060

120 119 H42 H2 E -0.8200 -0.3790 3.2300 0.1690

121 119 O23 OS M -0.1610 1.4080 3.9850 -0.2360

122 121 C40 CT M -0.9470 1.3020 5.1650 -0.0070

123 122 C41 CT 3 -1.8390 2.5040 5.3040 0.0200

124 123 O24 OS E -2.4280 2.5490 6.5990 -0.3150

125 123 C42 CT 3 -3.0920 2.3670 4.3360 -0.0170

126 125 O25 OS S -3.7130 3.6210 4.2940 -0.3000

127 126 C45 CT 3 -4.9120 3.5690 3.4110 0.0030

128 127 H27 H1 E -5.2680 2.5410 3.3410 0.0550

129 127 H76 H1 E -4.6480 3.9310 2.4170 0.0550

130 127 H94 H1 E -5.6980 4.1970 3.8290 0.0550

131 125 H24 H1 E -3.8250 1.6330 4.6210 0.0860

132 125 H53 H1 E -2.8320 2.2250 3.3630 0.0860

133 123 H52 H1 E -1.2650 3.3140 5.1370 0.1480

134 122 H51 H1 E -1.5510 0.4500 5.1630 0.1440

135 122 C39 CT M 0.0860 1.2280 6.3250 -0.0350

136 135 O22 OS S 0.7060 -0.0470 6.2870 -0.3450

137 136 C44 CT 3 2.1870 0.0070 6.3750 -0.0150

138 137 H26 H1 E 2.5680 0.7210 5.6450 0.0650

139 137 H75 H1 E 2.6000 -0.9800 6.1670 0.0650

140 137 H93 H1 E 2.4810 0.3200 7.3770 0.0650

141 135 H50 H1 E 0.9550 1.9650 6.1700 0.1660

142 135 C38 CT M -0.6280 1.4130 7.6840 0.0310

143 142 O21 OS S 0.3540 1.4890 8.6880 -0.2340

144 143 C43 CT 3 -0.0200 1.0110 9.9320 -0.1270

145 144 H25 H1 E 0.0530 1.8130 10.6670 0.0890

146 144 H74 H1 E 0.6390 0.1920 10.2200 0.0890

147 144 H92 H1 E -1.0480 0.6520 9.8880 0.0890

148 142 H23 H1 E -1.1140 0.5680 7.9570 0.1470

149 142 C37 CT M -1.5060 2.6750 7.6780 0.0060

150 149 H49 H2 E -2.0630 2.7690 8.6450 0.1690

151 149 O28 OS M -0.6810 3.8230 7.4510 -0.2360

152 151 C49 CT M -0.8700 4.9590 8.3180 -0.0070

153 152 H57 H1 E -1.2370 4.6860 9.2540 0.1440

154 152 C48 CT M 0.5240 5.5670 8.5400 -0.0350

155 154 O27 OS S 1.2770 4.7010 9.3590 -0.3450

156 155 C53 CT 3 2.4120 4.0780 8.8100 -0.0150

157 156 H29 H1 E 2.6660 4.5540 7.8630 0.0650

158 156 H78 H1 E 2.2020 3.0220 8.6410 0.0650

159 156 H96 H1 E 3.2490 4.1750 9.5010 0.0650

160 154 H56 H1 E 1.1160 5.6570 7.6790 0.1660

161 154 C47 CT M 0.4150 6.9440 9.2230 0.0310

162 161 O26 OS S 1.6880 7.5340 9.2360 -0.2340

163 162 C52 CT 3 2.1710 7.8790 10.4860 -0.1270

164 163 H28 H1 E 2.4400 6.9760 11.0340 0.0890

165 163 H77 H1 E 1.4030 8.4230 11.0360 0.0890

166 163 H95 H1 E 3.0520 8.5110 10.3730 0.0890

167 161 H55 H1 E 0.1590 6.9820 10.2870 0.1470

168 161 C46 CT M -0.6220 7.8440 8.5470 0.0060

169 168 H54 H2 E -0.7410 8.7580 9.1490 0.1690

170 168 O29 OS M -1.8610 7.1790 8.4490 -0.3150

171 170 C50 CT M -1.8420 5.9390 7.6470 0.0200

172 171 H58 H1 E -1.4590 6.0360 6.6330 0.1480

173 171 C51 CT M -3.2940 5.5170 7.5390 -0.0170

174 173 H59 H1 E -4.0180 6.4140 7.3480 0.0860

175 173 H60 H1 E -3.3910 4.8050 6.8450 0.0860

176 173 O30 OS M -3.7270 5.0300 8.7840 -0.3000

177 176 C54 CT M -5.1550 5.0790 8.8900 0.0030

178 177 H10 H1 E -5.4850 4.4000 9.6770 0.0550

179 177 H61 H1 E -5.4670 6.0950 9.1310 0.0550

180 177 H79 H1 E -5.6000 4.7780 7.9410 0.0550

JEJWOKg

1 0 C7 CT M 5.4230 7.7540 5.5710 -0.2910

2 1 H7 HC E 5.6320 8.7720 5.8990 0.0740

3 1 H8 HC E 5.1230 7.7630 4.5230 0.0740

4 1 H9 HC E 6.3190 7.1450 5.6870 0.0740

5 1 C4 CT M 4.3430 7.1910 6.3830 0.3160

6 5 O1 OH S 3.6020 8.1970 7.1710 -0.6770

7 6 H10 HO E 2.8800 7.6950 7.8150 0.4260

8 5 H3 H1 E 4.9860 6.7460 7.0830 0.0780

9 5 C1 CA M 3.3200 6.3950 5.5180 -0.0280

10 9 C2 CA M 3.4940 5.0860 5.3540 -0.1430

11 10 H1 HA E 4.6570 4.5920 5.6270 0.1330

12 10 C5 CA M 2.6960 4.2960 4.6260 -0.1530

13 12 H4 HA E 2.7310 3.1240 4.6210 0.1350

14 12 C8 CA M 1.6480 4.9040 3.9850 -0.1190

15 14 H6 HA E 1.0470 4.4260 3.4290 0.1300

16 14 C6 CA M 1.4040 6.2530 4.0550 -0.1530

17 16 H5 HA E 0.5600 6.6980 3.3500 0.1350

18 16 C3 CA M 2.3020 7.0150 4.8660 -0.1430

19 18 H2 HA E 2.1840 8.0240 4.8990 0.1330

JESCALh

1 0 O1 OS M 8.6420 13.4690 -0.8500 -0.4060

2 1 C1 CT M 9.5410 13.5500 0.2650 0.1150

3 2 C4 CT B 8.8520 14.3110 1.3970 -0.0350

4 3 H3 HC E 8.7460 15.2180 1.1430 0.0460

5 3 H4 HC E 9.3880 14.2580 2.1780 0.0460

6 2 H1 H1 E 10.3220 14.0250 0.0090 0.0930

7 2 C3 CA M 10.0140 12.1780 0.6960 -0.0100

8 7 C7 CA M 11.1730 12.0710 1.4770 -0.2200

9 8 H7 HA E 11.6370 12.8570 1.7310 0.1460

10 8 C11 CA M 11.6470 10.8390 1.8840 -0.2120

11 10 H9 HA E 12.4450 10.7730 2.3970 0.1780

12 10 C15 CB M 10.9460 9.6960 1.5310 -0.0330

13 12 C18 CB S 11.0760 8.2840 1.7840 0.0420

14 13 C19 CB B 9.9830 7.6790 1.1750 0.2350

15 14 O2 OS E 9.1800 8.6160 0.5320 -0.2820

16 14 C23 CA B 9.7080 6.3430 1.2270 -0.2250

17 16 C27 CA B 10.5950 5.5520 1.9310 -0.1230

18 17 C26 CA B 11.7220 6.1140 2.5290 -0.2140

19 18 C22 CA S 11.9710 7.4640 2.4770 -0.1800

20 19 H15 HA E 12.7330 7.8330 2.9030 0.1620

21 18 H19 HA E 12.3330 5.5440 2.9850 0.1570

22 17 H20 HA E 10.4390 4.6180 2.0030 0.1470

23 16 H16 HA E 8.9380 5.9750 0.8020 0.1620

24 12 C12 CB M 9.8010 9.8470 0.7750 0.1810

25 24 C8 CA M 9.2950 11.0530 0.3180 0.0270

26 25 C6 CT M 8.0720 11.1510 -0.5580 -0.0780

27 26 H5 HC E 8.2970 10.8900 -1.4430 0.0570

28 26 H6 HC E 7.3980 10.5720 -0.2190 0.0570

29 26 C2 CT M 7.5520 12.5840 -0.5690 0.1450

30 29 H2 H1 E 6.8930 12.6690 -1.2490 0.0880

31 29 C5 CA M 6.8550 12.9410 0.7130 -0.0100

32 31 C10 CA S 5.5700 12.4470 0.9730 -0.2320

33 32 H8 HA E 5.1320 11.9250 0.3100 0.1390

34 31 C9 CA M 7.4870 13.7170 1.6930 0.0370

35 34 C13 CA M 6.8530 13.9370 2.9010 -0.2320

36 35 H10 HA E 7.2990 14.4390 3.5720 0.1390

37 35 C16 CA M 5.5760 13.4370 3.1530 0.0010

38 37 C14 CA M 4.9160 12.7010 2.1750 0.0370

39 38 C17 CT M 3.5250 12.1780 2.4220 -0.0350

40 39 H11 HC E 2.8930 12.7440 1.9960 0.0460

41 39 H12 HC E 3.4500 11.2970 2.0680 0.0460

42 39 C21 CT M 3.2400 12.1430 3.9170 0.1150

43 42 H14 H1 E 2.3230 11.9340 4.0590 0.0930

44 42 O3 OS M 3.5130 13.4330 4.4670 -0.4040

45 44 C20 CT M 4.9290 13.6780 4.4930 0.1370

46 45 H13 H1 E 5.0920 14.5930 4.6820 0.0880

47 45 C24 CT M 5.5400 12.8190 5.5940 -0.0780

48 47 H17 HC E 5.2410 13.1330 6.4400 0.0570

49 47 H18 HC E 6.4850 12.8720 5.5500 0.0570

50 47 C28 CA M 5.1120 11.3930 5.4160 0.0280

51 50 C25 CA M 4.0160 11.0490 4.6100 -0.0100

52 51 C29 CA M 3.6440 9.7130 4.4430 -0.2200

53 52 H21 HA E 2.9000 9.5070 3.8920 0.1460

54 52 C31 CA M 4.3250 8.6840 5.0530 -0.2120

55 54 H22 HA E 4.0530 7.7800 4.9230 0.1780

56 54 C32 CB M 5.4160 8.9920 5.8620 -0.0190

57 56 C30 CB M 5.7530 10.3260 6.0160 0.1630

58 57 O4 OS M 6.8640 10.4890 6.8450 -0.2860

59 58 C33 CB M 7.2080 9.1930 7.2150 0.2480

60 59 C34 CB M 6.3800 8.2460 6.6420 0.0360

61 60 C36 CA M 6.6220 6.8990 6.8920 -0.1880

62 61 H24 HA E 6.0710 6.2230 6.5110 0.1610

63 61 C38 CA M 7.6860 6.5680 7.7060 -0.1920

64 63 H26 HA E 7.8670 5.6550 7.8860 0.1530

65 63 C37 CA M 8.4980 7.5430 8.2700 -0.1440

66 65 H25 HA E 9.2180 7.2780 8.8310 0.1530

67 65 C35 CA M 8.2770 8.9040 8.0260 -0.2240

68 67 H23 HA E 8.8250 9.5820 8.3980 0.1640

JESCALg

1 0 O1 O M 6.5040 7.4590 2.5150 -0.4250

2 1 N1 NO M 6.8540 8.6010 2.7120 0.7500

3 2 O2 O E 6.3330 9.5610 2.2080 -0.4250

4 2 C1 CA M 7.9910 8.8130 3.6220 -0.0020

5 4 C3 CA S 8.6120 7.7090 4.1820 -0.0990

6 5 H2 HA E 8.3070 6.8200 4.0150 0.2000

7 4 C2 CA M 8.3920 10.1190 3.8430 -0.0990

8 7 H1 HA E 7.9410 10.8580 3.4490 0.2000

9 7 C4 CA M 9.4790 10.2830 4.6640 -0.0020

10 9 N2 NO B 9.9340 11.6520 4.9310 0.7500

11 10 O3 O E 9.3040 12.5710 4.4970 -0.4250

12 10 O4 O E 10.9630 11.7760 5.5280 -0.4250

13 9 C6 CA M 10.1540 9.2400 5.2480 -0.0990

14 13 H3 HA E 10.9070 9.3900 5.8070 0.2000

15 13 C5 CA M 9.6910 7.9680 4.9900 -0.0020

16 15 N3 NO M 10.4050 6.8370 5.6090 0.7500

17 16 O6 O E 9.9610 5.7190 5.4210 -0.4250

18 16 O5 O M 11.3900 7.0810 6.2730 -0.4250

JIVMEGh

1 0 N1 NB M 0.5180 -0.7610 1.7830 -0.5390

2 1 C1 CC M -0.1570 -1.7320 2.4920 0.3172

3 2 C2 C* M 0.5970 -2.1110 3.6620 -0.2977

4 3 H1 HA E 0.3210 -2.7870 4.3610 0.1296

5 3 C4 C* M 1.7240 -1.3740 3.6680 -0.2977

6 5 H2 HA E 2.4580 -1.4020 4.3570 0.1296

7 5 C7 CC M 1.6790 -0.5360 2.4960 0.3172

8 7 C11 CD M 2.7080 0.3310 2.1240 -0.1342

9 8 C17 CA S 3.8480 0.5660 3.0600 0.0177

10 9 C24 CA B 5.1580 0.2280 2.7230 -0.0664

11 10 C31 CA B 6.2170 0.5720 3.5660 -0.1900

12 11 C36 CA B 5.9750 1.2760 4.7420 -0.1629

13 12 C32 CA B 4.6700 1.5950 5.0870 -0.1900

14 13 C25 CA S 3.6180 1.2270 4.2680 -0.0664

15 14 H12 HA E 2.6670 1.4400 4.5320 0.0998

16 13 H17 HA E 4.4780 2.0980 5.9460 0.1132

17 12 H20 HA E 6.7470 1.5410 5.3270 0.1071

18 11 H16 HA E 7.1520 0.2930 3.3200 0.1132

19 10 H11 HA E 5.3450 -0.2580 1.8690 0.0998

20 8 C16 CC M 2.6970 1.0580 0.9270 0.3172

21 20 C23 C* B 3.7100 2.0230 0.5780 -0.2977

22 21 C26 C* S 3.3320 2.5880 -0.5890 -0.2977

23 22 H13 HA E 3.8180 3.3050 -1.1060 0.1296

24 21 H10 HA E 4.5500 2.2250 1.0970 0.1296

25 20 N3 NB M 1.7040 1.0330 -0.0330 -0.5390

26 25 C18 CC M 2.0790 1.9770 -0.9630 0.3172

27 26 C27 CD M 1.3680 2.3200 -2.1210 -0.1342

28 27 C33 CA S 1.9010 3.4330 -2.9640 0.0177

29 28 C37 CA B 1.8570 4.7420 -2.4850 -0.0664

30 29 C41 CA B 2.3150 5.7980 -3.2630 -0.1900

31 30 C44 CA B 2.8290 5.5500 -4.5270 -0.1629

32 31 C42 CA B 2.9000 4.2540 -5.0070 -0.1900

33 32 C38 CA S 2.4390 3.1980 -4.2290 -0.0664

34 33 H22 HA E 2.4930 2.2590 -4.5860 0.0998

35 32 H26 HA E 3.2950 4.0740 -5.9150 0.1132

36 31 H28 HA E 3.1660 6.3190 -5.0780 0.1071

37 30 H25 HA E 2.2600 6.7390 -2.9180 0.1132

38 29 H21 HA E 1.4900 4.9280 -1.5630 0.0998

39 27 C19 CC M 0.1570 1.7320 -2.4920 0.3172

40 39 C28 C* B -0.5970 2.1110 -3.6620 -0.2977

41 40 C29 C* S -1.7240 1.3740 -3.6680 -0.2977

42 41 H15 HA E -2.4580 1.4020 -4.3570 0.1296

43 40 H14 HA E -0.3210 2.7870 -4.3610 0.1296

44 39 N4 NB M -0.5180 0.7610 -1.7830 -0.5390

45 44 C20 CC M -1.6790 0.5360 -2.4960 0.3172

46 45 C22 CD M -2.7080 -0.3310 -2.1240 -0.1342

47 46 C30 CA S -3.8480 -0.5660 -3.0600 0.0177

48 47 C34 CA B -5.1580 -0.2280 -2.7230 -0.0664

49 48 C39 CA B -6.2170 -0.5720 -3.5660 -0.1900

50 49 C43 CA B -5.9750 -1.2760 -4.7420 -0.1629

51 50 C40 CA B -4.6700 -1.5950 -5.0870 -0.1900

52 51 C35 CA S -3.6180 -1.2270 -4.2680 -0.0664

53 52 H19 HA E -2.6670 -1.4400 -4.5320 0.0998

54 51 H24 HA E -4.4780 -2.0980 -5.9460 0.1132

55 50 H27 HA E -6.7470 -1.5410 -5.3270 0.1071

56 49 H23 HA E -7.1520 -0.2930 -3.3200 0.1132

57 48 H18 HA E -5.3450 0.2580 -1.8690 0.0998

58 46 C14 CC M -2.6970 -1.0580 -0.9270 0.3172

59 58 C15 C* B -3.7100 -2.0230 -0.5780 -0.2977

60 59 C10 C* S -3.3320 -2.5880 0.5890 -0.2977

61 60 H5 HA E -3.8180 -3.3050 1.1060 0.1296

62 59 H8 HA E -4.5500 -2.2250 -1.0970 0.1296

63 58 N2 NB M -1.7040 -1.0330 0.0330 -0.5390

64 63 C6 CC M -2.0790 -1.9770 0.9630 0.3172

65 64 C3 CD M -1.3680 -2.3200 2.1210 -0.1342

66 65 C5 CA M -1.9010 -3.4330 2.9640 0.0177

67 66 C8 CA M -1.8570 -4.7420 2.4850 -0.0664

68 67 H3 HA E -1.4900 -4.9280 1.5630 0.0998

69 67 C12 CA M -2.3150 -5.7980 3.2630 -0.1900

70 69 H6 HA E -2.2600 -6.7390 2.9180 0.1132

71 69 C21 CA M -2.8290 -5.5500 4.5270 -0.1629

72 71 H9 HA E -3.1660 -6.3190 5.0780 0.1071

73 71 C13 CA M -2.9000 -4.2540 5.0070 -0.1900

74 73 H7 HA E -3.2950 -4.0740 5.9150 0.1132

75 73 C9 CA M -2.4390 -3.1980 4.2290 -0.0664

76 75 H4 HA E -2.4930 -2.2590 4.5860 0.0998

77 75 Fe1 Fe M 0.0000 0.0000 0.0000 2.0000

JIVMEGg

1 0 C2 CA M -0.7410 3.4770 2.3800 -0.4193

2 1 H2 HA E -0.2280 3.9390 3.1150 0.1538

3 1 C1 CA M -0.2680 2.2880 1.8770 0.3519

4 3 H1 H4 E 0.5630 1.9020 2.2730 0.0502

5 3 N1 NC M -0.8760 1.6030 0.8820 -0.6069

6 5 C4 CA M -2.0230 2.1350 0.4130 0.3519

7 6 H3 H4 E -2.5180 1.6430 -0.3190 0.0502

8 6 C5 CA M -2.5460 3.3380 0.8560 -0.4193

9 8 H7 HA E -3.3970 3.7140 0.4920 0.1538

10 8 C3 CA M -1.9010 4.0460 1.8570 0.3539

11 10 C6 CT M -2.4570 5.3760 2.3670 -0.2809

12 11 H4 HC E -3.1450 5.2110 2.9690 0.0869

13 11 H5 HC E -2.8200 5.8960 1.5950 0.0869

14 11 H6 HC E -1.7350 5.8820 2.8150 0.0869

JIVMUWh

1 0 N1 NB M 0.9980 -1.4170 1.0890 -0.5390

2 1 C1 CC M 0.5400 -2.6720 1.4050 0.3172

3 2 C2 C* M 1.4880 -3.3160 2.2960 -0.2977

4 3 H1 HA E 1.4070 -4.2350 2.6960 0.1296

5 3 C4 C* M 2.5060 -2.4340 2.4910 -0.2977

6 5 H2 HA E 3.3310 -2.5850 3.0480 0.1296

7 5 C7 CC M 2.2010 -1.2550 1.7450 0.3172

8 7 C11 CD M 3.0000 -0.0930 1.7030 -0.1342

9 8 C17 CA S 4.3420 -0.1620 2.3780 0.0177

10 9 C24 CA B 5.4170 -0.7750 1.7310 -0.0664

11 10 C31 CA B 6.6750 -0.8050 2.3180 -0.1900

12 11 C36 CA B 6.8650 -0.2240 3.5660 -0.1629

13 12 C32 CA B 5.7930 0.3880 4.2130 -0.1900

14 13 C25 CA S 4.5420 0.4150 3.6290 -0.0664

15 14 H12 HA E 3.7730 0.8590 4.1060 0.0998

16 13 H17 HA E 5.9320 0.8090 5.1210 0.1132

17 12 H20 HA E 7.7730 -0.2390 4.0020 0.1071

18 11 H16 HA E 7.4430 -1.2450 1.8360 0.1132

19 10 H11 HA E 5.2700 -1.2020 0.8340 0.0998

20 8 C16 CC M 2.6660 1.1130 1.0640 0.3172

21 20 C23 C* B 3.4870 2.2960 1.0580 -0.2977

22 21 C26 C* S 2.8400 3.2290 0.3160 -0.2977

23 22 H13 HA E 3.1520 4.1640 0.1130 0.1296

24 21 H10 HA E 4.3740 2.4110 1.5250 0.1296

25 20 N3 NB M 1.5200 1.3390 0.3390 -0.5390

26 25 C18 CC M 1.6060 2.6200 -0.1360 0.3172

27 26 C27 CD M 0.6550 3.2490 -0.9620 -0.1342

28 27 C33 CA S 0.9630 4.6570 -1.3910 0.0177

29 28 C37 CA B 0.6200 5.7260 -0.5770 -0.0664

30 29 C41 CA B 0.9220 7.0310 -0.9540 -0.1900

31 30 C44 CA B 1.5620 7.2820 -2.1640 -0.1629

32 31 C42 CA B 1.9260 6.2140 -2.9790 -0.1900

33 32 C38 CA S 1.6360 4.9050 -2.5870 -0.0664

34 33 H22 HA E 1.9110 4.1270 -3.1690 0.0998

35 32 H26 HA E 2.4020 6.3870 -3.8500 0.1132

36 31 H28 HA E 1.7640 8.2310 -2.4480 0.1071

37 30 H25 HA E 0.6670 7.7990 -0.3470 0.1132

38 29 H21 HA E 0.1560 5.5510 0.3000 0.0998

39 27 C19 CC M -0.5400 2.6720 -1.4050 0.3172

40 39 C28 C* B -1.4880 3.3160 -2.2960 -0.2977

41 40 C29 C* S -2.5060 2.4340 -2.4910 -0.2977

42 41 H15 HA E -3.3310 2.5850 -3.0480 0.1296

43 40 H14 HA E -1.4070 4.2350 -2.6960 0.1296

44 39 N4 NB M -0.9980 1.4170 -1.0890 -0.5390

45 44 C20 CC M -2.2010 1.2550 -1.7450 0.3172

46 45 C22 CD M -3.0000 0.0930 -1.7030 -0.1342

47 46 C30 CA S -4.3420 0.1620 -2.3780 0.0177

48 47 C34 CA B -5.4170 0.7750 -1.7310 -0.0664

49 48 C39 CA B -6.6750 0.8050 -2.3180 -0.1900

50 49 C43 CA B -6.8650 0.2240 -3.5660 -0.1629

51 50 C40 CA B -5.7930 -0.3880 -4.2130 -0.1900

52 51 C35 CA S -4.5420 -0.4150 -3.6290 -0.0664

53 52 H19 HA E -3.7730 -0.8590 -4.1060 0.0998

54 51 H24 HA E -5.9320 -0.8090 -5.1210 0.1132

55 50 H27 HA E -7.7730 0.2390 -4.0020 0.1071

56 49 H23 HA E -7.4430 1.2450 -1.8360 0.1132

57 48 H18 HA E -5.2700 1.2020 -0.8340 0.0998

58 46 C14 CC M -2.6660 -1.1130 -1.0640 0.3172

59 58 C15 C* B -3.4870 -2.2960 -1.0580 -0.2977

60 59 C10 C* S -2.8400 -3.2290 -0.3160 -0.2977

61 60 H5 HA E -3.1520 -4.1640 -0.1130 0.1296

62 59 H8 HA E -4.3740 -2.4110 -1.5250 0.1296

63 58 N2 NB M -1.5200 -1.3390 -0.3390 -0.5390

64 63 C6 CC M -1.6060 -2.6200 0.1360 0.3172

65 64 C3 CD M -0.6550 -3.2490 0.9620 -0.1342

66 65 C5 CA M -0.9630 -4.6570 1.3910 0.0177

67 66 C8 CA M -0.6200 -5.7260 0.5770 -0.0664

68 67 H3 HA E -0.1560 -5.5510 -0.3000 0.0998

69 67 C12 CA M -0.9220 -7.0310 0.9540 -0.1900

70 69 H6 HA E -0.6670 -7.7990 0.3470 0.1132

71 69 C21 CA M -1.5620 -7.2820 2.1640 -0.1629

72 71 H9 HA E -1.7640 -8.2310 2.4480 0.1071

73 71 C13 CA M -1.9260 -6.2140 2.9790 -0.1900

74 73 H7 HA E -2.4020 -6.3870 3.8500 0.1132

75 73 C9 CA M -1.6360 -4.9050 2.5870 -0.0664

76 75 H4 HA E -1.9110 -4.1270 3.1690 0.0998

77 75 Zn1 Zn M 0.0000 0.0000 0.0000 2.0000

JIVMUWg

1 0 Cl1 Cl M -0.0380 3.4980 3.0540 -0.1278

2 1 C1 CA M 0.1140 1.9720 3.8980 -0.0656

3 2 C2 CA M -0.4050 0.8310 3.2860 0.3945

4 3 O1 OH S -1.0640 0.8790 2.0760 -0.5965

5 4 H2 HO E -1.1050 1.6790 1.9290 0.4256

6 3 C4 CA M -0.2700 -0.3950 3.9180 -0.2338

7 6 H3 HA E -0.6410 -1.2270 3.4790 0.1735

8 6 C6 CA M 0.3710 -0.4780 5.1540 -0.1417

9 8 H5 HA E 0.4790 -1.3710 5.6120 0.1559

10 8 C5 CA M 0.8650 0.6740 5.7680 -0.1793

11 10 H4 HA E 1.3200 0.6100 6.6600 0.1475

12 10 C3 CA M 0.7300 1.9090 5.1400 -0.1092

13 12 H1 HA E 1.0800 2.7440 5.5780 0.1570

JULJABh

1 0 C21 CA M -0.6940 0.5000 17.5990 -0.1520

2 1 C22 CA B 0.6940 0.5000 17.7030 -0.1520

3 2 C16 CA B 1.3710 1.6750 17.7570 -0.1450

4 3 C10 CA S 0.7090 2.9140 17.7120 0.0560

5 4 C4 CA B 1.4140 4.1460 17.7710 -0.2720

6 5 C2 CB S 0.6940 5.2790 17.7080 0.2190

7 6 O2 OS E 1.1970 6.5800 17.7570 -0.4070

8 5 H2 HA E 2.3130 4.1800 17.8700 0.1970

9 3 H8 HA E 2.3130 1.6720 17.8630 0.1320

10 2 H10 HA E 1.1140 -0.3020 17.6980 0.1450

11 1 H9 HA E -1.1140 -0.3020 17.6040 0.1450

12 1 C15 CA M -1.3710 1.6750 17.5450 -0.1450

13 12 H7 HA E -2.3130 1.6720 17.4390 0.1320

14 12 C9 CA M -0.7090 2.9140 17.5900 0.0560

15 14 C3 CA M -1.4140 4.1460 17.5310 -0.2720

16 15 H1 HA E -2.3130 4.1800 17.4320 0.1970

17 15 C1 CB M -0.6940 5.2790 17.5950 0.2190

18 17 O1 OS M -1.1970 6.5800 17.5450 -0.4070

19 18 P1 PY M 0.0000 7.6420 17.6510 1.3750

20 19 N2 NC E 0.1160 8.4630 16.3210 -0.9200

21 19 N1 NC M -0.1160 8.4630 18.9810 -0.9200

22 21 P2 PY M -0.1270 10.0360 19.0260 1.3750

23 22 O3 OS S -1.4140 10.5580 19.8220 -0.4070

24 23 C5 CB S -1.0400 11.0240 21.0900 0.2190

25 24 C6 CB B 0.3460 11.0130 21.2290 0.2190

26 25 O4 OS E 0.9760 10.5670 20.0580 -0.4070

27 25 C12 CA B 0.9550 11.3960 22.3700 -0.2720

28 27 C18 CA S 0.1300 11.8070 23.4500 0.0560

29 28 C17 CA B -1.2810 11.8150 23.3070 0.0560

30 29 C11 CA S -1.8620 11.4110 22.0780 -0.2720

31 30 H3 HA E -2.7370 11.3890 21.9930 0.1970

32 29 C23 CA B -2.0760 12.2110 24.4060 -0.1450

33 32 C27 CA B -1.5020 12.5650 25.5830 -0.1520

34 33 C28 CA B -0.1200 12.5580 25.7330 -0.1520

35 34 C24 CA S 0.6780 12.1950 24.6980 -0.1450

36 35 H12 HA E 1.5810 12.1840 24.7610 0.1320

37 34 H16 HA E 0.2550 12.8000 26.5710 0.1450

38 33 H15 HA E -2.0480 12.7730 26.3120 0.1450

39 32 H11 HA E -3.0340 12.1560 24.2880 0.1320

40 27 H4 HA E 1.8880 11.3890 22.4640 0.1970

41 22 N3 NC M 0.0000 10.7840 17.6510 -0.9200

42 41 P3 PY M 0.1270 10.0360 16.2760 1.3750

43 42 O6 OS E -0.9760 10.5670 15.2440 -0.4070

44 42 O5 OS M 1.4140 10.5580 15.4800 -0.4070

45 44 C7 CB M 1.0400 11.0240 14.2130 0.2190

46 45 C13 CA S 1.8620 11.4110 13.2240 -0.2720

47 46 H5 HA E 2.7370 11.3890 13.3090 0.1970

48 45 C8 CB M -0.3460 11.0130 14.0740 0.2190

49 48 C14 CA M -0.9550 11.3960 12.9320 -0.2720

50 49 H6 HA E -1.8880 11.3890 12.8380 0.1970

51 49 C20 CA M -0.1300 11.8070 11.8520 0.0560

52 51 C19 CA M 1.2810 11.8150 11.9960 0.0560

53 52 C25 CA M 2.0760 12.2110 10.8970 -0.1450

54 53 H13 HA E 3.0340 12.1560 11.0140 0.1320

55 53 C29 CA M 1.5020 12.5650 9.7200 -0.1520

56 55 H17 HA E 2.0480 12.7730 8.9900 0.1450

57 55 C30 CA M 0.1200 12.5580 9.5690 -0.1520

58 57 H18 HA E -0.2550 12.8000 8.7310 0.1450

59 57 C26 CA M -0.6780 12.1950 10.6050 -0.1450

60 59 H14 HA E -1.5810 12.1840 10.5410 0.1320

JULJABg

1 0 C4 CT M 0.4500 8.5220 9.4300 -0.1250

2 1 H3 HC E 1.1350 8.2500 8.8960 0.0460

3 1 H4 HC E -0.3180 8.5660 8.7790 0.0460

4 1 H5 HC E 0.6580 9.3190 9.6020 0.0460

5 1 C1 CA M 0.2030 7.6680 10.6400 0.1110

6 5 C2 CA M 0.6510 8.0420 11.8850 -0.2110

7 6 H1 HA E 1.1030 8.8400 12.0030 0.1490

8 6 C5 CA M 0.4560 7.2490 12.9890 -0.2110

9 8 H6 HA E 0.7850 7.4830 13.7920 0.1490

10 8 C7 CA M -0.2030 6.0370 12.8950 0.1110

11 10 C8 CT 3 -0.4500 5.1830 14.1050 -0.1250

12 11 H8 HC E -1.1350 5.4550 14.6390 0.0460

13 11 H9 HC E 0.3180 5.1390 14.7560 0.0460

14 11 H10 HC E -0.6580 4.3860 13.9330 0.0460

15 10 C6 CA M -0.6510 5.6630 11.6500 -0.2110

16 15 H7 HA E -1.1030 4.8650 11.5320 0.1490

17 15 C3 CA M -0.4560 6.4560 10.5460 -0.2110

18 17 H2 HA E -0.7850 6.2220 9.7430 0.1490

JUMYOFh

1 0 O5 OH M 0.1560 -15.4420 14.2290 -0.6510

2 1 H13 HO E -0.0710 -16.3450 14.4640 0.4300

3 1 C6 CT M 0.5130 -14.7310 15.3880 0.1630

4 3 H6 H1 E -0.0820 -15.0000 16.0870 0.0640

5 3 H7 H1 E 0.3670 -13.7960 15.2160 0.0640

6 3 C5 CT M 1.9450 -14.9890 15.7530 -0.0230

7 6 O4 OS E 2.0370 -16.3790 16.1220 -0.3480

8 6 H5 H1 E 2.1860 -14.4500 16.5120 0.0980

9 6 C4 CT M 2.9610 -14.7200 14.6340 0.0230

10 9 O6 OS E 2.9910 -13.3100 14.3760 -0.2540

11 9 H4 H1 E 2.7170 -15.2270 13.8360 0.1390

12 9 C3 CT M 4.3470 -15.1050 15.1000 0.1650

13 12 O3 OH S 5.2610 -14.9760 13.9930 -0.6790

14 13 H12 HO E 5.3930 -14.0460 13.7930 0.4430

15 12 H3 H1 E 4.6370 -14.4880 15.8100 0.0760

16 12 C2 CT M 4.3880 -16.5150 15.6360 0.1460

17 16 O2 OH S 5.6620 -16.7810 16.2120 -0.6440

18 17 H11 HO E 5.9920 -17.6210 15.8850 0.4520

19 16 H2 H1 E 4.2520 -17.1180 14.8970 0.0990

20 16 C1 CT M 3.2930 -16.7230 16.6570 0.0670

21 20 H1 H2 E 3.2530 -17.6630 16.9270 0.1700

22 20 O1 OS M 3.5940 -15.9400 17.7810 -0.2540

23 22 C34 CT M 3.4960 -16.5450 19.0790 0.0230

24 23 C35 CT 3 2.3280 -15.9210 19.8570 -0.0230

25 24 O29 OS E 2.3750 -16.4200 21.2100 -0.3480

26 24 C36 CT 3 0.9760 -16.2410 19.3170 0.1630

27 26 O30 OH S 0.7490 -17.6170 19.3620 -0.6510

28 27 H10 HO E 0.7060 -17.9050 20.2770 0.4300

29 26 H59 H1 E 0.2880 -15.7850 19.8350 0.0640

30 26 H60 H1 E 0.8890 -15.9120 18.4120 0.0640

31 24 H58 H1 E 2.4740 -14.9550 19.8940 0.0980

32 23 H57 H1 E 3.3400 -17.4910 18.9980 0.1390

33 23 C33 CT M 4.8030 -16.2680 19.7670 0.1650

34 33 O28 OH S 5.8320 -16.9950 19.0920 -0.6790

35 34 H27 HO E 6.0220 -16.5740 18.2500 0.4430

36 33 H56 H1 E 4.9950 -15.3040 19.7400 0.0760

37 33 C32 CT M 4.7540 -16.6770 21.2200 0.1460

38 37 O27 OH S 5.9540 -16.2760 21.9040 -0.6440

39 38 H26 HO E 6.2700 -17.0010 22.4480 0.4520

40 37 H55 H1 E 4.6920 -17.6450 21.2250 0.0990

41 37 C31 CT M 3.5490 -16.0770 21.9140 0.0670

42 41 H54 H2 E 3.4990 -16.4310 22.8180 0.1700

43 41 O26 OS M 3.7030 -14.6710 22.0200 -0.2540

44 43 C28 CT M 3.0960 -14.0050 23.1610 0.0230

45 44 C29 CT 3 1.7100 -13.4430 22.7810 -0.0230

46 45 O24 OS E 1.3020 -12.3930 23.6860 -0.3480

47 45 C30 CT 3 0.6390 -14.4700 22.9030 0.1630

48 47 O25 OH S -0.5990 -13.9700 22.2820 -0.6510

49 48 H25 HO E -0.9130 -14.6060 21.6350 0.4300

50 47 H52 H1 E 0.8960 -15.3110 22.5010 0.0640

51 47 H53 H1 E 0.4610 -14.6810 23.8480 0.0640

52 45 H51 H1 E 1.7340 -13.1050 21.8800 0.0980

53 44 H50 H1 E 3.0150 -14.6590 23.8900 0.1390

54 44 C27 CT M 4.1000 -12.9250 23.5560 0.1650

55 54 O23 OH S 5.1700 -13.5010 24.2710 -0.6790

56 55 H24 HO E 5.4570 -14.3020 23.8270 0.4430

57 54 H49 H1 E 4.4350 -12.5160 22.7270 0.0760

58 54 C26 CT M 3.4980 -11.8300 24.4150 0.1460

59 58 O22 OH S 4.4020 -10.7450 24.5850 -0.6440

60 59 H23 HO E 4.6170 -10.6500 25.5160 0.4520

61 58 H48 H1 E 3.2720 -12.2120 25.2890 0.0990

62 58 C25 CT M 2.1960 -11.3290 23.8070 0.0670

63 62 H47 H2 E 1.8260 -10.6280 24.3740 0.1700

64 62 O21 OS M 2.5000 -10.7650 22.5480 -0.2540

65 64 C22 CT M 2.0290 -9.4440 22.2730 0.0230

66 65 C23 CT 3 0.9060 -9.4820 21.2740 -0.0230

67 66 O19 OS E 0.5820 -8.1210 20.8590 -0.3480

68 66 C24 CT 3 -0.3790 -10.0600 21.8190 0.1630

69 68 O20 OH S -0.7700 -9.3790 23.0190 -0.6510

70 69 H22 HO E -0.7110 -8.4310 22.8830 0.4300

71 68 H45 H1 E -1.0680 -10.0230 21.1660 0.0640

72 68 H46 H1 E -0.2320 -11.0000 22.0320 0.0640

73 66 H44 H1 E 1.1920 -9.9850 20.4930 0.0980
[truncated: 292,291 more chars]
