# Supplementary material for: Accurate Reproduction of 161 Small-Molecule Complex Crystal Structures using the EUDOC Program: Expanding the Use of EUDOC to Supramolecular Chemistry
Source: PLoS One. 2007 Jun 13;2(6):e531. doi: 10.1371/journal.pone.0000531 (PMC1888730; doi:10.1371/journal.pone.0000531)
Supplement: Table S2 — The docking box size and the interaction energy cutoff used by the EUDOC program for reproducing the 161 host-guest complexes (0.24 MB DOC) [file pone.0000531.s002.doc]

Table S2. The docking box size and the interaction energy cutoff used by the EUDOC program for reproducing the 161 host-guest complexes.

| CSD code* | host atom No. | guest atom No. | R factor | cubic box size (Å) | energy cutoff (kcal/  mol) | CSD code* | host atom No. | guest atom No. | R factor | cubic box size (Å) | energy cutoff (kcal/  mol) |
| --- | --- | --- | --- | --- | --- | --- | --- | --- | --- | --- | --- |
| ABELAU | 48 | 14 | 6.35 | 6 | -10 | HUNXUJ | 50 | 11 | 4.11 | 6 | -10 |
| ABULOZ | 42 | 5 | 5.68 | 6 | -20 | HUNYAQ | 50 | 11 | 9.73 | 6 | -20 |
| ACPHDR | 82 | 24 | 6.50 | 6 | -15 | IKARUH | 72 | 22 | 4.11 | 6 | -5 |
| AHOYEB | 176 | 17 | 6.94 | 6 | -20 | IKUTOX | 124 | 18 | 6.83 | 6 | -5 |
| AJUROM | 108 | 29 | 6.40 | 6 | -15 | INUJAC | 66 | 8 | 6.52 | 6 | -10 |
| AJUXOS | 62 | 10 | 4.21 | 6 | -10 | ITAMIZ | 72 | 28 | 6.74 | 6 | -10 |
| AJUXUY | 42 | 14 | 3.34 | 6 | -10 | IXEKAX | 90 | 20 | 7.35 | 6 | -20 |
| AJUYAF | 62 | 14 | 3.03 | 6 | -10 | IXEKEB | 90 | 14 | 4.11 | 6 | -20 |
| ASOKIC | 70 | 8 | 2.90 | 6 | -10 | JAXPON | 142 | 12 | 3.39 | 6 | -5 |
| ATUKEF | 114 | 18 | 6.13 | 6 | -15 | JEBTAL | 78 | 12 | 7.20 | 6 | -5 |
| AWUGEE | 76 | 12 | 5.26 | 6 | -10 | JEJWOK | 180 | 19 | 7.70 | 6 | -10 |
| AXEZIM | 136 | 16 | 5.38 | 6 | -20 | JESCAL | 68 | 18 | 4.80 | 6 | -20 |
| AYIBEP | 74 | 6 | 3.99 | 6 | -150 | JIVMEG | 77 | 14 | 3.70 | 6 | -10 |
| BAFZEN | 52 | 14 | 5.20 | 6 | -30 | JIVMUW | 77 | 13 | 3.80 | 6 | -10 |
| BAHDEU | 146 | 8 | 6.80 | 6 | -10 | JULJAB | 60 | 18 | 4.10 | 8 | -5 |
| BAKHIE | 42 | 18 | 5.70 | 6 | -15 | JUMYOF | 126 | 13 | 5.50 | 6 | -10 |
| BAMQAH | 50 | 10 | 4.33 | 6 | -20 | KAXPOO | 69 | 29 | 5.60 | 6 | -10 |
| BAPRAM | 42 | 24 | 5.37 | 6 | -10 | KOHJAS | 86 | 13 | 8.30 | 6 | -8 |
| BAPREQ | 42 | 19 | 4.66 | 6 | -10 | KOLMAZ | 78 | 22 | 5.50 | 6 | -40 |
| BAXZAB | 49 | 17 | 4.20 | 6 | -20 | LAYMAZ | 48 | 22 | 4.40 | 6 | -20 |
| BAYXII | 67 | 10 | 7.70 | 6 | -30 | LODNOH01 | 104 | 3 | 5.89 | 6 | -5 |
| BECVEK | 42 | 18 | 4.70 | 6 | -10 | MAFRAN | 96 | 9 | 9.70 | 6 | -13 |
| BEGVOZ | 46 | 15 | 4.74 | 6 | -45 | MEXPEK | 50 | 12 | 4.39 | 6 | -5 |
| BEVHER | 68 | 16 | 6.50 | 6 | -20 | MNPOCB01 | 74 | 17 | 4.30 | 6 | -20 |
| BEVWAA | 62 | 20 | 8.20 | 6 | -20 | MODTII | 136 | 5 | 9.55 | 6 | -100 |
| BIFKIK | 67 | 9 | 5.60 | 6 | -20 | MOZNIY | 124 | 14 | 5.24 | 8 | -5 |
| BOHWUQ | 180 | 14 | 10.00 | 6 | -10 | MUTFEM | 92 | 14 | 11.33 | 6 | -10 |
| CACQED | 147 | 20 | 8.18 | 6 | -10 | NEBQOA | 78 | 32 | 9.59 | 6 | -40 |
| CAWRAT10 | 68 | 17 | 6.00 | 6 | -20 | NEPQUU | 118 | 12 | 7.28 | 6 | -10 |
| CECMEC10 | 180 | 19 | 5.50 | 6 | -20 | NETKOM | 61 | 7 | 5.20 | 6 | -10 |
| CENHAE | 42 | 5 | 2.50 | 4 | -10 | NETKOM01 | 61 | 7 | 7.40 | 6 | -10 |
| CICVUF | 42 | 22 | 4.90 | 6 | -10 | NEXLUX | 50 | 14 | 3.89 | 6 | -10 |
| CIXCOB | 84 | 28 | 6.29 | 6 | -15 | NOHHOH | 78 | 67 | 8.51 | 8 | -20 |
| COBTIW | 66 | 10 | 3.50 | 6 | -30 | NOYNAQ | 92 | 10 | 7.17 | 6 | -10 |
| COXLEG10 | 22 | 17 | 4.80 | 6 | -10 | NUDHOJ | 90 | 36 | 5.86 | 6 | -40 |
| COXQEL | 104 | 14 | 6.30 | 6 | -10 | OBOHAO | 100 | 12 | 4.54 | 6 | -10 |
| COYBOH | 42 | 38 | 6.40 | 6 | -15 | OCAMIO | 99 | 14 | 6.80 | 6 | -10 |
| CRAMCA10 | 42 | 7 | 5.70 | 6 | -30 | QAJKAN | 67 | 16 | 4.45 | 6 | -5 |
| CRAMCB10 | 42 | 6 | 6.60 | 6 | -30 | QAKNAR | 50 | 9 | 6.29 | 6 | -20 |
| CRAMCC10 | 42 | 8 | 8.60 | 6 | -10 | QAKNIZ | 46 | 10 | 6.37 | 6 | -20 |
| CUDXUU | 98 | 12 | 8.20 | 6 | -8 | QAKNOF | 42 | 10 | 6.05 | 6 | -5 |
| CYCBOB | 68 | 6 | 5.10 | 6 | -10 | QATDIY | 144 | 17 | 7.32 | 6 | -10 |
| CYCBOF11 | 85 | 6 | 8.30 | 6 | -10 | RABJIJ | 108 | 13 | 8.60 | 6 | -10 |
| DENFOR | 106 | 12 | 4.20 | 6 | -10 | RACKAH | 130 | 14 | 9.54 | 6 | -20 |
| DERFUB | 88 | 6 | 8.70 | 6 | 0 | RAHWED | 96 | 15 | 4.31 | 6 | -5 |
| DESHEO | 43 | 18 | 6.50 | 6 | -20 | RALQAW01 | 112 | 12 | 4.57 | 6 | -10 |
| DIZTIP | 63 | 7 | 6.00 | 6 | -10 | RIBBUZ | 52 | 6 | 4.88 | 6 | -10 |
| DOXWAO | 74 | 24 | 8.60 | 6 | -30 | RUYWIR | 62 | 18 | 4.49 | 6 | -20 |
| DUGGUH10 | 76 | 8 | 3.60 | 6 | -20 | SAKTII | 111 | 28 | 9.46 | 6 | -40 |
| DUKHUM | 72 | 10 | 7.80 | 6 | -20 | SEPKON | 67 | 37 | 4.00 | 6 | -15 |
| EBASEF | 70 | 16 | 4.32 | 6 | -10 | SEPNEG | 67 | 37 | 4.70 | 6 | -15 |
| EGIRIV | 104 | 6 | 9.46 | 6 | -150 | SOVJIW | 46 | 8 | 5.10 | 6 | -20 |
| EGIROB | 95 | 6 | 9.98 | 6 | -150 | SOVJOC | 42 | 8 | 6.70 | 6 | -20 |
| EMOZOV | 83 | 10 | 4.98 | 6 | -30 | TONFOR | 62 | 11 | 6.60 | 6 | -10 |
| EMOZUB | 84 | 8 | 6.17 | 6 | -10 | UBESOJ | 39 | 16 | 5.79 | 6 | -10 |
| EZAVOQ | 210 | 29 | 11.08 | 6 | -20 | UBETAW | 66 | 27 | 4.62 | 6 | -20 |
| EZUMER | 104 | 12 | 5.58 | 6 | -10 | UBETEA | 62 | 16 | 7.08 | 6 | -20 |
| FADCAP | 64 | 5 | 5.08 | 6 | -30 | UBEVAY | 56 | 16 | 6.44 | 6 | -20 |
| FAHDOH | 64 | 17 | 8.40 | 6 | -10 | UFIWAH | 84 | 12 | 4.80 | 6 | -4 |
| FANJAG | 90 | 23 | 7.37 | 6 | -10 | UJEFIY | 78 | 64 | 12.75 | 6 | -40 |
| FIKVIE | 78 | 26 | 7.50 | 6 | -10 | VAFRUP | 72 | 20 | 10.10 | 6 | -20 |
| FIRXOT | 78 | 28 | 8.00 | 6 | -40 | VAKJEX | 72 | 30 | 8.21 | 6 | -20 |
| FODTIB | 53 | 27 | 7.70 | 6 | -20 | VAVLUZ | 42 | 17 | 5.10 | 6 | -10 |
| FUCVAA | 52 | 7 | 7.90 | 6 | -50 | VOHVIX | 62 | 24 | 7.50 | 6 | -20 |
| GAMBIF | 80 | 18 | 10.30 | 6 | -100 | VOTNEX | 72 | 42 | 8.50 | 6 | -30 |
| GIGKEM | 104 | 26 | 13.20 | 6 | -40 | VOTNOH | 72 | 70 | 8.70 | 6 | -40 |
| GIKKEQ | 47 | 14 | 5.80 | 6 | -50 | XAGLOG | 78 | 24 | 6.20 | 6 | -15 |
| GIXNOQ | 64 | 13 | 8.60 | 6 | -10 | XAGMAT | 78 | 26 | 6.08 | 6 | -15 |
| GIYKOO | 64 | 16 | 5.60 | 6 | -10 | XAQJAA | 82 | 16 | 3.85 | 6 | -130 |
| GOBYOL | 44 | 15 | 4.15 | 6 | -10 | XAQJEE | 82 | 16 | 6.29 | 6 | -120 |
| GOKQUS | 104 | 11 | 4.70 | 6 | -5 | XIVVAZ | 70 | 22 | 6.83 | 6 | -60 |
| GUGGUK | 67 | 14 | 4.77 | 6 | -160 | XOFSUG | 130 | 24 | 6.33 | 6 | -15 |
| GUQHUV | 78 | 14 | 5.35 | 6 | -20 | XUGPUK | 63 | 15 | 5.31 | 6 | -5 |
| GUQJEH | 78 | 20 | 4.34 | 6 | -20 | XUTBET | 96 | 17 | 7.29 | 6 | -10 |
| GUQJIL | 72 | 20 | 7.10 | 6 | -20 | YACVEE | 144 | 16 | 7.50 | 6 | -20 |
| HASWUT | 59 | 16 | 5.26 | 6 | -90 | YACVII | 144 | 15 | 6.60 | 6 | -10 |
| HIWNIK | 72 | 20 | 5.05 | 6 | -10 | YAWJIP | 93 | 16 | 5.56 | 6 | -5 |
| HUNWUI | 35 | 11 | 4.87 | 6 | -12 | YOCLUX | 85 | 28 | 11.34 | 6 | -5 |
| HUNXAP | 39 | 11 | 5.22 | 6 | -12 | YONVAY | 89 | 17 | 5.16 | 6 | -10 |
| HUNXIX | 46 | 11 | 5.50 | 6 | -20 | ZESFEI | 52 | 15 | 12.40 | 6 | -20 |
| HUNXOD | 50 | 11 | 2.64 | 6 | -20 |  | | | | | |

* Cambridge Structural Database code
